# Genome-wide study for circulating metabolites identifies 62 loci and reveals novel systemic effects of *LPA*

**DOI:** 10.1038/ncomms11122

**Published:** 2016-03-23

**Authors:** Johannes Kettunen, Ayşe Demirkan, Peter Würtz, Harmen H.M. Draisma, Toomas Haller, Rajesh Rawal, Anika Vaarhorst, Antti J. Kangas, Leo-Pekka Lyytikäinen, Matti Pirinen, René Pool, Antti-Pekka Sarin, Pasi Soininen, Taru Tukiainen, Qin Wang, Mika Tiainen, Tuulia Tynkkynen, Najaf Amin, Tanja Zeller, Marian Beekman, Joris Deelen, Ko Willems van Dijk, Tõnu Esko, Jouke-Jan Hottenga, Elisabeth M van Leeuwen, Terho Lehtimäki, Evelin Mihailov, Richard J. Rose, Anton J.M. de Craen, Christian Gieger, Mika Kähönen, Markus Perola, Stefan Blankenberg, Markku J. Savolainen, Aswin Verhoeven, Jorma Viikari, Gonneke Willemsen, Dorret I. Boomsma, Cornelia M. van Duijn, Johan Eriksson, Antti Jula, Marjo-Riitta Järvelin, Jaakko Kaprio, Andres Metspalu, Olli Raitakari, Veikko Salomaa, P. Eline Slagboom, Melanie Waldenberger, Samuli Ripatti, Mika Ala-Korpela

**Affiliations:** 1 Computational Medicine, Faculty of Medicine, University of Oulu, PO Box 5000, 90014 Oulu, Finland; 2 National Institute for Health and Welfare, PO Box 30, FI-00271 Helsinki, Finland; 3 NMR Metabolomics Laboratory, School of Pharmacy, University of Eastern Finland, Yliopistonranta 1C, Kuopio 70210, Finland; 4 Biocenter Oulu, University of Oulu, PO Box 5000, FI-90014 Oulu, Finland; 5 Department of Human Genetics, Leiden University Medical Center, PO Box 9600, 2300 RC Leiden, The Netherlands; 6 Department of Epidemiology, Erasmus Medical Center, PO Box 2040, 3000 CA Rotterdam, The Netherlands; 7 Department of Biological Psychology, VU University Amsterdam, Van der Boechorststraat 1, Room 2B-29, 1081 BT Amsterdam, The Netherlands; 8 EMGO Institute for Health and Care Research, Van der Boechorststraat 7, 1081BT Amsterdam, The Netherlands; 9 Neuroscience Campus Amsterdam, De Boelelaan 1085, 1081HV Amsterdam, The Netherlands; 10 Estonian Genome Center, University of Tartu, Riia 23b, 51010 Tartu, Estonia; 11 Research Unit of Molecular Epidemiology, Helmholtz Zentrum München, Ingolstädter Landstraße 1, 85764 Neuherberg, Germany; 12 Institute of Epidemiology II, Helmholtz Zentrum München, Ingolstädter Landstraße 1, 85764 Neuherberg, Germany; 13 Department of Molecular Epidemiology, Leiden University Medical Center, PO Box 9600, 2300 RC Leiden, The Netherlands; 14 Department of Clinical Chemistry, Fimlab Laboratories, University of Tampere School of Medicine, Tampere University, Kalevantie 4, Tampere 33014, Finland; 15 Institute for Molecular Medicine (FIMM), University of Helsinki, Biomedicum 2, Tukholmankatu 8, Helsinki 00290, Finland; 16 Analytic and Translational Genetics Unit, Department of Medicine, Massachusetts General Hospital, 55 Fruit Street, Boston, Massachusetts 02114, USA; 17 Program in Medical and Population Genetics, Broad Institute, 415 Main Street Cambridge, Massachusetts 02142, USA; 18 Department of Genetics, Harvard Medical School, 77 Avenue Louis Pasteur, NRB 0330, Boston, Massachusetts 02115, USA; 19 German Center for Cardiovascular Research (DZHK e.V.), Partner Site Hamburg/Lübeck/Kiel, Martinistraße 52, 20246 Hamburg, Germany; 20 University Heart Center Hamburg, Clinic of general and interventional Cardiology, Martinistraße 52, 20246 Hamburg, Germany; 21 Department of Endocrinology, Leiden University Medical Center, PO Box 9600, 2300 RC Leiden, The Netherlands; 22 Department of Public Health, Hjelt Institute, University of Helsinki, PO Box 41 Mannerheimintie 172, Helsinki 00014, Finland; 23 Department of Psychological and Brain Sciences, Indiana University, 1101 E 10th Street, Bloomington, Indiana 47405, USA; 24 Department of Geriatrics and Gerontology, Leiden University Medical Center, Postzone C7-Q, PO Box 9600, 2300RC Leiden, The Netherlands; 25 Department of Clinical Physiology, University of Tampere and Tampere, University Hospital, PO Box 2000, FIN-33521 Tampere, Finland; 26 Medical Research Center, Internal Medicine, Oulu University Hospital, University of Oulu, Aapistie 5A, Oulu FI-90220, Finland; 27 Center for Proteomics and Metabolomics, Leiden University Medical Center, Albinusdreef 2, 2333 ZA Leiden, The Netherlands; 28 Department of Medicine, University of Turku and Turku University Hospital, PB 52, 20521 Turku, Finland; 29 Department of General Practice and Primary Health Care, University of Helsinki, PL 20, Tukholmankatu 8B, Helsinki 00029, Finland; 30 Folkhälsan Research Centre, Helsingfors Universitet, PB 63, Helsinki 00014, Finland; 31 Department of Epidemiology and Biostatistics, MRC-PHE Centre for Environment and Health, School of Public Health, Imperial College London, London SW7 2AZ, UK; 32 Center for Life Course and Systems Epidemiology, Faculty of Medicine, University of Oulu, PL 5000, 90014 Oulu, Finland; 33 Unit of Primary Care, Oulu University Hospital, P.O. Box 20, OYS, Oulu 90029, Finland; 34 Department of Mental Health and Substance Abuse Services, National Institute for Health and Welfare, PO Box 30 (Mannerheimintie 166), Helsinki 00300, Finland; 35 Research Centre of Applied and Preventive Cardiovascular Medicine, University of Turku, Kiinamyllynkatu 4-8, Turku 20521, Finland; 36 Department of Clinical Physiology, Turku University Hospital, Kiinamyllynkatu 4-8, Turku 20521, Finland; 37 Human Genetics, Wellcome Trust Sanger Institute, Wellcome Trust Genome Campus, Hinxton CB10 1SA, UK; 38 Oulu University Hospital, Kajaanintie 50, Oulu 90220, Finland; 39 Computational Medicine, School of Social and Community Medicine, University of Bristol, Senate House, Tyndall Avenue, Bristol, Bristol BS8 1TH, UK; 40 Medical Research Council Integrative Epidemiology Unit, University of Bristol, Bristol, Bristol BS8 1TH, UK

## Abstract

Genome-wide association studies have identified numerous loci linked with complex diseases, for which the molecular mechanisms remain largely unclear. Comprehensive molecular profiling of circulating metabolites captures highly heritable traits, which can help to uncover metabolic pathophysiology underlying established disease variants. We conduct an extended genome-wide association study of genetic influences on 123 circulating metabolic traits quantified by nuclear magnetic resonance metabolomics from up to 24,925 individuals and identify eight novel loci for amino acids, pyruvate and fatty acids. The *LPA* locus link with cardiovascular risk exemplifies how detailed metabolic profiling may inform underlying aetiology via extensive associations with very-low-density lipoprotein and triglyceride metabolism. Genetic fine mapping and Mendelian randomization uncover wide-spread causal effects of lipoprotein(a) on overall lipoprotein metabolism and we assess potential pleiotropic consequences of genetically elevated lipoprotein(a) on diverse morbidities via electronic health-care records. Our findings strengthen the argument for safe *LPA*-targeted intervention to reduce cardiovascular risk.

## Abstract (web summary)

Circulating metabolites reflect human health and disease. Here, Kettunen *et al*. perform a genome-wide association study on 123 circulating metabolic traits and identify novel genetic loci influencing systemic metabolism. They also link new molecular pathways with a known cardiovascular risk factor Lp(a).

## Graphical abstract

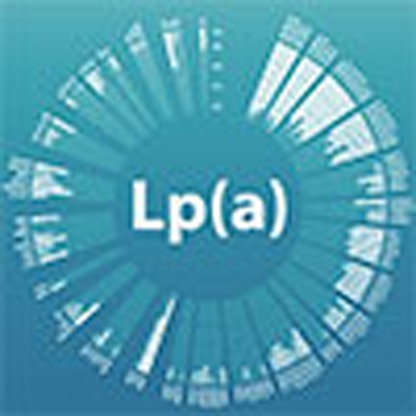

An understanding of the genetic factors involved in systemic metabolism and their associations with chronic disease is a key objective, as large disease consortia have now uncovered numerous variants associated with metabolic diseases^1^,^2^. Metabolic phenotypes serve as good intermediate traits for a genome-wide association study (GWAS) and blood metabolites can be potentially used to discover genetic determinants of circulating metabolites, and particularly to understand the metabolic context of disease-associated genetic variants. Advances in nuclear magnetic resonance (NMR) spectroscopy and mass spectrometry have enabled analytical techniques that can provide hundreds of quantitative metabolic measures from large biological sample collections^3^. GWAS meta-analysis of metabolic measures from these methodologies have been performed, however, the sample sizes have only reached several thousand, which is still modest compared with disease consortia studies^4^,^5^,^6^. The size of previous GWASs utilizing metabolic profiling techniques may partially explain the modest new biological insight added for known disease-associated variants.

To overcome the challenge of small sample size, we perform an expanded GWAS from our previous study^4^ by combining up to 24,925 individuals in a meta-analysis of 123 metabolic measures. We discover eight new loci for circulating metabolites. We focus on a new metabolite association with variants in *LPA*, a known coronary heart disease (CHD) risk locus. We follow up the novel association by constructing a strong genetic risk score for *LPA* and use the risk score for the molecular characterization of the metabolic effects of Lp(a) synthesis and assessment of causality for the metabolic associations. Finally, we perform reverse genetics using electronic health records together with the genetic risk score to test if *LPA* targeting treatment for reducing CHD risk would be associated with potential strong comorbidities. To conclude, we demonstrate how intermediate phenotypes can provide new biological information for known disease loci and how large multi-omics biobank data could be used to inform drug discovery already at an early stage.

## Results

### Genome-wide association study

**Figure 1:**
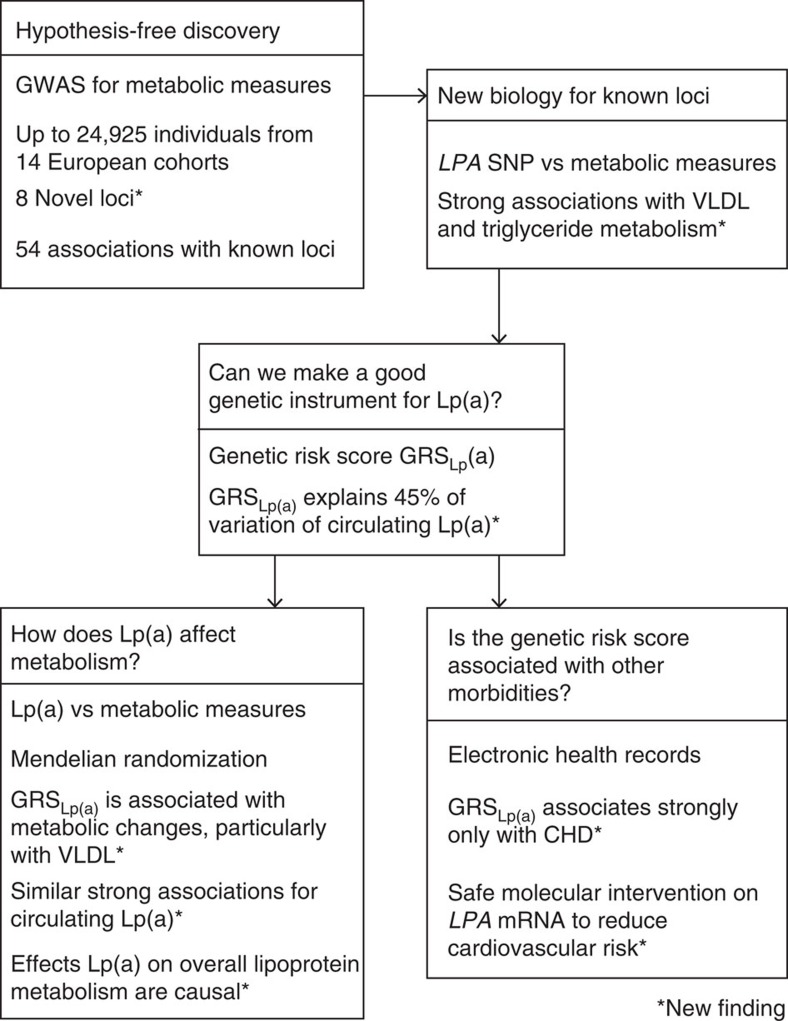
A genome-wide association study for circulating metabolites. Study was conducted to elucidate the genetic variation of systemic metabolism and to discover new metabolic associations in established loci. We also revealed an intriguing novel relation between Lp(a) and systemic triglyceride and VLDL metabolism. Thereby, we highlighted the *LPA* locus and generated the best possible Lp(a) genetic risk score (GRS_Lp(a)_) that enabled us to clarify causal associations between Lp(a) and systemic triglyceride and lipoprotein metabolism. Further, with the aid of extensive electronic health-care records, we were able to use the GRS_Lp(a)_ to show that Lp(a) is associated with ischaemic heart disease but not strongly with other morbidities. Put together, these findings suggest safe molecular intervention on *LPA* to reduce individual cardiovascular risk.

Using the additive genetic model, we tested for univariate associations between genome-wide single-nucleotide polymorphism (SNP) panels imputed to 39 million genetic markers and 123 human blood lipid and metabolite concentrations quantified by high-throughput NMR spectroscopy metabolomics (Supplementary Table 1 for trait information, Methods for analysis details) in 14 genotyped data sets derived from ten European studies (Fig. 1) for up to 24,925 individuals (Table 1 for study characteristics, Supplementary Table 2 for study details and Supplementary Notes 1 for study descriptions). Cohorts were analysed individually and summary statistics were combined in a meta-analysis (Methods). Up to 12,133,295 SNPs, small insertions and deletions were included in the meta-analysis after applying quality control filters. All meta-analysis results are available through URL: http://www.computationalmedicine.fi/data/NMR_GWAS/. To correct for multiple testing, genome- and metabolome-wide statistical significance was set to *P*<2.3 × 10^−9^, where the standard genome-wide significance level (5 × 10^−8^) is divided by the number of principal components (22) that explain over 95% of variation in the metabolomics data. Overall, 62 loci were significantly associated with at least one metabolic measure. Supplementary Fig. 1 presents the associations in 2 Mb windows around the strongest individual variant for the 62 loci. The forest plots for all 62 lead variant associations are shown in Supplementary Fig. 2. We tested if the identified 62 loci harboured additional independent variants. In 9 out of the 62 loci (*PCSK9*, *LPL*, *PPM1K*, *HAL*, *CETP*, *CILP, PLTP, APOB* and *LIPC*), we found a secondary statistically independent association, in 2 of these loci (*APOB* and *LIPC*), we found a third independent variant and *LIPC* additionally harboured a fourth independent variant (Supplementary Table 3 and Methods). The formal conditional testing was first performed in a subset of cohorts and after conditioning with significant variants, the resulting lead variant was then tested using the genomic correlation structure information and summary statistics (Methods). Our correlation structure was obtained from the Finnish population that has longer linkage disequilibrium structure than more outbred populations^7^ and as a result our discovered number of independent variants may be an underestimate. The strength of our approach was to first optimize the variance explained by the next best variant. However, our approach may result in an underestimate of the number of independent variants in a locus, as the variant that explains largest proportion of variance in a trait may be tagging two or more functional variants^8^. In contrast, if the independent variant detection relied only on correlation structure and summary statistics, it may result in a gross overestimate on the number of independent variants in a locus if data are imputed with 1,000 Genomes reference panel. This is because an algorithm based on r-squared between markers does not perform well with rare or low-frequency variants. Overall, this resulted in a total of 74 variants that were independently associated with one or more of the 123 metabolic traits. We estimated the proportion of variance explained by these 74 variants on the metabolic traits (Supplementary Table 1 and Methods). For all, but six, metabolite traits, we observed at least one genome-wide significant association, with the proportion of variance explained ranging from 0.2% for acetoacetate to 12.5% for glycine, with a median of 5%. The average increase in the proportion of variance explained was 1.1% when comparing with our previous study^4^. In 8 of the 62 loci, we found the lead variant to be a non-synonymous substitution. The *SERPINA1* missense variant (rs28929474) had not been identified as the lead variant for the associated metabolite in prior GWAS. The variant had not been available from the HapMap-panel or through genotyping arrays but has become available through 1,000 Genomes imputation. This may explain why we see it as a new lead variant. The *PCSK9* locus also harboured a missense variant as a secondary signal that was independent of the lead variant (Supplementary Table 3). We used the Genotype-Tissue Expression (GTEx) project database to further evaluate if the independent SNPs would be associated with the expression of nearby genes in various human tissues^9^ and the expression quantitative loci (eQTLs) are presented in Supplementary Table 4 (Methods). Although GTEx is still in pilot phase, we were able to link 14 variants with gene expression of nearby genes, and in six loci, our manually curated functional candidate was confirmed as an eQTL (Table 2 and Supplementary Table 4). The eight loci that have not previously been associated with the same or similar metabolic measures in population samples are listed in Table 2. Six of the eight novel loci were associated with the blood concentration of amino acids, one with pyruvate and one with polyunsaturated fatty acids. The glycine decarboxylase (*GLDC*) on chromosome 9q24.1 (rs140348140, *P*=3.7 × 10^−40^) and glycine cleavage system protein H (*GCSH*) on chromosome 16q23.2 (rs10083777, *P*=3.0 × 10^−13^) gene regions showed association with glycine concentrations. In addition, rs10083777 was associated with the expression of *GCSH* in the tibial nerve in the GTEx data (Supplementary Table 5). As a potential limitation, because of GTEx still being in pilot phase, we cannot assess if the variant is also associated with *GCSH* expression in other tissues. Mutations in these two genes have been previously shown to cause Glycine Encephalopathy (OMIM: 605899), a rare recessive disorder of glycine metabolism that manifests as severe early onset neurological complications and is diagnosed by abnormally high glycine concentration in the blood. In this study, we have linked the neuronal expression of *GCSH* and circulating glycine levels with a common variant on the population level.

### Known loci and *LPA* association

**Figure 2:**
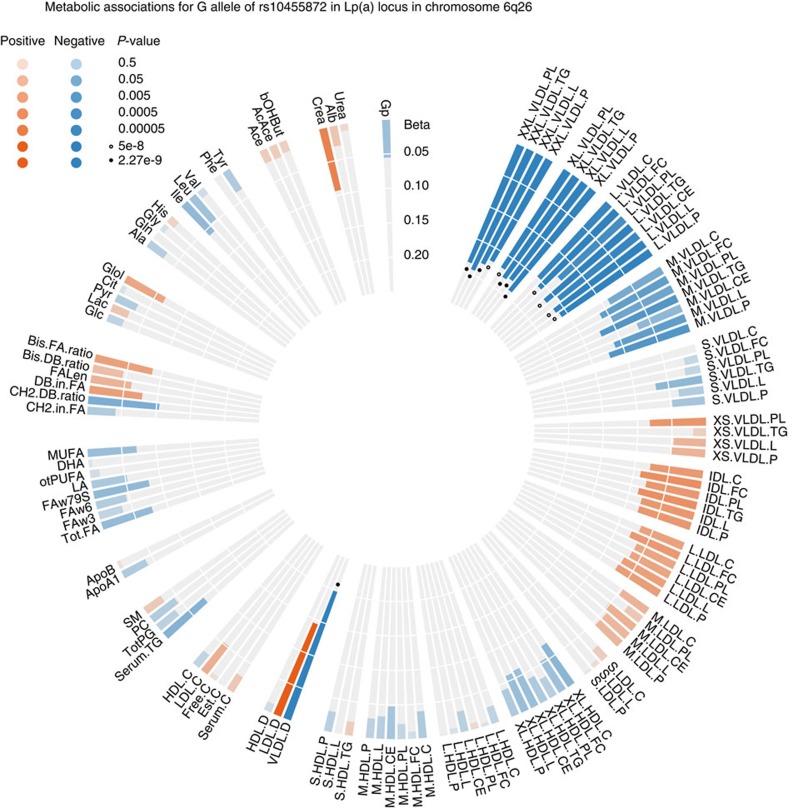
The association pattern of the Lp(a) variant rs10455872 G-allele across all circulating metabolic traits. Each bar represents the association with respective metabolic trait, the size of the bar is the linear regression effect estimate, colouring refers to effect direction and significance is indicated with filled circles for *P*<2.27 × 10^−9^ and unfilled circles for *P*<5 × 10^−8^. Metabolite abbreviations and sample sizes are given in Supplementary Table 1, the strongest association was observed for the mean diameter of very-low-density lipoprotein particles (VLDL.D).

In addition to the new loci discovered, we found significant SNPs spread in 54 loci that have already been associated with the same or related metabolic measures as presented in the catalogue of published GWASs^1^ or recently discovered^5^ (Supplementary Table 4). We then went through the loci that had been associated with similar metabolic traits compared with the prior published findings to pinpoint potential novel biological functions for the already known loci. Here, we noted that the Lp(a)-raising allele rs10455872-G located in the intron of *LPA* was associated with a smaller diameter of very-low-density lipoprotein (VLDL) particles (*P*=1.3 × 10^−12^). This allele was also associated with lower concentrations of extra-large, large and medium VLDL particles (Fig. 2). This metabolic link found between circulating Lp(a) with VLDL metabolism is novel. Lp(a) is thought to be comprised of an low-density lipoprotein (LDL) particle and a covalently bound protein product of the *LPA* gene, apo(a). Although the same variant in the *LPA* locus has been associated with LDL and total cholesterol in over 100,000 individuals^10^, our association in this study had nearly twice the effect estimate for the VLDL associations using the same variant. Both studies had standardized values and compared effect estimates were in standard deviation units. The *LPA* locus is known for its association with CHD risk^11^,^12^ and the genetic variants associated with higher CHD risk are also associated with higher Lp(a) concentrations^13^ making it a potentially important drug target for CHD. Furthermore, the Lp(a) increasing allele rs10455872-G has also been shown to reduce statin response, which implies that LPA targeting treatment could also potentially improve statin efficacy^14^,^15^.

### Genetic risk score for *LPA* and metabolite associations

**Figure 3:**
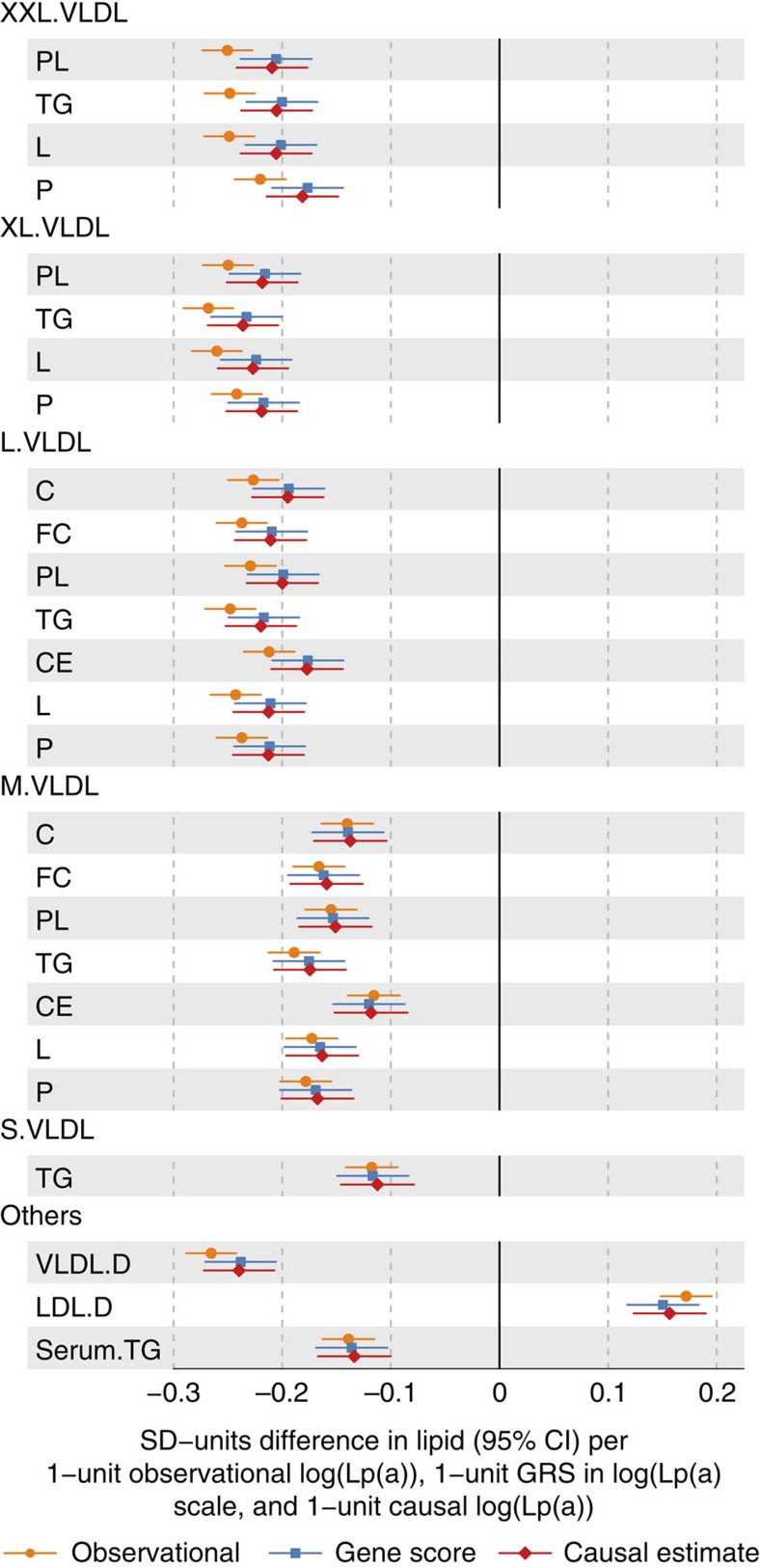
Evaluation of the causative role of the Lp(a) on the circulating metabolic measures via Mendelian randomization. Yellow linear regression estimates are observational associations, blue are GRS_Lp(a)_ estimates and red are the causal effect estimates. Those metabolic traits are listed for which the associations in the meta-analysis were significant with genome-wide threshold (*P*<2.3 × 10^−9^). Metabolite abbreviations are given in Supplementary Table 1.

We have discovered new and stronger metabolic associations for a known important CHD risk locus than identified previously^10^. This intriguing finding directed us to fine map the genetic architecture of Lp(a) in order to generate the best possible Lp(a) genetic risk score (GRS_Lp(a)_) that would enable us to clarify associations with the intricate aspects of lipoprotein metabolism. The gene score was generated by performing GWAS on circulating Lp(a) levels in FINRISK97 (*N*=4,935) using stepwise incremental conditioning (Supplementary Methods). The resulting gene score consisted of 18 independent genetic variants located near the *LPA* gene and associated with Lp(a) at genome-wide significance (*P*<5 × 10^−8^). All 18 SNPs were further replicated for circulating Lp(a) in The Cardiovascular Risk in Young Finns Study (YFS; *N*=2,022, Supplementary Table 6). The effect estimate weighted gene score explained 54% of Lp(a) variation in the discovery and 45% in the replication cohort. Notably, the effect estimates for the 18 variants were generally larger in the replication cohort, which might be reflective of the different assay methods used (Supplementary Table 5). We then assessed whether the metabolic associations were strengthened by the better instrument for genetically elevated Lp(a) by meta-analysing risk score associations with the metabolic measures in FINRISK97 and YFS (Fig. 3). The strongest association for the GRS_Lp(a)_ was again with the diameter of VLDL particles (*P*=8.6 × 10^−47^, N=7,365, Supplementary Data 1 for all associations in both individuals cohorts). We used Mendelian randomization^16^ to evaluate causality of Lp(a) for metabolic disturbances (Supplementary Methods and Fig. 3); the detailed lipoprotein measures and circulating Lp(a) levels were available in the FINRISK97 and YFS cohort for 4,889 and 1,991 individuals, respectively. The similar association pattern between observational associations and causal effect estimates strongly support that Lp(a) synthesis is causally altering lipoprotein concentrations (observational associations and instrumental variable estimates for the metabolites are presented in Fig. 3, and Supplementary Data 1). These findings suggest, maybe somewhat surprisingly, that Lp(a) synthesis widely affects overall lipoprotein metabolism, and in particular, the synthesis of large VLDL particles in the liver and thereby the triglyceride metabolism in general. Based on these results, we propose that the apoB-containing lipoprotein particle used to form Lp(a) by the covalent attachment of apo(a), may actually also be a poorly lipidated VLDL-type of particle. This suggests that circulating Lp(a) particles are likely to be a more heterogeneous group than simply an apo(a) component added to LDL particles^17^.

### *LPA* genetic risk score with electronic health records

Although several pharmaceutical agents are known to cause a modest decrease of circulating Lp(a), no drugs exist yet to effectively lower Lp(a)^18^. Statins do not lower the risk due to Lp(a) as statin use was not associated with a change in Lp(a) levels in a study by Cobbaert *et al*.^19^ and the JUPITER trial showed that Lp(a) was a significant predictor of residual risk in participants treated with potent statin therapy^20^. However, an antisense oligonucleotide targeting *LPA* mRNA was shown to effectively lower circulating Lp(a) in a phase 1 trial^21^ and is now in phase 2 trial (ClinicalTrials.gov Identifier: NCT02160899). The pharmacological use still depends on whether the *LPA* expression modifying treatment would be associated with unintended side effects. Since we were able to derive an exceptionally strong genetic instrument for the metabolic associations, we used reverse genetics to assess whether genetically elevated Lp(a) would be associated with any disease leading to hospitalization or death across the nation-wide electronic health-care registers in Finland in the FINRISK samples (*N*=17,487; 429,357 person-year follow-up; Supplementary Methods). We found that the gene score for Lp(a) was associated with ischaemic heart diseases (ICD10 I20-I25, *P*=6.8 × 10^−9^, *N*_events_=1,634, odds ratio (OR)=1.25 per unit increment in log(Lp(a))) but not with any of the other of the 218 summary diagnoses tested (Supplementary Data 2). Consistently, the gene score was only associated with diagnoses within this ICD-block when testing associations across all the 615 diagnoses in the electronic health-care records at the accuracy of three-digit ICD codes (Supplementary Data 2). Our study extends the results from a loss-of-function study by Lim *et al*.^22^ that used two truncating *LPA* splice variants serving as a human knock-out model for *LPA.* Their study found no increased morbidity for the individuals carrying the *LPA* knockout alleles. The present study had a considerably stronger genetic instrument for evaluating co-morbidities linked with Lp(a) in the general population and thus strengthens the evidence that no strong common disease co-morbidities are caused by Lp(a). However, these reverse genetic analyses prevent conclusions for rare disease events or weak association for common diseases. In addition, the Hospital Discharge Register Diagnoses are non-validated outcomes and this may reduce our power to detect associations. However, the general validity of the Finnish Hospital Discharge Register Diagnoses has been examined in numerous studies and found to be good^23^. Nevertheless, these novel findings support the notion that lowering circulating Lp(a) levels would be a suitable therapeutic target to reduce residual CHD risk, and that *LPA* targeting therapy could be a beneficial addition to statin treatment.

## Discussion

In this study, detailed molecular profiles of circulating metabolites were analysed for almost 25,000 individuals to increase knowledge on genetic regulation of systemic metabolism. Our main findings were twofold. First, a discovery of eight new genetic loci for circulating metabolites and fatty acids. The new associated loci contained either transporters or enzymes closely involved in the metabolism or trafficking of the associated metabolite as shown in Table 2. These new data are now available to be used to study the potential causality of a plethora of biomarkers and to better understand the intricate metabolic effects of known risk factors. Second, in our search for new metabolic pathways in relation to known disease-associated variants, we found that a known CHD-associated variant near *LPA* was linked with circulating triglycerides and VLDL metabolism. Because of these new metabolic findings for this particular variant, we focused on this region and fine mapped the genetic architecture of Lp(a). In fact, we were able to generate a gene score that explained over 45% of the variation in Lp(a) in the replication cohort. The metabolic associations were strengthened with the stronger genetic instrument. Subsequently, we used the genetic risk score in Mendelian randomization to show that the discovered novel effects of Lp(a) synthesis on overall lipoprotein and triglyceride metabolism are causal. Furthermore, as we now had a strong genetic risk score for Lp(a), we could use it for reverse genetics in combination with electronic health records. Intriguingly, according to extensive electronic health record data, the genetic variation in *LPA* appears to be associated with ischaemic heart disease but not with other common adverse disease events. Thus, our results provide the first evidence of the potential consequences to lipoprotein metabolism when people are treated with emerging drugs (a phase 2 trial for *LPA* mRNA antisense oligonucleotides is currently active (ClinicalTrials.gov Identifier: NCT02160899)). Our findings also provide support that the treatment may well be suitable for CHD risk reduction and is likely to be free of other strong morbidities. This study also serves as a proof of concept in terms of how large multiomics biobank data could be efficiently used to inform drug discovery at an early stage.

## Methods

### Metabolite quantification

This work is an extension of our previous GWA-metabolomics study, in which the quantitative high-throughput NMR metabolomics platform, used to quantify human blood metabolites, was applied^4^. In this study, we have utilized the same platform to quantify 123 metabolite measures that represent a broad molecular signature of systemic metabolism. The metabolite set covers multiple metabolic pathways, including lipoprotein lipids and subclasses, fatty acids as well as amino acids and glycolysis precursors. Most of the NMR-based metabolomics analyses were performed with the comprehensive quantitative serum/plasma platform described originally by Soininen *et al*.^24^ and reviewed recently^25^. This same platform was used here to analyse samples in Estonian Genome Center of University of Tartu Cohort (EGCUT), Finnish Twin Cohort, a subsample of FINRISK 1997 (FR97), Genetic Predisposition of Coronary Heart Disease in Patients Verified with Coronary Angiogram (COROGENE), Genetics of METabolic Syndrome, Helsinki Birth Cohort Study (HBCS), Cooperative Health Research in the Region of Augsburg (KORA), Northern Finland Birth Cohort 1966 (NFBC 1966), FINRISK subsample of incident cardiovascular cases and controls (PredictCVD), EGCUT sub-cohort (PROTE) and YFS. Metabolite-specific untransformed distributions and descriptive summary statistics from the largest cohort, NFBC 1966, are presented in Supplementary Fig. 3. Chemical shifts and the coefficients of variation for inter-assay variability are presented in Supplementary Data 3 for each metabolite. Here, the study was extended with Erasmus Rucphen Family Study (ERF), Leiden Longevity Study (LLS) and Netherlands Twin Register (NTR) cohorts for which the small-molecule information was available from another NMR-based method (Supplementary Table 2 for details)^26^. Metabolite-specific untransformed distributions and descriptive summary statistics for these measures from the ERF cohort are given in Supplementary Fig. 4. Chemical shifts and the coefficients of variation for inter-assay variability are presented in Supplementary Table 7. The sample material was mostly serum, except for EGCUT, PROTE, NTR and LLS in which the sample material was EDTA-plasma. The ERF cohort had additional lipoprotein measures available through the method developed by Bruker Ltd. (https://www.bruker.com/fileadmin/user_upload/8-PDF-Docs/MagneticResonance/NMR/brochures/lipo-analysis_apps.pdf). The terminology of this method utilized for lipoprotein analyses in ERF was matched based on the lipoprotein particle size with the comprehensive quantitative serum/plasma platform to enable meta-analyses. The vast majority of blood samples were fasting, however, if a study did not have overnight fasting samples, we corrected the fasting time effect by using R package gam and fitting a smoothed spline to adjust for fasting. All metabolites were first adjusted for age, sex, time from last meal, if applicable, and ten first principal components from genomic data and the resulting residuals were transformed to normal distribution by inverse rank-based normal transformation.

### Genome-wide association study

We performed a GWAS for metabolites from 14 cohorts from Europe, totaling up to 24,925 individuals (cohorts are described in Table 1, Supplementary Table 2 and Supplementary Notes 1) to include as many samples with NMR metabolite data and genome-wide SNP array data as possible. Written informed consent was obtained from all participants. Studies were approved by the following ethical committees: Ethical Committee of Oulu University Faculty of Medicine for NFBC 1966; Ethics Committee of the National Public Health Institute for Health2000 and HBCS; Helsinki University Hospital Coordinating Ethical Committee for FINRISK and Twins; The KORA studies have been approved by the ethics committee of the Bavarian Medical Association; NTR, Central Ethics Committee on Research Involving Human Subjects of the VU University Medical Center, Amsterdam; EGCUT, Ethics Review Committee on Human Research of the University of Tartu; ERF, medical ethics board of the Erasmus MC Rotterdam, the Netherlands; LLS, Medical Ethical Committee of the Leiden University Medical Centre; and Ethics Committee of the Hospital District of Southwest Finland for YFS. Individuals under lipid-lowering medication or pregnant were excluded form the analyses. FINRISK cohorts included genotype batches PredictCVD, COROGENE, DILGOM and FINRISK97. Estonian biobank had two genotype batches included in this study: EGCUT and PROTE. Genotype batches were analysed separately. We used an additive model implemented in analysis software (Supplementary Table 2) for each cohort. All studies were approved by local ethical committees. SNPs were imputed up to 39 million markers using a 1000 Genomes Project March 2012 version as described in Supplementary Table 2 (ref. 27). The genomic positions used throughout this study are human genome build 39. Each cohort was analysed separately and SNPs with accurate imputation (proper info>0.4) and minor allele count >3 were combined in fixed-effects meta-analysis using double genomic control correction, that is, both individual cohort results and meta-analysis results were corrected for the genomic inflation factor as implemented in GWAMA^28^. Variants, after filtering and meta-analysis, present in more than seven studies were considered for the final results. A genome-wide significance level was set to 2.27 × 10^−9^ correcting for 22 independent tests as the metabolite data are correlated (standard genome-wide significance threshold of 5 × 10^−8^/22, the number of principal components explaining over 95% of the variance in the metabolomics data). The number of independent tests was derived from the number of principal components that explain over 95% of variation in the metabolite data. All traits gave genomic inflation factors in the meta-analysis less than 1.034 showing that there was little evidence of systematic bias in the test statistics. Quantile plots for measurements listed in Supplementary Table 1 are presented in Supplementary Fig. 5.

### Conditional analyses and proportion of variance explained

We conducted an initial formal conditional analysis for each of the 62 significant loci. We performed an association test for all SNP—trait pairs in a 2- or 4-Mb window around the lead SNP. The 4-Mb window was used for seven loci where the association peak was so wide that it spanned over the 2-Mb window, as in the case of *CPS1* locus. The associations in each window were first screened in the seven Finnish cohorts only. The lead SNP-trait pair was then analysed using the meta-analysis summary statistics and correlation structure from the FINRISK-cohort to adjust for the correlation between the lead SNP and possible secondary variant using the method proposed by Yang *et al*.^29^. Further association was similarly adjusted for correlation between preceding variants. The proportion of variance explained was calculated based on the summary statistics for each trait accounting for all independent SNPs from the primary meta analysis and conditional analyses that were significant at the pre-specified threshold (*P*=2.27 × 10^−9^) for that trait.

### GTEx eQTL analyses

We investigated whether the lead SNPs of our associated loci were also associated with the expression levels of nearby genes by querying the multi-tissue gene expression resource from The GTEx project^9^,^30^. The project, data collection and analysis methods were recently described in detail^9^. Briefly, the pilot data set of the GTEx Project (dbGaP accession number phs000424.v3.p1) provides expression data for multiple tissues from up to 156 densely genotyped individuals per tissue. The eQTL analysis was focused on nine tissues having greater than 80 samples (Adipose—Subcutaneous, Artery—Tibial, Heart—Left Ventricle, Lung, Muscle—Skeletal, Nerve—Tibial, Skin—Sun Exposed Lower leg, Thyroid and Whole Blood) and genes expressed at least 0.1 reads per kilobase per million mapped reads (RPKM) in two or more individuals in a given tissue. For this paper, cis-eQTLs were calculated for those 57 independent SNPs from the association analysis that had minor allele frequency (MAF)>5% in the GTEx data using a cis window of 1 Mb up- and down-stream from the transcription start site of a gene. The analysis was conducted using the Matrix-eQTL R package^31^ in linear regression mode correcting for sex, the first 15 probabilistic estimation of expression residual factors, and the first three principal components from the genotype data. The false discovery rate was estimated across the tested SNP–gene pairs (between 1,933 and 2,269 per tissue) using the Benjamini–Hochberg procedure.

### Gene score for elevated Lp(a)

Lp(a) was measured in YFS from serum stored at –70 °C by the immunoturbidimetric method (Lp(a)-HA reagent, Wako Chemicals GmbH). Lp(a) was measured in FINRISK97 from serum stored at –70 °C using a commercially available latex immunoassay on an Architect c8000 system (Quantia Lp(a), Abbott Diagnostics). The imputed genotype batches PredictCVD, COROGENE and FR97 were combined to generate as complete a genotyped sample as possible for the genetic analyses (FINRISK97). We then performed a GWAS for natural logarithm transformed Lp(a) in FINRISK97 using sex, age and ten genetic principal components as covariates in linear models. Variants associated with Lp(a) at genome-wide significance were iteratively added to the association model for identification of independent variants. SNPs with info >0.7 and minor allele frequency >0.5% were considered. All 18 independent variants identified in FINRISK97 were replicated in the independent YFS cohort (Supplementary Data 1).

We used weighted effect estimates from FINRISK97 to generate a gene score for Lp(a) and tested the proportion of variance explained in the FINRISK97 discovery and YFS replication cohorts. We also tested the association between the Lp(a) gene score and metabolites using linear regression adjusted for the same covariates as for the GWAS.

### Causality estimates for Lp(a) on lipoprotein metabolism

We used natural logarithm to transform the Lp(a) distribution and performed linear regression to test for association between Lp(a) and metabolites using linear regression adjusted for the same covariates as for the GWAS. As the effect estimates in FINRISK97 for circulating Lp(a) were larger than in YFS, we tested if differences in fasting time could account for the deviations—participants in FINRISK97 were only instructed to fast 4 h before the blood samples in contrast to overnight fasting in YFS. We observed no differences in the effect estimates between fasting over 8 h (*N*=4,269) or fasting less than 8 h (*N*=620) subgroups (*β*=0.40 for both groups) in FINRISK97.

Causal estimates of Lp(a) on metabolite measures were assessed by two-stage least-squares regression with the Lp(a) gene score as instrument. To enable comparison between the observational and causal effect estimates from these Mendelian randomization analyses, Lp(a) and metabolites had been corrected and transformed as in the GWAS. Observational associations, genetic risk score associations and instrumental variable estimates from FINRISK97 and YFS were combined with inverse variance weighted meta-analysis.

### Reverse genetics for LPA with nationwide electron health records

The gene score for Lp(a) was tested in the FINRISK field studies conducted in 1992, 1997, 2002 and 2007 for association with any disease event leading to hospitalization or death in Finland during January 1987 to December 2010. PredictCVD, COROGENE, FR97 and additional Illumina core-exome genotyped sample of 9,906 FINRISK individuals were combined after imputation to form as complete and an unrelated data set as possible from FINRISK-samples. Genetic principal components were generated from the combined genotyped SNPs to account for population stratification and also to exclude related individuals. Maximum relatedness between individuals was set to 0.1 between genotyping batches to remove related individuals from the combined sample resulting in 17,496 unrelated individuals. Disease tracking was enabled by uniform diagnosis data obtained from the Finnish National Hospital Discharge Register and the National Causes-of-Death Register. These registers cover all events that have led to either overnight hospitalization or death in Finland. The disease events are linked to study participants using their social security number, which is assigned to every permanent resident of Finland. Both direct and side causes of the diagnoses and cause of death were analysed. Hospitalization and fatal events were combined.

The disease diagnoses were encoded according to the International Classification of Diseases 10th Revision (ICD-10) from 1997 onwards. Disease events occurring from 1987 to 1996 were encoded in ICD-9 format across Finland; these diagnoses were converted to ICD-10 format by the scheme provided by the United States Center for Disease Control Diagnosis Code Set General Equivalence Mappings (ftp://ftp.cdc.gov/pub/Health_Statistics/NCHS/Publications/ICD10CM/2011/), including combination codes. All diagnosis conversions were further verified according to the mapping scheme provided by the New Zealand Ministry of Health, National Data Policy Group (http://www.health.govt.nz/system/files/documents/pages/masterf4.xls). Manual curation of the conversion was conducted for diagnoses with mismatch in the conversion to the degree of three digits.

Testing of the Lp(a) gene score against disease events was conducted by logistic regression, using the gene score as a predictor and ever-occurrence of a disease from 1987 onwards as a binary outcome, with adjustment for sex, age at end-of-follow-up, study-collection-year and the first four principal components accounting for population structure. The cohorts were analysed here jointly. Similar results were obtained when the cohorts were also analysed separately and meta-analysed (data not shown). Diagnoses with more than ten events were analysed. The disease events tested were 18 ICD-10 chapters (for example, Diseases of the circulatory system: I00-I99), 189 ICD-10 blocks (A00-09 to T90-98, for example, Ischaemic heart diseases: I20-I25) and 615 ICD-10 codes (from A00 to T98, for example, Angina pectoris: I20). Overall, 822 outcomes were tested. We therefore corrected for multiple testing of 822 tests. The total follow-up time amounted to 429,357 person-years. Disease-specific follow-up for time to event models are given in the Supplementary Data 2.

## Additional information

**How to cite this article:** Kettunen, J. *et al*. Genome-wide study for circulating metabolites identifies 62 loci and reveals novel systemic effects of *LPA*. *Nat. Commun.* 7:11122 doi: 10.1038/ncomms11122 (2016).

## Supplementary Material

Supplementary Information Supplementary Figures 1-5, Supplementary Tables 1-7, Supplementary Notes 1-2 and Supplementary References

Supplementary Data 1 Mendelian randomization analyses of Lp(a), first are the three estimates listed for FINRISK97, and below same estimates for YFS

Supplementary Data 2 Reverse genetics analyses for ICD categories, below this table are presented the three digit accuracy codes.

Supplementary Data 3 For the NMR method (described in Soininen et al, Analyst, 2009) the most representative chemical shift values for the metabolic measures in the NMR spectra are shown (many metabolites have a complicated signal with components at multiple chemical shift locations).The representative coefficients of variation (in percent) are calculated for each metabolic measure based on 100 quality control serum samples in relation to the population mean of each metabolic measure. The control samples are processed identically with the actual study samples; one sample being placed in every 96-sample box, i.e., in this case the percentages represent an averaged situation for 9600 samples analysed.

## Figures and Tables

**Table 1:** Sample demographics.

**Study**		**Age**		**BMI**		**Female%**
	***N***	**Mean**	**s.d.**	**Mean**	**s.d.**	
EGCUT	3,287	46.3	19.5	26.4	5.4	58
ERF	2,118	48.2	14.7	26.7	4.7	58
FTC	664	23.9	2.1	23.1	3.7	50
FR97	3,661	45.3	12.8	26.3	4.5	55
COROGENE	828	53.2	13.2	26.6	4.1	54
GenMets	572	55.8	7.3	27.2	4.5	57
HBCS	708	61.3	2.9	27.1	4.1	60
KORA	1,745	60.9	8.8	28.2	4.8	52
LLS	2,227	59.2	6.8	25.4	3.5	54
NTR	1,192	38.8	12.8	24.6	4.2	64
NFBC 1966	4,709	31.2	0.4	24.6	4.1	51
PredictCVD	374	47.5	14.6	26.6	4.4	37
PROTE	597	38.3	16	25.2	4.6	51
YFS	2,390	37.7	5.0	26	4.7	54

BMI, body mass index; COROGENE, Genetic Predisposition of Coronary Heart Disease in Patients Verified with Coronary Angiogram; EGCUT, Estonian Genome Center of University of Tartu Cohort; ERF, Erasmus Rucphen Family Study; FR97, a subsample of FINRISK 1997; FTC, Finnish Twin Cohort; GenMets, Genetics of METabolic Syndrome; HBCS, Helsinki Birth Cohort Study; KORA, Cooperative Health Research in the Region of Augsburg; LLS, Leiden Longevity Study; *N*, number of individuals with both genotype and metabolite traits analysed; NFBC 1966, Northern Finland Birth Cohort 1966; NTR, Netherlands Twin Register; PredictCVD, FINRISK subsample of incident cardiovascular cases and controls; PROTE, EGCUT sub-cohort; YFS, The Cardiovascular Risk in Young Finns Study.

**Table 2:** Novel significant loci identified in the GWAS.

**Trait**	**Variantidentifier**	**Chr**	**Position**	**ea/nea**	**Eaf**	**Beta**	**s.e.**	***P*** **-value**	**Q** ***P*** **-value**	***N*** **samples**	**Candidategene**	**eQTL**	**Function**
Glycine	chr3:125905336:D	3	125905336	A/ACCTGACCCTGAC	0.40	0.07	0.01	1.1 × 10^−9^	0.03	17,541	*SLC41A3*	—	TFBS
Glycine	rs140348140	9	5877295	TA/T	0.05	0.33	0.03	3.7 × 10^−40^	4.7 × 10^−6^	17,535	*GLDC*	—	—
otPUFA	rs186183604	11	67128733	A/G	0.04	−0.24	0.04	3.2 × 10^−11^	0.71	13,545	*CLCF1*	—	Intron;*LOC100130987*
Alanine	rs4554975	12	47201814	G/A	0.64	−0.07	0.01	6.1 × 10^−13^	0.76	24,792	*SLC38A4*	—	Intron
Histidine	rs7954638	12	96314795	A/C	0.48	−0.08	0.01	7.3 × 10^−15^	0.53	19,240	*HAL*	*AMDH1*	Intron;CCDC38
Histidine	rs1998848	14	21492229	A/G	0.05	0.15	0.02	4.9 × 10^−10^	0.06	19,239	*NDRG2*	—	TFBS/5'UTR
Pyruvate	rs74249229	16	69979271	T/C	0.05	−0.15	0.02	2.1 × 10^−11^	0.17	23,561	*PDPR*	—	—
Glycine	rs10083777	16	81065282	T/C	0.17	−0.11	0.01	3.0 × 10^−13^	0.92	18,732	*GCSH*	*GCSH,ATMIN*,*LOC102724325*	TFBS

Beta, effect estimate; ea, effect allele; Eaf, effect allele frequency; eQTL, expression quantitative trait locus from GTEx; GWAS, genome-wide association study; nea, non-effect allele; otPUFA, polyunsaturated fatty acids (other than 18:2); Q, heterogeneity statistics; TFBS, transcription factor-binding site.

If the SNP is located in an intron of a different gene than the candidate, then the gene is presented in the Function column after semicolon.

Beta refers to one copy addition of the effect allele in s.d. units.
