# Supplementary material for: Genome-wide study for circulating metabolites identifies 62 loci and reveals novel systemic effects of LPA
Source: Nat Commun. 2016 Mar 23;7:11122. doi: 10.1038/ncomms11122 (PMC4814583; doi:10.1038/ncomms11122)
Supplement: Supplementary Information — Supplementary Figures 1-5, Supplementary Tables 1-7, Supplementary Notes 1-2 and Supplementary References [file ncomms11122-s1.pdf]

Supplementary Figure 1. Regional Manhattan plots for all 62 lead loci. Annotation key for the symbols is presented below. Correlation structure with the lead variant is presented as sliding color and key is present in all figures. Correlation structure could not be calculated for insertions/deletions and therefore those figures contain only grey symbols.

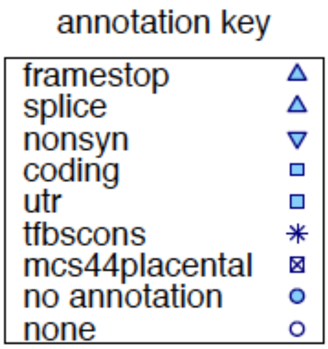

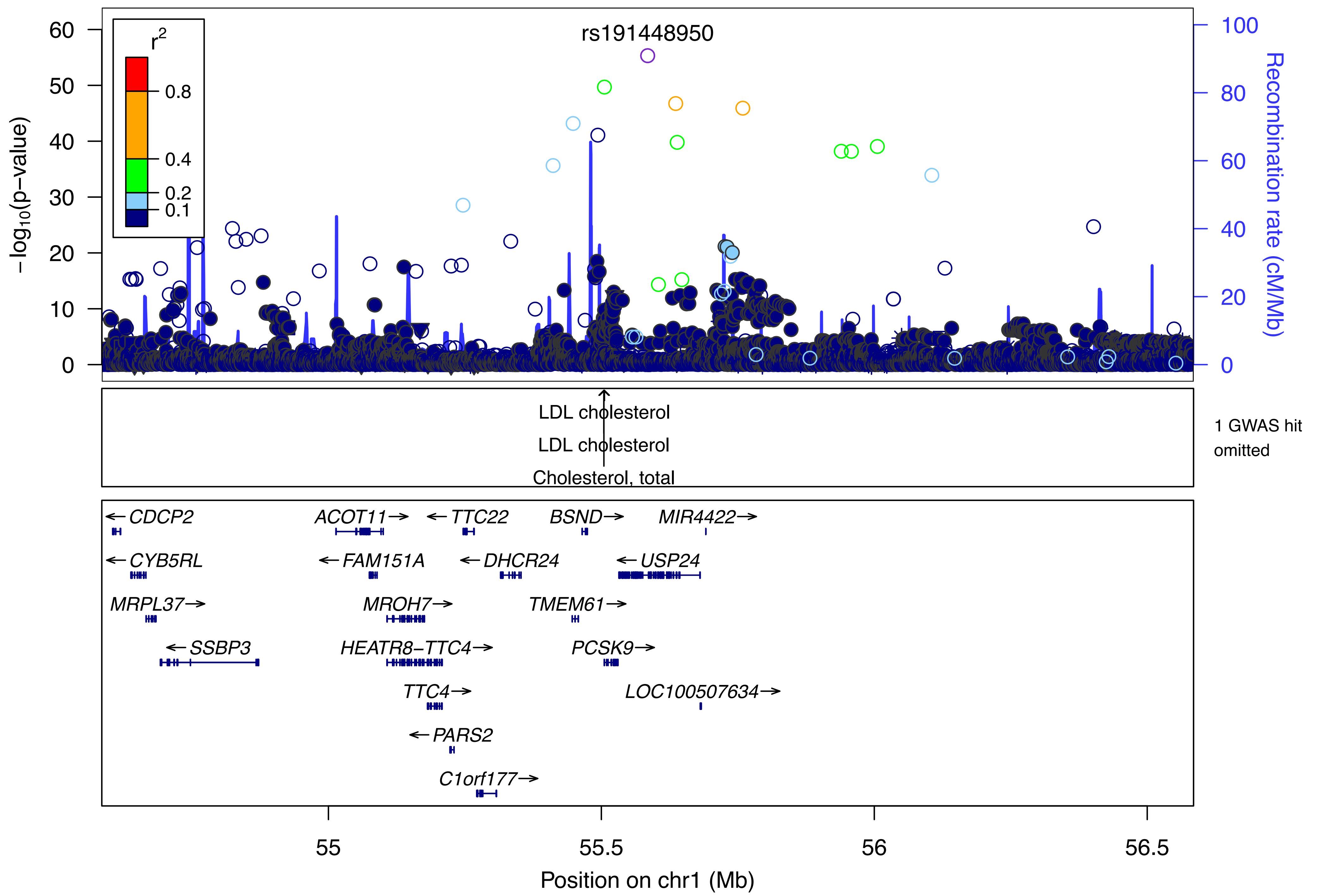
rs191448950
r2
0.8
0.4
0.2
0.1
−log10(p−value)
0
10
20
30
40
50
60
Recombination rate (cM/Mb)
0
20
40
60
80
100
LDL cholesterol
LDL cholesterol
Cholesterol, total
1 GWAS hit omitted
←CDCP2
←CYB5RL
MRPL37→
←SSBP3
ACOT11→
←FAM151A
MROH7→
HEATR8−TTC4→
TTC4→
←PARS2
C1orf177→
←TTC22
←DHCR24
BSND→
TMEM61→
PCSK9→
MIR4422→
←USP24
LOC100507634→
55
55.5
56
56.5
Position on chr1 (Mb)

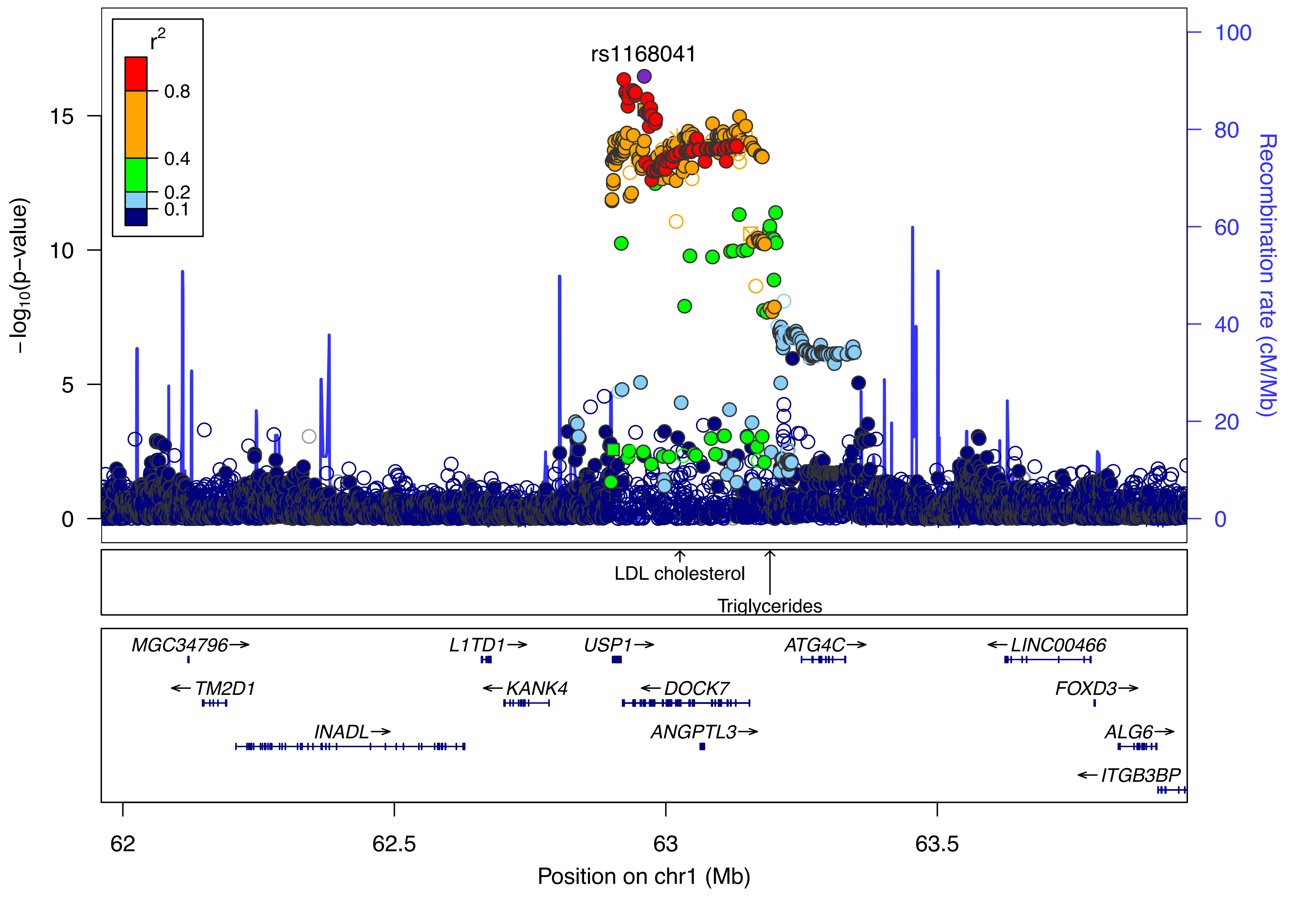

r2
0.8
0.4
0.2
0.1
rs1168041
15
10
5
0
−log10(p−value)
100
80
60
40
20
0
Recombination rate (cM/Mb)
LDL cholesterol
Triglycerides
MGC34796→
←TM2D1
INADL→
L1TD1→
←KANK4
USP1→
←DOCK7
ANGPTL3→
ATG4C→
←LINC00466
FOXD3→
ALG6→
←ITGB3BP
62
62.5
63
63.5
Position on chr1 (Mb)

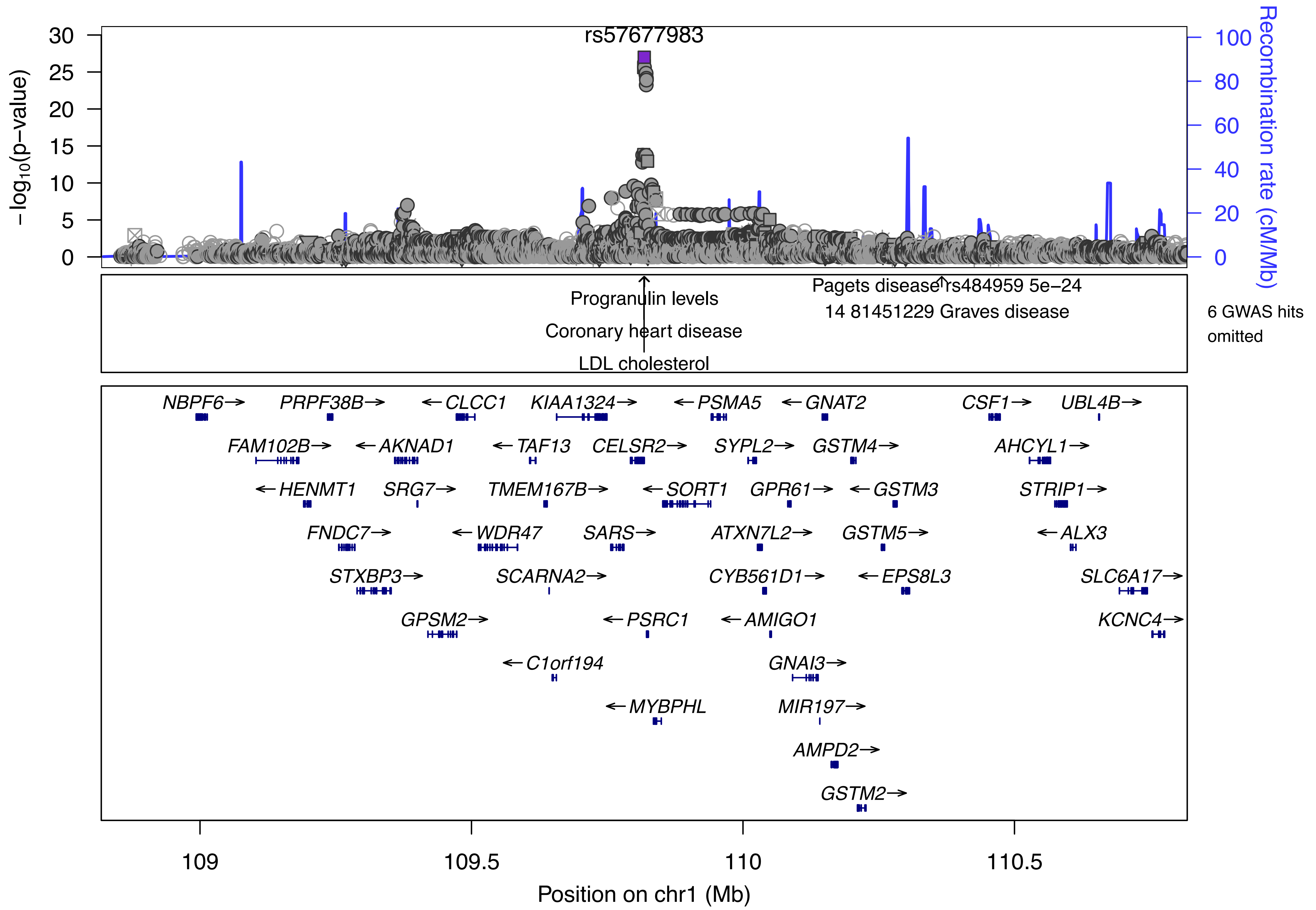
rs57677983
−log10(p−value)
Recombination rate (cM/Mb)
Progranulin levels
Coronary heart disease
LDL cholesterol
Pagets disease rs484959 5e−24
14 81451229 Graves disease
6 GWAS hits omitted
NBPF6→ PRPF38B→ ←CLCC1 KIAA1324→ ←PSMA5 ←GNAT2 CSF1→ UBL4B→
FAM102B→ ←AKNAD1 ←TAF13 CELSR2→ SYPL2→ GSTM4→ AHCYL1→
←HENMT1 SRG7→ TMEM167B→ ←SORT1 GPR61→ ←GSTM3 STRIP1→
FNDC7→ ←WDR47 SARS→ ATXN7L2→ GSTM5→ ←ALX3
STXBP3→ SCARNA2→ CYB561D1→ ←EPS8L3 SLC6A17→
GPSM2→ ←PSRC1 ←AMIGO1 KCNC4→
←C1orf194 GNAI3→
←MYBPHL MIR197→
AMPD2→
GSTM2→
109 109.5 110 110.5
Position on chr1 (Mb)

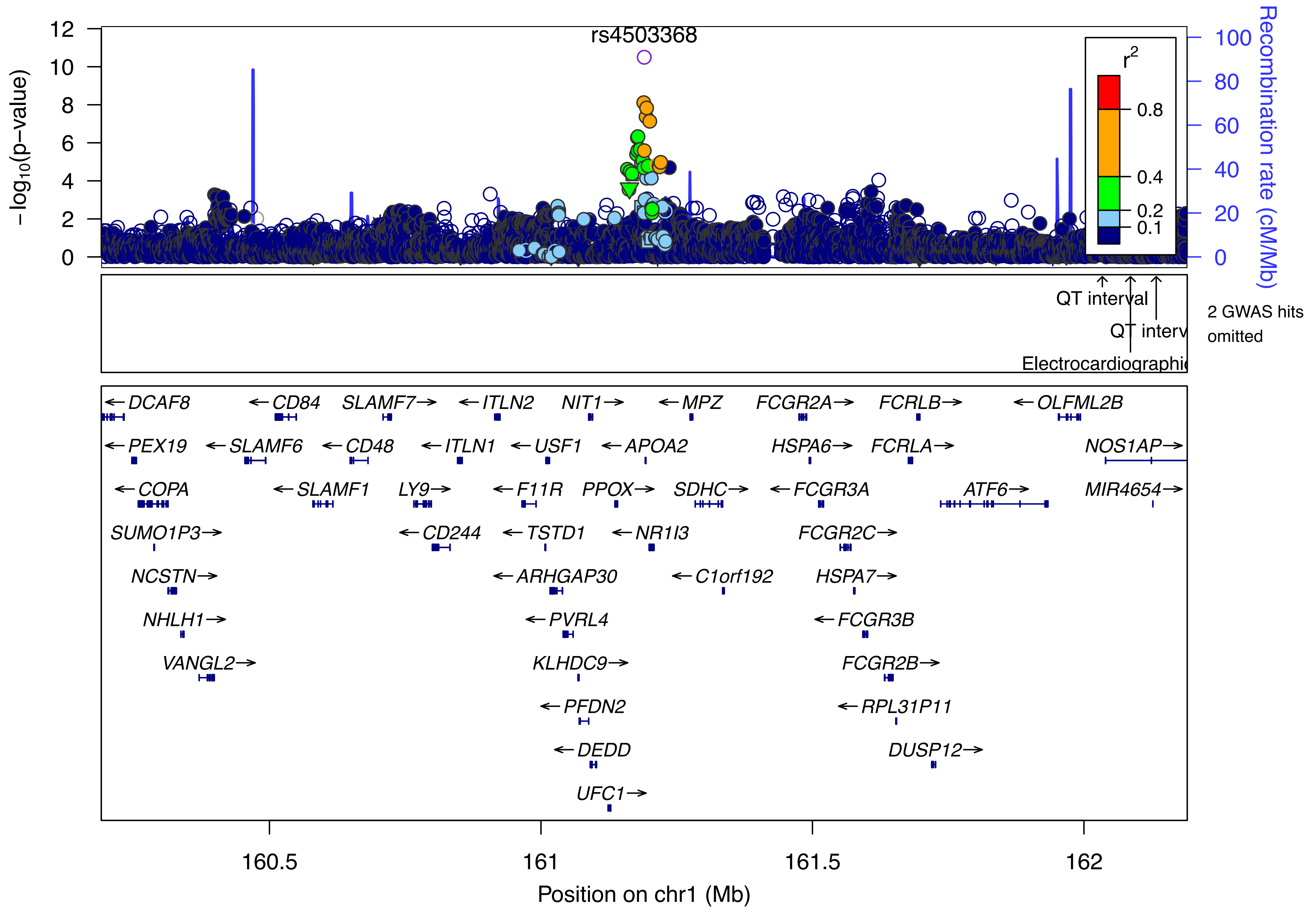

rs4503368
−log10(p−value)
Recombination rate (cM/Mb)
r2
0.8
0.4
0.2
0.1
QT interval
QT interv
Electrocardiographic
2 GWAS hits omitted
←DCAF8
←CD84
SLAMF7→
←ITLN2
NIT1→
←MPZ
FCGR2A→
FCRLB→
←OLFML2B
←PEX19
←SLAMF6
←CD48
←ITLN1
←USF1
←APOA2
HSPA6→
FCRLA→
NOS1AP→
←COPA
←SLAMF1
LY9→
←F11R
PPOX→
SDHC→
←FCGR3A
ATF6→
MIR4654→
SUMO1P3→
←CD244
←TSTD1
←NR1I3
FCGR2C→
NCSTN→
←ARHGAP30
←C1orf192
HSPA7→
NHLH1→
←PVRL4
←FCGR3B
VANGL2→
KLHDC9→
FCGR2B→
←PFDN2
←RPL31P11
←DEDD
DUSP12→
UFC1→
160.5
161
161.5
162
Position on chr1 (Mb)

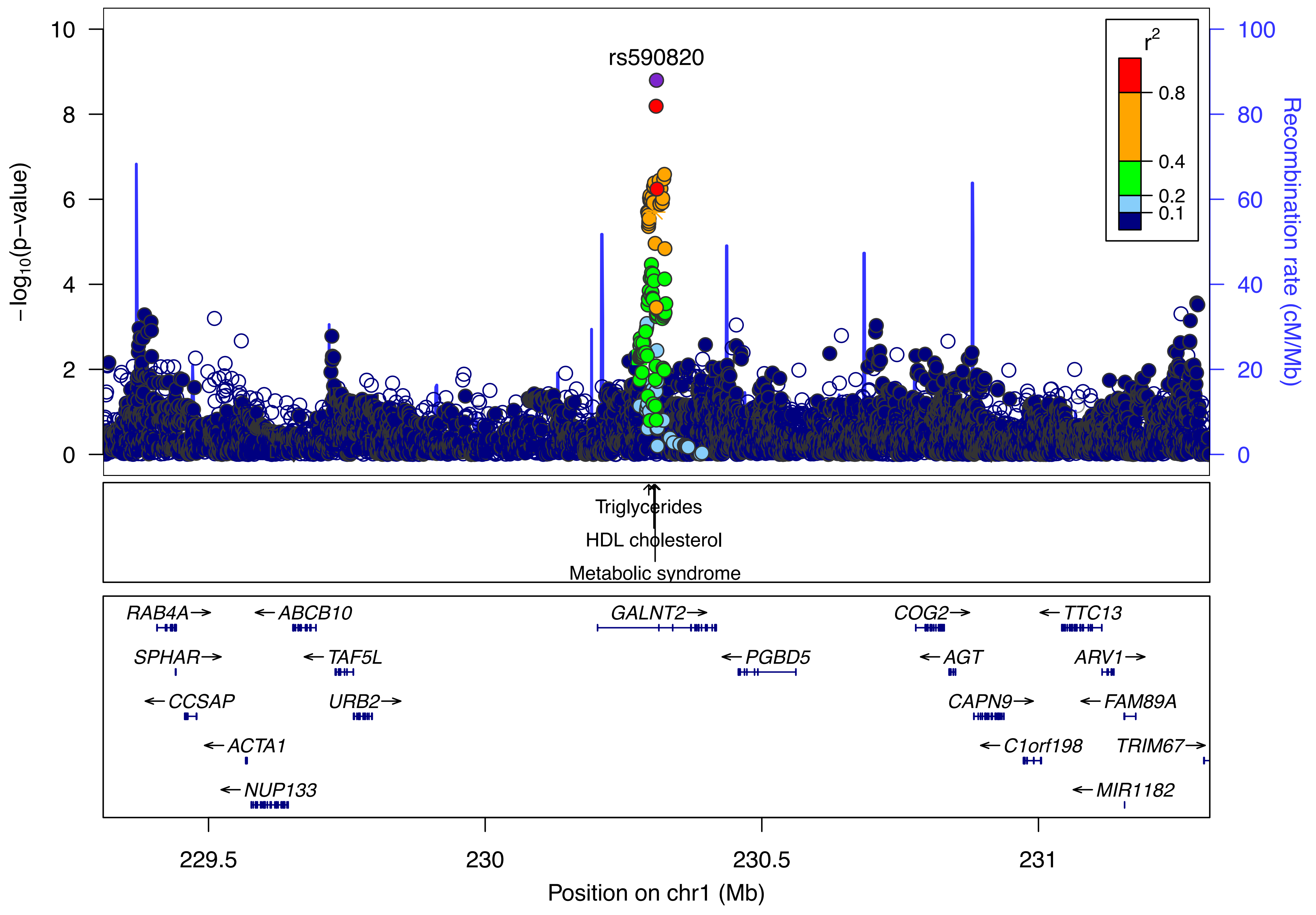

rs590820
−log10(p−value)
Recombination rate (cM/Mb)
r2
0.8
0.4
0.2
0.1
Triglycerides
HDL cholesterol
Metabolic syndrome
RAB4A→
←ABCB10
GALNT2→
COG2→
←TTC13
SPHAR→
←TAF5L
←PGBD5
←AGT
ARV1→
←CCSAP
URB2→
CAPN9→
←FAM89A
←ACTA1
←C1orf198
TRIM67→
←NUP133
←MIR1182
229.5
230
230.5
231
Position on chr1 (Mb)

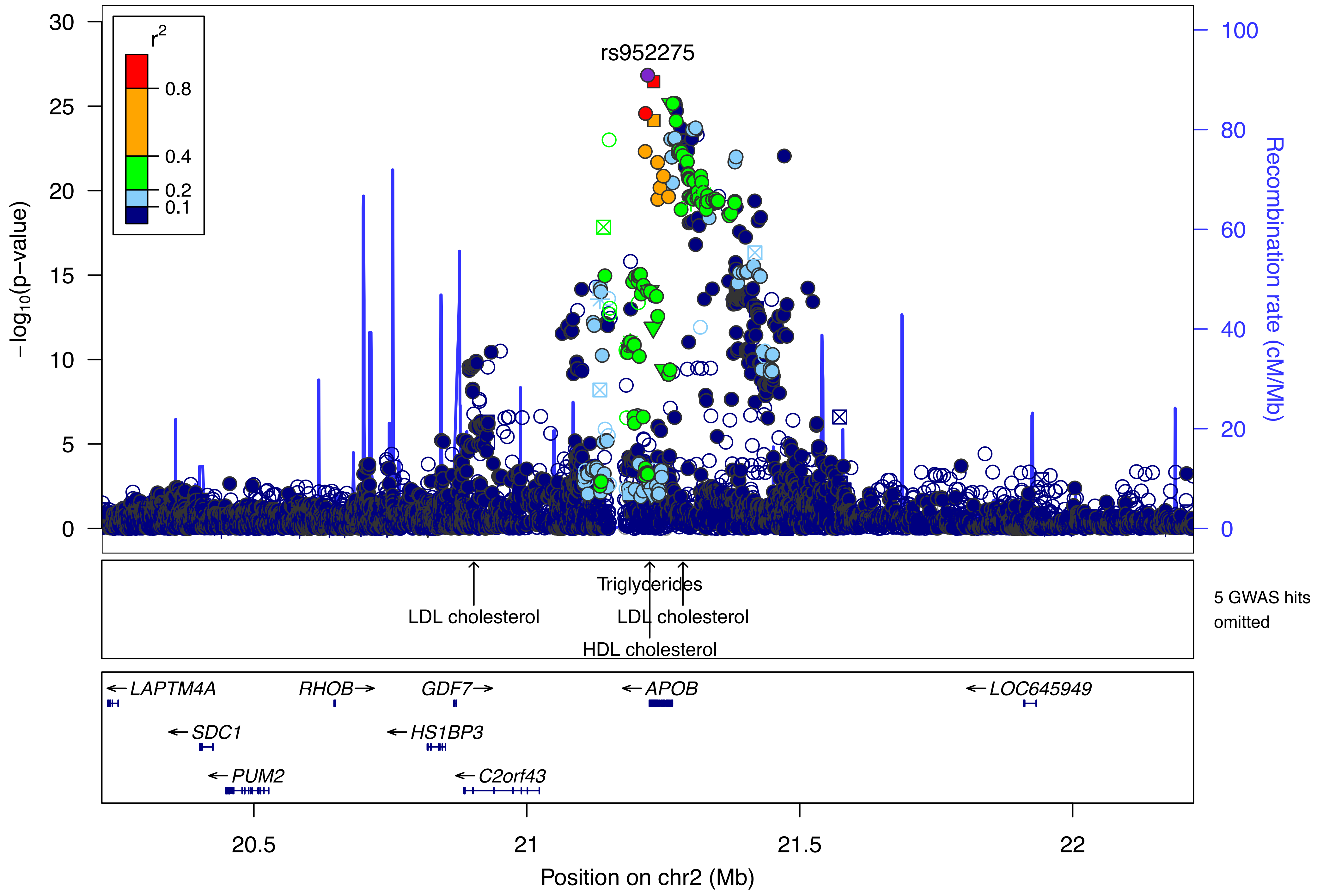
rs952275
r2
0.8
0.4
0.2
0.1
−log10(p−value)
Recombination rate (cM/Mb)
LDL cholesterol
Triglycerides
LDL cholesterol
HDL cholesterol
5 GWAS hits omitted
LAPTM4A
SDC1
PUM2
RHOB
GDF7
HS1BP3
C2orf43
APOB
LOC645949
Position on chr2 (Mb)

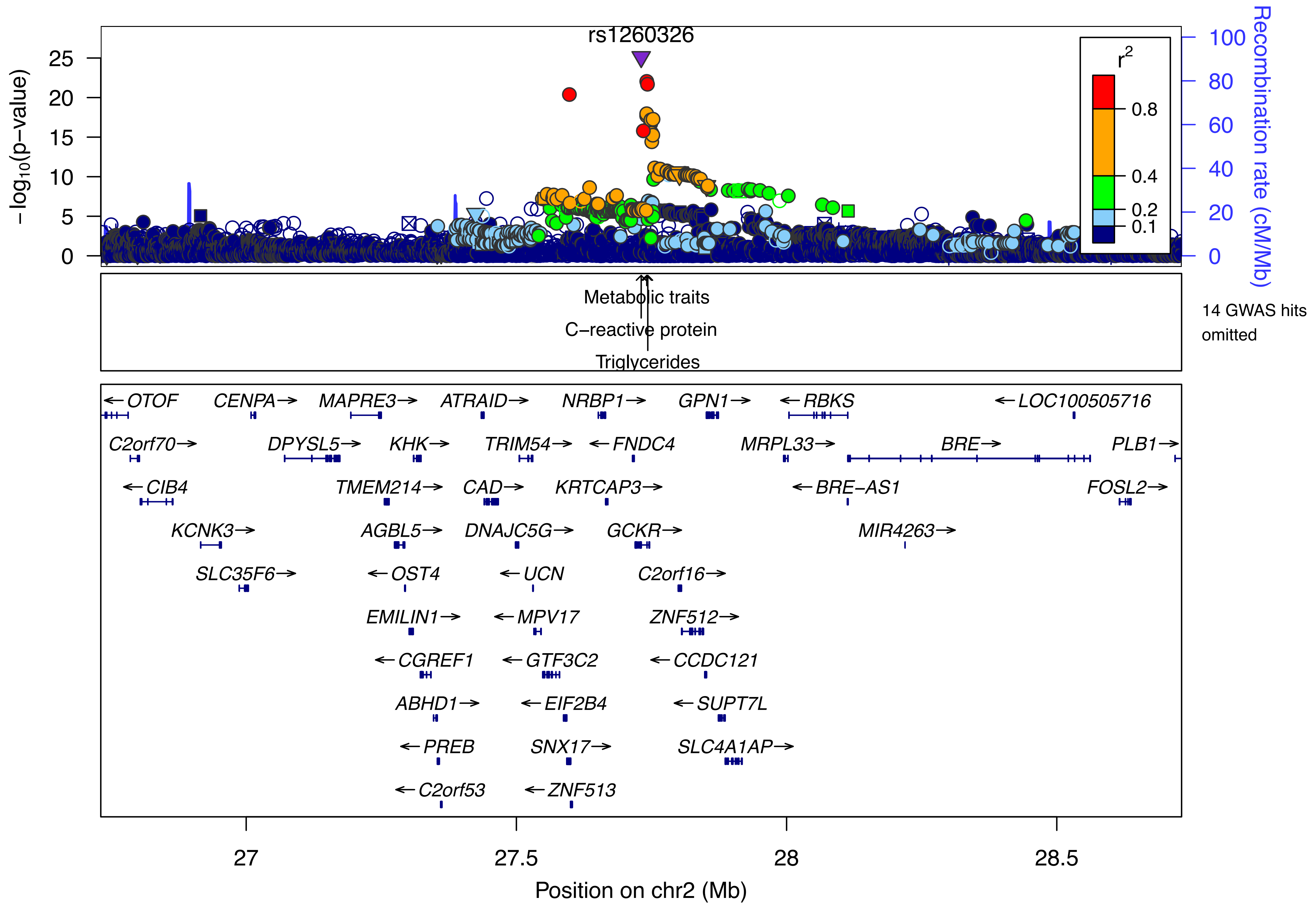
rs1260326
−log10(p−value)
Recombination rate (cM/Mb)
r2
0.8
0.4
0.2
0.1
Metabolic traits
C−reactive protein
Triglycerides
14 GWAS hits omitted
←OTOF
CENPA→
MAPRE3→
ATRAID→
NRBP1→
GPN1→
←RBKS
←LOC100505716
C2orf70→
DPYSL5→
KHK→
TRIM54→
←FNDC4
MRPL33→
BRE→
PLB1→
←CIB4
TMEM214→
CAD→
KRTCAP3→
←BRE-AS1
FOSL2→
KCNK3→
AGBL5→
DNAJC5G→
GCKR→
MIR4263→
SLC35F6→
←OST4
←UCN
C2orf16→
EMILIN1→
←MPV17
ZNF512→
←CGREF1
←GTF3C2
←CCDC121
ABHD1→
←EIF2B4
←SUPT7L
←PREB
SNX17→
SLC4A1AP→
←C2orf53
←ZNF513
27
27.5
28
28.5
Position on chr2 (Mb)

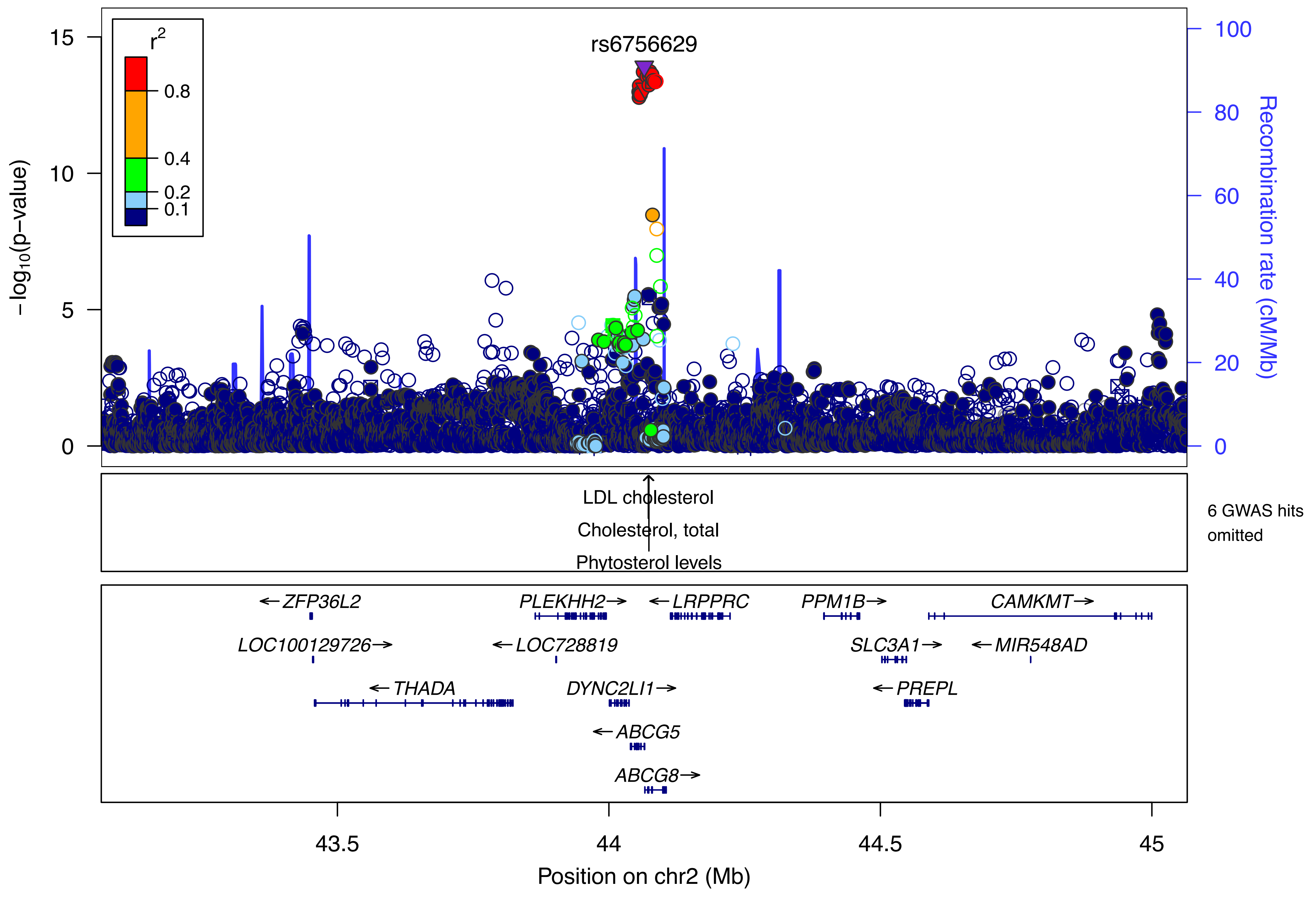
rs6756629
r2
0.8
0.4
0.2
0.1
−log10(p−value)
0
5
10
15
Recombination rate (cM/Mb)
0
20
40
60
80
100
LDL cholesterol
Cholesterol, total
Phytosterol levels
6 GWAS hits
omitted
←ZFP36L2
LOC100129726→
←THADA
PLEKHH2→
←LOC728819
DYNC2LI1→
←ABCG5
ABCG8→
←LRPPRC
PPM1B→
SLC3A1→
←PREPL
CAMKMT→
←MIR548AD
43.5
44
44.5
45
Position on chr2 (Mb)

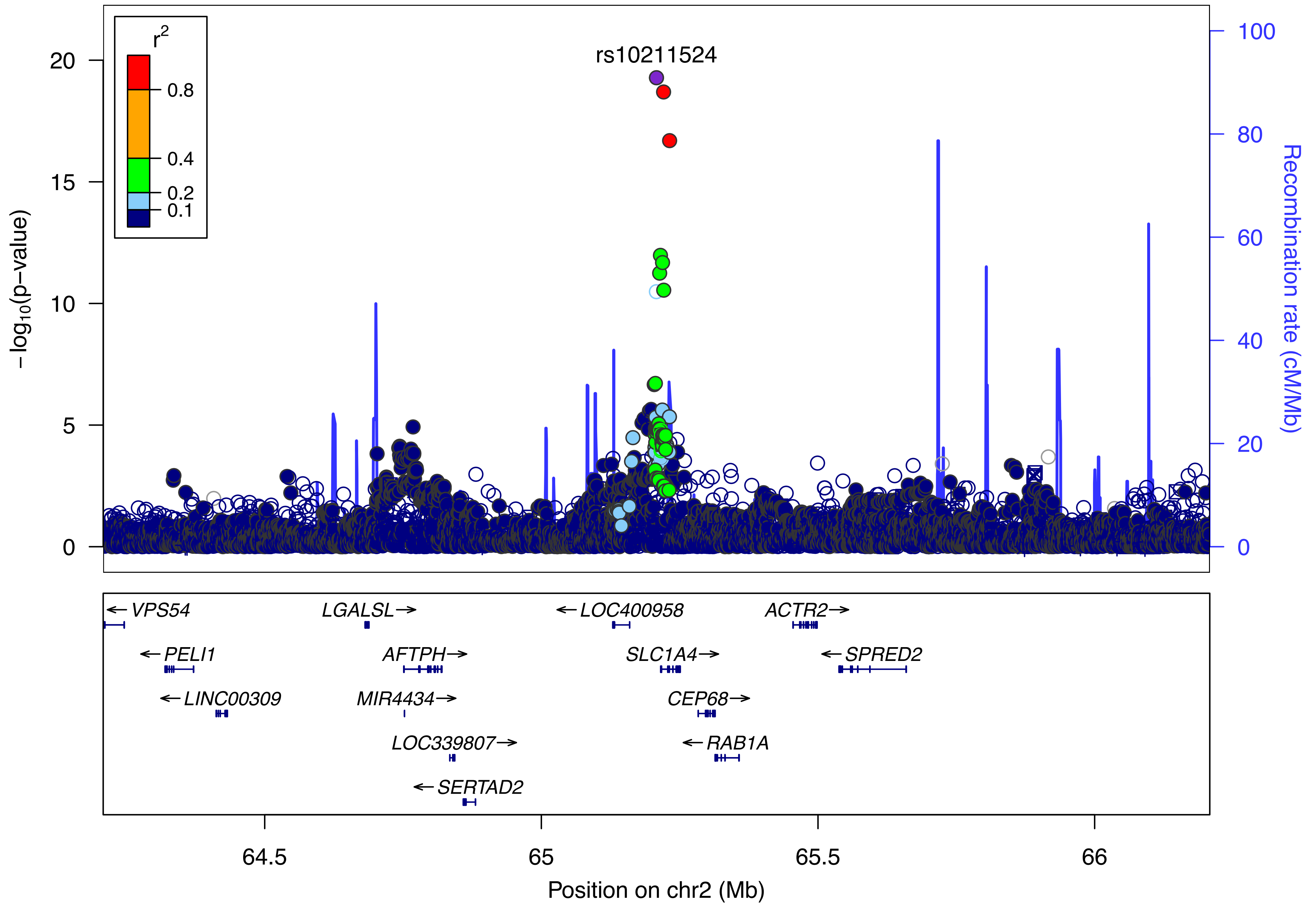

r2
0.8
0.4
0.2
0.1
rs10211524
−log10(p−value)
Recombination rate (cM/Mb)
VPS54
PELI1
LINC00309
LGALSL
AFTPH
MIR4434
LOC339807
SERTAD2
LOC400958
SLC1A4
CEP68
RAB1A
ACTR2
SPRED2
64.5
65
65.5
66
Position on chr2 (Mb)

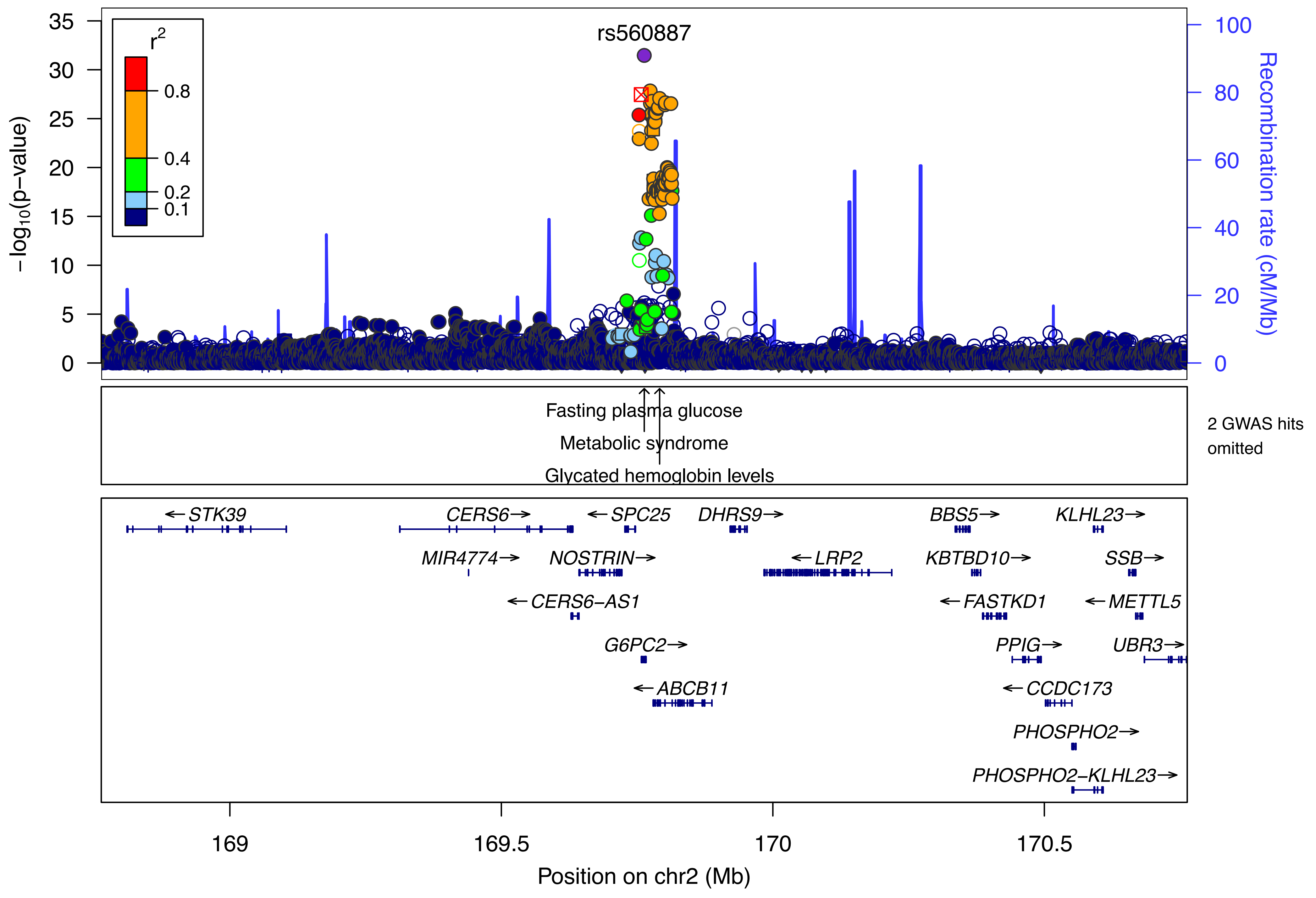
rs560887
r2
0.8
0.4
0.2
0.1
−log10(p−value)
Recombination rate (cM/Mb)
Fasting plasma glucose
Metabolic syndrome
Glycated hemoglobin levels
2 GWAS hits omitted
←STK39
CERS6→
MIR4774→
←SPC25
NOSTRIN→
←CERS6−AS1
G6PC2→
←ABCB11
DHRS9→
←LRP2
BBS5→
KBTBD10→
←FASTKD1
PPIG→
←CCDC173
PHOSPHO2→
PHOSPHO2−KLHL23→
KLHL23→
SSB→
←METTL5
UBR3→
169
169.5
170
170.5
Position on chr2 (Mb)

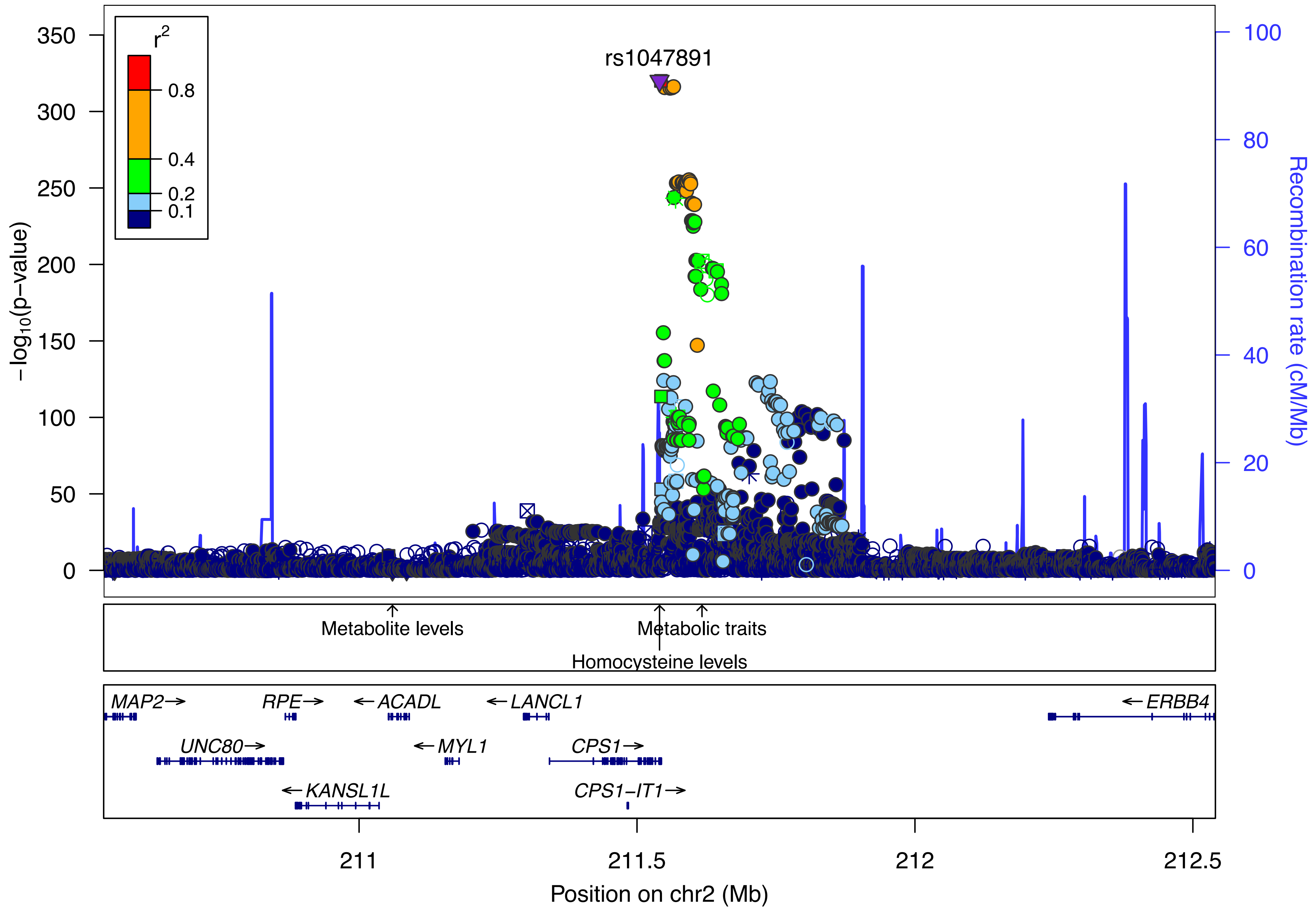
rs1047891
r2
0.8
0.4
0.2
0.1
−log10(p−value)
Recombination rate (cM/Mb)
Metabolite levels
Metabolic traits
Homocysteine levels
MAP2→
RPE→
←ACADL
←LANCL1
←ERBB4
UNC80→
←MYL1
CPS1→
←KANSL1L
CPS1−IT1→
211
211.5
212
212.5
Position on chr2 (Mb)

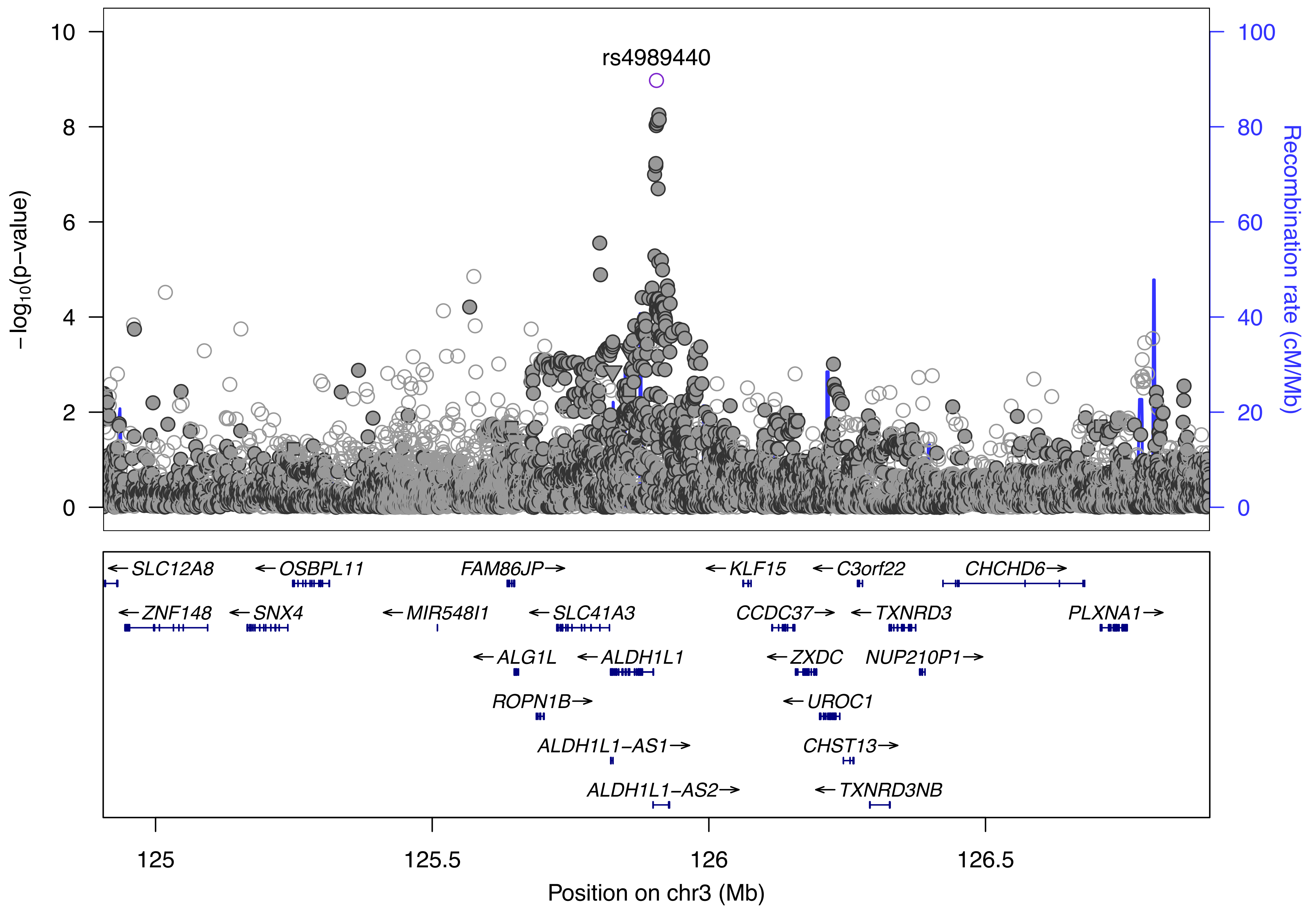
rs4989440
−log10(p−value)
Recombination rate (cM/Mb)
SLC12A8
OSBPL11
FAM86JP
KLF15
C3orf22
CHCHD6
ZNF148
SNX4
MIR548I1
SLC41A3
CCDC37
TXNRD3
PLXNA1
ALG1L
ALDH1L1
ZXDC
NUP210P1
ROPN1B
UROC1
ALDH1L1−AS1
CHST13
ALDH1L1−AS2
TXNRD3NB
125
125.5
126
126.5
Position on chr3 (Mb)

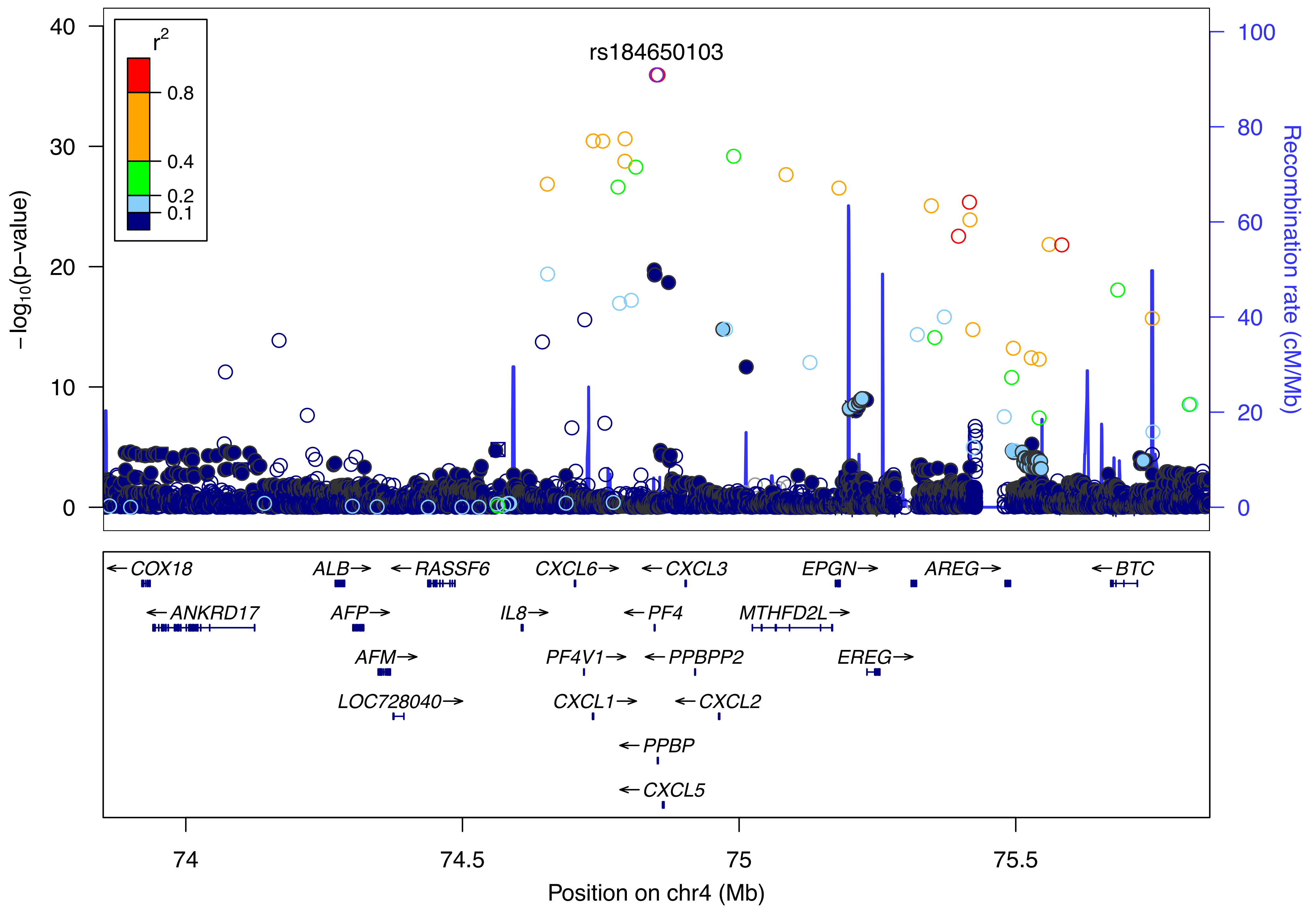

rs184650103
r2
0.8
0.4
0.2
0.1
−log10(p−value)
0
10
20
30
40
Recombination rate (cM/Mb)
0
20
40
60
80
100
←COX18
←ANKRD17
ALB→
AFP→
AFM→
LOC728040→
←RASSF6
IL8→
CXCL6→
PF4V1→
CXCL1→
←CXCL3
←PF4
←PPBPP2
←CXCL2
←PPBP
←CXCL5
MTHFD2L→
EPGN→
EREG→
AREG→
←BTC
74
74.5
75
75.5
Position on chr4 (Mb)

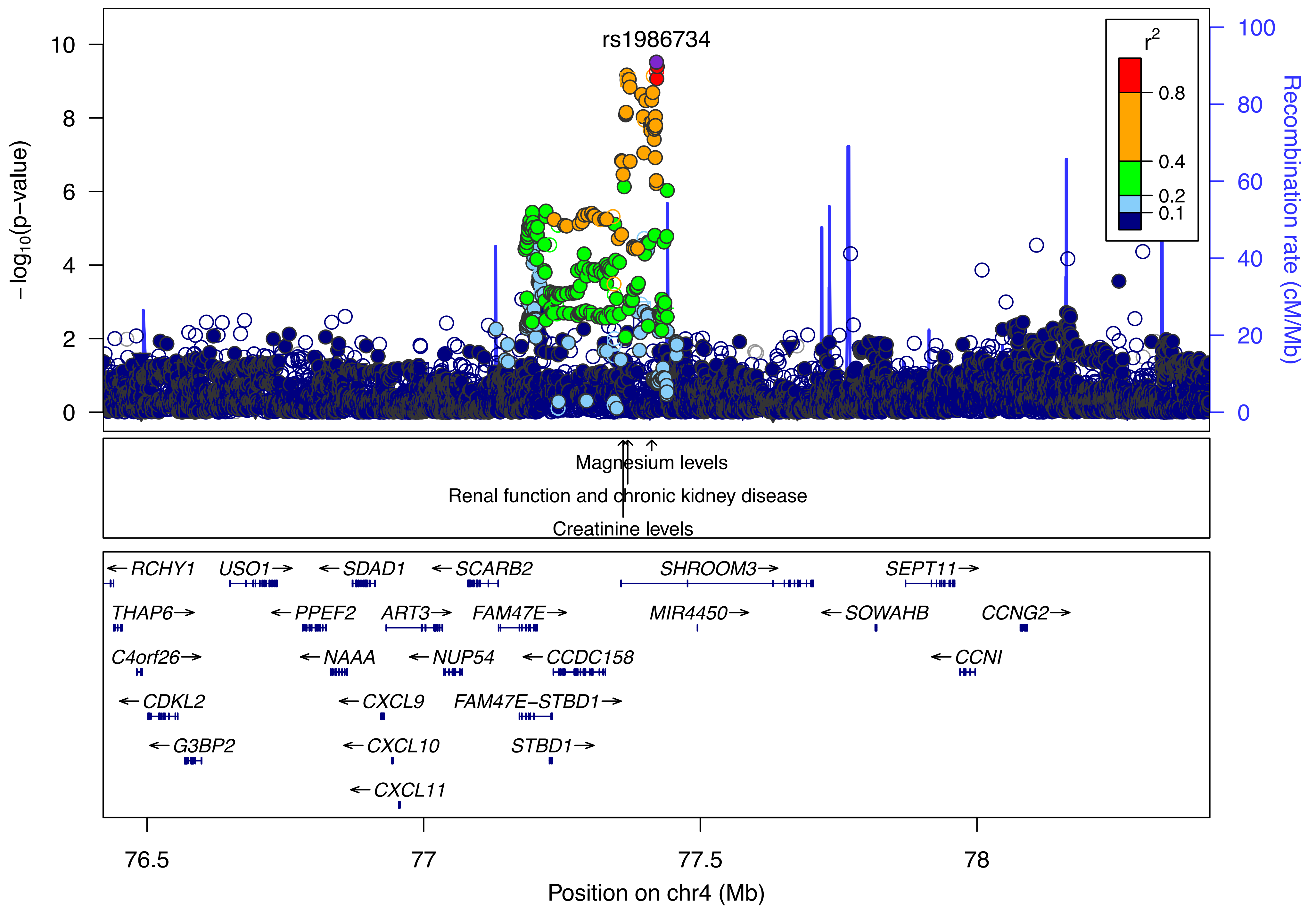
rs1986734
-log10(p-value)
Recombination rate (cM/Mb)
r2
0.8
0.4
0.2
0.1
Magnesium levels
Renal function and chronic kidney disease
Creatinine levels
RCHY1
USO1
SDAD1
SCARB2
SHROOM3
SEPT11
THAP6
PPEF2
ART3
FAM47E
MIR4450
SOWAHB
CCNG2
C4orf26
NAAA
NUP54
CCDC158
CCNI
CDKL2
CXCL9
FAM47E-STBD1
G3BP2
CXCL10
STBD1
CXCL11
76.5
77
77.5
78
Position on chr4 (Mb)

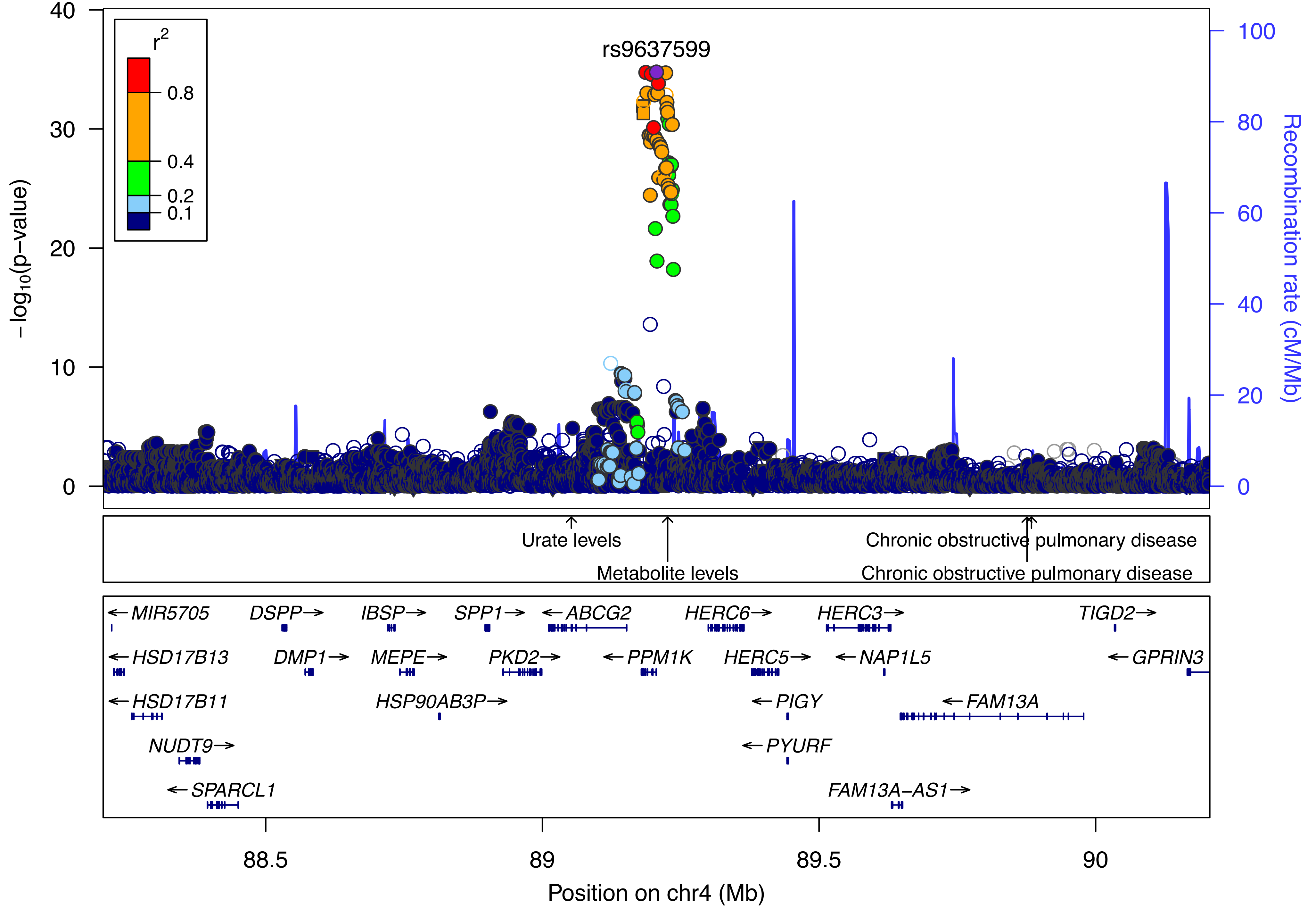

r2
0.8
0.4
0.2
0.1
rs9637599
−log10(p−value)
0
10
20
30
40
Recombination rate (cM/Mb)
0
20
40
60
80
100
Urate levels
Metabolite levels
Chronic obstructive pulmonary disease
Chronic obstructive pulmonary disease
←MIR5705
DSPP→
IBSP→
SPP1→
←ABCG2
HERC6→
HERC3→
TIGD2→
←HSD17B13
DMP1→
MEPE→
PKD2→
←PPM1K
HERC5→
←NAP1L5
←GPRIN3
←HSD17B11
HSP90AB3P→
←PIGY
←FAM13A
NUDT9→
←PYURF
←SPARCL1
FAM13A−AS1→
88.5
89
89.5
90
Position on chr4 (Mb)

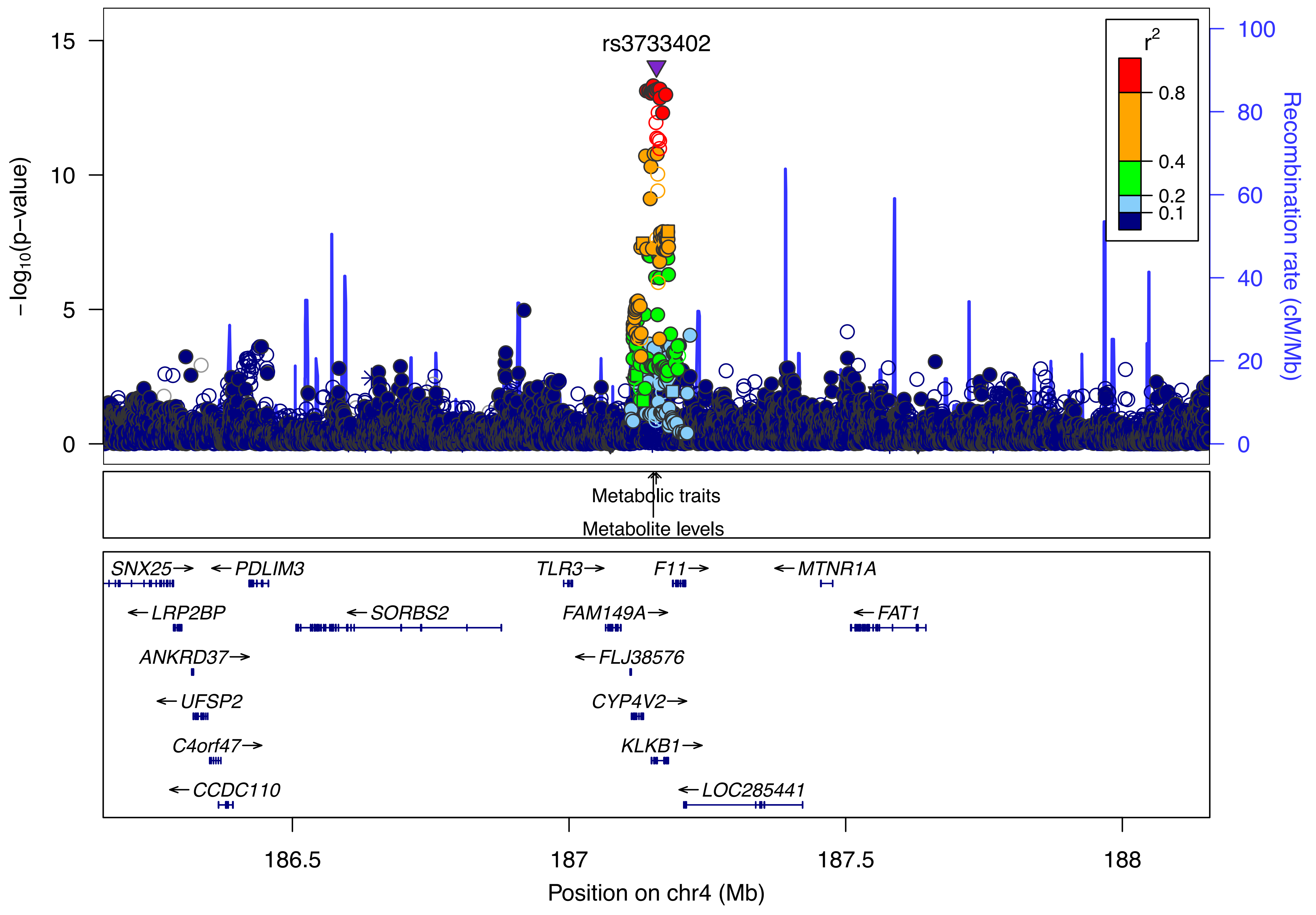

rs3733402
−log10(p−value)
15
10
5
0
r2
0.8
0.4
0.2
0.1
100
80
60
40
20
0
Recombination rate (cM/Mb)
Metabolic traits
Metabolite levels
SNX25→
←PDLIM3
TLR3→
F11→
←MTNR1A
←LRP2BP
←SORBS2
FAM149A→
←FAT1
ANKRD37→
←FLJ38576
←UFSP2
CYP4V2→
C4orf47→
KLKB1→
←CCDC110
←LOC285441
186.5
187
187.5
188
Position on chr4 (Mb)

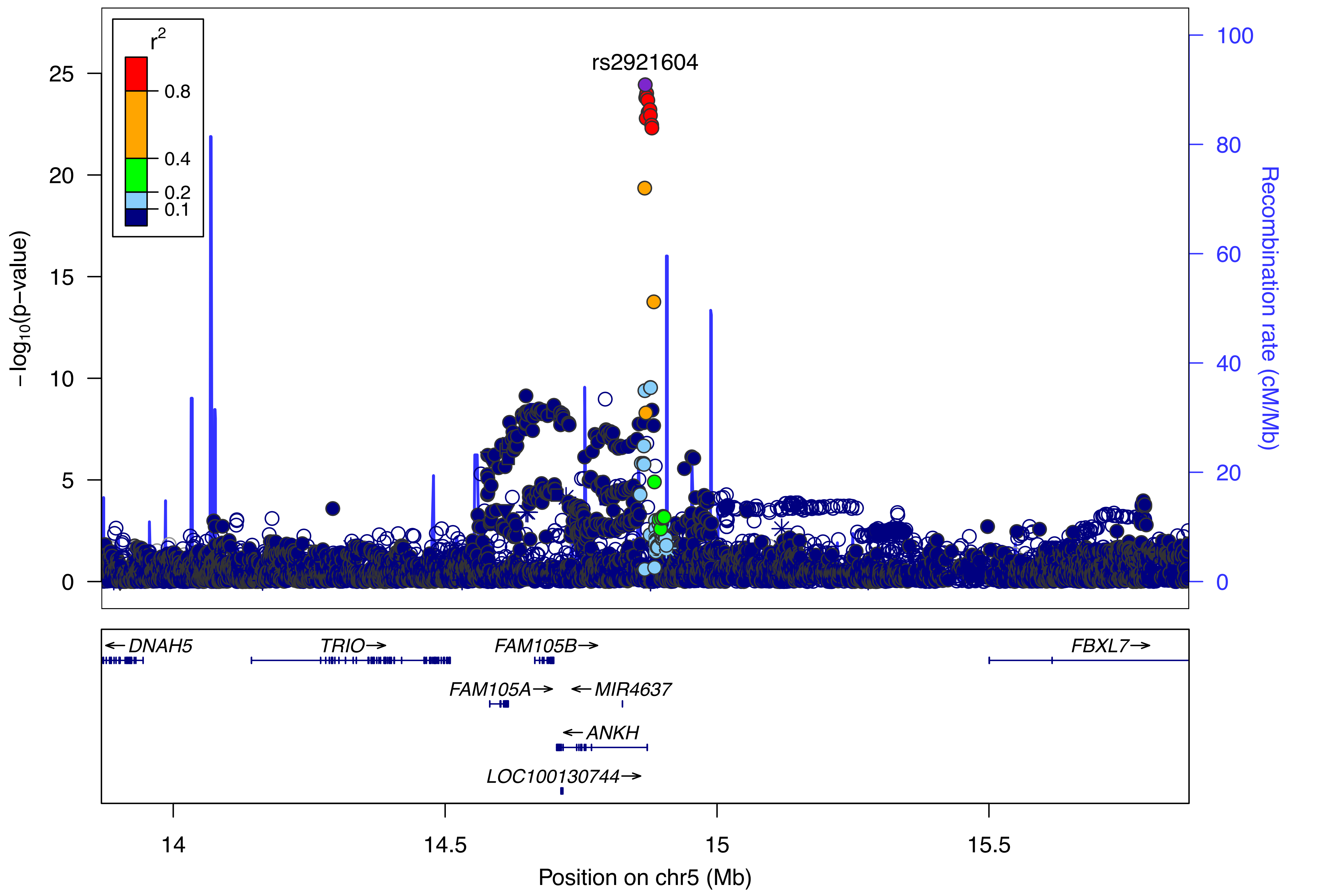

r2
0.8
0.4
0.2
0.1
rs2921604
−log10(p−value)
0
5
10
15
20
25
Recombination rate (cM/Mb)
0
20
40
60
80
100
←DNAH5
TRIO→
FAM105B→
FBXL7→
FAM105A→
←MIR4637
←ANKH
LOC100130744→
14
14.5
15
15.5
Position on chr5 (Mb)

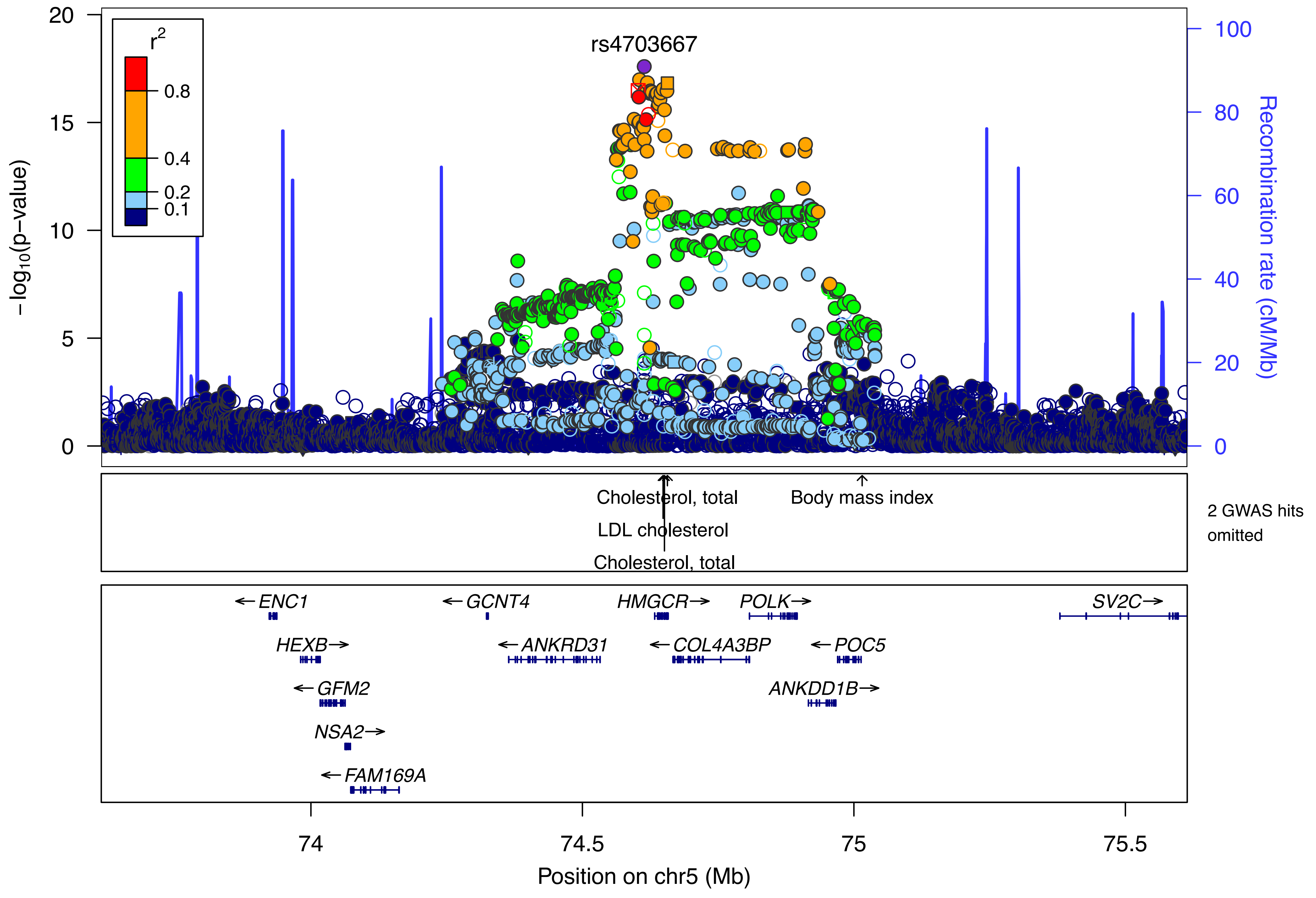
rs4703667
r2
0.8
0.4
0.2
0.1
−log10(p−value)
20
15
10
5
0
Recombination rate (cM/Mb)
100
80
60
40
20
0
Cholesterol, total
LDL cholesterol
Cholesterol, total
Body mass index
2 GWAS hits omitted
←ENC1
HEXB→
←GFM2
NSA2→
←FAM169A
←GCNT4
←ANKRD31
HMGCR→
←COL4A3BP
POLK→
←POC5
ANKDD1B→
SV2C→
74
74.5
75
75.5
Position on chr5 (Mb)

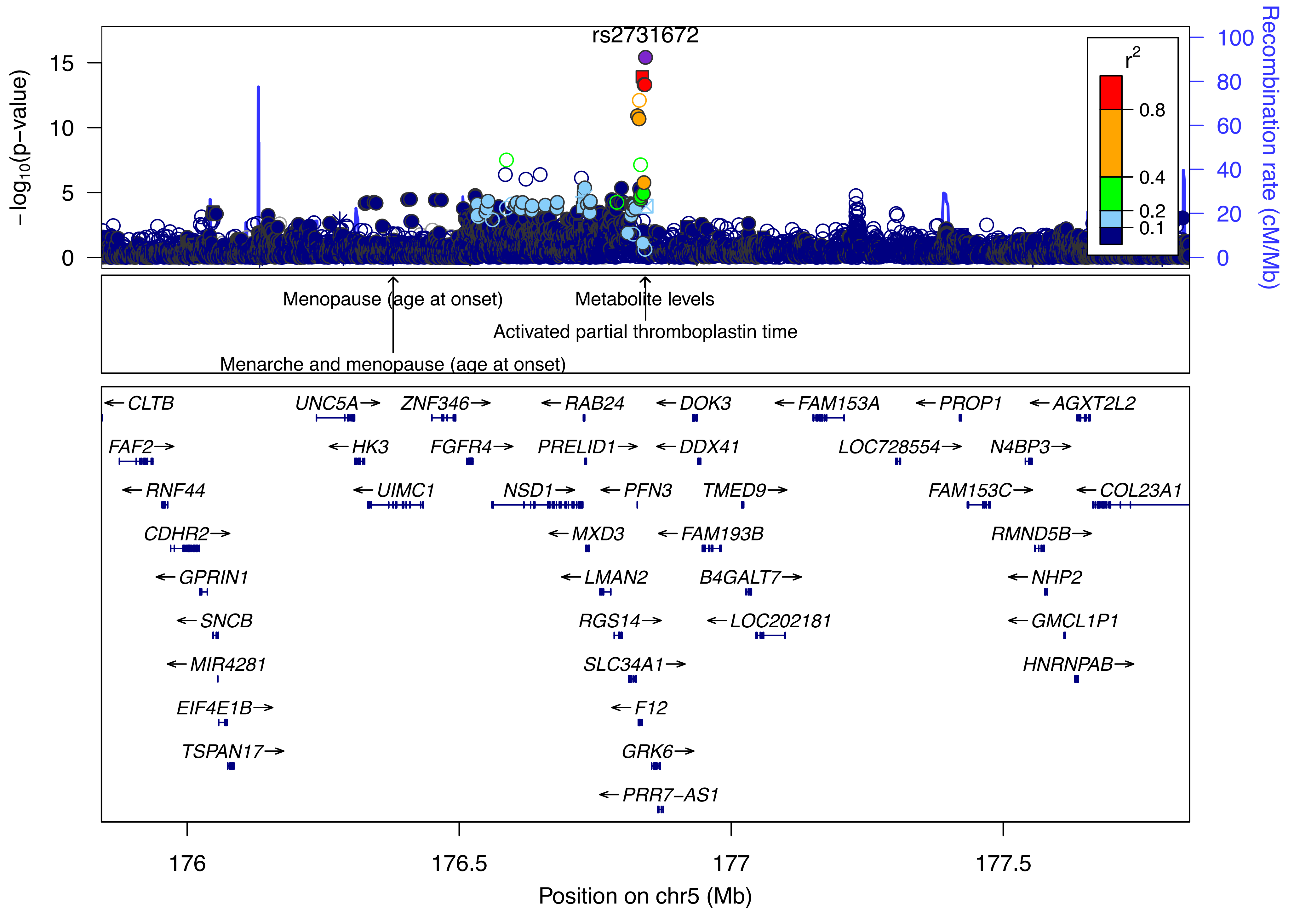

rs2731672
−log10(p−value)
0
5
10
15
Recombination rate (cM/Mb)
0
20
40
60
80
100
r2
0.8
0.4
0.2
0.1
Menopause (age at onset)
Metabolite levels
Activated partial thromboplastin time
Menarche and menopause (age at onset)
←CLTB
UNC5A→
ZNF346→
←RAB24
←DOK3
←FAM153A
←PROP1
←AGXT2L2
FAF2→
←HK3
FGFR4→
PRELID1→
←DDX41
LOC728554→
N4BP3→
←RNF44
←UIMC1
NSD1→
←PFN3
TMED9→
FAM153C→
←COL23A1
CDHR2→
←MXD3
←FAM193B
RMND5B→
←GPRIN1
←LMAN2
B4GALT7→
←NHP2
←SNCB
RGS14→
←LOC202181
←GMCL1P1
←MIR4281
SLC34A1→
HNRNPAB→
EIF4E1B→
←F12
TSPAN17→
GRK6→
←PRR7−AS1
176
176.5
177
177.5
Position on chr5 (Mb)

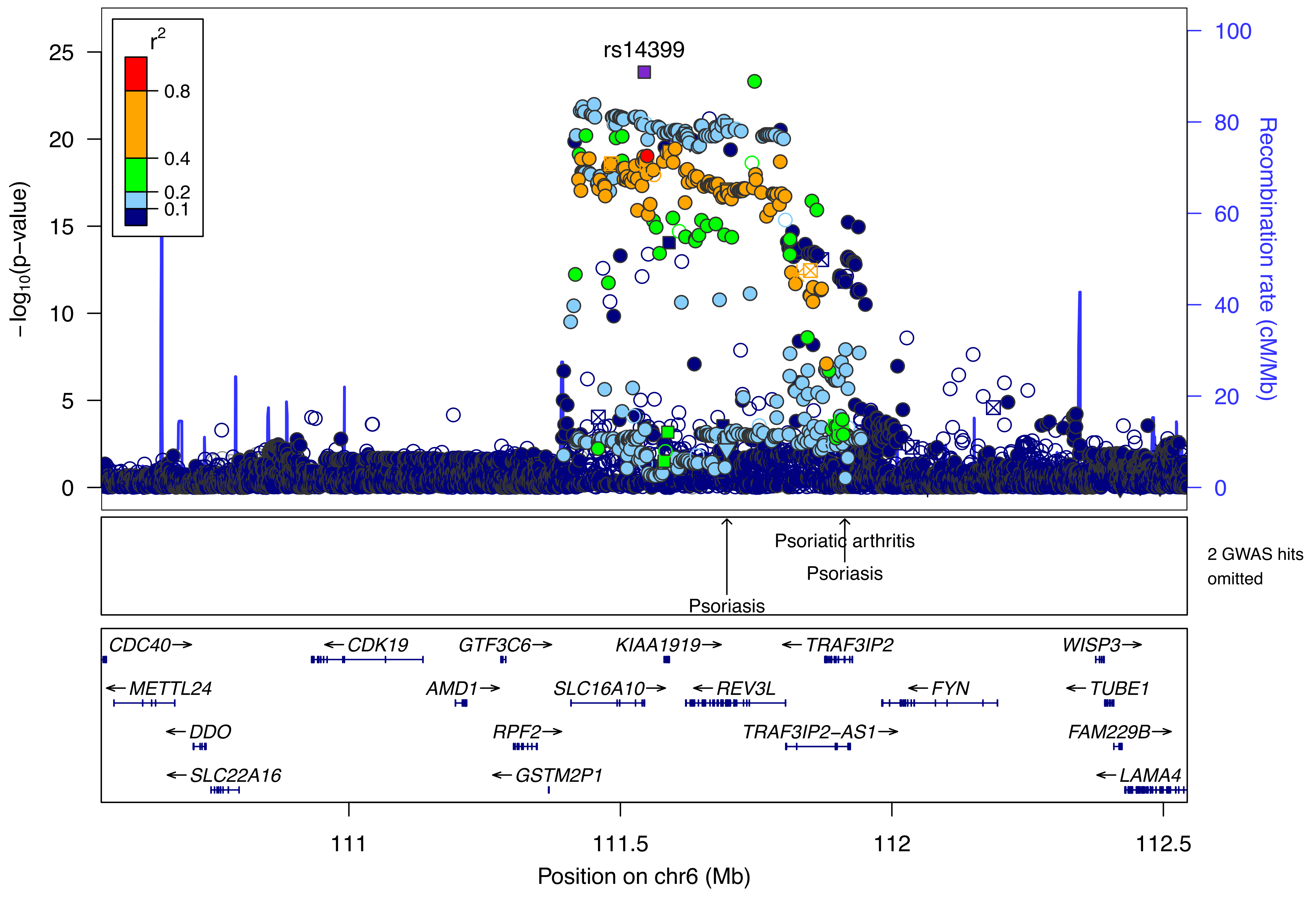

rs14399
r2
0.8
0.4
0.2
0.1
−log10(p−value)
0
5
10
15
20
25
Recombination rate (cM/Mb)
0
20
40
60
80
100
Psoriasis
Psoriatic arthritis
Psoriasis
2 GWAS hits omitted
CDC40→
←METTL24
←DDO
←SLC22A16
←CDK19
GTF3C6→
AMD1→
RPF2→
←GSTM2P1
KIAA1919→
SLC16A10→
←REV3L
←TRAF3IP2
TRAF3IP2−AS1→
←FYN
WISP3→
←TUBE1
FAM229B→
←LAMA4
111
111.5
112
112.5
Position on chr6 (Mb)

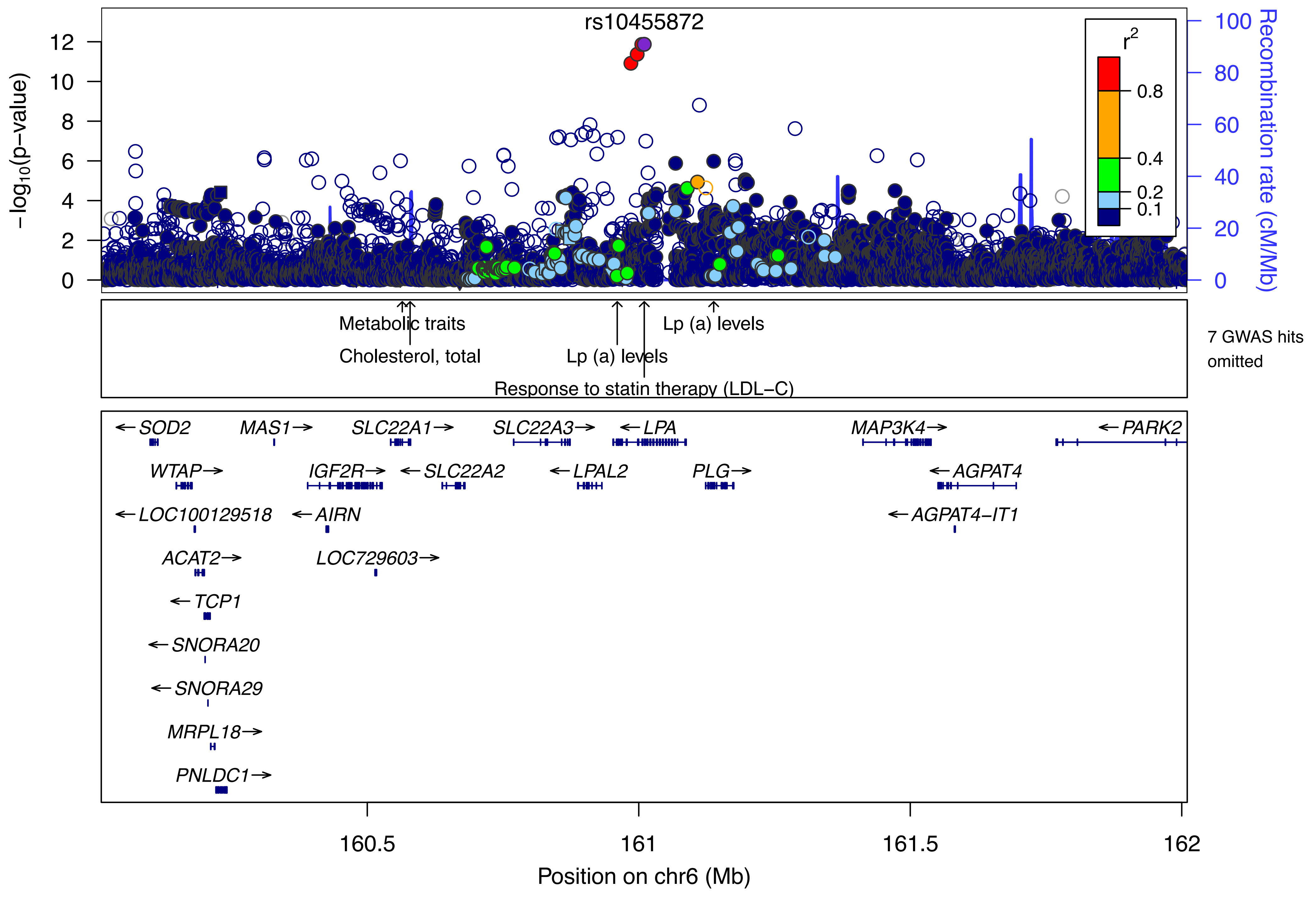

rs10455872
−log10(p−value)
Recombination rate (cM/Mb)
r2
0.8
0.4
0.2
0.1
Metabolic traits
Cholesterol, total
Lp (a) levels
Lp (a) levels
Response to statin therapy (LDL−C)
7 GWAS hits omitted
SOD2
MAS1
SLC22A1
SLC22A3
LPA
MAP3K4
PARK2
WTAP
IGF2R
SLC22A2
LPAL2
PLG
AGPAT4
LOC100129518
AIRN
AGPAT4−IT1
ACAT2
LOC729603
TCP1
SNORA20
SNORA29
MRPL18
PNLDC1
160.5
161
161.5
162
Position on chr6 (Mb)

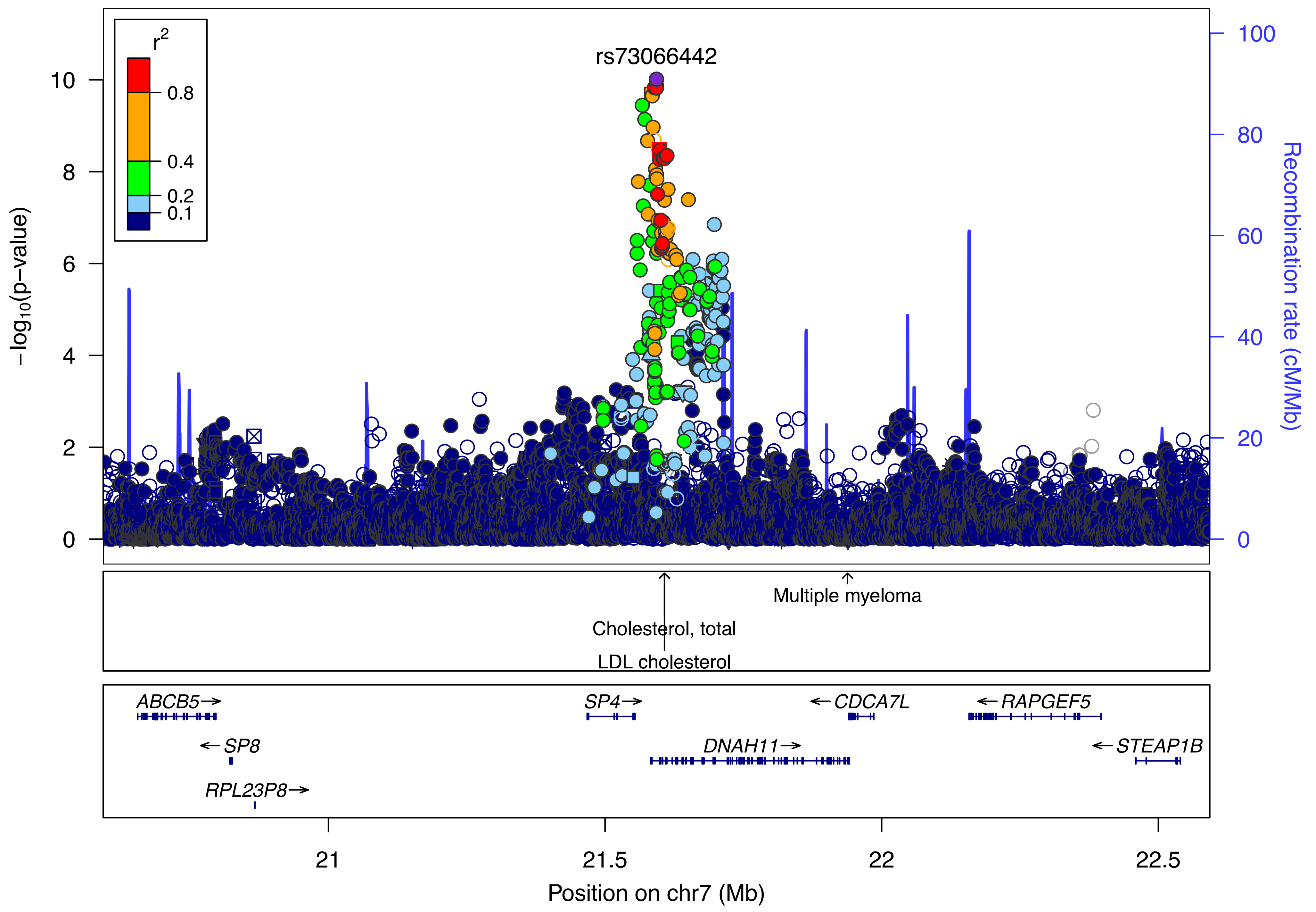

rs73066442
r^2
0.8
0.4
0.2
0.1
−log10(p−value)
0
2
4
6
8
10
Recombination rate (cM/Mb)
0
20
40
60
80
100
Multiple myeloma
Cholesterol, total
LDL cholesterol
ABCB5→
←SP8
RPL23P8→
SP4→
DNAH11→
←CDCA7L
←RAPGEF5
←STEAP1B
21
21.5
22
22.5
Position on chr7 (Mb)

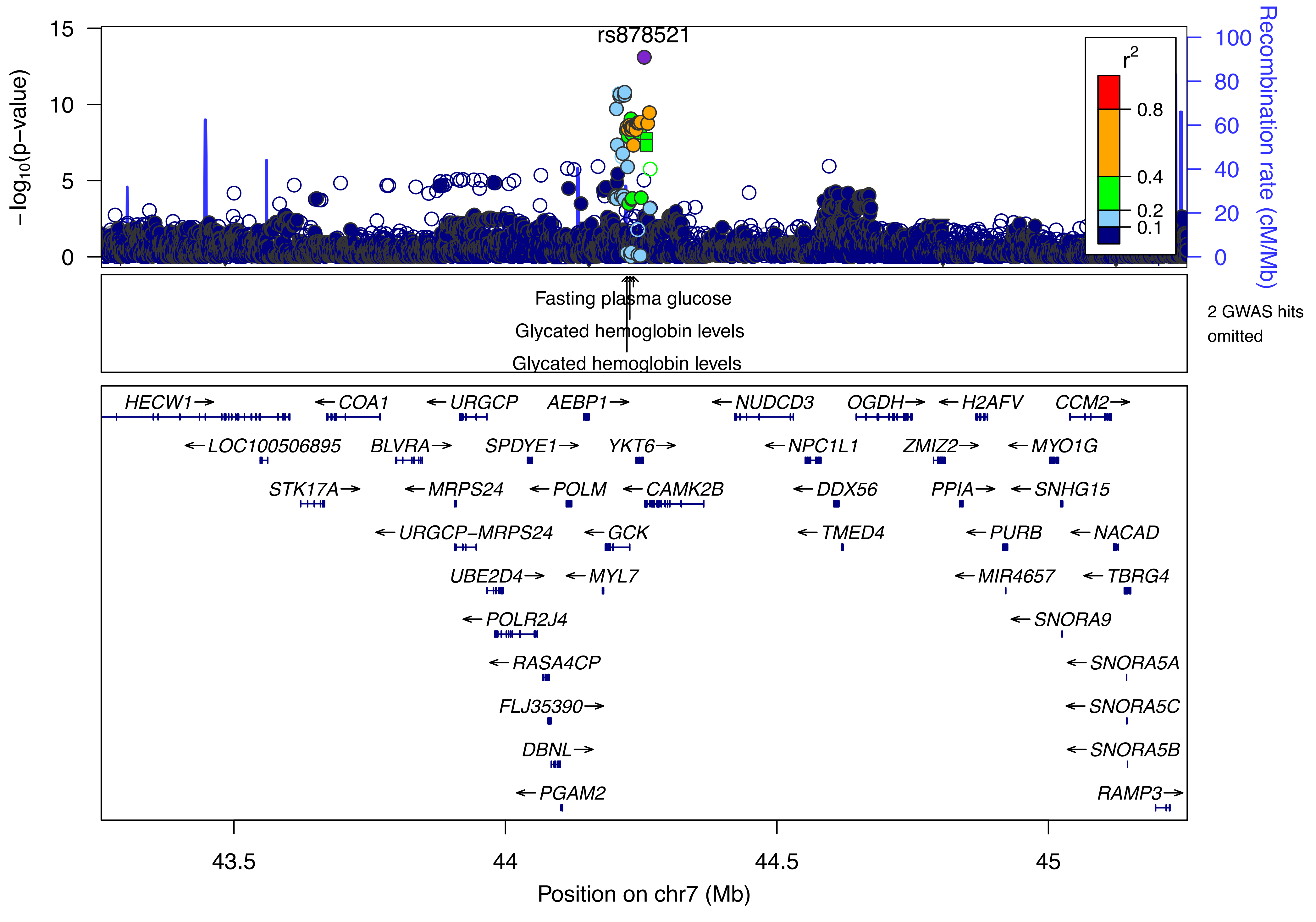
rs878521
−log10(p−value)
Recombination rate (cM/Mb)
r2
0.8
0.4
0.2
0.1
Fasting plasma glucose
Glycated hemoglobin levels
Glycated hemoglobin levels
2 GWAS hits omitted
HECW1
COA1
URGCP
AEBP1
NUDCD3
OGDH
H2AFV
CCM2
LOC100506895
BLVRA
SPDYE1
YKT6
NPC1L1
ZMIZ2
MYO1G
STK17A
MRPS24
POLM
CAMK2B
DDX56
PPIA
SNHG15
URGCP−MRPS24
GCK
TMED4
PURB
NACAD
UBE2D4
MYL7
MIR4657
TBRG4
POLR2J4
SNORA9
RASA4CP
SNORA5A
FLJ35390
SNORA5C
DBNL
SNORA5B
PGAM2
RAMP3
43.5
44
44.5
45
Position on chr7 (Mb)

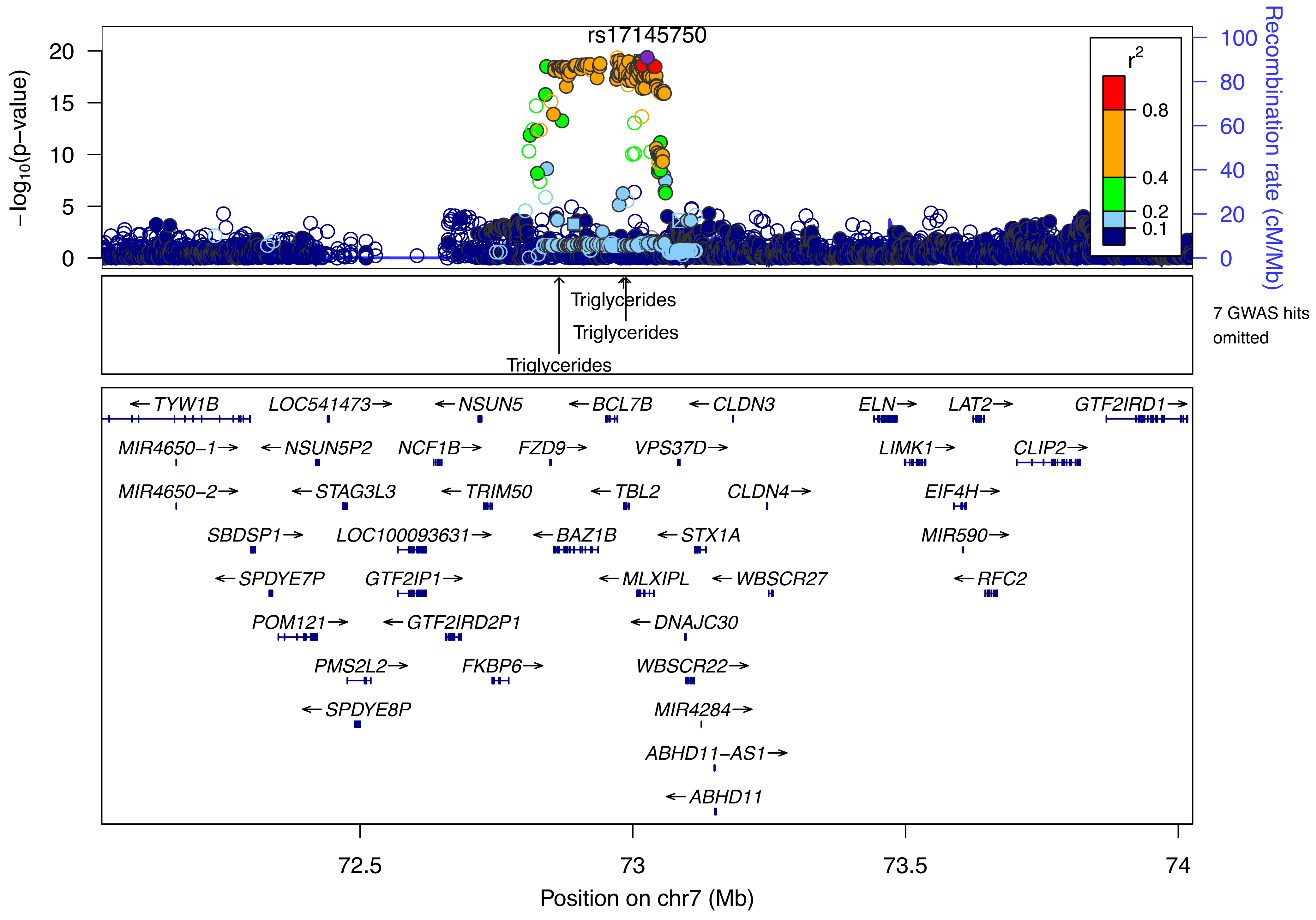
rs17145750
−log10(p−value)
20
15
10
5
0
r2
0.8
0.4
0.2
0.1
100
80
60
40
20
0
Recombination rate (cM/Mb)
Triglycerides
Triglycerides
Triglycerides
7 GWAS hits omitted
←TYW1B
LOC541473→
←NSUN5
←BCL7B
←CLDN3
ELN→
LAT2→
GTF2IRD1→
MIR4650−1→
←NSUN5P2
NCF1B→
FZD9→
VPS37D→
LIMK1→
CLIP2→
MIR4650−2→
←STAG3L3
←TRIM50
←TBL2
CLDN4→
EIF4H→
SBDSP1→
LOC100093631→
←BAZ1B
←STX1A
MIR590→
←SPDYE7P
GTF2IP1→
←MLXIPL
←WBSCR27
←RFC2
POM121→
←GTF2IRD2P1
←DNAJC30
PMS2L2→
FKBP6→
WBSCR22→
←SPDYE8P
MIR4284→
ABHD11−AS1→
←ABHD11
72.5
73
73.5
74
Position on chr7 (Mb)

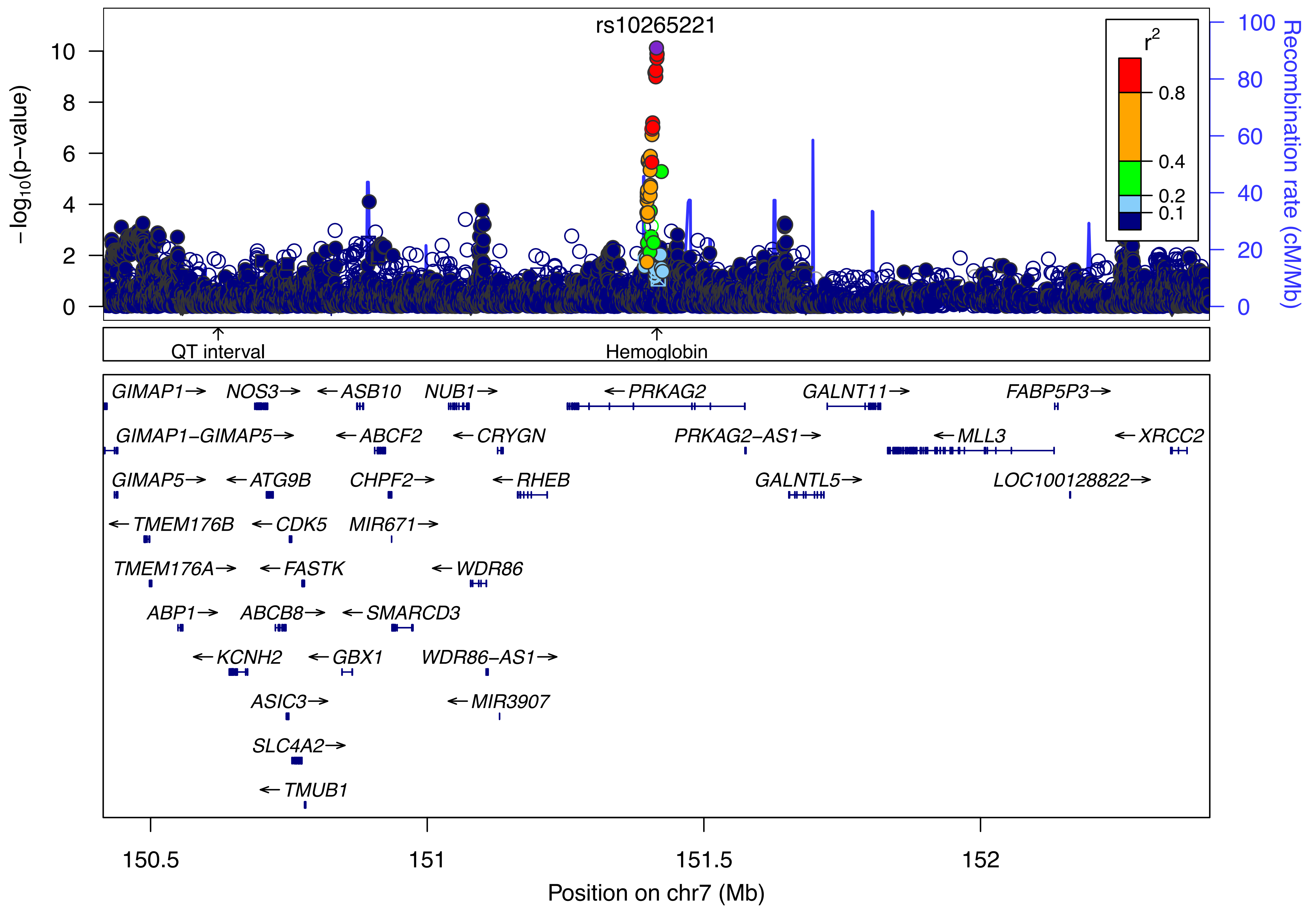
rs10265221
−log10(p−value)
Recombination rate (cM/Mb)
r2
0.8
0.4
0.2
0.1
QT interval
Hemoglobin
GIMAP1→
NOS3→
←ASB10
NUB1→
←PRKAG2
GALNT11→
FABP5P3→
GIMAP1−GIMAP5→
←ABCF2
←CRYGN
PRKAG2−AS1→
←MLL3
←XRCC2
GIMAP5→
←ATG9B
CHPF2→
←RHEB
GALNTL5→
LOC100128822→
←TMEM176B
←CDK5
MIR671→
TMEM176A→
←FASTK
←WDR86
ABP1→
ABCB8→
←SMARCD3
←KCNH2
←GBX1
WDR86−AS1→
ASIC3→
←MIR3907
SLC4A2→
←TMUB1
150.5
151
151.5
152
Position on chr7 (Mb)

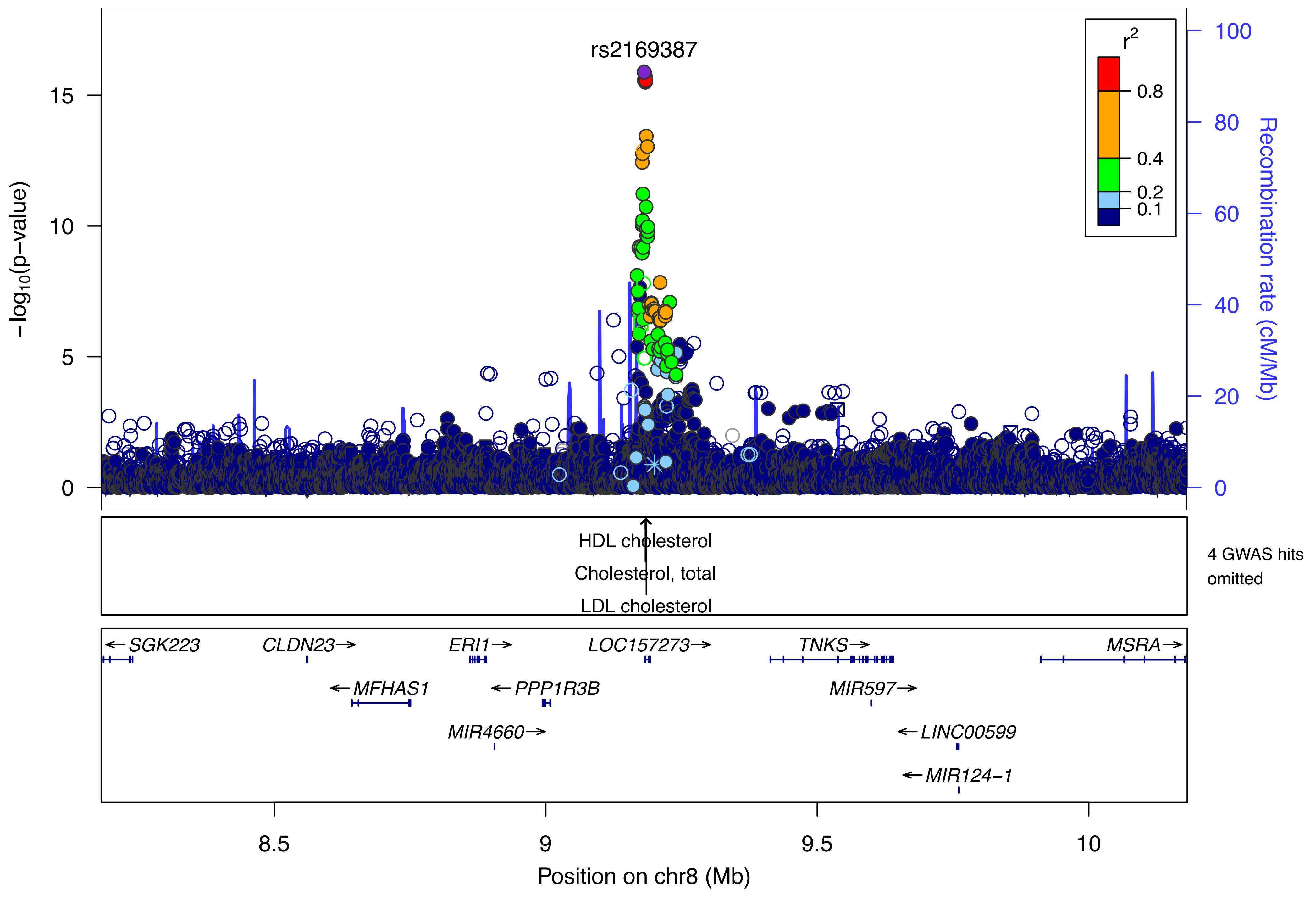
rs2169387
r2
0.8
0.4
0.2
0.1
−log10(p−value)
15
10
5
0
100
80
60
40
20
0
Recombination rate (cM/Mb)
HDL cholesterol
Cholesterol, total
LDL cholesterol
4 GWAS hits omitted
SGK223
CLDN23
ERI1
LOC157273
TNKS
MSRA
MFHAS1
PPP1R3B
MIR597
MIR4660
LINC00599
MIR124−1
8.5
9
9.5
10
Position on chr8 (Mb)

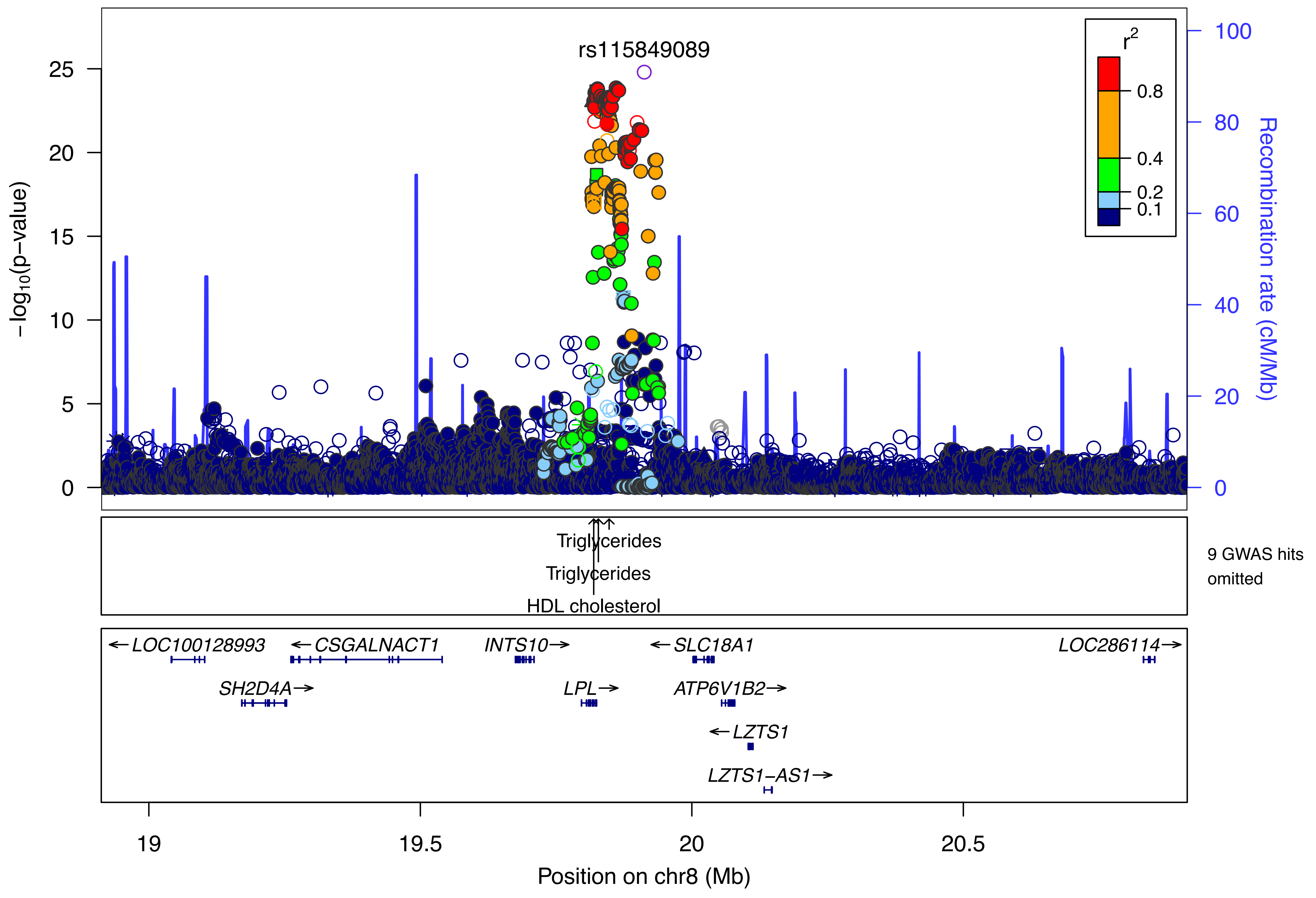

rs115849089
r2
0.8
0.4
0.2
0.1
−log10(p−value)
Recombination rate (cM/Mb)
Triglycerides
Triglycerides
HDL cholesterol
9 GWAS hits omitted
←LOC100128993
←CSGALNACT1
INTS10→
←SLC18A1
LOC286114→
SH2D4A→
LPL→
ATP6V1B2→
←LZTS1
LZTS1−AS1→
Position on chr8 (Mb)

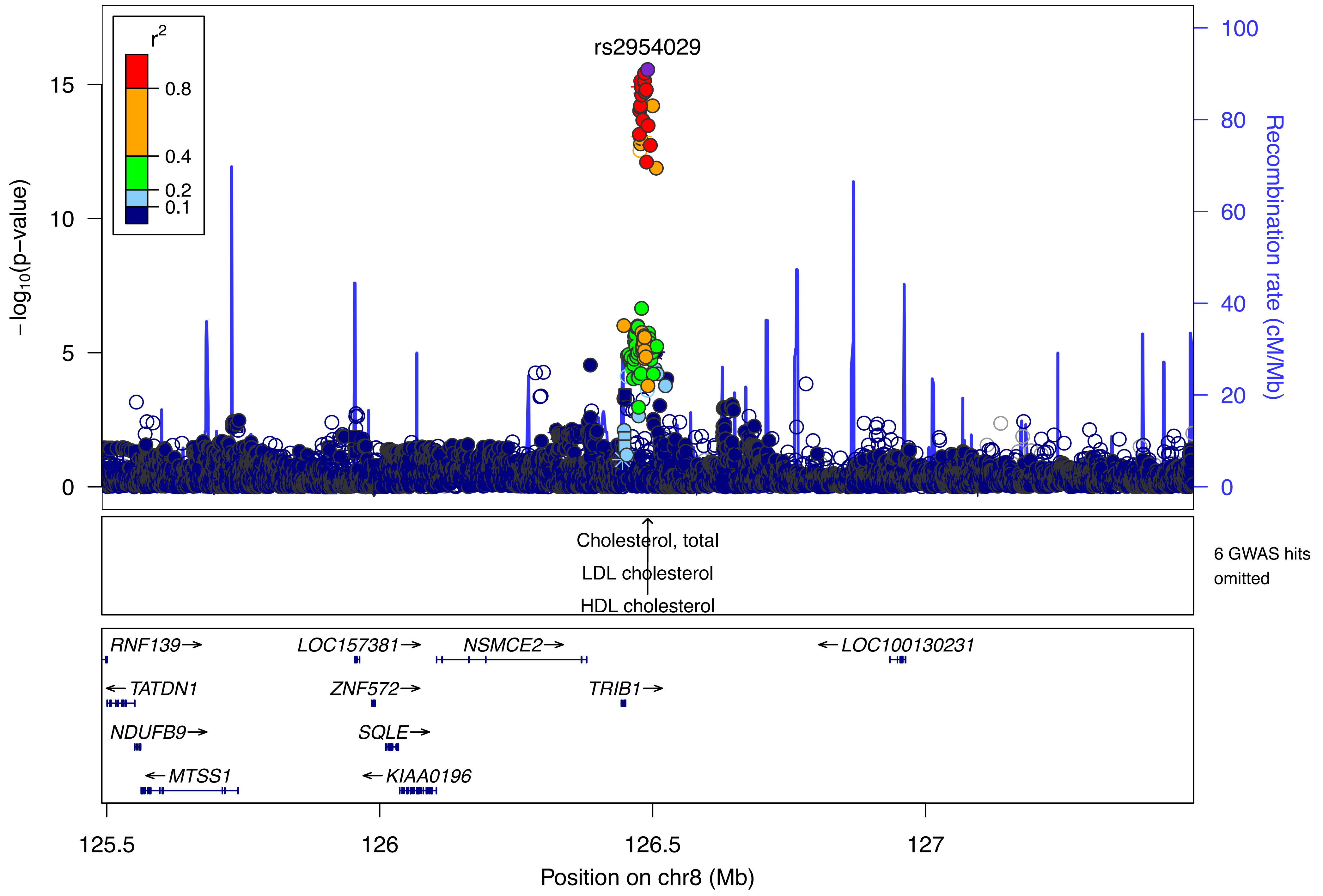

r2
0.8
0.4
0.2
0.1
rs2954029
−log10(p−value)
15
10
5
0
100
80
60
40
20
0
Recombination rate (cM/Mb)
Cholesterol, total
LDL cholesterol
HDL cholesterol
6 GWAS hits omitted
RNF139→
LOC157381→
NSMCE2→
←LOC100130231
←TATDN1
ZNF572→
TRIB1→
NDUFB9→
SQLE→
←MTSS1
←KIAA0196
125.5
126
126.5
127
Position on chr8 (Mb)

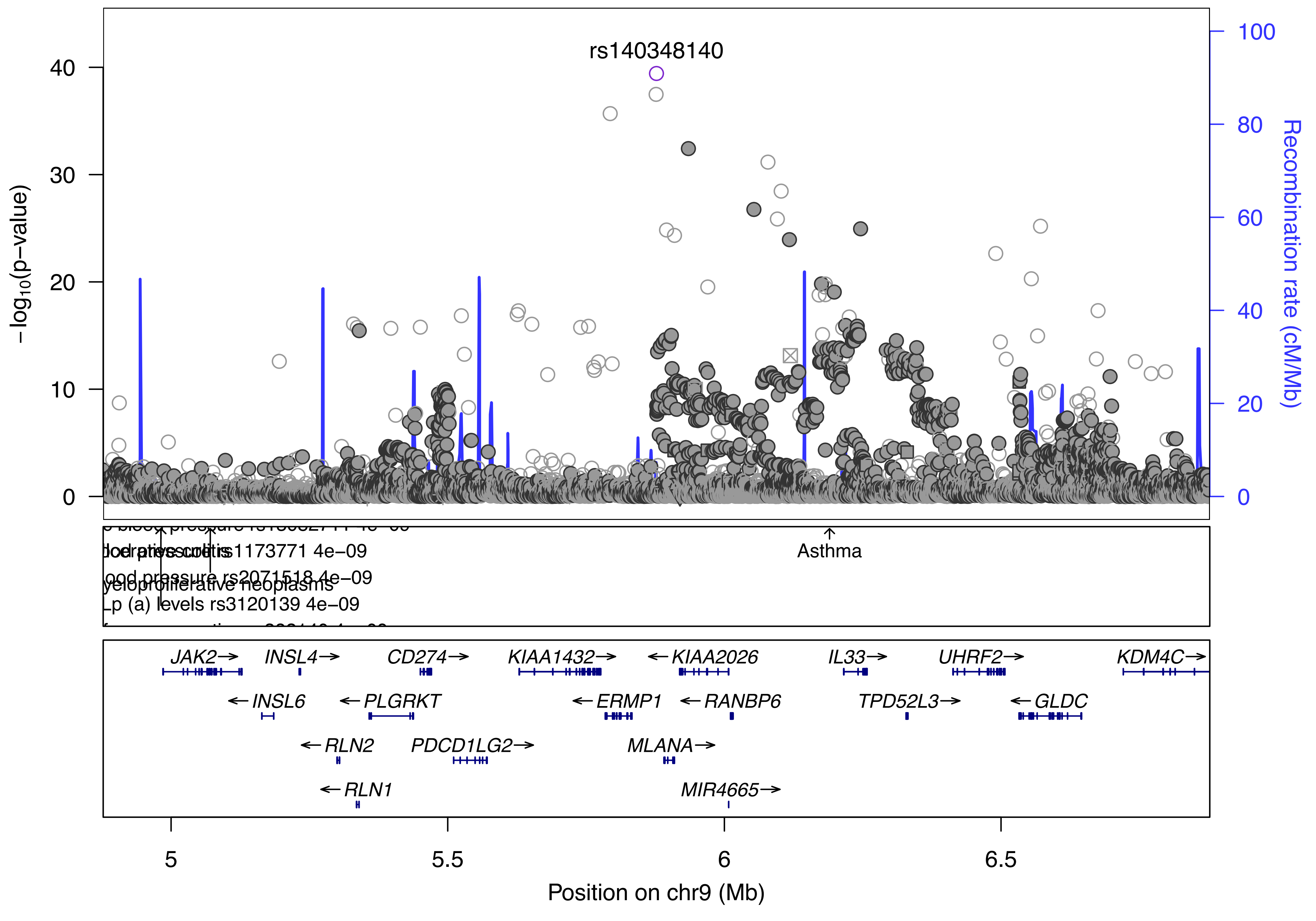
rs140348140
−log10(p−value)
Recombination rate (cM/Mb)
Asthma
Lp (a) levels rs3120139 4e−09
JAK2→
INSL4→
CD274→
KIAA1432→
←KIAA2026
IL33→
UHRF2→
KDM4C→
←INSL6
←PLGRKT
←ERMP1
←RANBP6
TPD52L3→
←GLDC
←RLN2
PDCD1LG2→
MLANA→
←RLN1
MIR4665→
5
5.5
6
6.5
Position on chr9 (Mb)

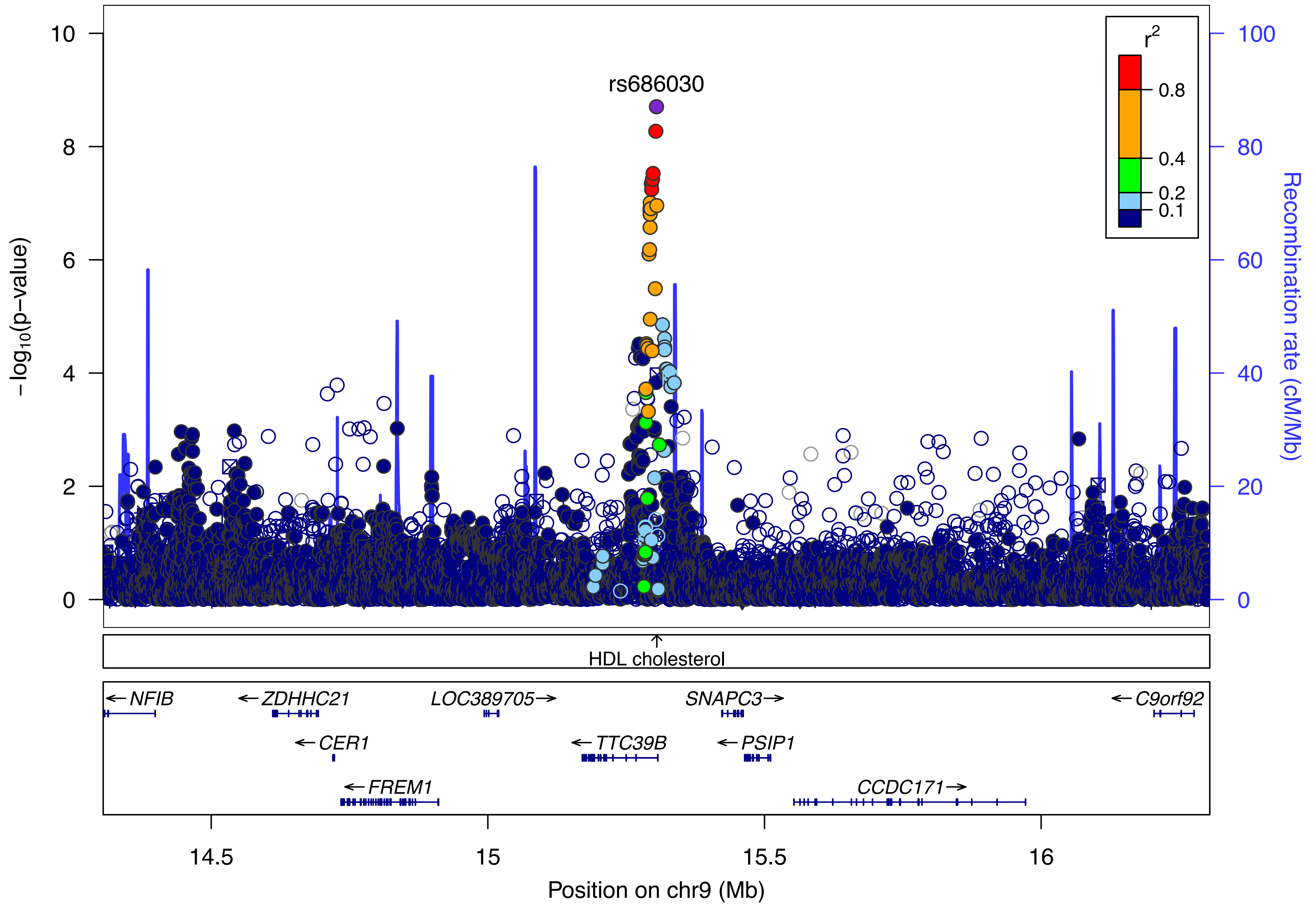

rs686030
−log10(p−value)
Recombination rate (cM/Mb)
r2
0.8
0.4
0.2
0.1
HDL cholesterol
← NFIB
← ZDHHC21
LOC389705 →
SNAPC3 →
← C9orf92
← CER1
← TTC39B
← PSIP1
← FREM1
CCDC171 →
14.5
15
15.5
16
Position on chr9 (Mb)

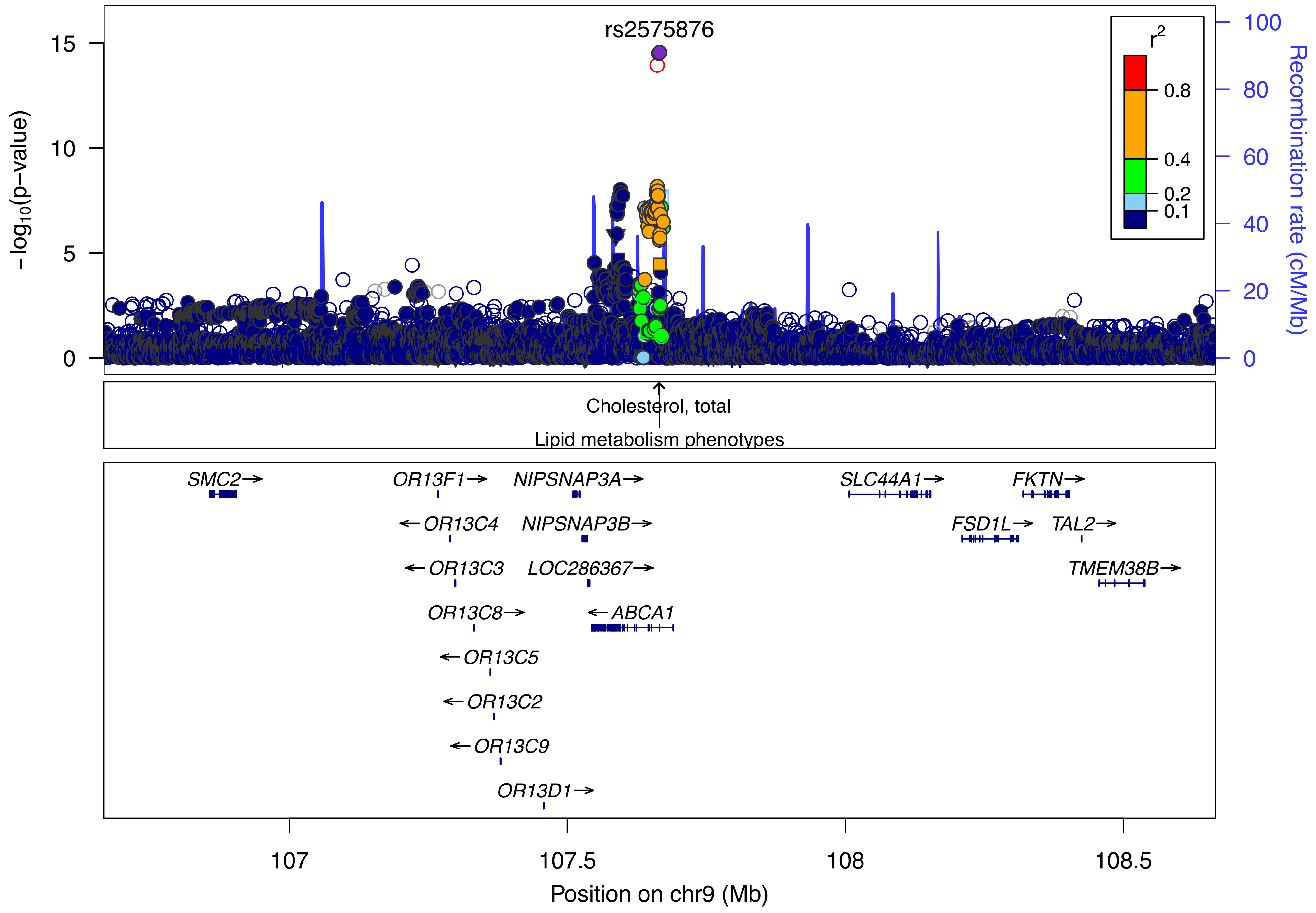

rs2575876
−log10(p−value)
15
10
5
0
Recombination rate (cM/Mb)
100
80
60
40
20
0
r2
0.8
0.4
0.2
0.1
Cholesterol, total
Lipid metabolism phenotypes
SMC2→
OR13F1→
NIPSNAP3A→
←OR13C4
NIPSNAP3B→
←OR13C3
LOC286367→
OR13C8→
←ABCA1
←OR13C5
←OR13C2
←OR13C9
OR13D1→
SLC44A1→
FKTN→
FSD1L→
TAL2→
TMEM38B→
107
107.5
108
108.5
Position on chr9 (Mb)

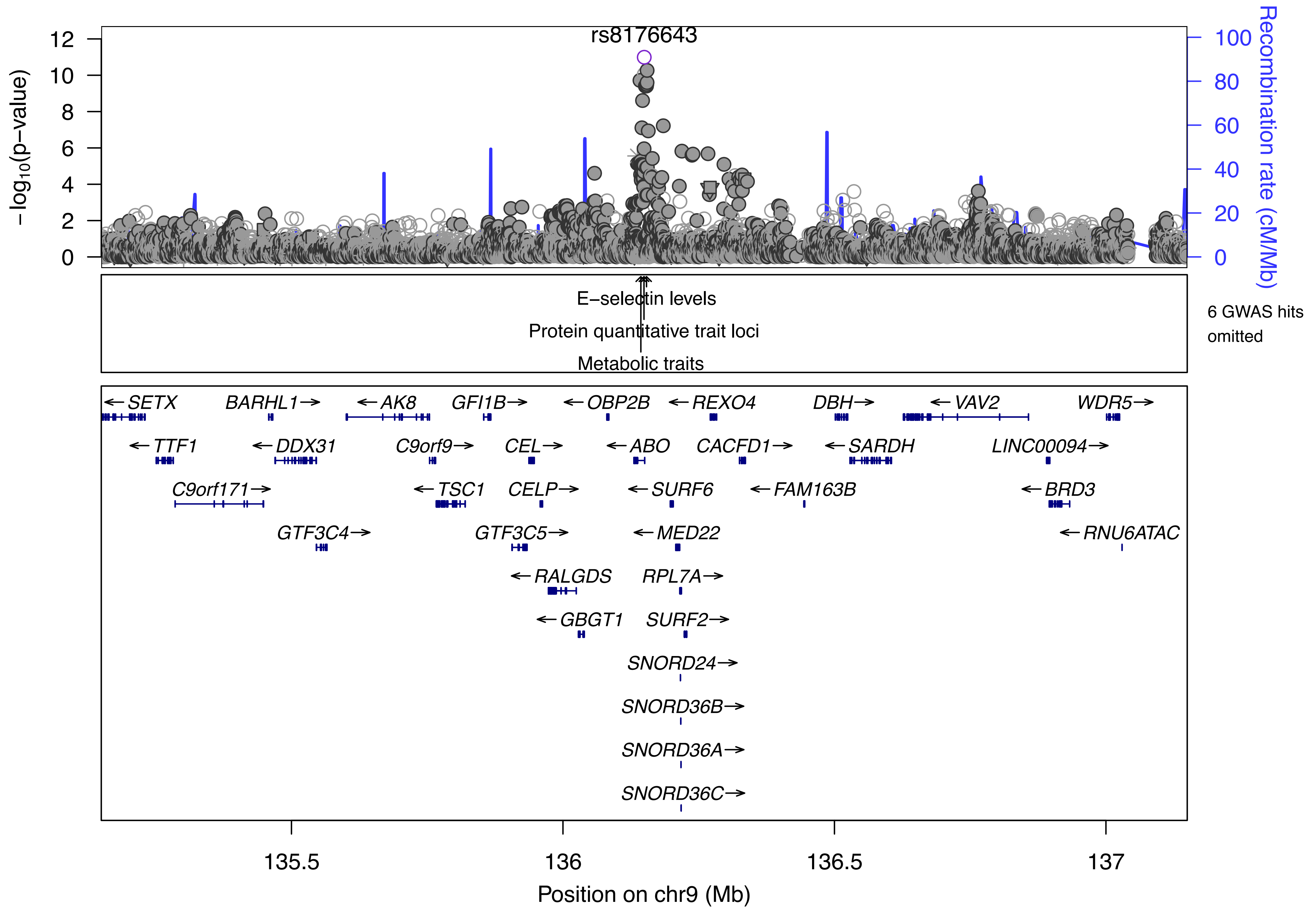

rs8176643
−log10(p−value)
Recombination rate (cM/Mb)
E−selectin levels
Protein quantitative trait loci
Metabolic traits
6 GWAS hits omitted
←SETX
BARHL1→
←AK8
GFI1B→
←OBP2B
←REXO4
DBH→
←VAV2
WDR5→
←TTF1
←DDX31
C9orf9→
CEL→
←ABO
CACFD1→
←SARDH
LINC00094→
C9orf171→
←TSC1
CELP→
←SURF6
←FAM163B
←BRD3
GTF3C4→
GTF3C5→
←MED22
←RNU6ATAC
←RALGDS
RPL7A→
←GBGT1
SURF2→
SNORD24→
SNORD36B→
SNORD36A→
SNORD36C→
135.5
136
136.5
137
Position on chr9 (Mb)

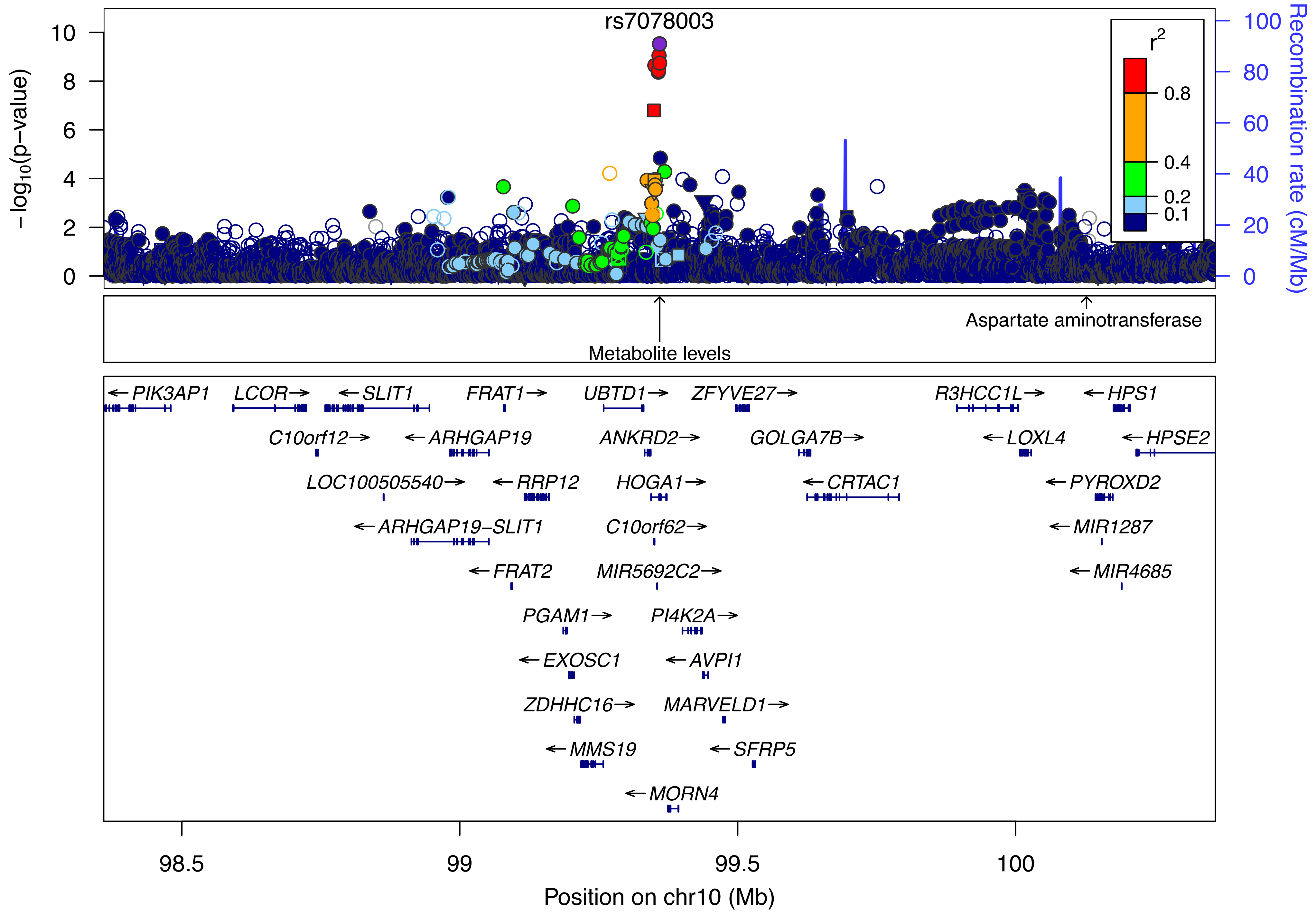
rs7078003
−log10(p−value)
10
8
6
4
2
0
Recombination rate (cM/Mb)
100
80
60
40
20
0
r2
0.8
0.4
0.2
0.1
Metabolite levels
Aspartate aminotransferase
←PIK3AP1
LCOR→
←SLIT1
FRAT1→
UBTD1→
ZFYVE27→
R3HCC1L→
←HPS1
C10orf12→
←ARHGAP19
ANKRD2→
GOLGA7B→
←LOXL4
←HPSE2
LOC100505540→
←RRP12
HOGA1→
←CRTAC1
←PYROXD2
←ARHGAP19−SLIT1
C10orf62→
←MIR1287
←FRAT2
MIR5692C2→
←MIR4685
PGAM1→
PI4K2A→
←EXOSC1
←AVPI1
ZDHHC16→
MARVELD1→
←MMS19
←SFRP5
←MORN4
98.5
99
99.5
100
Position on chr10 (Mb)

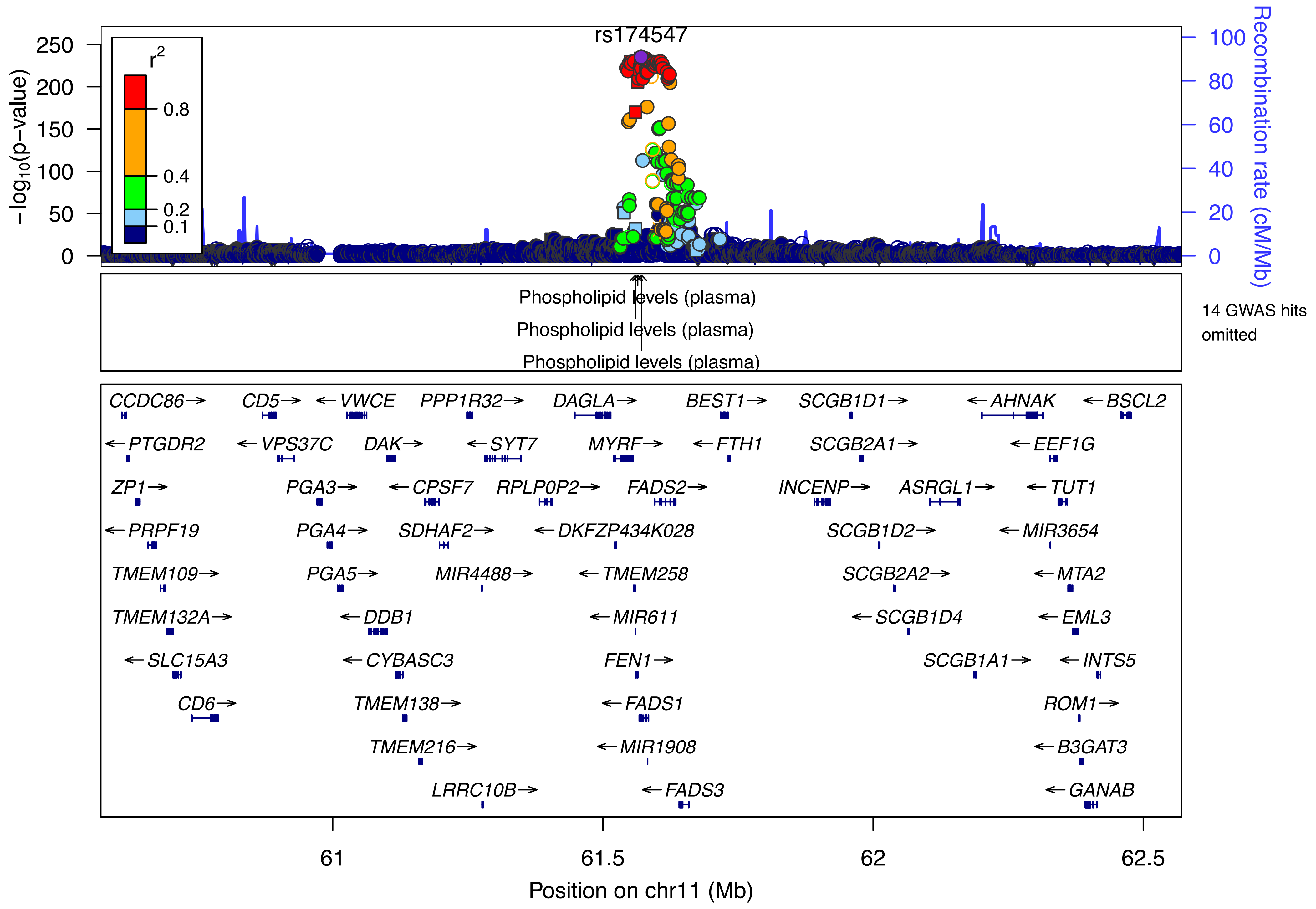
rs174547
$-\log_{10}$(p−value)
250
200
150
100
50
0
r^2
0.8
0.4
0.2
0.1
Recombination rate (cM/Mb)
100
80
60
40
20
0
Phospholipid levels (plasma)
Phospholipid levels (plasma)
Phospholipid levels (plasma)
14 GWAS hits omitted
CCDC86→
CD5→
←VWCE
PPP1R32→
DAGLA→
BEST1→
SCGB1D1→
←AHNAK
←BSCL2
←PTGDR2
←VPS37C
DAK→
←SYT7
MYRF→
←FTH1
SCGB2A1→
←EEF1G
ZP1→
PGA3→
←CPSF7
RPLP0P2→
FADS2→
INCENP→
ASRGL1→
←TUT1
←PRPF19
PGA4→
SDHAF2→
←DKFZP434K028
SCGB1D2→
←MIR3654
TMEM109→
PGA5→
MIR4488→
←TMEM258
SCGB2A2→
←MTA2
TMEM132A→
←DDB1
←MIR611
←SCGB1D4
←EML3
←SLC15A3
←CYBASC3
FEN1→
SCGB1A1→
←INTS5
CD6→
TMEM138→
←FADS1
ROM1→
TMEM216→
←MIR1908
←B3GAT3
LRRC10B→
←FADS3
←GANAB
61
61.5
62
62.5
Position on chr11 (Mb)

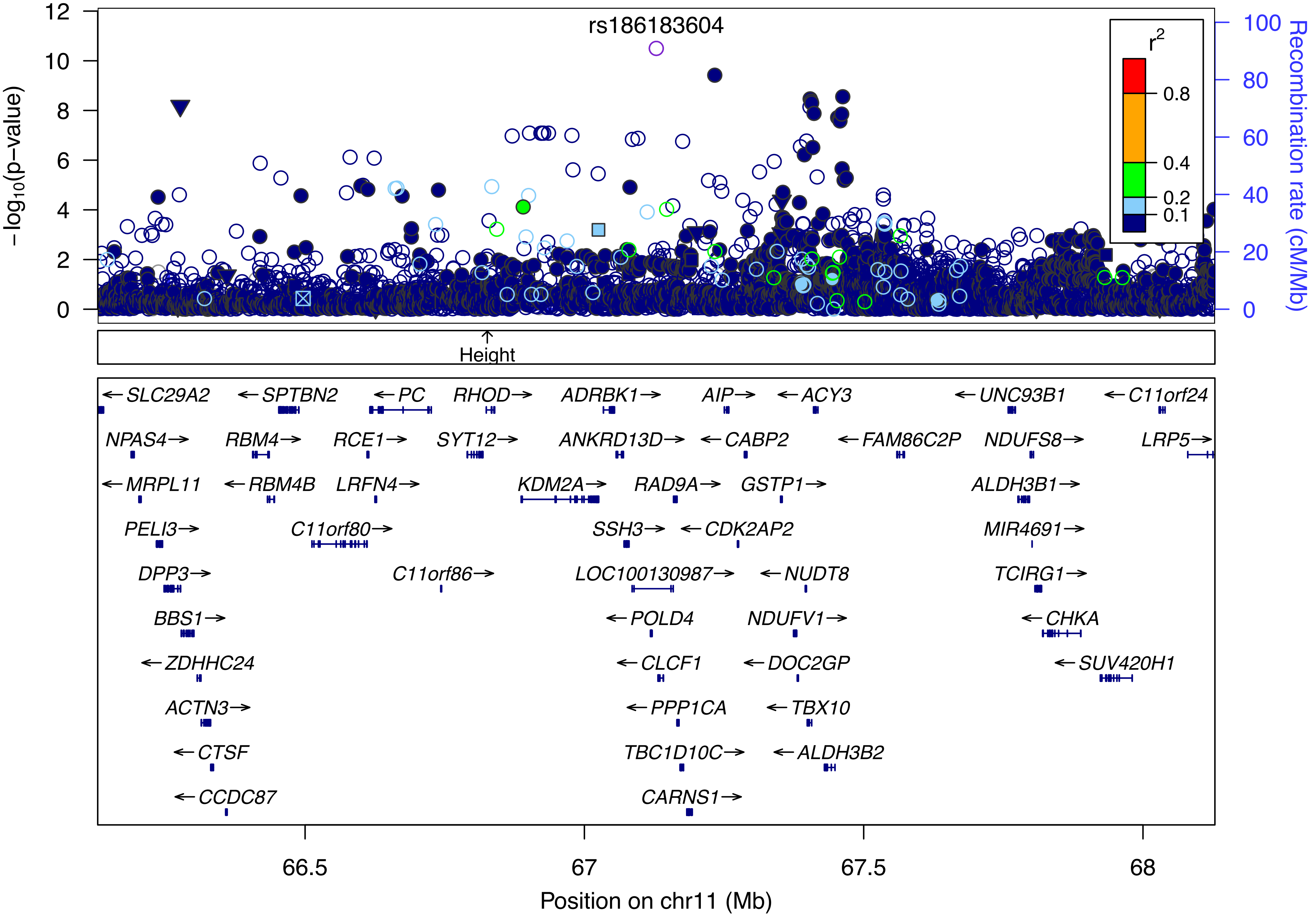

rs186183604
−log10(p−value)
Recombination rate (cM/Mb)
r2
0.8
0.4
0.2
0.1
Height
←SLC29A2
←SPTBN2
←PC
RHOD→
ADRBK1→
AIP→
←ACY3
←UNC93B1
←C11orf24
NPAS4→
RBM4→
RCE1→
SYT12→
ANKRD13D→
←CABP2
←FAM86C2P
NDUFS8→
LRP5→
←MRPL11
←RBM4B
LRFN4→
KDM2A→
RAD9A→
GSTP1→
ALDH3B1→
PELI3→
C11orf80→
SSH3→
←CDK2AP2
MIR4691→
DPP3→
C11orf86→
LOC100130987→
←NUDT8
TCIRG1→
BBS1→
←POLD4
NDUFV1→
←CHKA
←ZDHHC24
←CLCF1
←DOC2GP
←SUV420H1
ACTN3→
←PPP1CA
←TBX10
←CTSF
TBC1D10C→
←ALDH3B2
←CCDC87
CARNS1→
66.5
67
67.5
68
Position on chr11 (Mb)

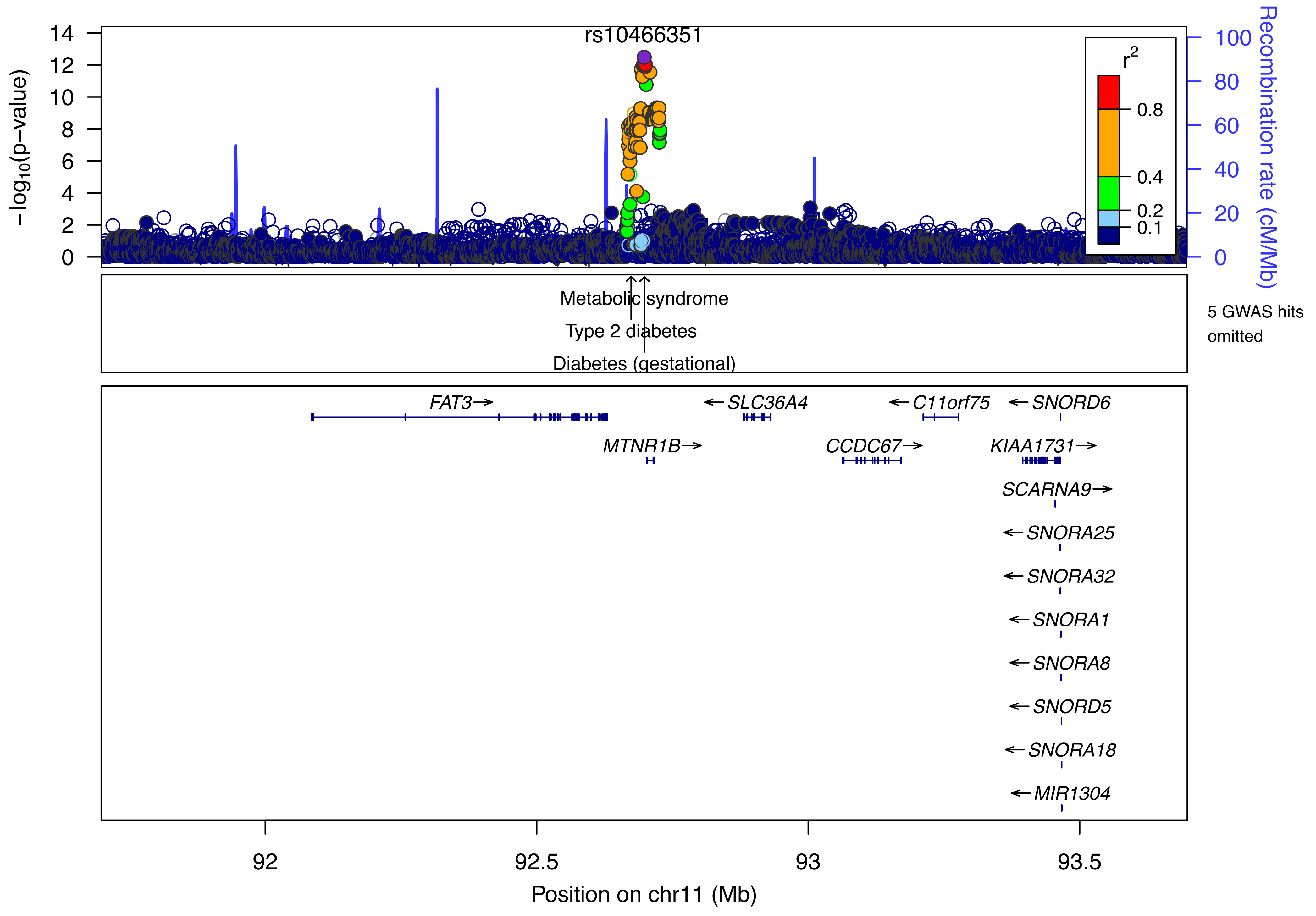

rs10466351
−log10(p−value)
0
2
4
6
8
10
12
14
Recombination rate (cM/Mb)
0
20
40
60
80
100
r2
0.8
0.4
0.2
0.1
Metabolic syndrome
Type 2 diabetes
Diabetes (gestational)
5 GWAS hits omitted
FAT3→
←SLC36A4
←C11orf75
←SNORD6
MTNR1B→
CCDC67→
KIAA1731→
SCARNA9→
←SNORA25
←SNORA32
←SNORA1
←SNORA8
←SNORD5
←SNORA18
←MIR1304
92
92.5
93
93.5
Position on chr11 (Mb)

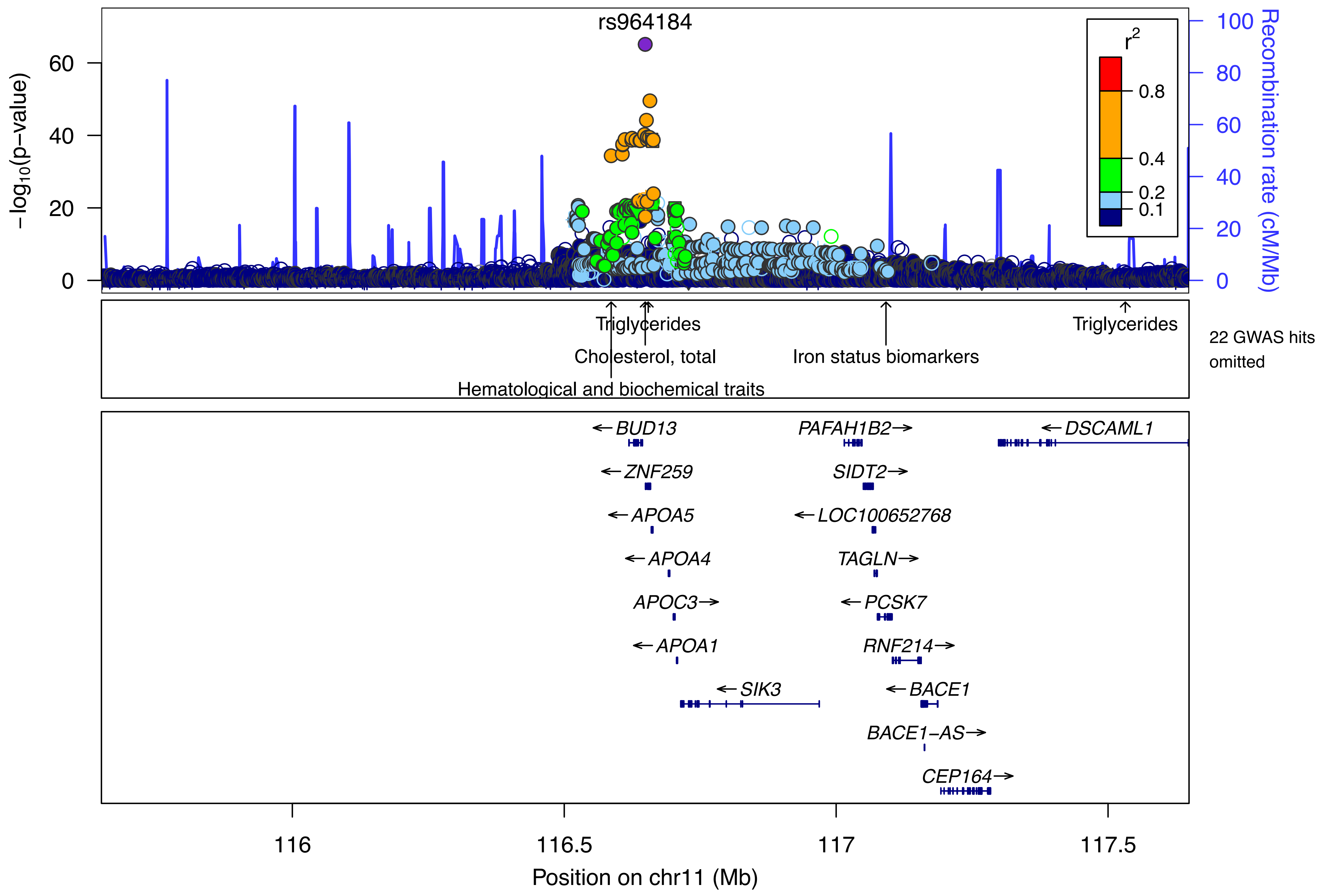
rs964184
−log10(p−value)
0
20
40
60
Recombination rate (cM/Mb)
0
20
40
60
80
100
r2
0.8
0.4
0.2
0.1
Triglycerides
Cholesterol, total
Hematological and biochemical traits
Iron status biomarkers
Triglycerides
22 GWAS hits omitted
←BUD13
←ZNF259
←APOA5
←APOA4
APOC3→
←APOA1
←SIK3
PAFAH1B2→
SIDT2→
←LOC100652768
TAGLN→
←PCSK7
RNF214→
←BACE1
BACE1−AS→
CEP164→
←DSCAML1
116
116.5
117
117.5
Position on chr11 (Mb)

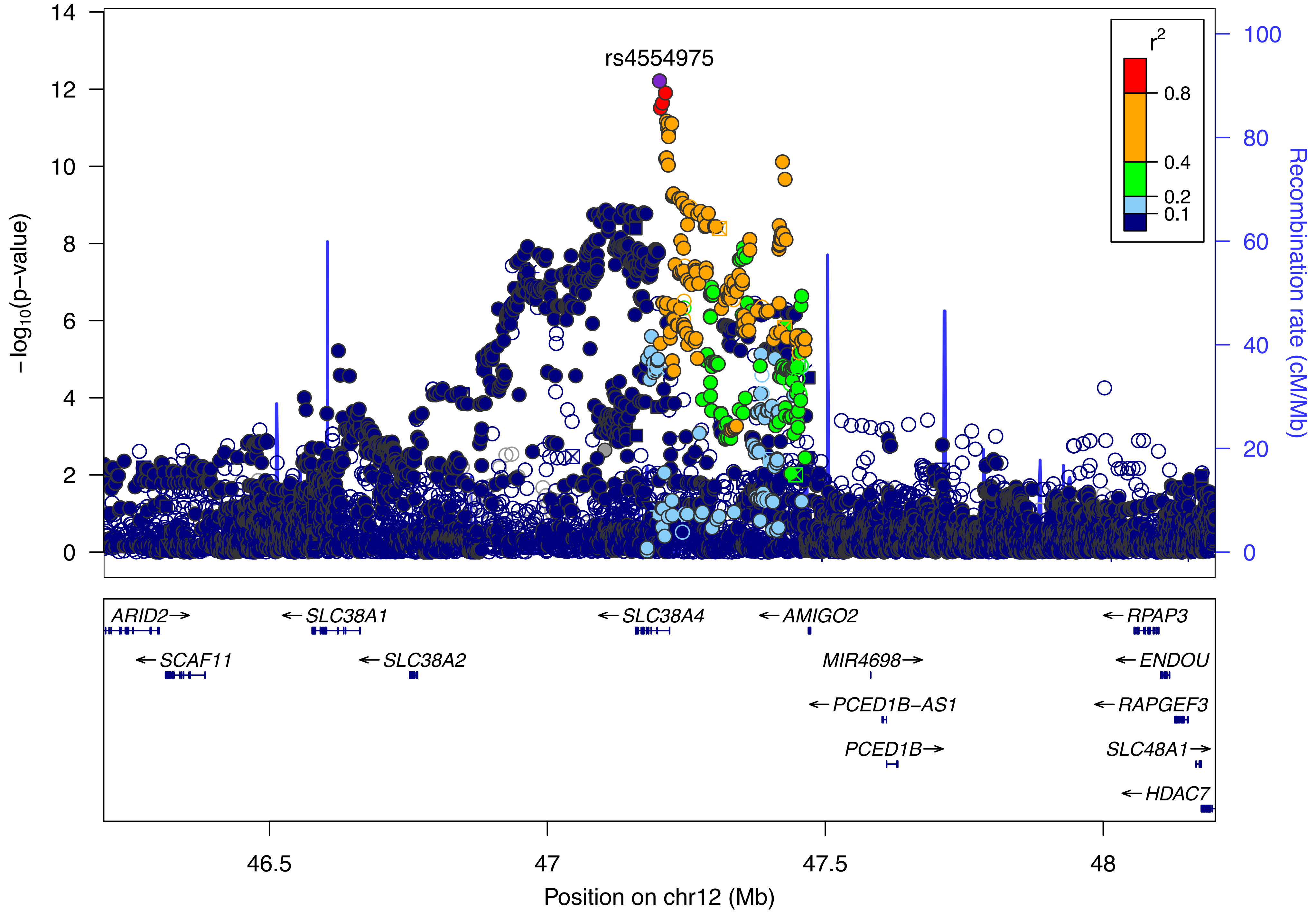
rs4554975
-log10(p-value)
Recombination rate (cM/Mb)
r2
0.8
0.4
0.2
0.1
ARID2→
←SCAF11
←SLC38A1
←SLC38A2
←SLC38A4
←AMIGO2
MIR4698→
←PCED1B–AS1
PCED1B→
←RPAP3
←ENDOU
←RAPGEF3
SLC48A1→
←HDAC7
46.5
47
47.5
48
Position on chr12 (Mb)

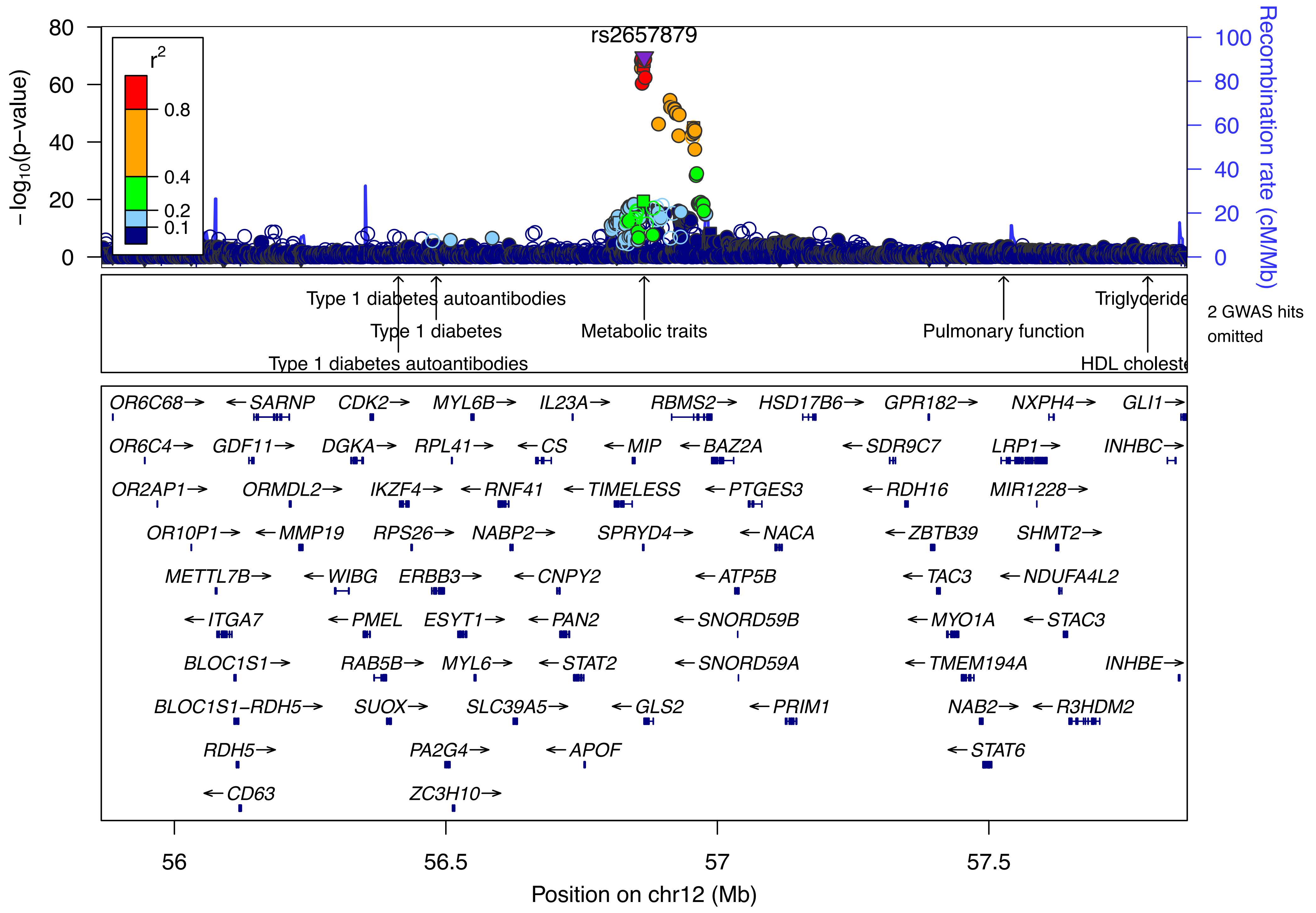
rs2657879
r^2
0.8
0.4
0.2
0.1
−log10(p−value)
Recombination rate (cM/Mb)
Type 1 diabetes autoantibodies
Type 1 diabetes
Type 1 diabetes autoantibodies
Metabolic traits
Pulmonary function
Triglyceride
HDL choleste
2 GWAS hits omitted
OR6C68→ ←SARNP CDK2→ MYL6B→ IL23A→ RBMS2→ HSD17B6→ GPR182→ NXPH4→ GLI1→
OR6C4→ GDF11→ DGKA→ RPL41→ ←CS ←MIP ←BAZ2A ←SDR9C7 LRP1→ INHBC→
OR2AP1→ ORMDL2→ IKZF4→ ←RNF41 ←TIMELESS ←PTGES3 ←RDH16 MIR1228→
OR10P1→ ←MMP19 RPS26→ NABP2→ SPRYD4→ ←NACA ←ZBTB39 SHMT2→
METTL7B→ ←WIBG ERBB3→ ←CNPY2 ←ATP5B ←TAC3 ←NDUFA4L2
←ITGA7 ←PMEL ESYT1→ ←PAN2 ←SNORD59B ←MYO1A ←STAC3
BLOC1S1→ RAB5B→ MYL6→ ←STAT2 ←SNORD59A ←TMEM194A INHBE→
BLOC1S1−RDH5→ SUOX→ SLC39A5→ ←GLS2 ←PRIM1 NAB2→ ←R3HDM2
RDH5→ PA2G4→ ←APOF ←STAT6
←CD63 ZC3H10→
56
56.5
57
57.5
Position on chr12 (Mb)

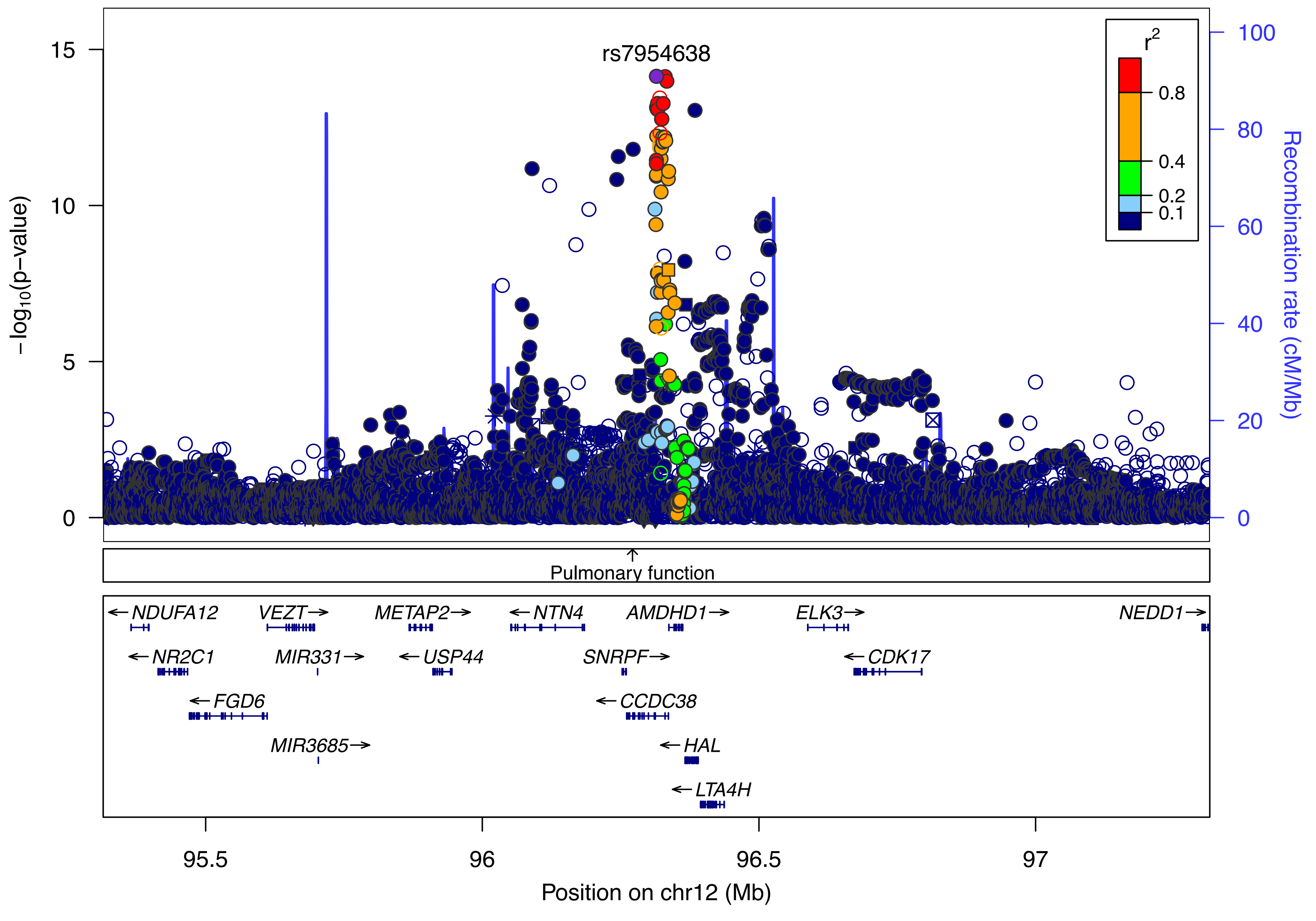
rs7954638
−log10(p−value)
Recombination rate (cM/Mb)
r2
0.8
0.4
0.2
0.1
Pulmonary function
NDUFA12
NR2C1
FGD6
VEZT
MIR331
MIR3685
METAP2
USP44
NTN4
SNRPF
CCDC38
AMDHD1
HAL
LTA4H
ELK3
CDK17
NEDD1
95.5
96
96.5
97
Position on chr12 (Mb)

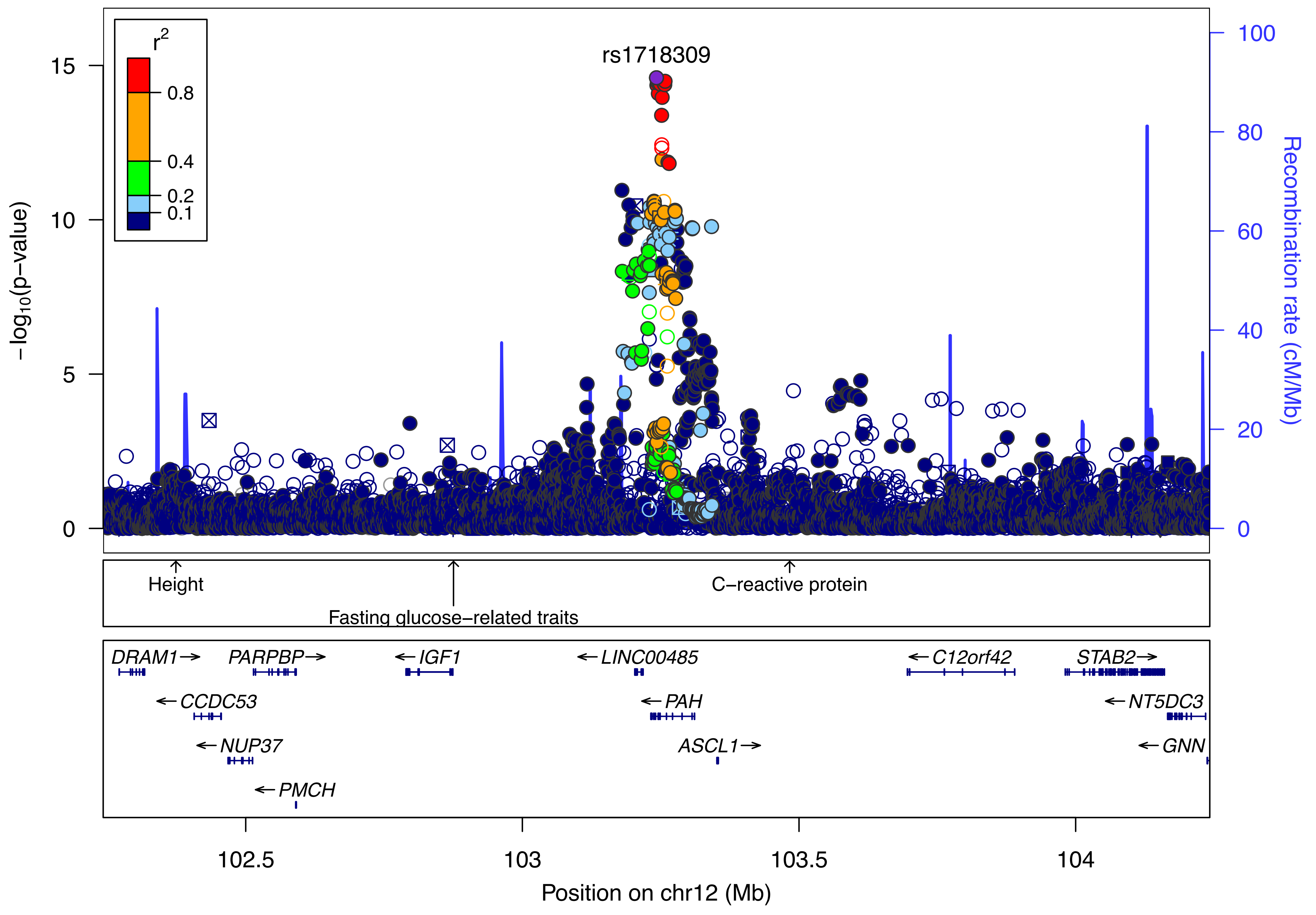
rs1718309
r2
0.8
0.4
0.2
0.1
−log10(p−value)
Recombination rate (cM/Mb)
Height
Fasting glucose−related traits
C−reactive protein
DRAM1→
PARPBP→
←IGF1
←LINC00485
←C12orf42
STAB2→
←CCDC53
←PAH
←NT5DC3
←NUP37
ASCL1→
←GNN
←PMCH
102.5
103
103.5
104
Position on chr12 (Mb)

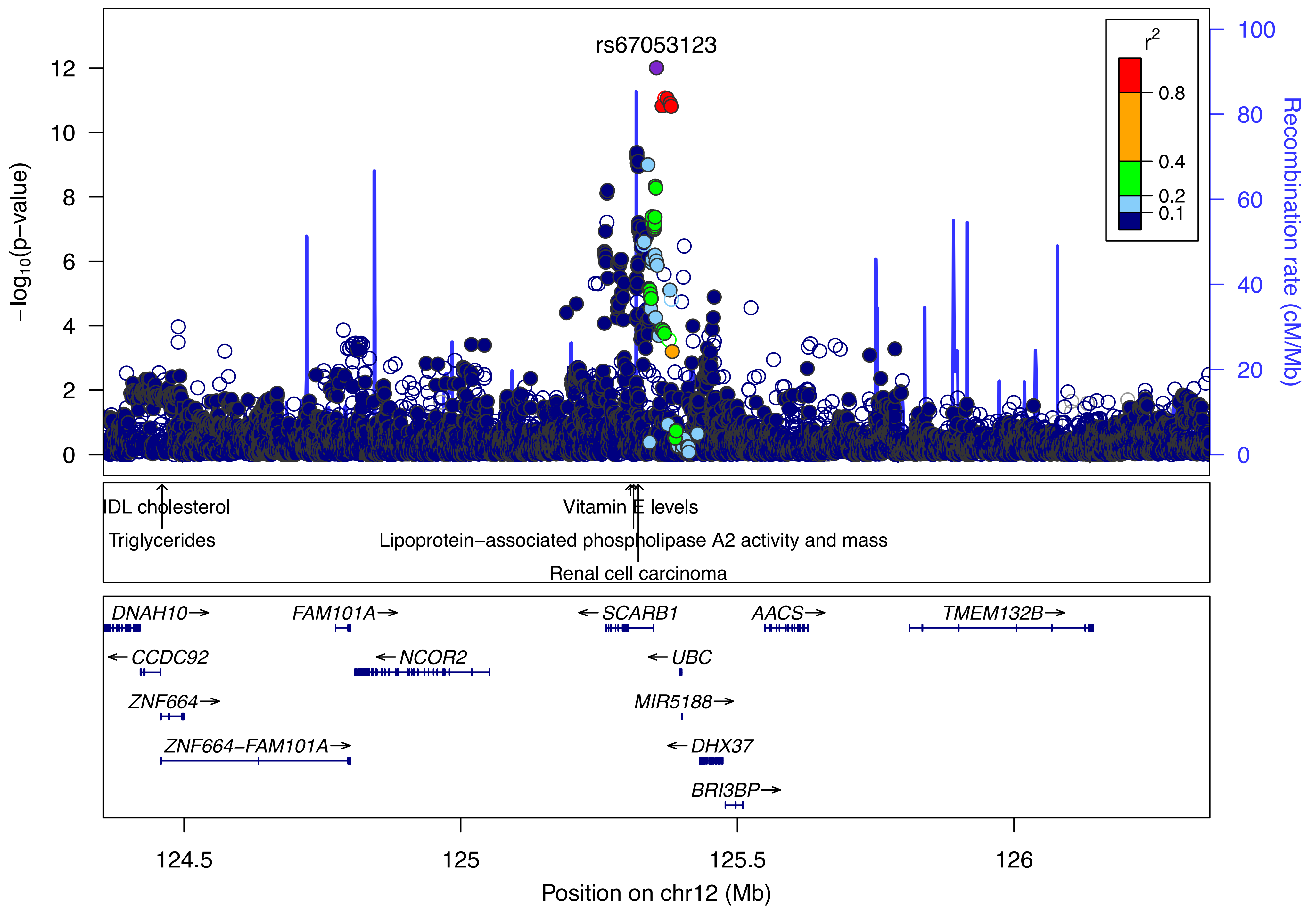
rs67053123
r²
0.8
0.4
0.2
0.1
−log₁₀(p−value)
Recombination rate (cM/Mb)
IDL cholesterol
Triglycerides
Vitamin E levels
Lipoprotein−associated phospholipase A2 activity and mass
Renal cell carcinoma
DNAH10→
FAM101A→
←SCARB1
AACS→
TMEM132B→
←CCDC92
←NCOR2
←UBC
ZNF664→
MIR5188→
ZNF664−FAM101A→
←DHX37
BRI3BP→
124.5
125
125.5
126
Position on chr12 (Mb)

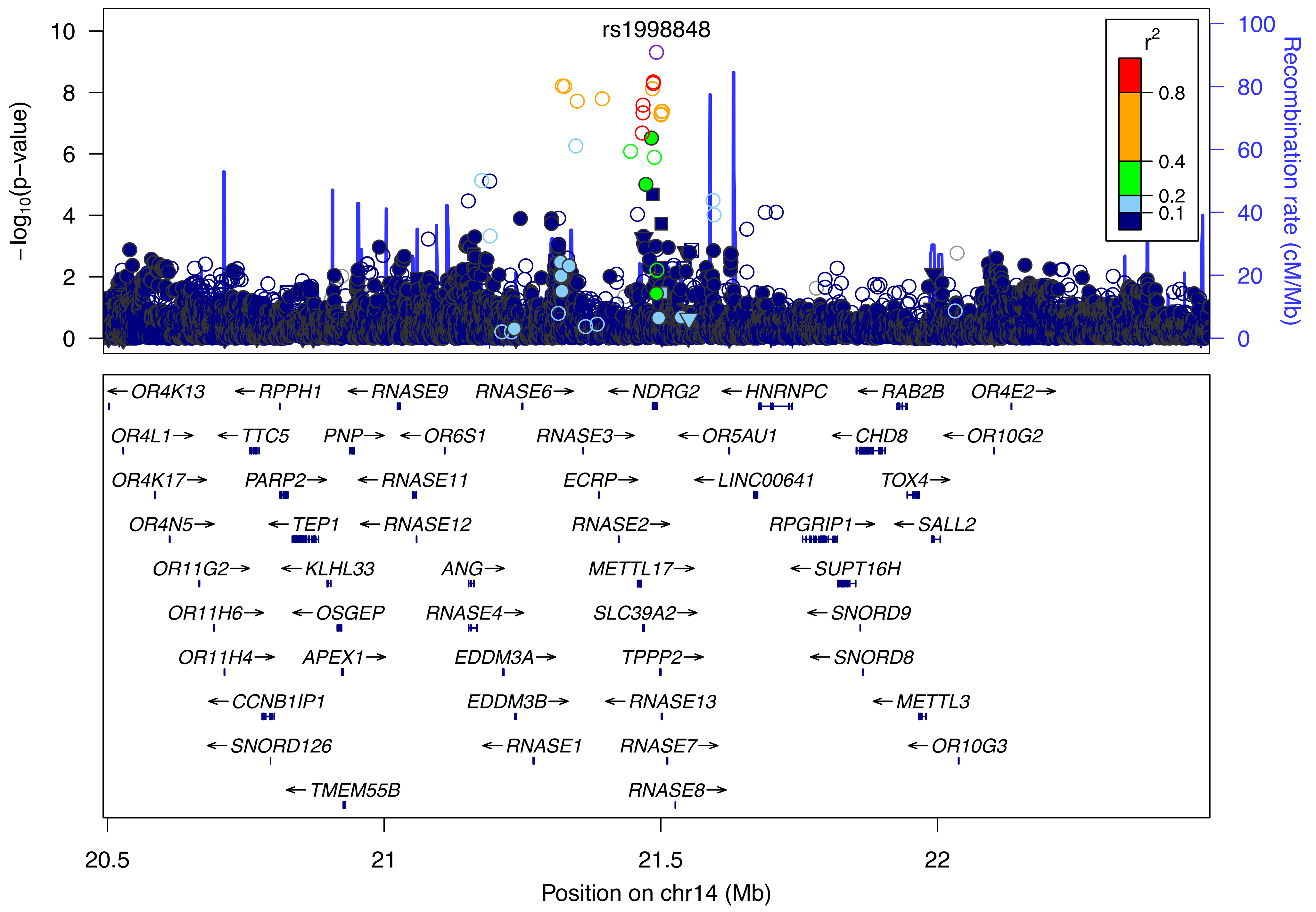
rs1998848
−log10(p−value)
Recombination rate (cM/Mb)
r2
0.8
0.4
0.2
0.1
←OR4K13
←RPPH1
←RNASE9
RNASE6→
←NDRG2
←HNRNPC
←RAB2B
OR4E2→
OR4L1→
←TTC5
PNP→
←OR6S1
RNASE3→
←OR5AU1
←CHD8
←OR10G2
OR4K17→
PARP2→
←RNASE11
ECRP→
←LINC00641
TOX4→
OR4N5→
←TEP1
←RNASE12
RNASE2→
RPGRIP1→
←SALL2
OR11G2→
←KLHL33
ANG→
METTL17→
←SUPT16H
OR11H6→
←OSGEP
RNASE4→
SLC39A2→
←SNORD9
OR11H4→
APEX1→
EDDM3A→
TPPP2→
←SNORD8
←CCNB1IP1
EDDM3B→
←RNASE13
←METTL3
←SNORD126
←RNASE1
RNASE7→
←OR10G3
←TMEM55B
RNASE8→
20.5
21
21.5
22
Position on chr14 (Mb)

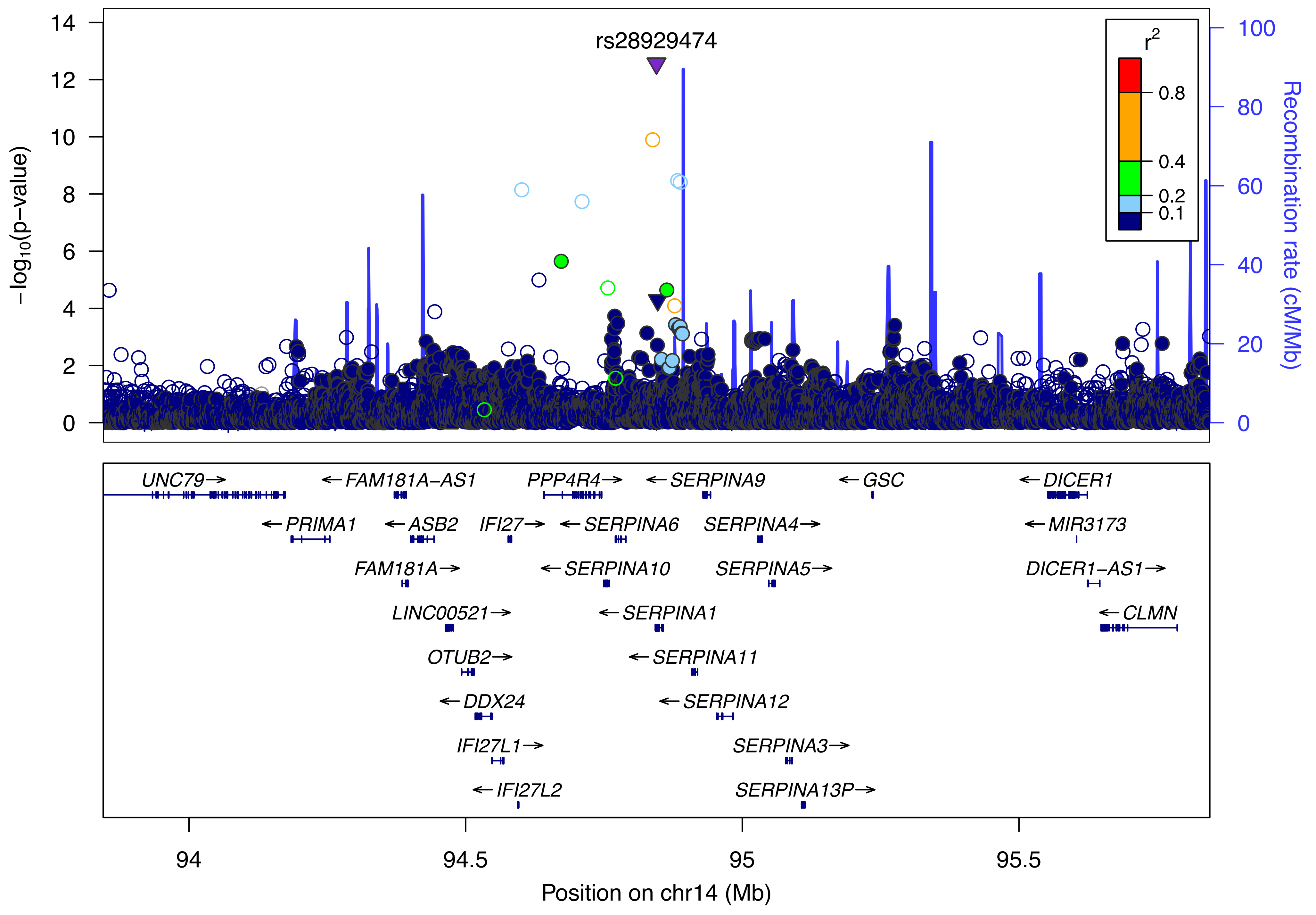
rs28929474
r^2
0.8
0.4
0.2
0.1
−log10(p−value)
Recombination rate (cM/Mb)
UNC79→
←FAM181A−AS1
PPP4R4→
←SERPINA9
←GSC
←DICER1
←PRIMA1
←ASB2
IFI27→
←SERPINA6
SERPINA4→
←MIR3173
FAM181A→
←SERPINA10
SERPINA5→
DICER1−AS1→
LINC00521→
←SERPINA1
←CLMN
OTUB2→
←SERPINA11
←DDX24
←SERPINA12
IFI27L1→
SERPINA3→
←IFI27L2
SERPINA13P→
Position on chr14 (Mb)

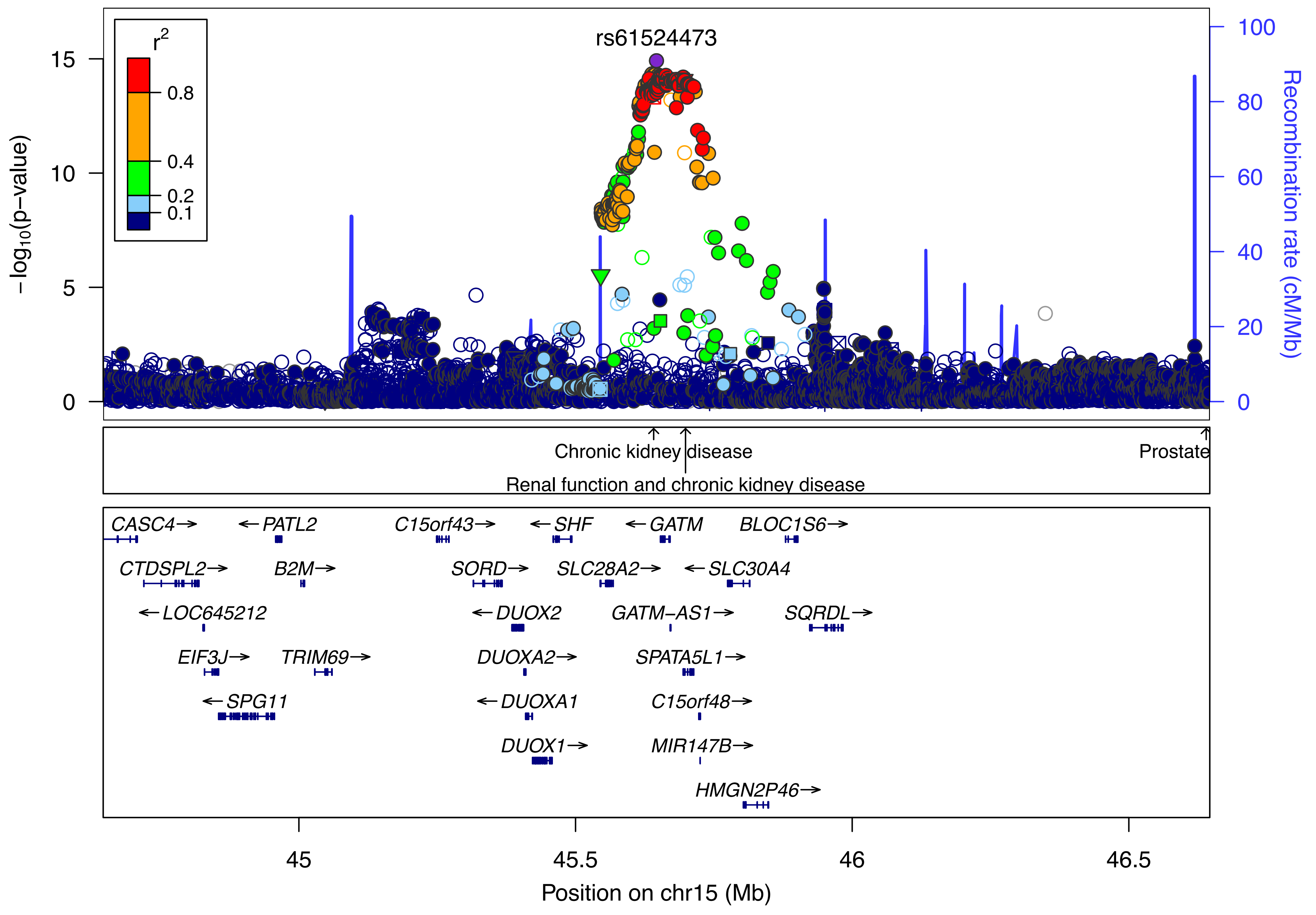

rs61524473
r2
0.8
0.4
0.2
0.1
−log10(p−value)
Recombination rate (cM/Mb)
Chronic kidney disease
Renal function and chronic kidney disease
Prostate
CASC4→
←PATL2
C15orf43→
←SHF
←GATM
BLOC1S6→
CTDSPL2→
B2M→
SORD→
SLC28A2→
←SLC30A4
←LOC645212
←DUOX2
GATM−AS1→
SQRDL→
EIF3J→
TRIM69→
DUOXA2→
SPATA5L1→
←SPG11
←DUOXA1
C15orf48→
DUOX1→
MIR147B→
HMGN2P46→
Position on chr15 (Mb)

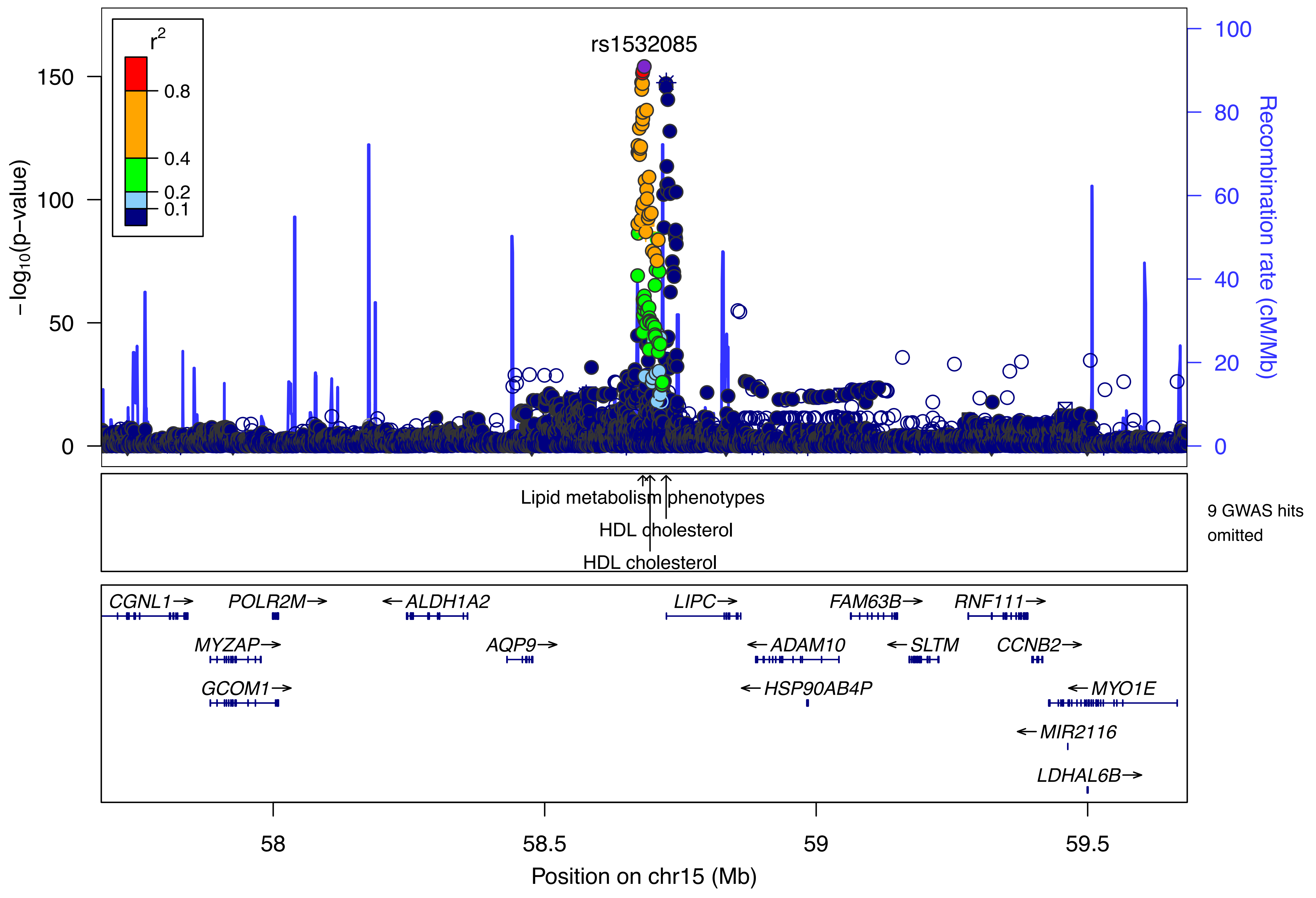

rs1532085
r²
0.8
0.4
0.2
0.1
−log10(p−value)
150
100
50
0
Recombination rate (cM/Mb)
100
80
60
40
20
0
Lipid metabolism phenotypes
HDL cholesterol
HDL cholesterol
9 GWAS hits omitted
CGNL1→
POLR2M→
←ALDH1A2
LIPC→
FAM63B→
RNF111→
MYZAP→
AQP9→
←ADAM10
←SLTM
CCNB2→
GCOM1→
←HSP90AB4P
←MYO1E
←MIR2116
LDHAL6B→
58
58.5
59
59.5
Position on chr15 (Mb)

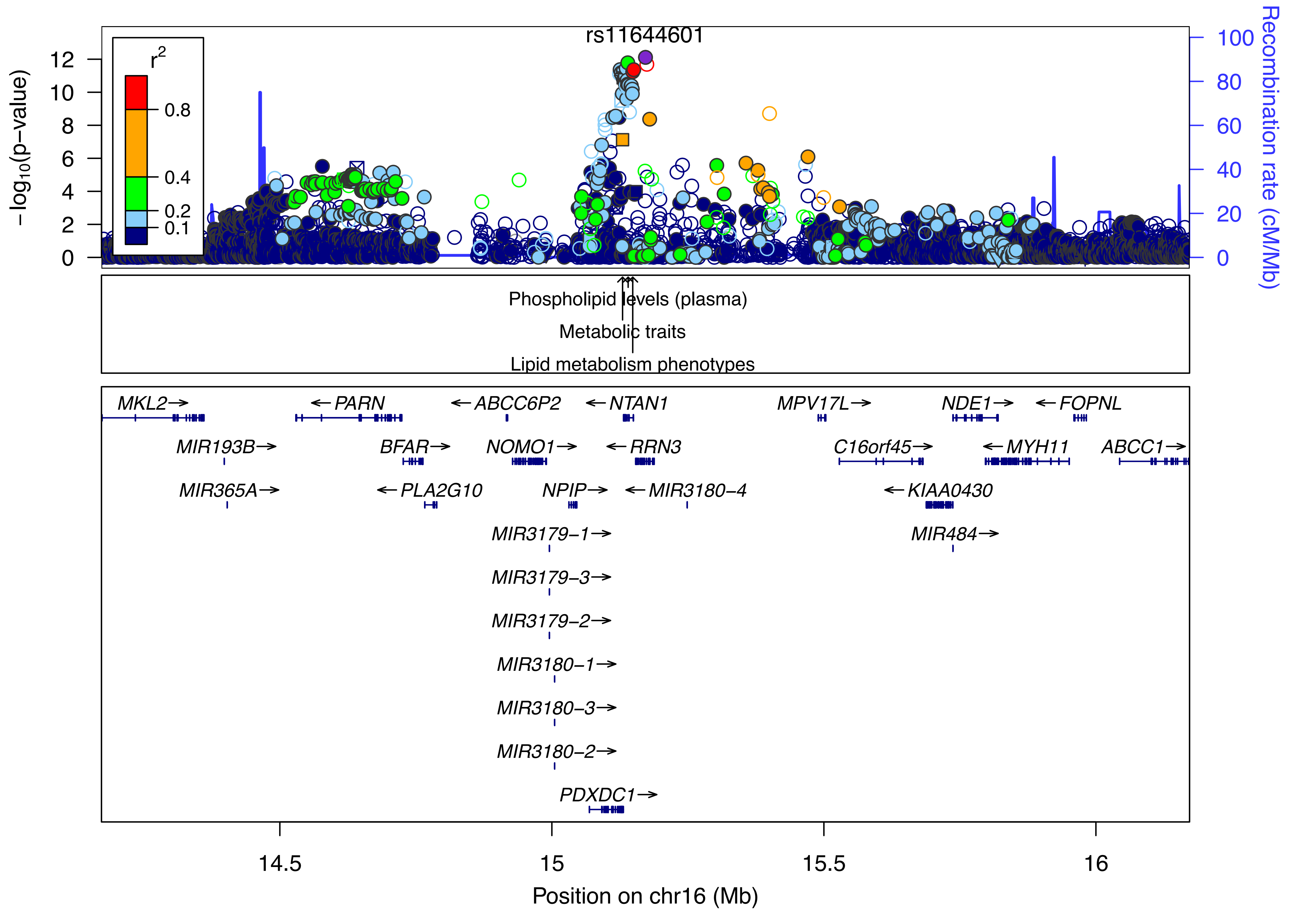

rs11644601
−log10(p−value)
Recombination rate (cM/Mb)
r2
0.8
0.4
0.2
0.1
Phospholipid levels (plasma)
Metabolic traits
Lipid metabolism phenotypes
MKL2→
←PARN
←ABCC6P2
←NTAN1
MPV17L→
NDE1→
←FOPNL
MIR193B→
BFAR→
NOMO1→
←RRN3
C16orf45→
←MYH11
ABCC1→
MIR365A→
←PLA2G10
NPIP→
←MIR3180−4
←KIAA0430
MIR3179−1→
MIR484→
MIR3179−3→
MIR3179−2→
MIR3180−1→
MIR3180−3→
MIR3180−2→
PDXDC1→
14.5
15
15.5
16
Position on chr16 (Mb)

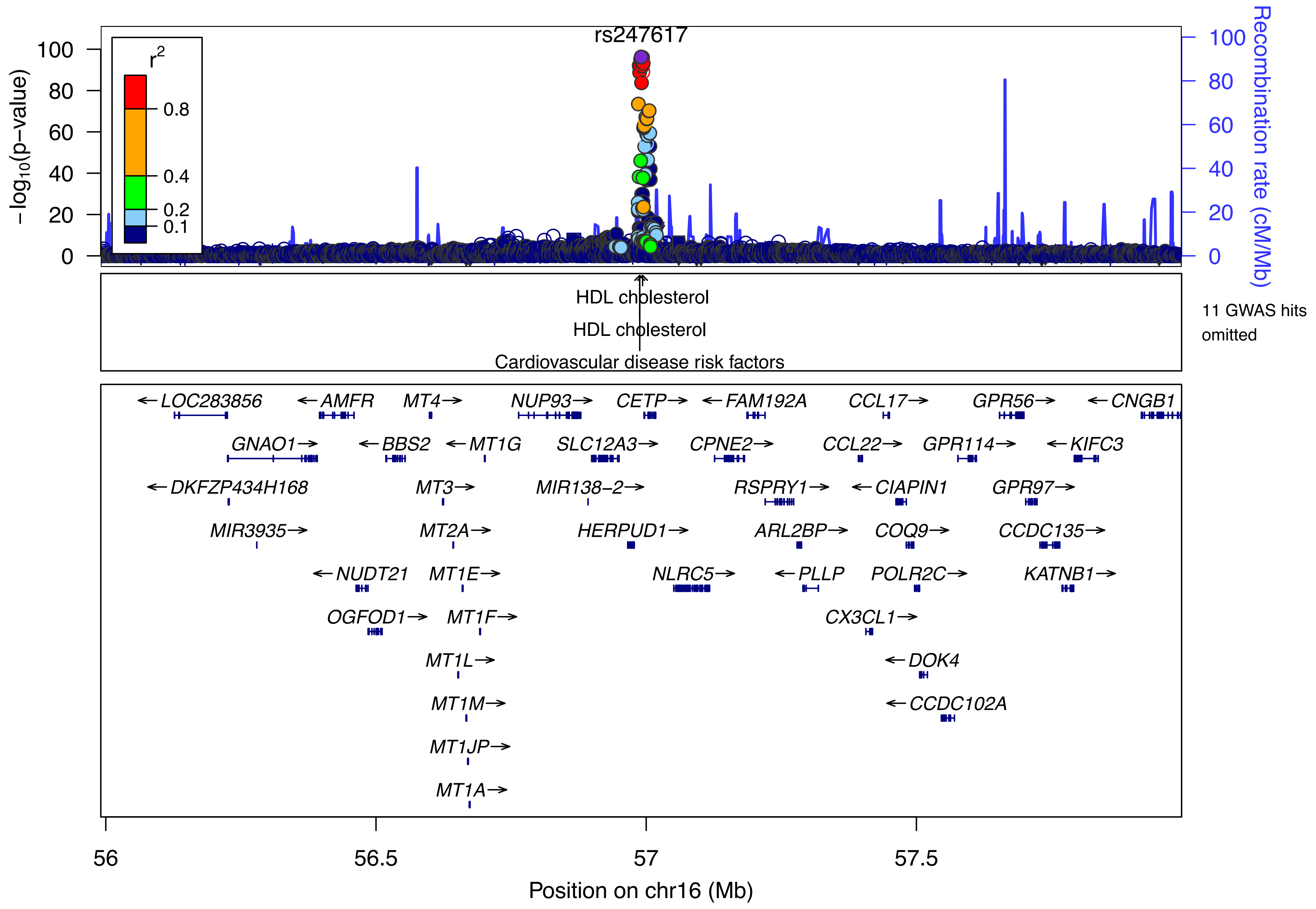
rs247617
−log10(p−value)
100
80
60
40
20
0
r2
0.8
0.4
0.2
0.1
Recombination rate (cM/Mb)
HDL cholesterol
HDL cholesterol
Cardiovascular disease risk factors
11 GWAS hits omitted
LOC283856
AMFR
MT4
NUP93
CETP
FAM192A
CCL17
GPR56
CNGB1
GNAO1
BBS2
MT1G
SLC12A3
CPNE2
CCL22
GPR114
KIFC3
DKFZP434H168
MT3
MIR138−2
RSPRY1
CIAPIN1
GPR97
MIR3935
MT2A
HERPUD1
ARL2BP
COQ9
CCDC135
NUDT21
MT1E
NLRC5
PLLP
POLR2C
KATNB1
OGFOD1
MT1F
CX3CL1
MT1L
DOK4
MT1M
CCDC102A
MT1JP
MT1A
56
56.5
57
57.5
Position on chr16 (Mb)

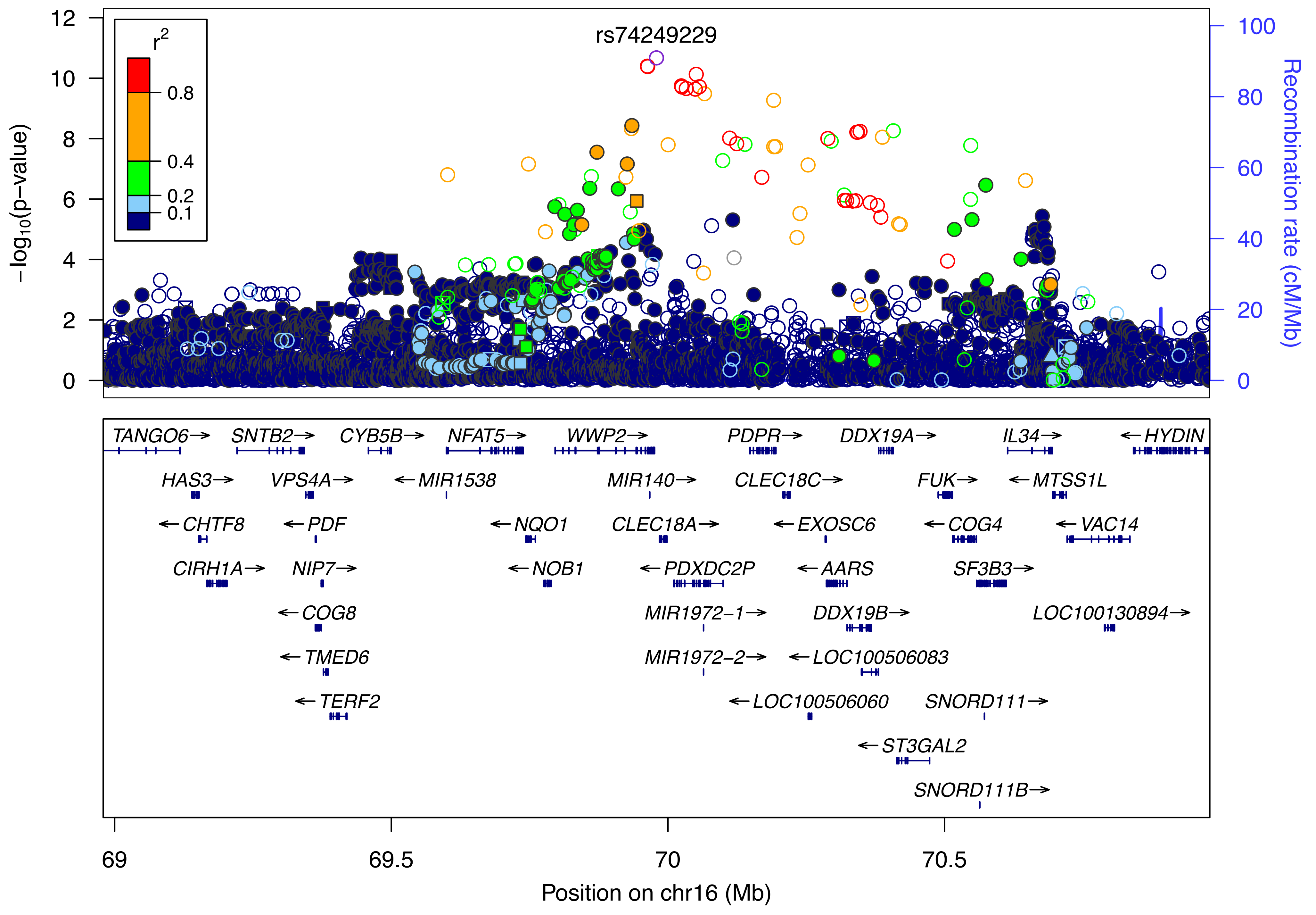
rs74249229
r2
0.8
0.4
0.2
0.1
−log10(p−value)
Recombination rate (cM/Mb)
TANGO6→
SNTB2→
CYB5B→
NFAT5→
WWP2→
PDPR→
DDX19A→
IL34→
←HYDIN
HAS3→
VPS4A→
←MIR1538
MIR140→
CLEC18C→
FUK→
←MTSS1L
←CHTF8
←PDF
←NQO1
CLEC18A→
←EXOSC6
←COG4
←VAC14
CIRH1A→
NIP7→
←NOB1
←PDXDC2P
←AARS
SF3B3→
←COG8
MIR1972−1→
DDX19B→
LOC100130894→
←TMED6
MIR1972−2→
←LOC100506083
←TERF2
←LOC100506060
SNORD111→
←ST3GAL2
SNORD111B→
69
69.5
70
70.5
Position on chr16 (Mb)

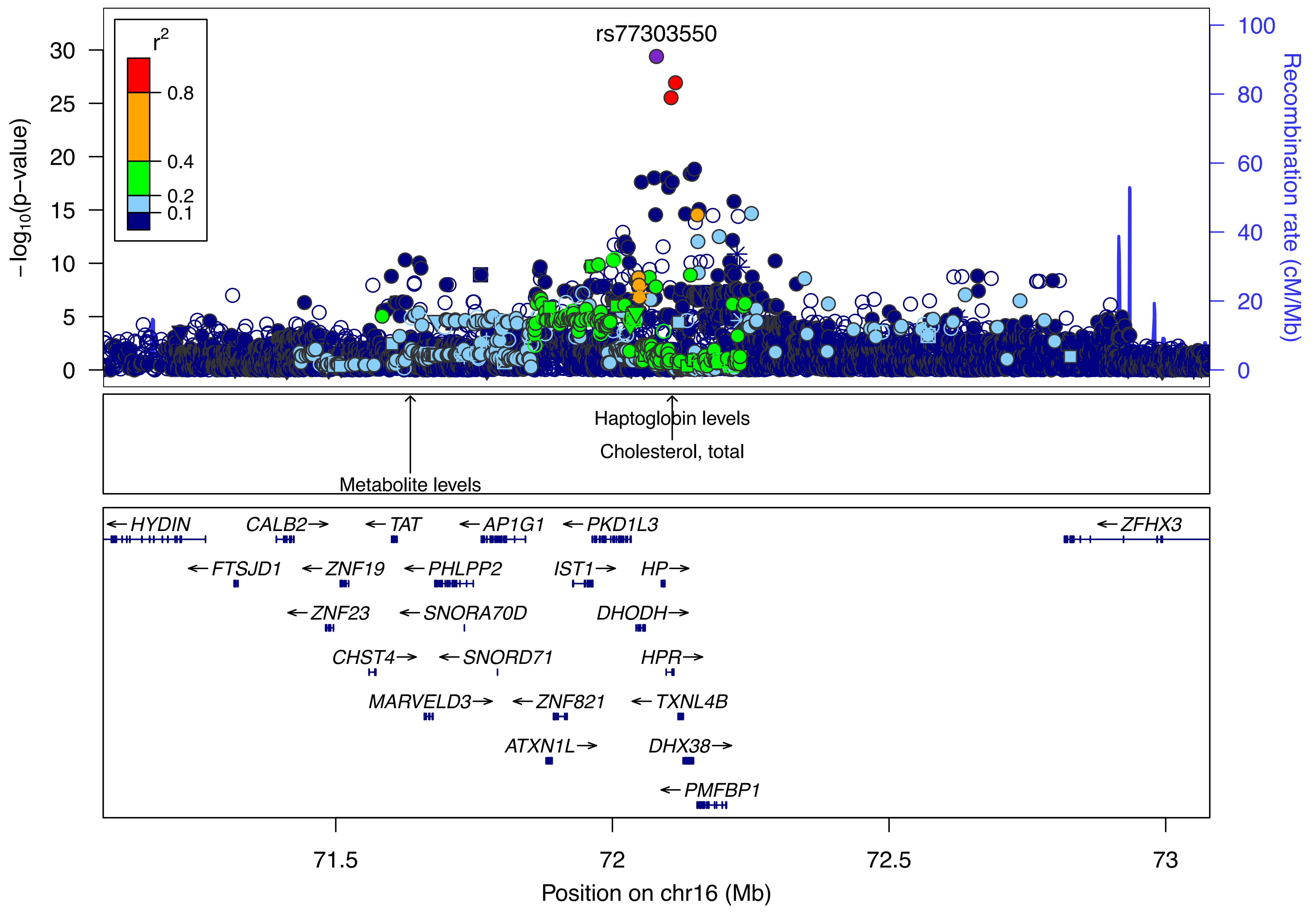

rs77303550
r^2
0.8
0.4
0.2
0.1
−log$_{10}$(p−value)
0
5
10
15
20
25
30
Recombination rate (cM/Mb)
0
20
40
60
80
100
Metabolite levels
Haptoglobin levels
Cholesterol, total
← HYDIN
CALB2 →
← TAT
← AP1G1
← PKD1L3
← ZFHX3
← FTSJD1
← ZNF19
← PHLPP2
IST1 →
HP →
← ZNF23
← SNORA70D
DHODH →
CHST4 →
← SNORD71
HPR →
MARVELD3 →
← ZNF821
← TXNL4B
ATXN1L →
DHX38 →
← PMFBP1
71.5
72
72.5
73
Position on chr16 (Mb)

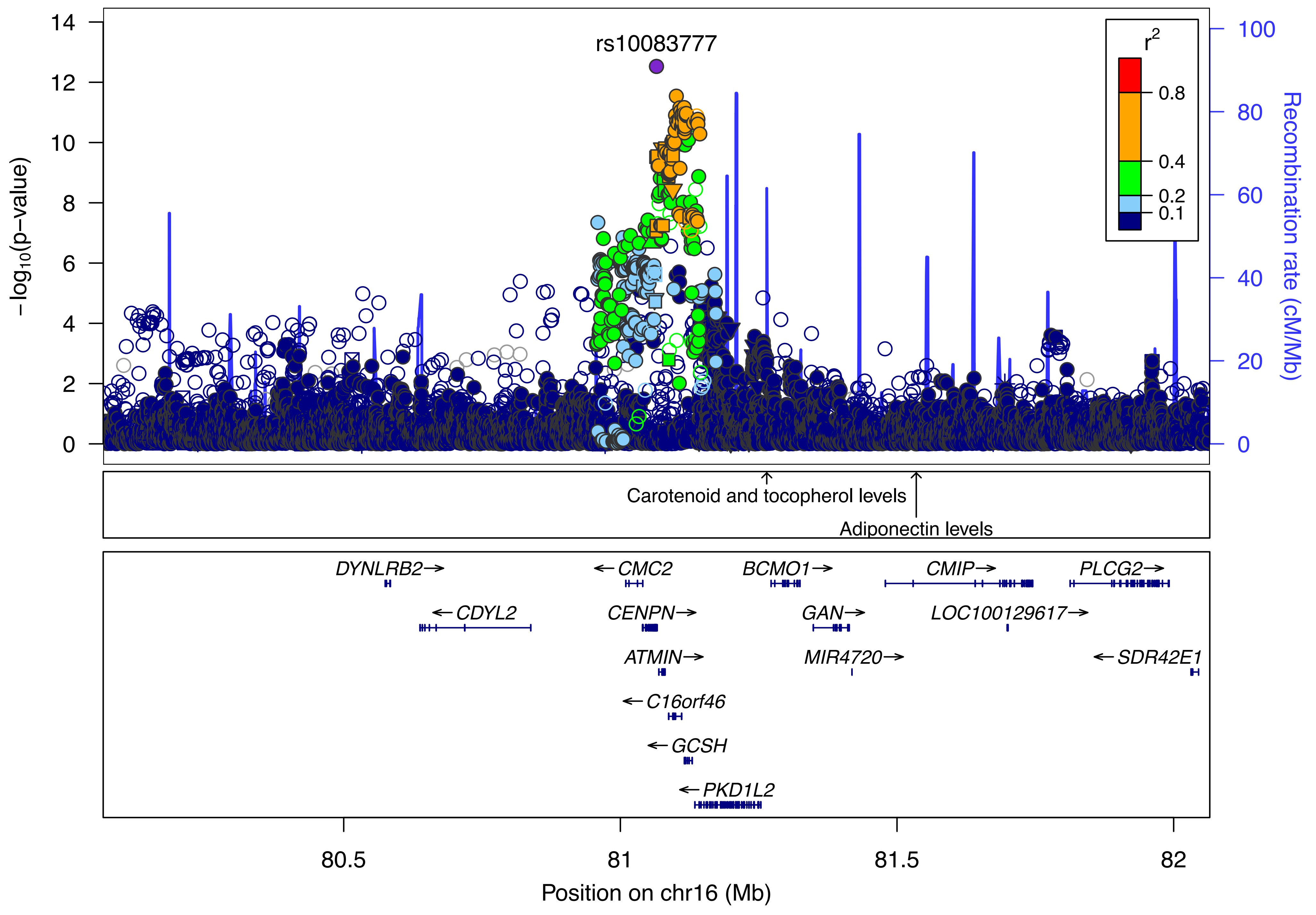

rs10083777
r^2
0.8
0.4
0.2
0.1
-log10(p-value)
Recombination rate (cM/Mb)
Carotenoid and tocopherol levels
Adiponectin levels
DYNLRB2→
←CDYL2
←CMC2
CENPN→
ATMIN→
←C16orf46
←GCSH
←PKD1L2
BCMO1→
GAN→
MIR4720→
CMIP→
LOC100129617→
PLCG2→
←SDR42E1
80.5
81
81.5
82
Position on chr16 (Mb)

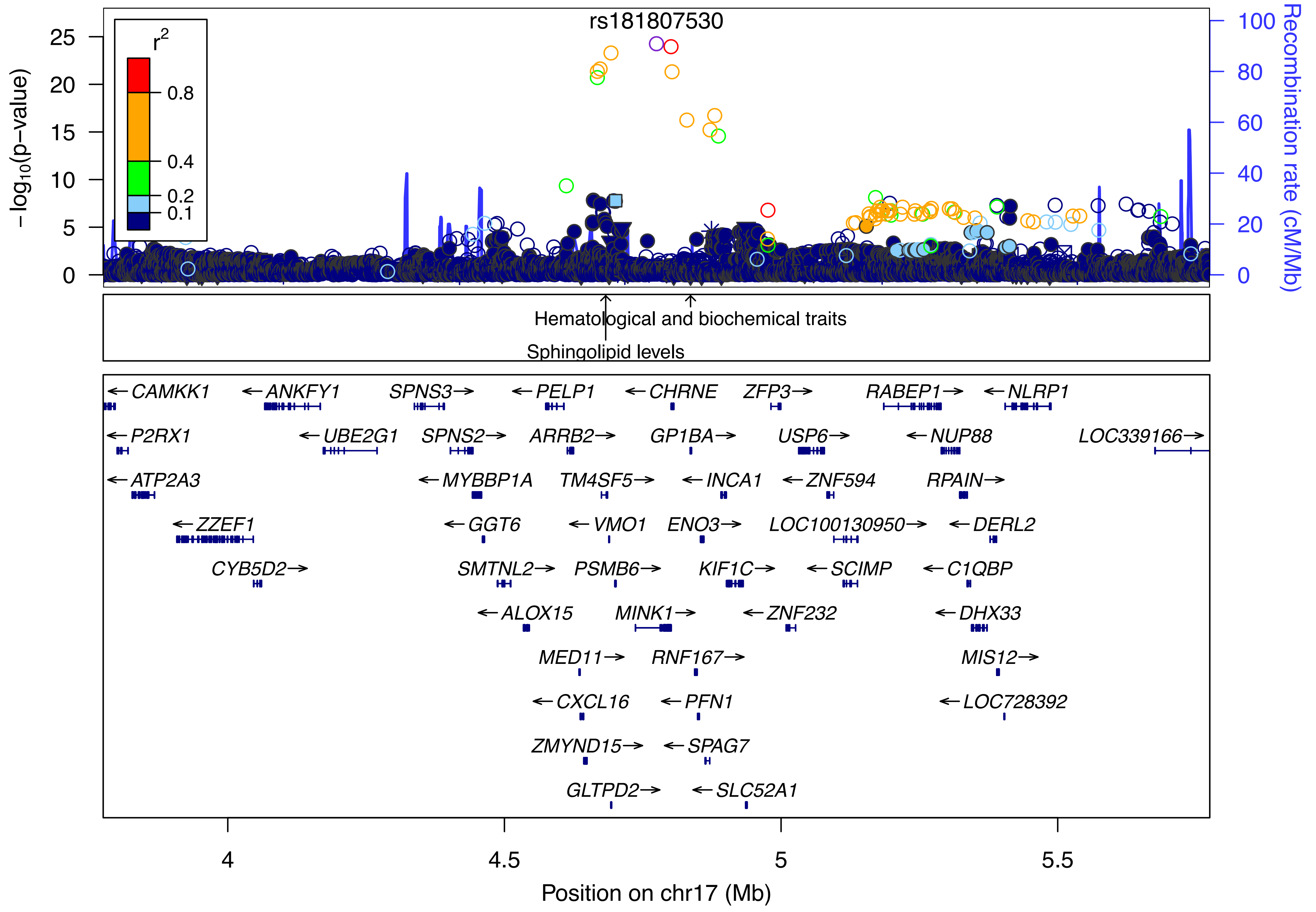

rs181807530
r^2
0.8
0.4
0.2
0.1
−log10(p−value)
0
5
10
15
20
25
Recombination rate (cM/Mb)
0
20
40
60
80
100
Hematological and biochemical traits
Sphingolipid levels
←CAMKK1
←ANKFY1
SPNS3→
←PELP1
←CHRNE
ZFP3→
RABEP1→
←NLRP1
←P2RX1
←UBE2G1
SPNS2→
ARRB2→
GP1BA→
USP6→
←NUP88
LOC339166→
←ATP2A3
←MYBBP1A
TM4SF5→
←INCA1
←ZNF594
RPAIN→
←ZZEF1
←GGT6
←VMO1
ENO3→
LOC100130950→
←DERL2
CYB5D2→
SMTNL2→
PSMB6→
KIF1C→
←SCIMP
←C1QBP
←ALOX15
MINK1→
←ZNF232
←DHX33
MED11→
RNF167→
MIS12→
←CXCL16
←PFN1
←LOC728392
ZMYND15→
←SPAG7
GLTPD2→
←SLC52A1
4
4.5
5
5.5
Position on chr17 (Mb)

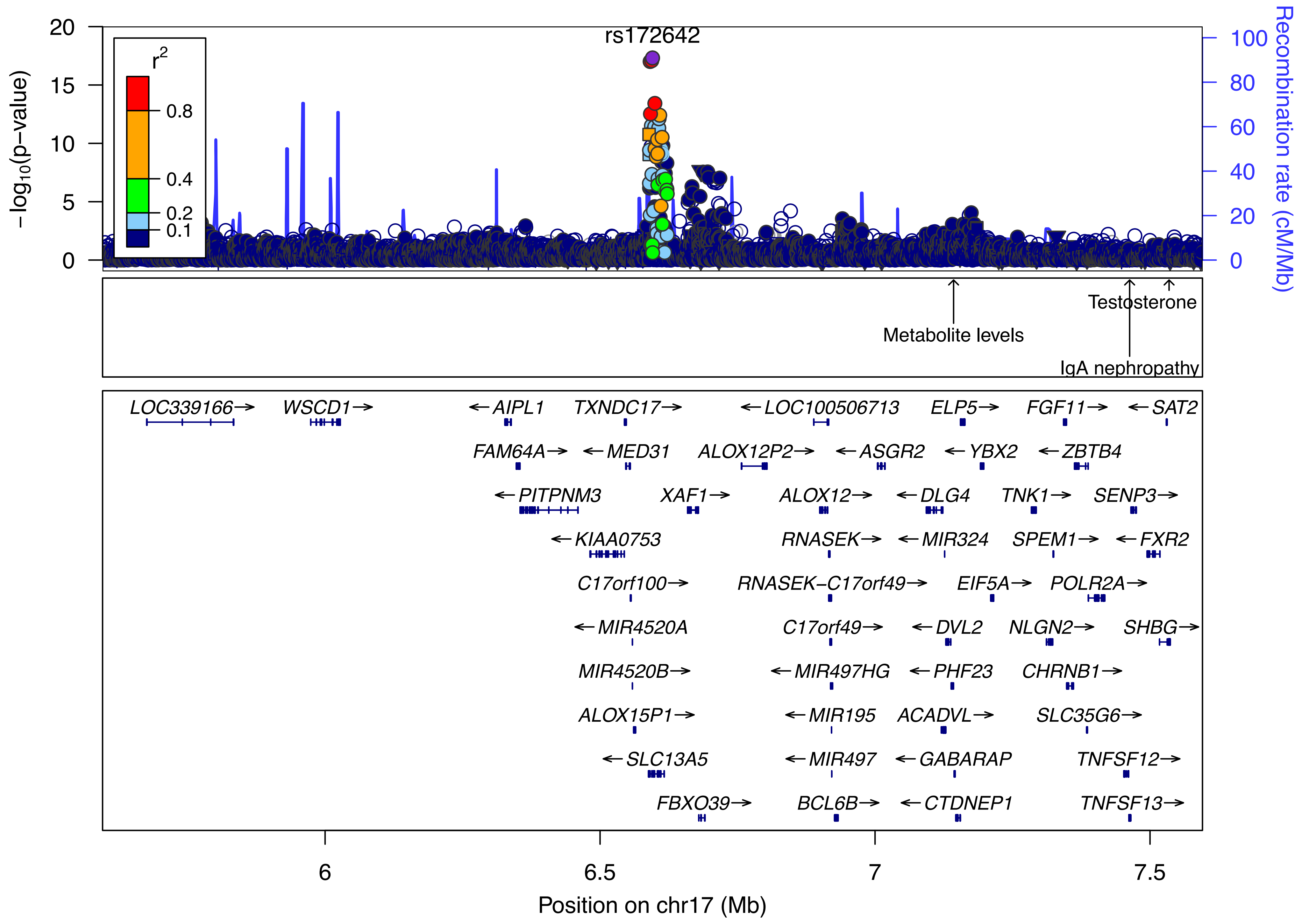
rs172642
−log10(p−value)
Recombination rate (cM/Mb)
r2
0.8
0.4
0.2
0.1
Metabolite levels
Testosterone
IgA nephropathy
LOC339166→
WSCD1→
←AIPL1
TXNDC17→
←LOC100506713
ELP5→
FGF11→
←SAT2
FAM64A→
←MED31
ALOX12P2→
←ASGR2
←YBX2
←ZBTB4
←PITPNM3
XAF1→
ALOX12→
←DLG4
TNK1→
SENP3→
←KIAA0753
RNASEK→
←MIR324
SPEM1→
←FXR2
C17orf100→
RNASEK−C17orf49→
EIF5A→
POLR2A→
←MIR4520A
C17orf49→
←DVL2
NLGN2→
SHBG→
MIR4520B→
←MIR497HG
←PHF23
CHRNB1→
ALOX15P1→
←MIR195
ACADVL→
SLC35G6→
←SLC13A5
←MIR497
←GABARAP
TNFSF12→
FBXO39→
BCL6B→
←CTDNEP1
TNFSF13→
Position on chr17 (Mb)

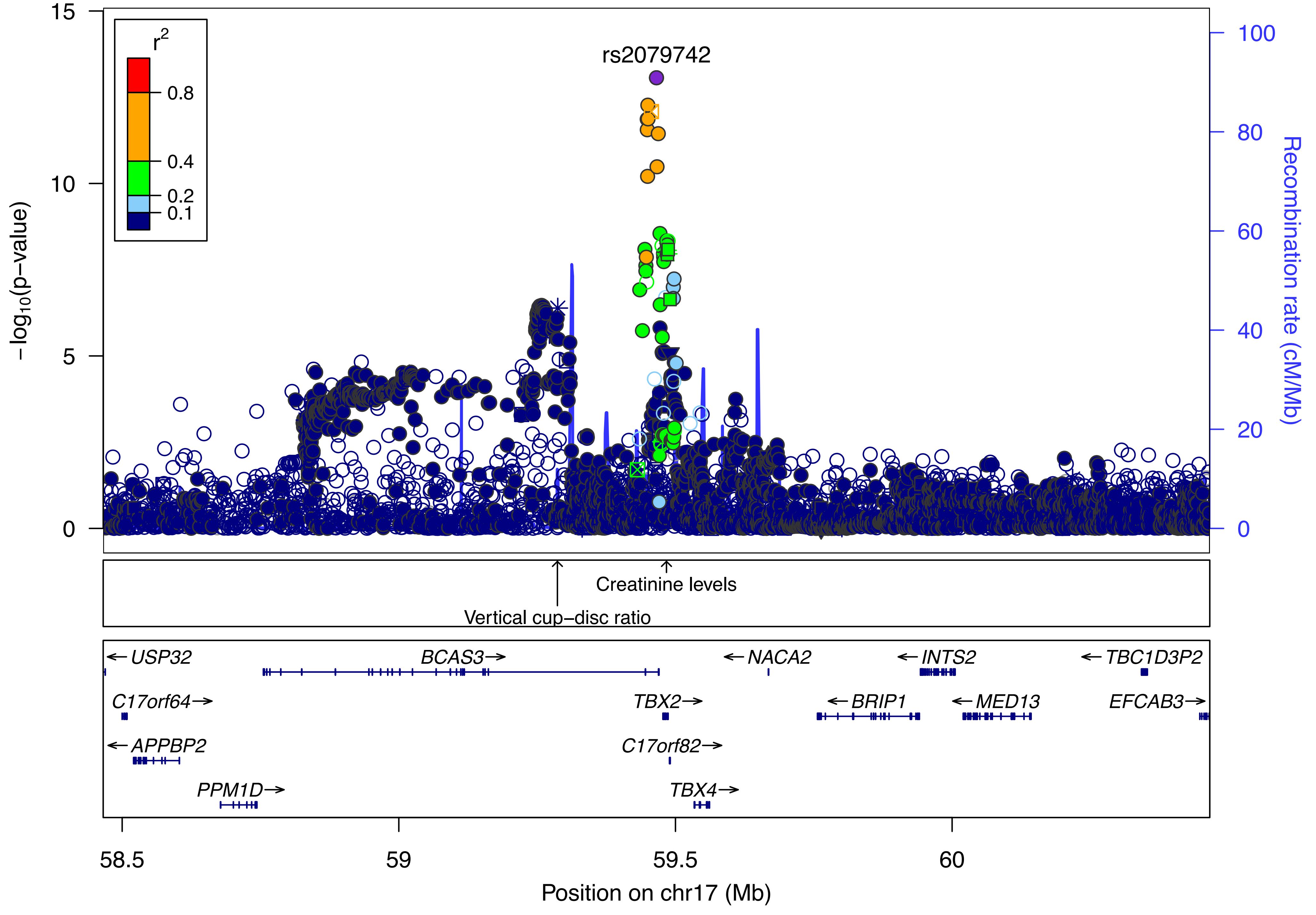

rs2079742
r^2
0.8
0.4
0.2
0.1
$-\log_{10}$(p−value)
Recombination rate (cM/Mb)
Creatinine levels
Vertical cup−disc ratio
←USP32
BCAS3→
←NACA2
←INTS2
←TBC1D3P2
C17orf64→
TBX2→
←BRIP1
←MED13
EFCAB3→
←APPBP2
C17orf82→
PPM1D→
TBX4→
58.5
59
59.5
60
Position on chr17 (Mb)

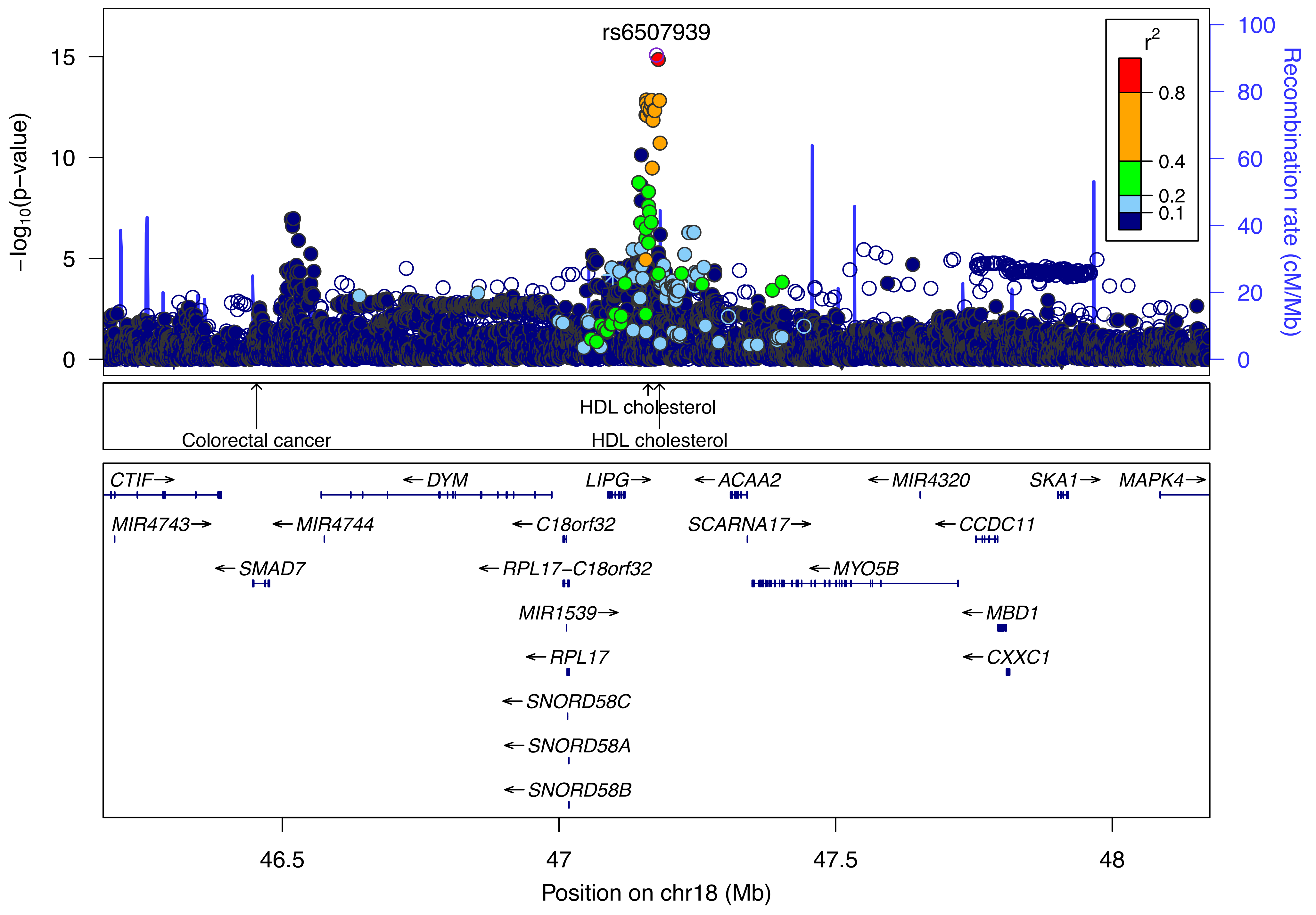

rs6507939
−log10(p−value)
Recombination rate (cM/Mb)
r2
0.8
0.4
0.2
0.1
Colorectal cancer
HDL cholesterol
HDL cholesterol
CTIF→
MIR4743→
←SMAD7
←MIR4744
←DYM
←C18orf32
←RPL17−C18orf32
MIR1539→
←RPL17
←SNORD58C
←SNORD58A
←SNORD58B
LIPG→
←ACAA2
SCARNA17→
←MYO5B
←MIR4320
←CCDC11
←MBD1
←CXXC1
SKA1→
MAPK4→
46.5
47
47.5
48
Position on chr18 (Mb)

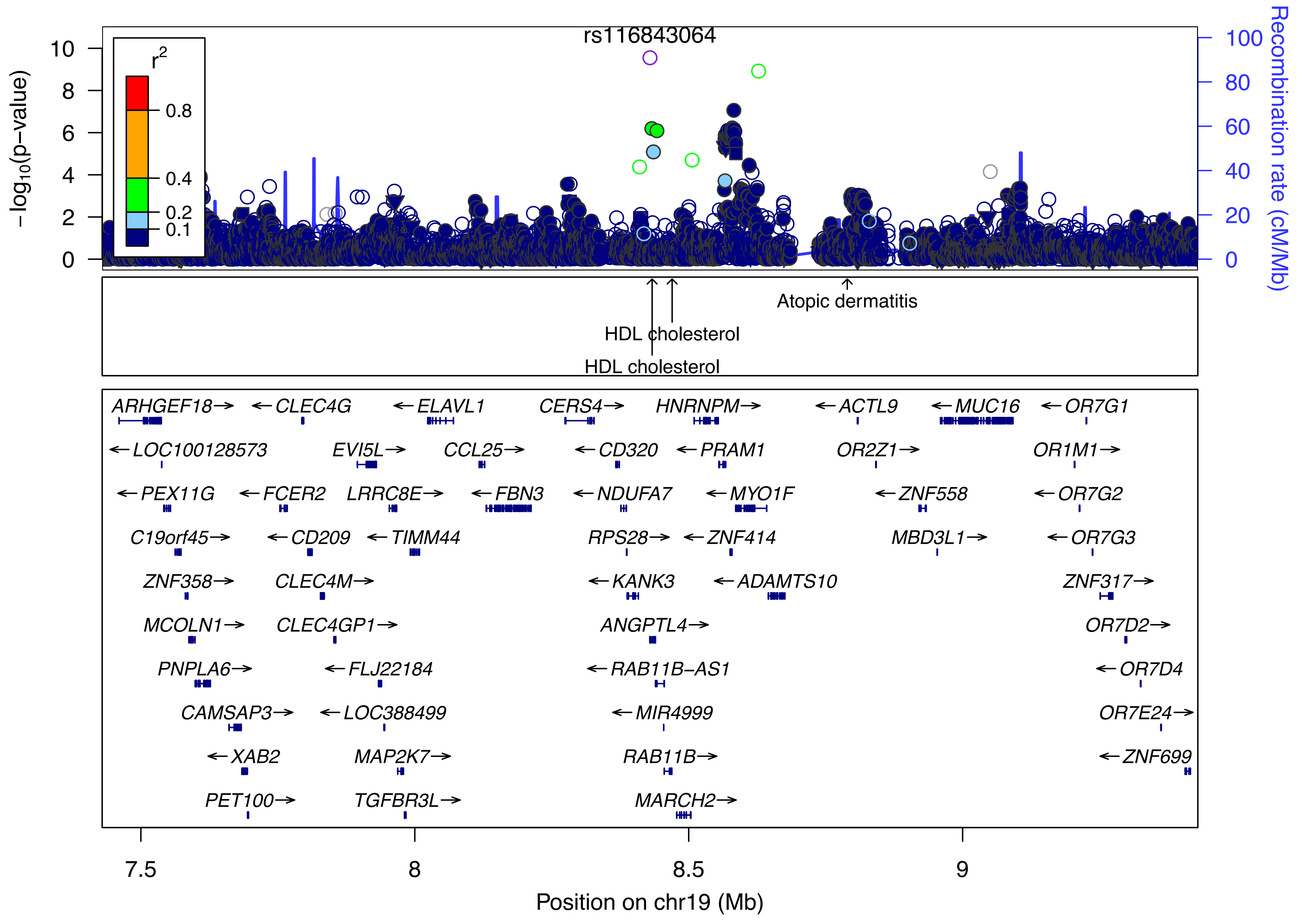

rs116843064
−log10(p−value)
10
8
6
4
2
0
r2
0.8
0.4
0.2
0.1
Recombination rate (cM/Mb)
100
80
60
40
20
0
Atopic dermatitis
HDL cholesterol
HDL cholesterol
ARHGEF18→
←CLEC4G
←ELAVL1
CERS4→
HNRNPM→
←ACTL9
←MUC16
←OR7G1
←LOC100128573
EVI5L→
CCL25→
←CD320
←PRAM1
OR2Z1→
OR1M1→
←PEX11G
←FCER2
LRRC8E→
←FBN3
←NDUFA7
←MYO1F
←ZNF558
←OR7G2
C19orf45→
←CD209
←TIMM44
RPS28→
←ZNF414
MBD3L1→
←OR7G3
ZNF358→
CLEC4M→
←KANK3
←ADAMTS10
ZNF317→
MCOLN1→
CLEC4GP1→
ANGPTL4→
OR7D2→
PNPLA6→
←FLJ22184
←RAB11B−AS1
←OR7D4
CAMSAP3→
←LOC388499
←MIR4999
OR7E24→
←XAB2
MAP2K7→
RAB11B→
←ZNF699
PET100→
TGFBR3L→
MARCH2→
7.5
8
8.5
9
Position on chr19 (Mb)

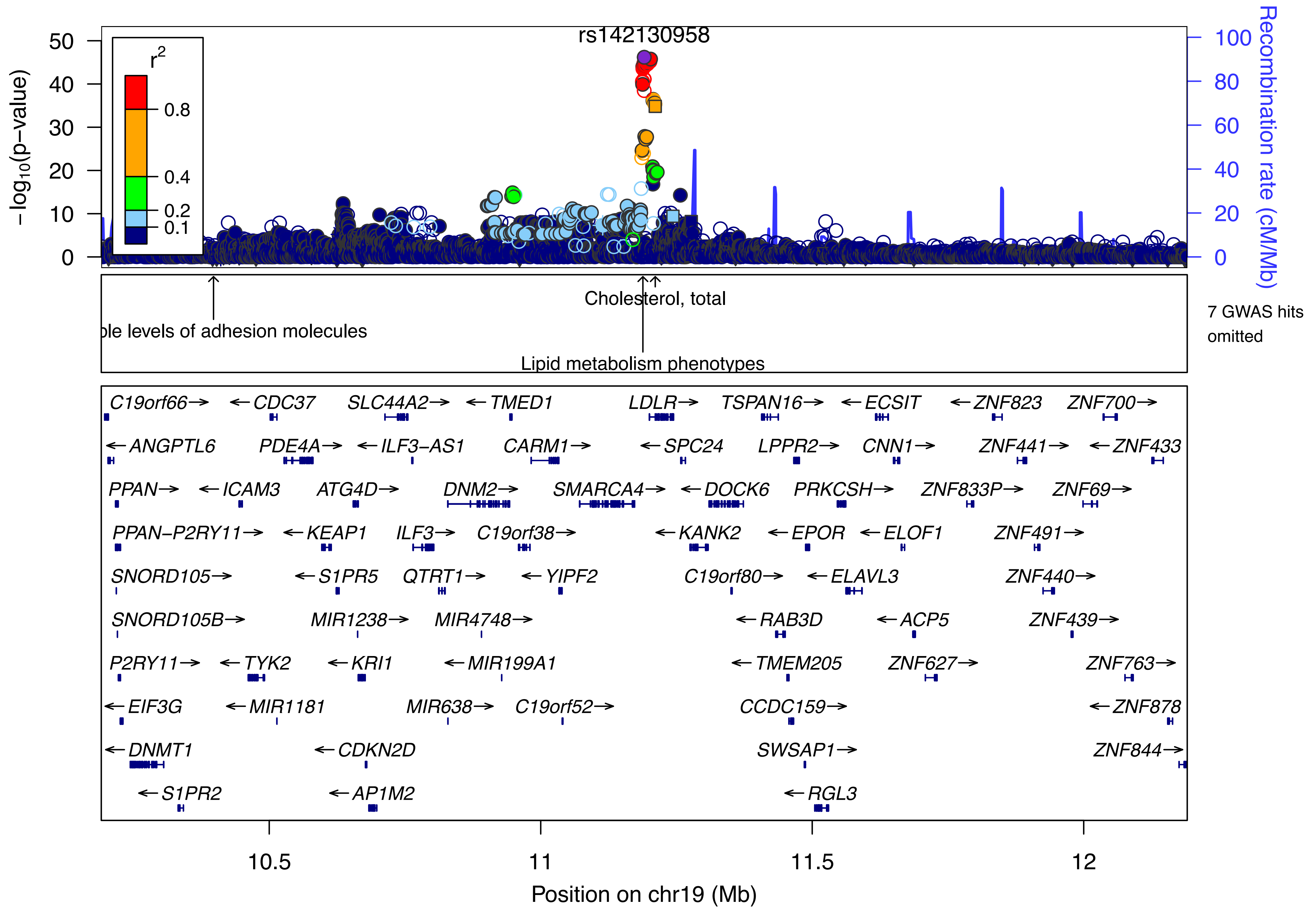

rs142130958
r2
0.8
0.4
0.2
0.1
−log10(p−value)
0
10
20
30
40
50
Recombination rate (cM/Mb)
0
20
40
60
80
100
ble levels of adhesion molecules
Cholesterol, total
Lipid metabolism phenotypes
7 GWAS hits omitted
C19orf66→ ←CDC37 SLC44A2→ ←TMED1 LDLR→ TSPAN16→ ←ECSIT ←ZNF823 ZNF700→
←ANGPTL6 PDE4A→ ←ILF3−AS1 CARM1→ ←SPC24 LPPR2→ CNN1→ ZNF441→ ←ZNF433
PPAN→ ←ICAM3 ATG4D→ DNM2→ SMARCA4→ ←DOCK6 PRKCSH→ ZNF833P→ ZNF69→
PPAN−P2RY11→ ←KEAP1 ILF3→ C19orf38→ ←KANK2 ←EPOR ←ELOF1 ZNF491→
SNORD105→ ←S1PR5 QTRT1→ ←YIPF2 C19orf80→ ←ELAVL3 ZNF440→
SNORD105B→ MIR1238→ MIR4748→ ←RAB3D ←ACP5 ZNF439→
P2RY11→ ←TYK2 ←KRI1 ←MIR199A1 ←TMEM205 ZNF627→ ZNF763→
←EIF3G ←MIR1181 MIR638→ C19orf52→ CCDC159→ ←ZNF878
←DNMT1 ←CDKN2D SWSAP1→ ZNF844→
←S1PR2 ←AP1M2 ←RGL3
10.5
11
11.5
12
Position on chr19 (Mb)

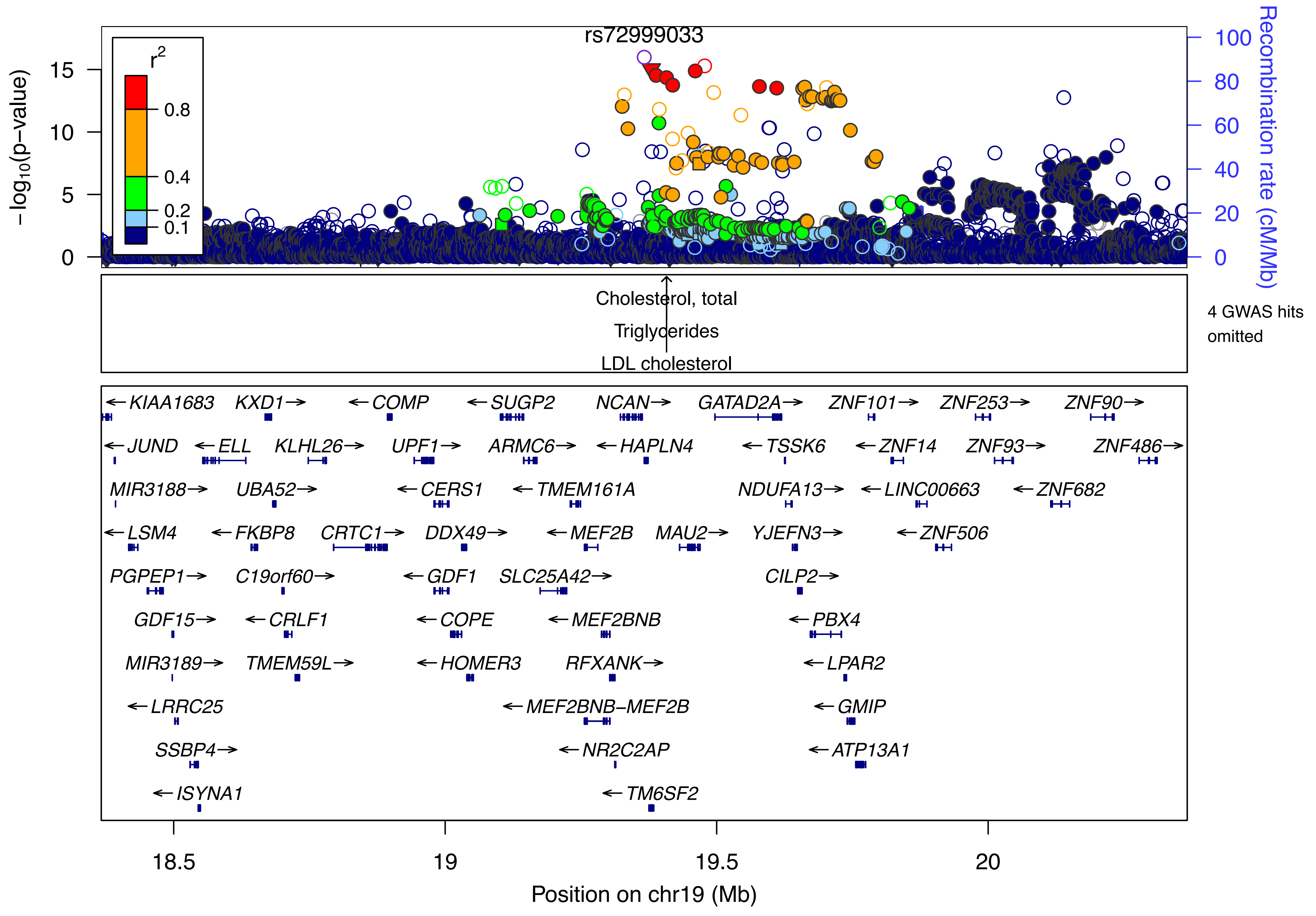
rs72999033
$-\log_{10}$(p−value)
Recombination rate (cM/Mb)
r^2
0.8
0.4
0.2
0.1
Cholesterol, total
Triglycerides
LDL cholesterol
4 GWAS hits omitted
←KIAA1683 KXD1→ ←COMP ←SUGP2 NCAN→ GATAD2A→ ZNF101→ ZNF253→ ZNF90→
←JUND ←ELL KLHL26→ UPF1→ ARMC6→ ←HAPLN4 ←TSSK6 ←ZNF14 ZNF93→ ZNF486→
MIR3188→ UBA52→ ←CERS1 ←TMEM161A NDUFA13→ ←LINC00663 ←ZNF682
←LSM4 ←FKBP8 CRTC1→ DDX49→ ←MEF2B MAU2→ YJEFN3→ ←ZNF506
PGPEP1→ C19orf60→ ←GDF1 SLC25A42→ CILP2→
GDF15→ ←CRLF1 ←COPE ←MEF2BNB ←PBX4
MIR3189→ TMEM59L→ ←HOMER3 RFXANK→ ←LPAR2
←LRRC25 ←MEF2BNB−MEF2B ←GMIP
SSBP4→ ←NR2C2AP ←ATP13A1
←ISYNA1 ←TM6SF2
18.5
19
19.5
20
Position on chr19 (Mb)

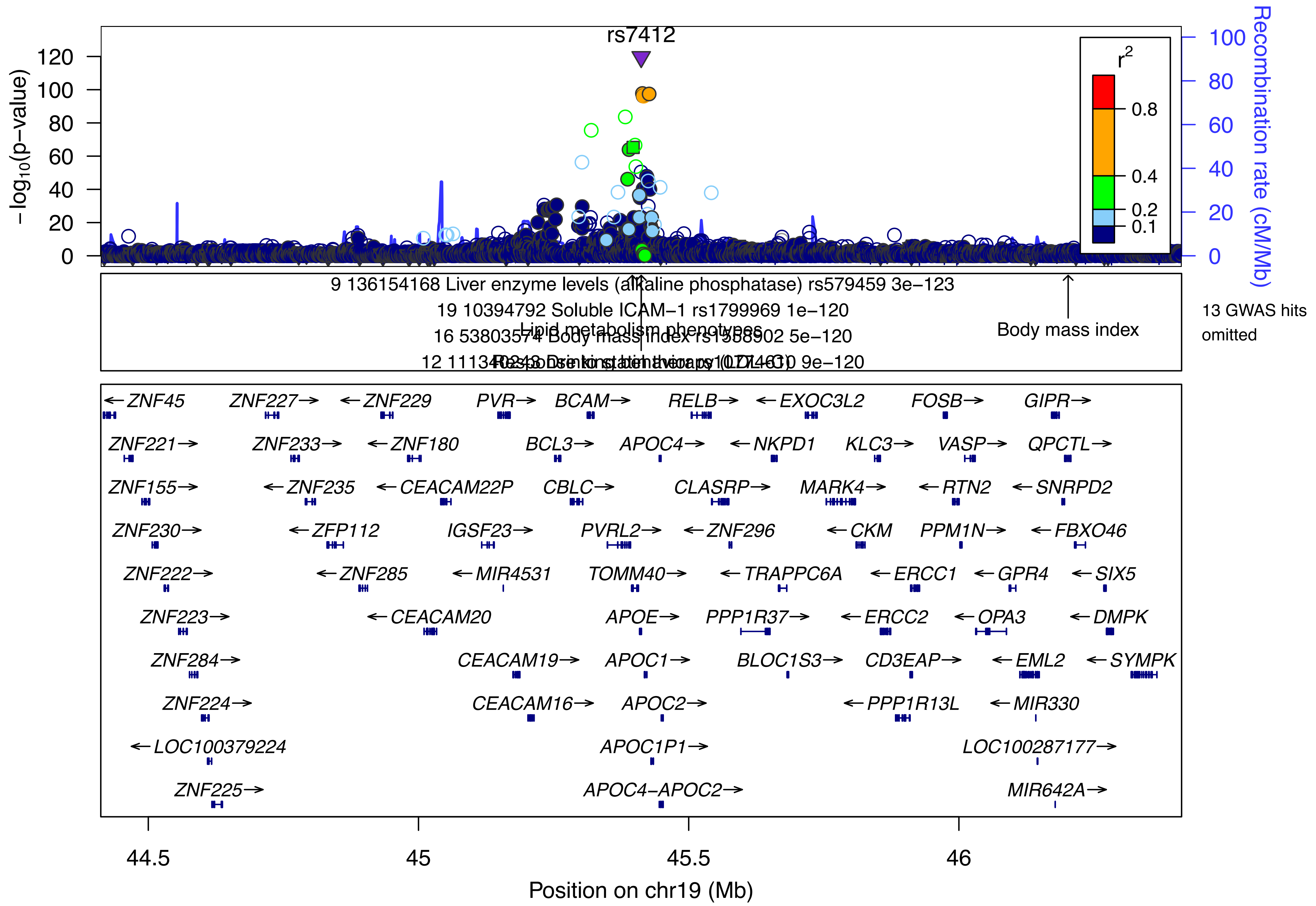
rs7412
−log10(p−value)
Recombination rate (cM/Mb)
r2
0.8
0.4
0.2
0.1
9 136154168 Liver enzyme levels (alkaline phosphatase) rs579459 3e−123
19 10394792 Soluble ICAM−1 rs1799969 1e−120
Body mass index
13 GWAS hits omitted
ZNF45 ZNF227 ZNF229 PVR BCAM RELB EXOC3L2 FOSB GIPR
ZNF221 ZNF233 ZNF180 BCL3 APOC4 NKPD1 KLC3 VASP QPCTL
ZNF155 ZNF235 CEACAM22P CBLC CLASRP MARK4 RTN2 SNRPD2
ZNF230 ZFP112 IGSF23 PVRL2 ZNF296 CKM PPM1N FBXO46
ZNF222 ZNF285 MIR4531 TOMM40 TRAPPC6A ERCC1 GPR4 SIX5
ZNF223 CEACAM20 APOE PPP1R37 ERCC2 OPA3 DMPK
ZNF284 CEACAM19 APOC1 BLOC1S3 CD3EAP EML2 SYMPK
ZNF224 CEACAM16 APOC2 PPP1R13L MIR330
LOC100379224 APOC1P1 LOC100287177
ZNF225 APOC4−APOC2 MIR642A
44.5
45
45.5
46
Position on chr19 (Mb)

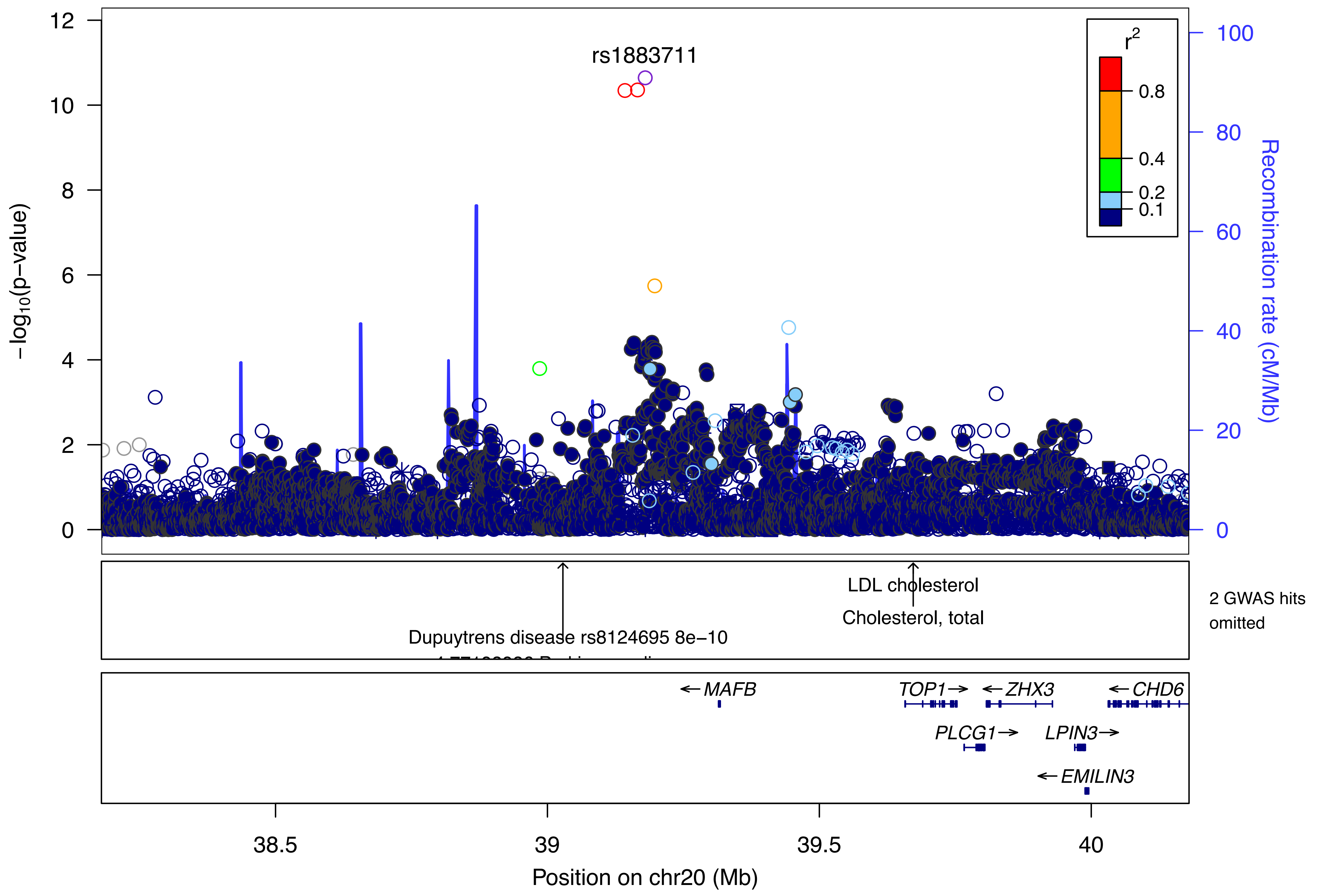

rs1883711
$-\log_{10}$(p−value)
Recombination rate (cM/Mb)
r^2
0.8
0.4
0.2
0.1
Dupuytrens disease rs8124695 8e−10
LDL cholesterol
Cholesterol, total
2 GWAS hits omitted
← MAFB
TOP1 →
← ZHX3
← CHD6
PLCG1 →
LPIN3 →
← EMILIN3
38.5
39
39.5
40
Position on chr20 (Mb)

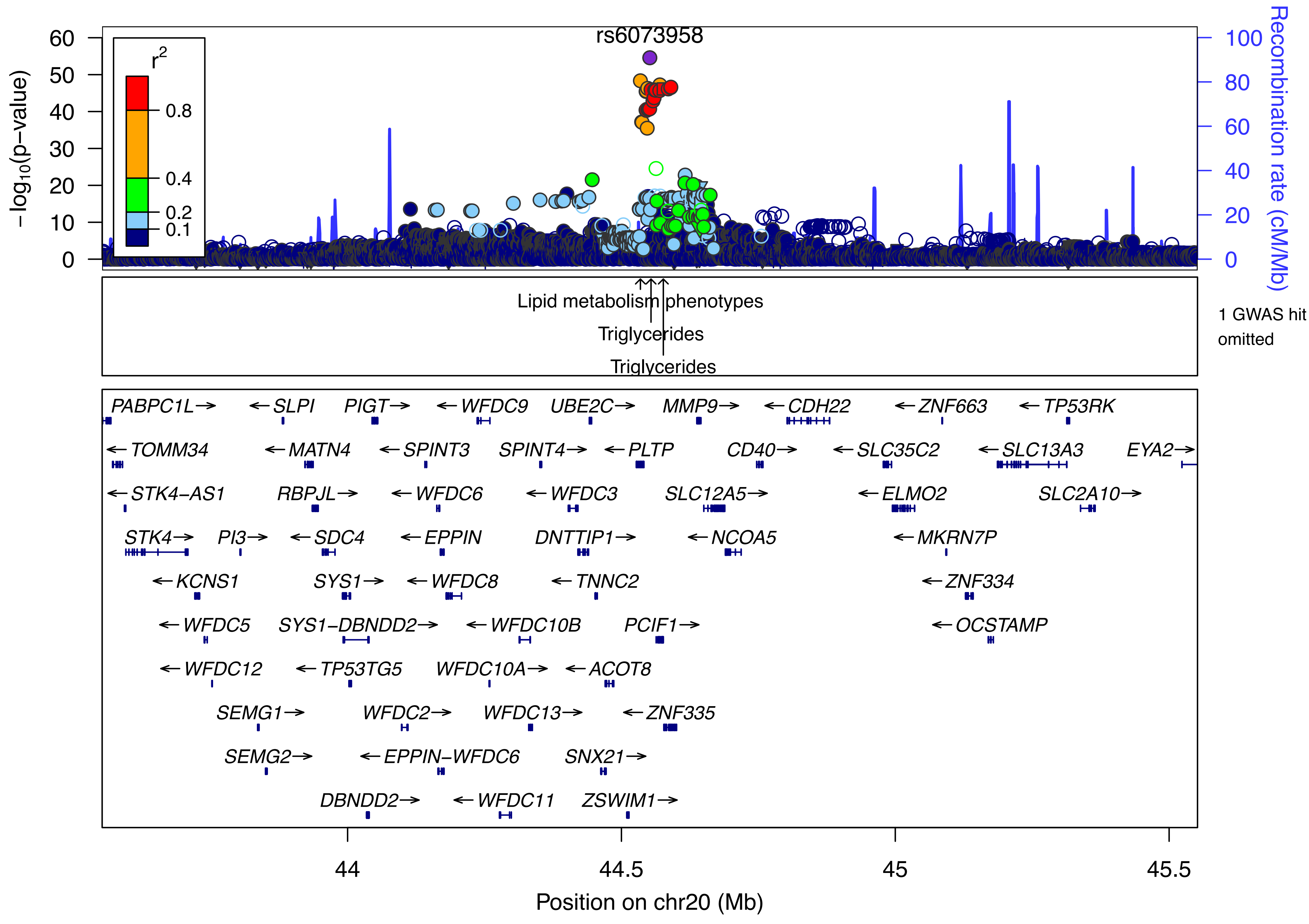
rs6073958
−log10(p−value)
Recombination rate (cM/Mb)
r2
0.8
0.4
0.2
0.1
Lipid metabolism phenotypes
Triglycerides
Triglycerides
1 GWAS hit omitted
PABPC1L→
←SLPI
PIGT→
←WFDC9
UBE2C→
MMP9→
←CDH22
←ZNF663
←TP53RK
←TOMM34
←MATN4
←SPINT3
SPINT4→
←PLTP
CD40→
←SLC35C2
←SLC13A3
EYA2→
←STK4−AS1
RBPJL→
←WFDC6
←WFDC3
SLC12A5→
←ELMO2
SLC2A10→
STK4→
PI3→
←SDC4
←EPPIN
DNTTIP1→
←NCOA5
←MKRN7P
←KCNS1
SYS1→
←WFDC8
←TNNC2
←ZNF334
←WFDC5
SYS1−DBNDD2→
←WFDC10B
PCIF1→
←OCSTAMP
←WFDC12
←TP53TG5
WFDC10A→
←ACOT8
SEMG1→
WFDC2→
WFDC13→
←ZNF335
SEMG2→
←EPPIN−WFDC6
SNX21→
DBNDD2→
←WFDC11
ZSWIM1→
44
44.5
45
45.5
Position on chr20 (Mb)

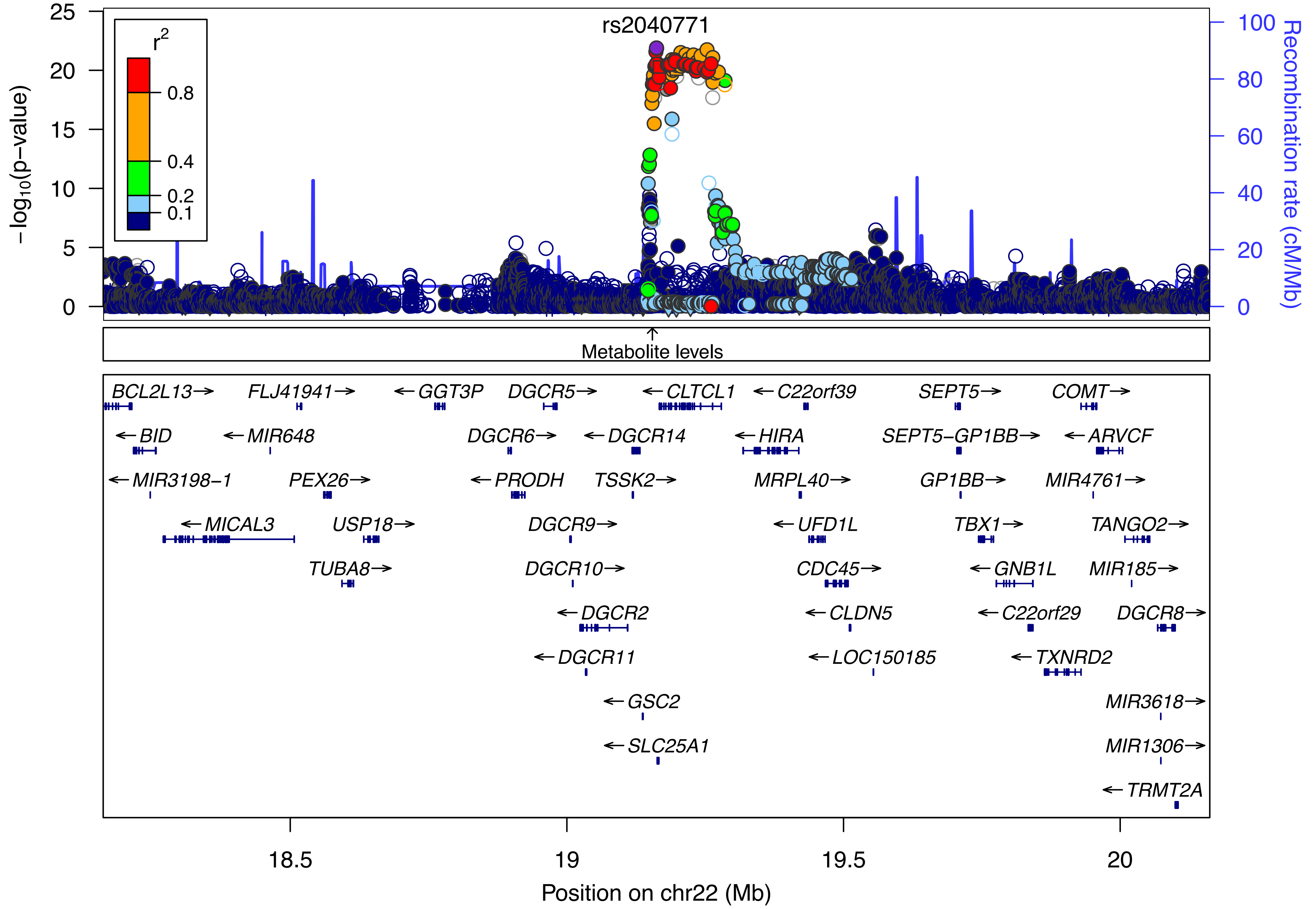
rs2040771
r2
0.8
0.4
0.2
0.1
−log10(p−value)
Recombination rate (cM/Mb)
Metabolite levels
BCL2L13→
FLJ41941→
←GGT3P
DGCR5→
←CLTCL1
←C22orf39
SEPT5→
COMT→
←BID
←MIR648
DGCR6→
←DGCR14
←HIRA
SEPT5−GP1BB→
←ARVCF
←MIR3198−1
PEX26→
←PRODH
TSSK2→
MRPL40→
GP1BB→
MIR4761→
←MICAL3
USP18→
DGCR9→
←UFD1L
TBX1→
TANGO2→
TUBA8→
DGCR10→
CDC45→
←GNB1L
MIR185→
←DGCR2
←CLDN5
←C22orf29
DGCR8→
←DGCR11
←LOC150185
←TXNRD2
←GSC2
MIR3618→
←SLC25A1
MIR1306→
←TRMT2A
Position on chr22 (Mb)

Supplementary Figure 2. Forest plots of individual cohort and meta-analysis association results for all lead SNP associations in **Table 2** and **Supplementary Table 4**.

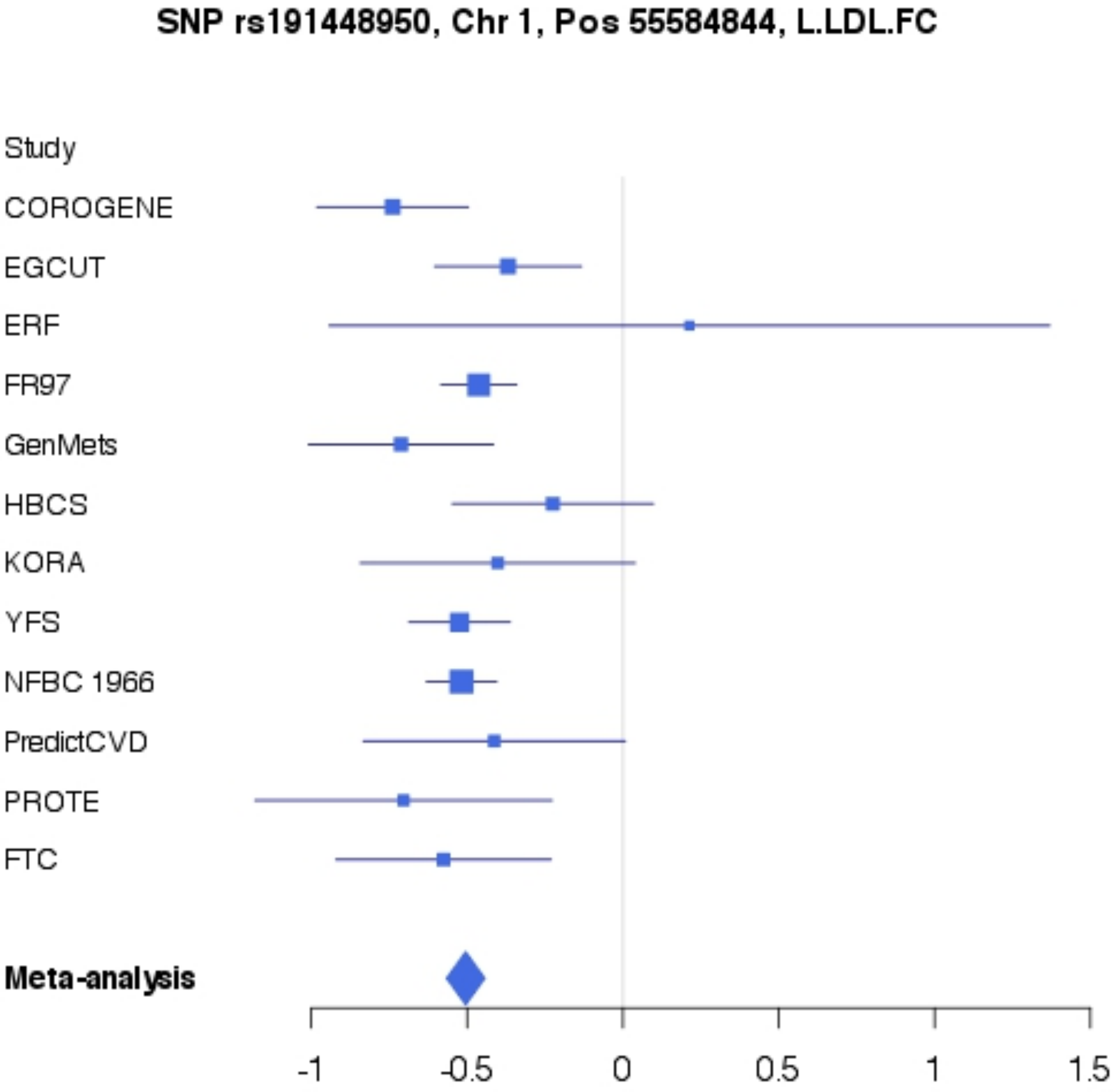
SNP rs191448950, Chr 1, Pos 55584844, L.LDL.FC
Study
COROGENE
EGCUT
ERF
FR97
GenMets
HBCS
KORA
YFS
NFBC 1966
PredictCVD
PROTE
FTC
Meta-analysis
-1
-0.5
0
0.5
1
1.5

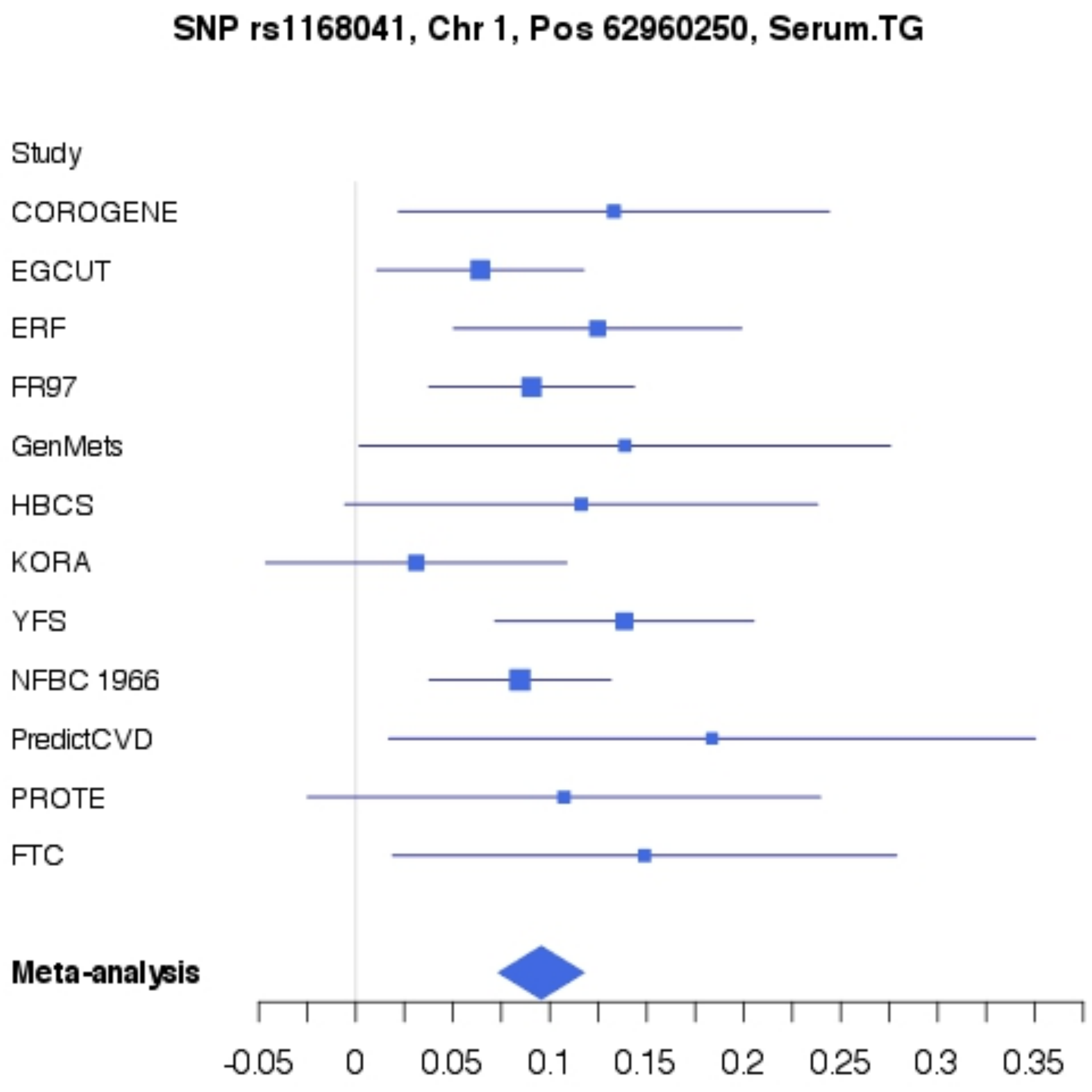

SNP rs1168041, Chr 1, Pos 62960250, Serum.TG
Study
COROGENE
EGCUT
ERF
FR97
GenMets
HBCS
KORA
YFS
NFBC 1966
PredictCVD
PROTE
FTC
Meta-analysis
-0.05
0
0.05
0.1
0.15
0.2
0.25
0.3
0.35

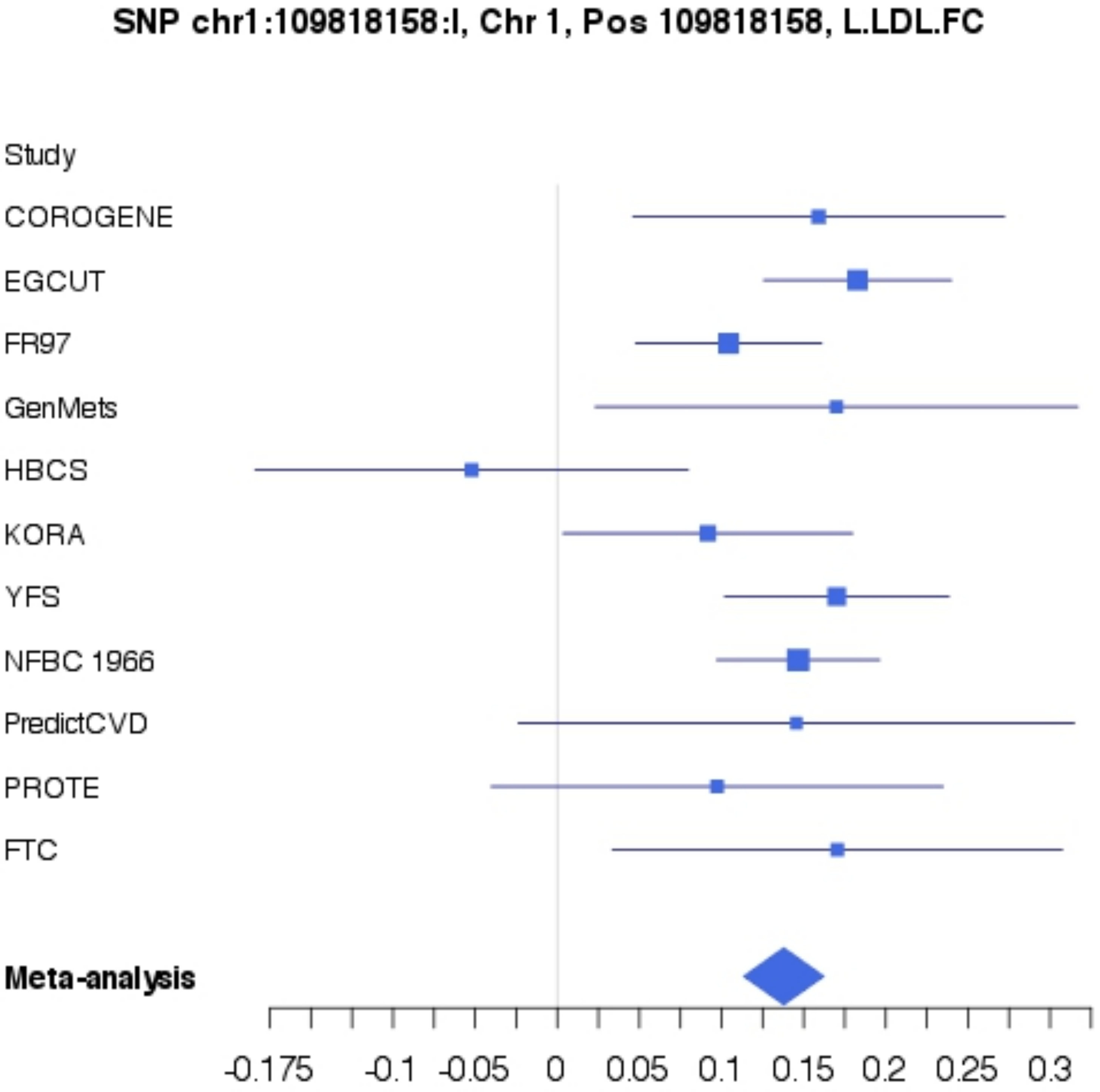

SNP chr1:109818158:I, Chr 1, Pos 109818158, L.LDL.FC
Study
COROGENE
EGCUT
FR97
GenMets
HBCS
KORA
YFS
NFBC 1966
PredictCVD
PROTE
FTC
Meta-analysis
-0.175
-0.1
-0.05
0
0.05
0.1
0.15
0.2
0.25
0.3

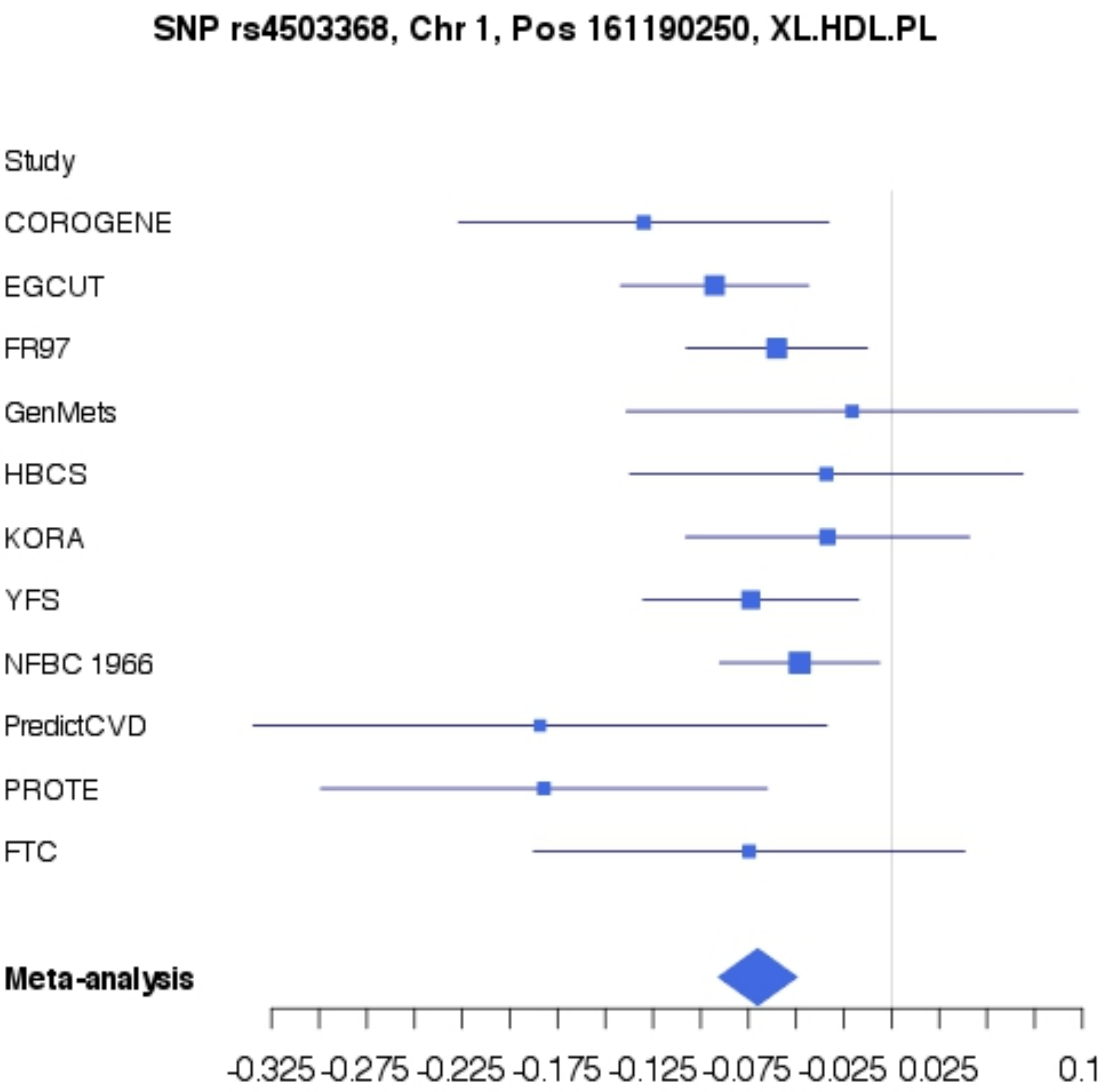
SNP rs4503368, Chr 1, Pos 161190250, XL.HDL.PL
Study
COROGENE
EGCUT
FR97
GenMets
HBCS
KORA
YFS
NFBC 1966
PredictCVD
PROTE
FTC
Meta-analysis
-0.325
-0.275
-0.225
-0.175
-0.125
-0.075
-0.025
0.025
0.1

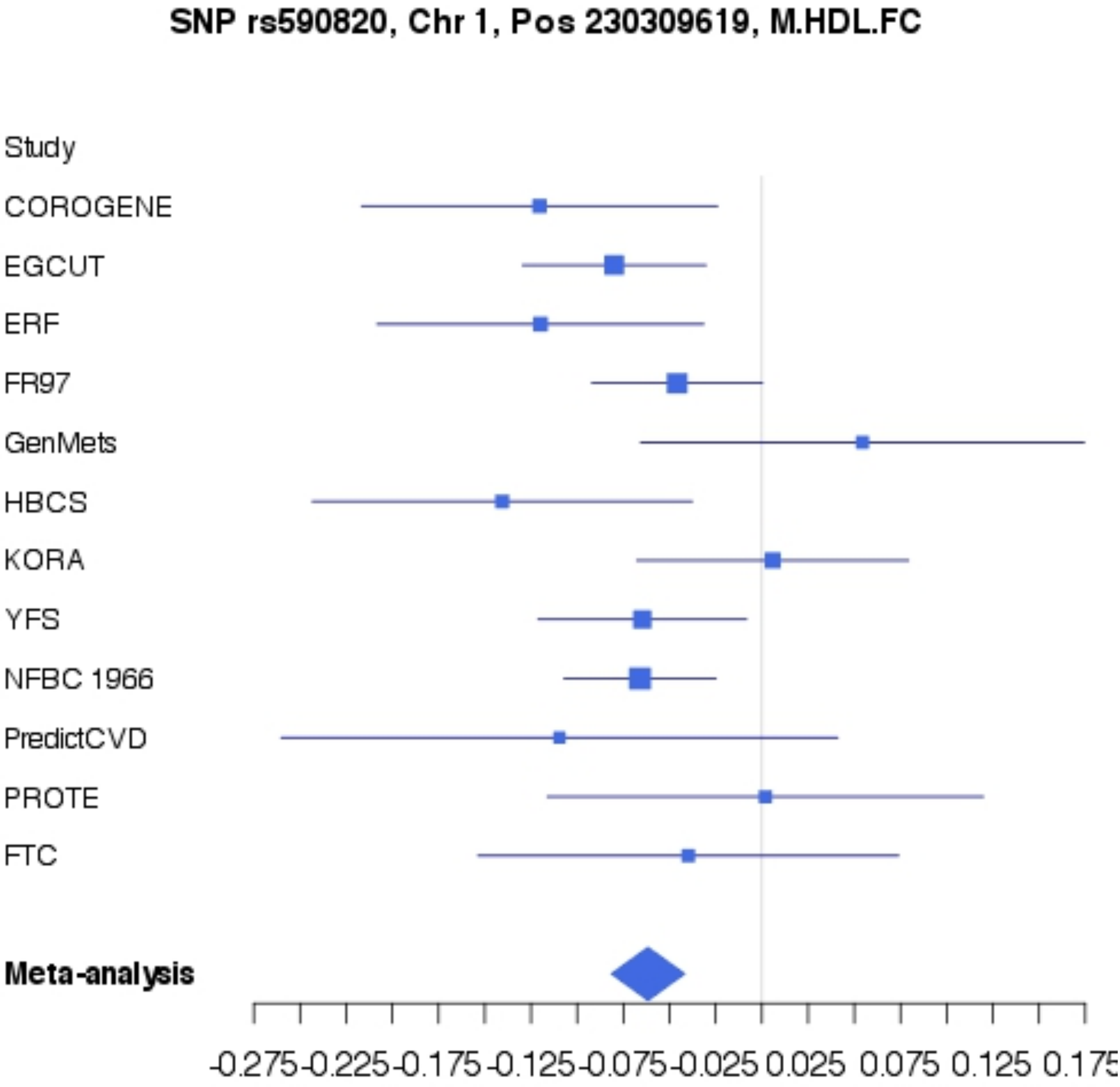
SNP rs590820, Chr 1, Pos 230309619, M.HDL.FC
Study
COROGENE
EGCUT
ERF
FR97
GenMets
HBCS
KORA
YFS
NFBC 1966
PredictCVD
PROTE
FTC
Meta-analysis
-0.275
-0.225
-0.175
-0.125
-0.075
-0.025
0.025
0.075
0.125

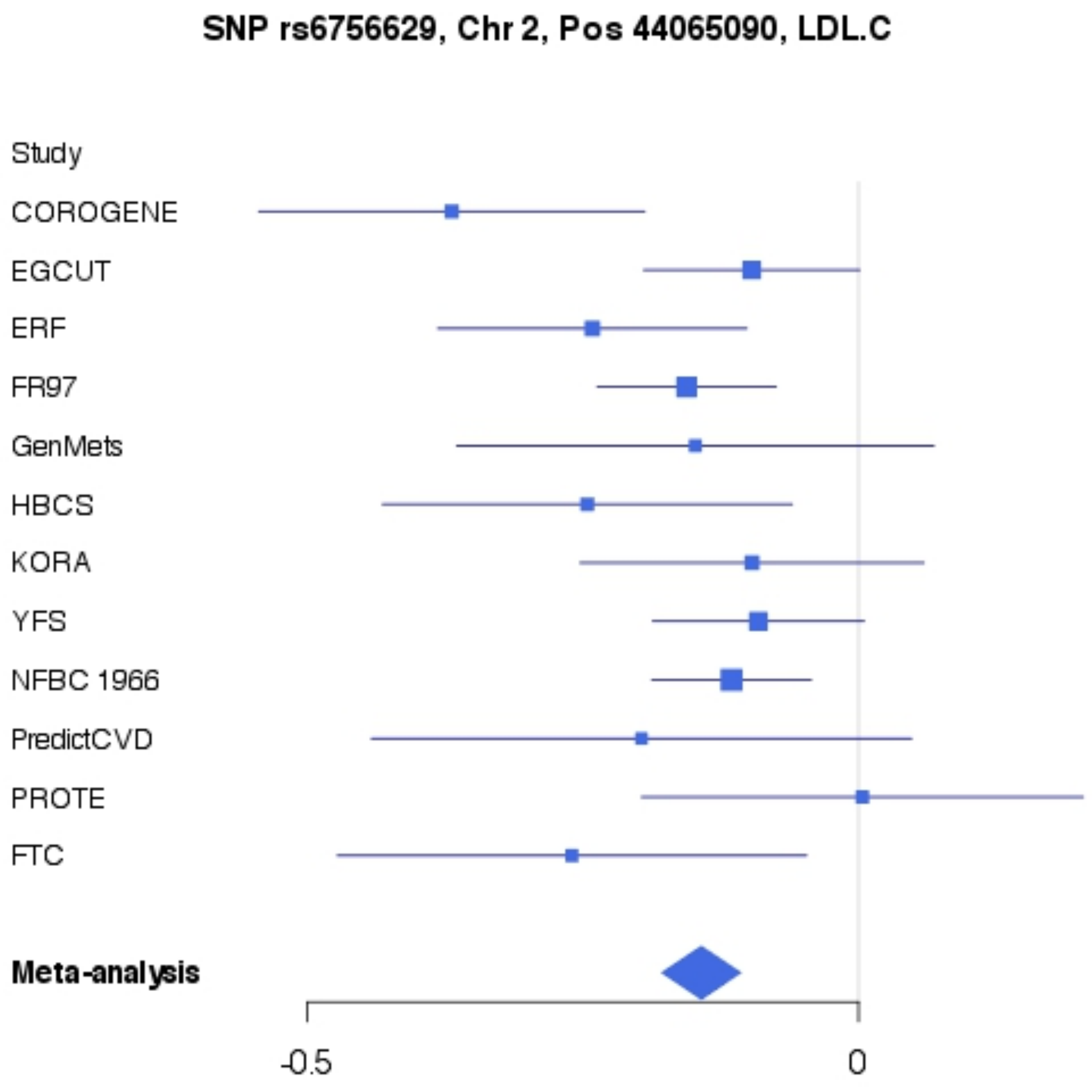
SNP rs6756629, Chr 2, Pos 44065090, LDL.C
Study
COROGENE
EGCUT
ERF
FR97
GenMets
HBCS
KORA
YFS
NFBC 1966
PredictCVD
PROTE
FTC
Meta-analysis
-0.5
0

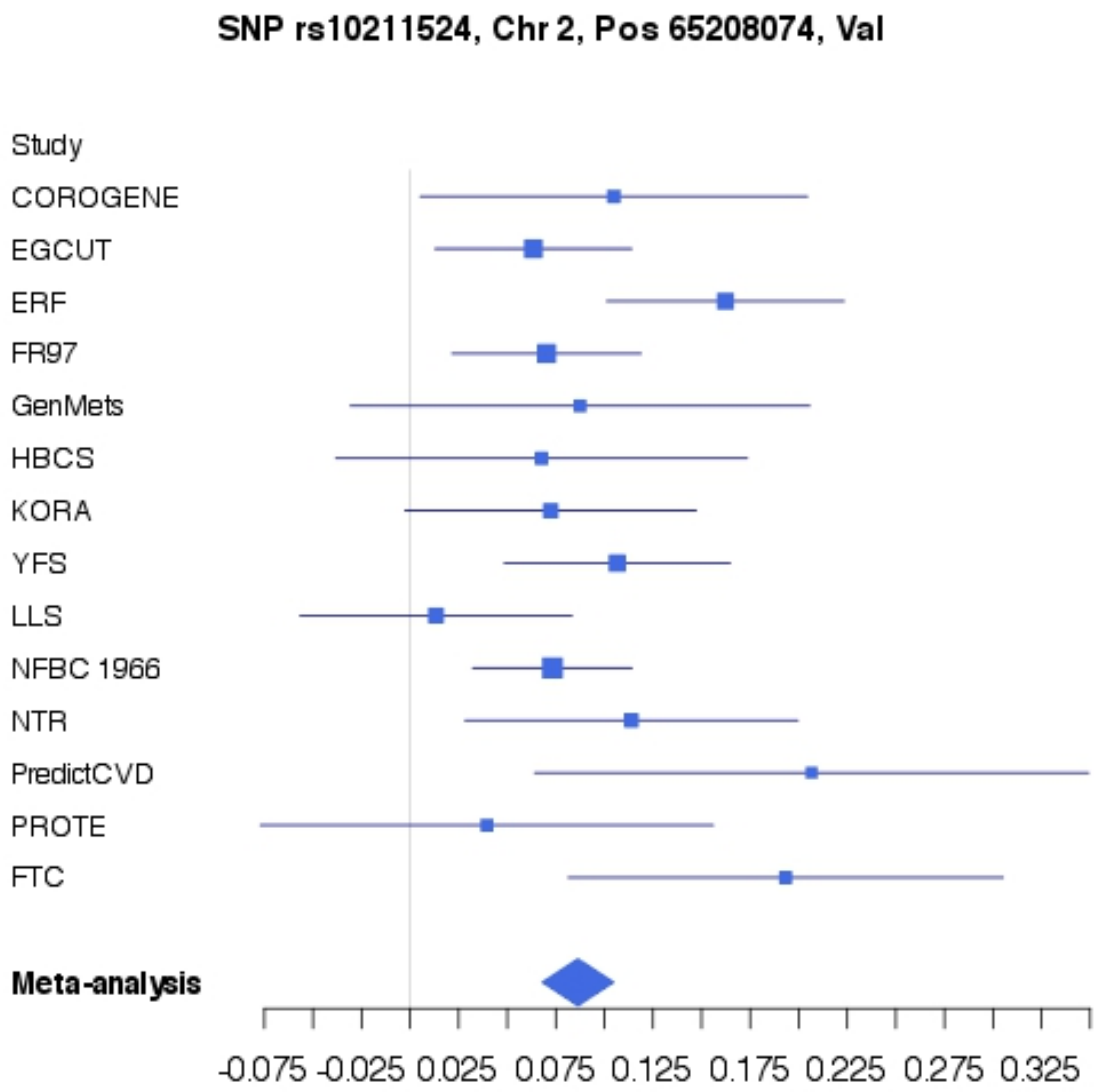

SNP rs10211524, Chr 2, Pos 65208074, Val
Study
COROGENE
EGCUT
ERF
FR97
GenMets
HBCS
KORA
YFS
LLS
NFBC 1966
NTR
PredictCVD
PROTE
FTC
Meta-analysis
-0.075
-0.025
0.025
0.075
0.125
0.175
0.225
0.275
0.325

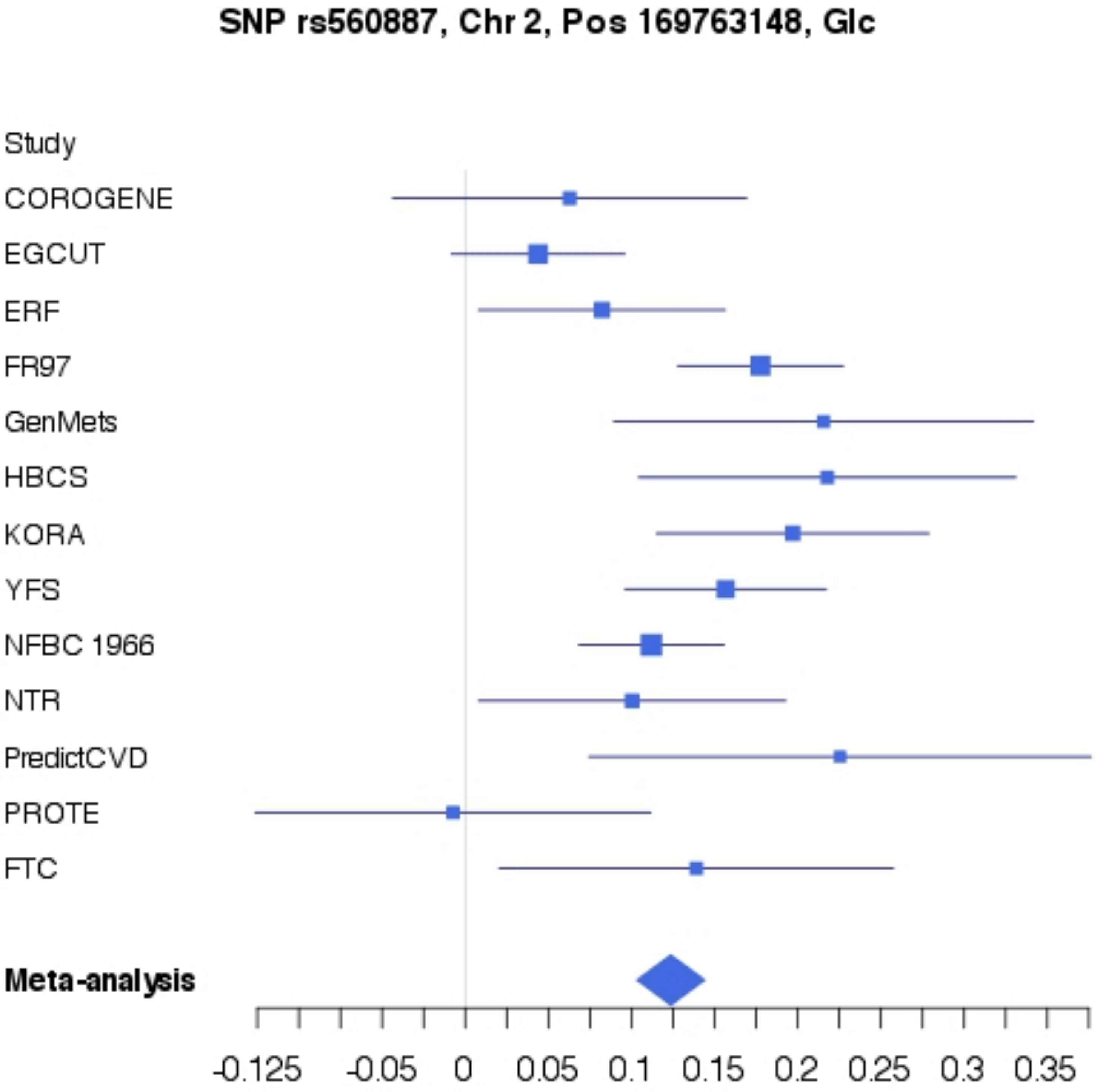

SNP rs560887, Chr 2, Pos 169763148, Glc
Study
COROGENE
EGCUT
ERF
FR97
GenMets
HBCS
KORA
YFS
NFBC 1966
NTR
PredictCVD
PROTE
FTC
Meta-analysis
-0.125
-0.05
0
0.05
0.1
0.15
0.2
0.25
0.3
0.35

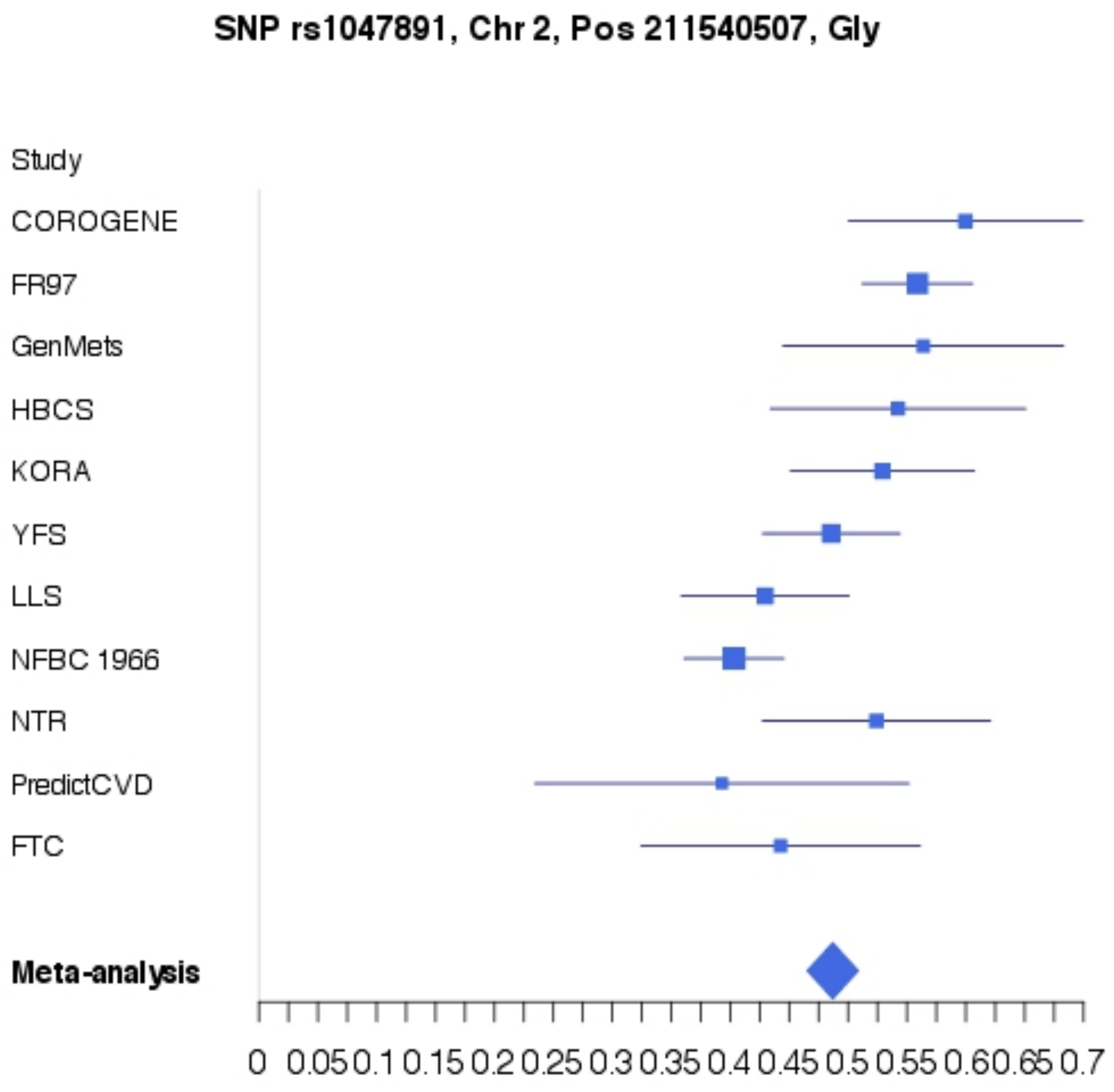
SNP rs1047891, Chr 2, Pos 211540507, Gly
Study
COROGENE
FR97
GenMets
HBCS
KORA
YFS
LLS
NFBC 1966
NTR
PredictCVD
FTC
Meta-analysis
0
0.05
0.1
0.15
0.2
0.25
0.3
0.35
0.4
0.45
0.5
0.55
0.6
0.65
0.7

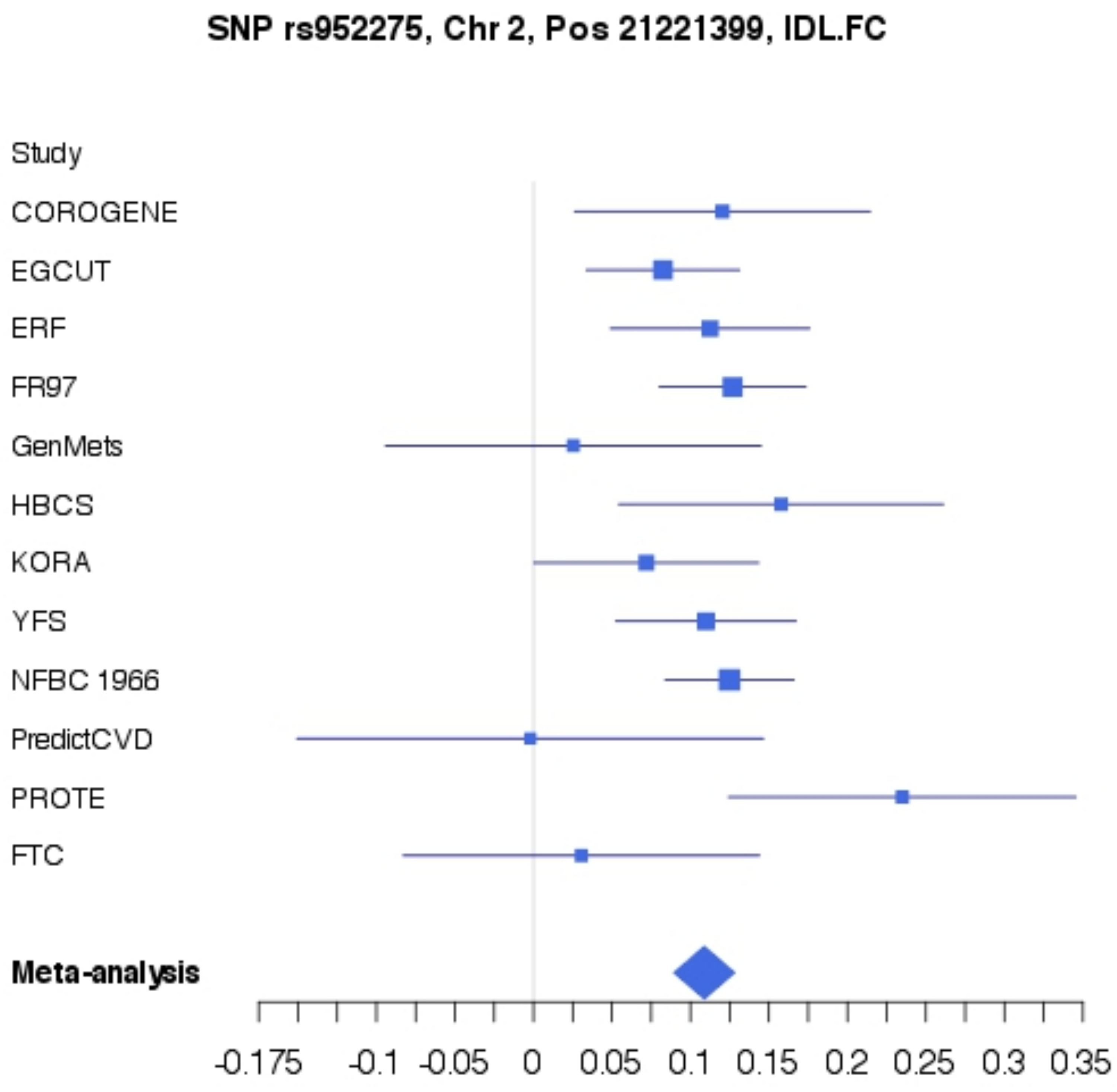
SNP rs952275, Chr 2, Pos 21221399, IDL.FC
Study
COROGENE
EGCUT
ERF
FR97
GenMets
HBCS
KORA
YFS
NFBC 1966
PredictCVD
PROTE
FTC
Meta-analysis
-0.175
-0.1
-0.05
0
0.05
0.1
0.15
0.2
0.25
0.3
0.35

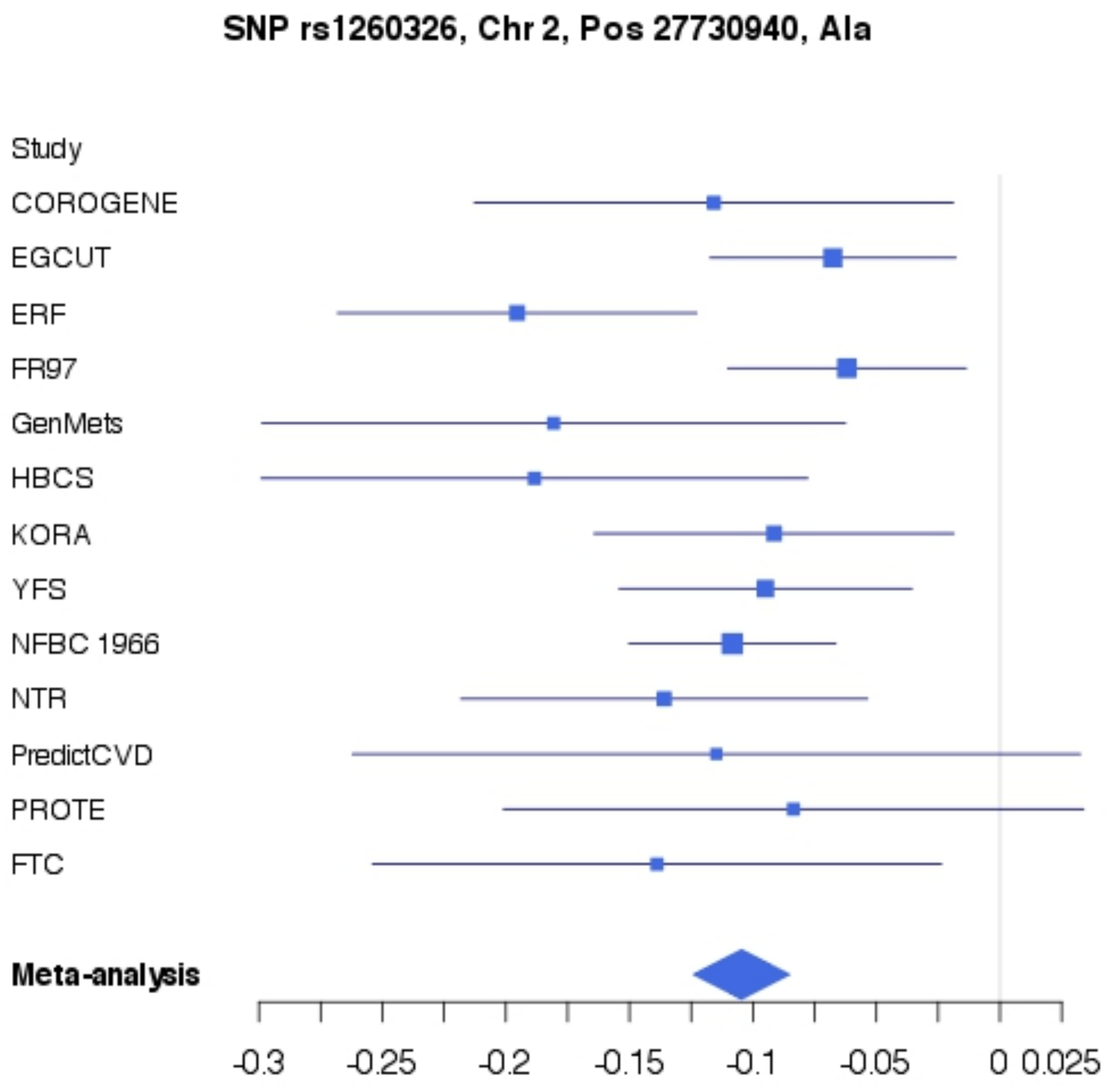

SNP rs1260326, Chr 2, Pos 27730940, Ala
Study
COROGENE
EGCUT
ERF
FR97
GenMets
HBCS
KORA
YFS
NFBC 1966
NTR
PredictCVD
PROTE
FTC
Meta-analysis
-0.3
-0.25
-0.2
-0.15
-0.1
-0.05
0
0.025

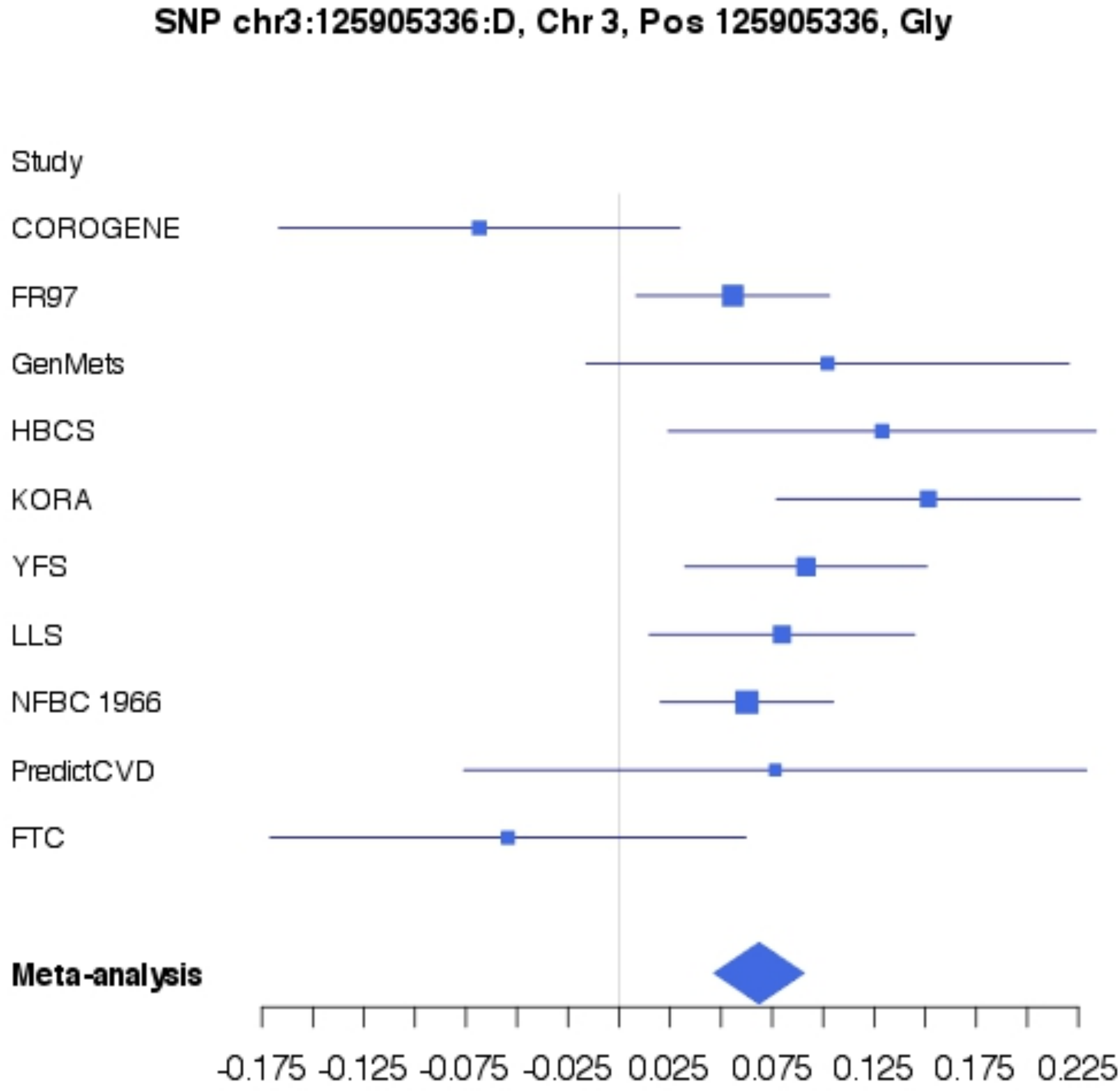

SNP chr3:125905336:D, Chr 3, Pos 125905336, Gly
Study
COROGENE
FR97
GenMets
HBCS
KORA
YFS
LLS
NFBC 1966
PredictCVD
FTC
Meta-analysis
-0.175
-0.125
-0.075
-0.025
0.025
0.075
0.125
0.175
0.225

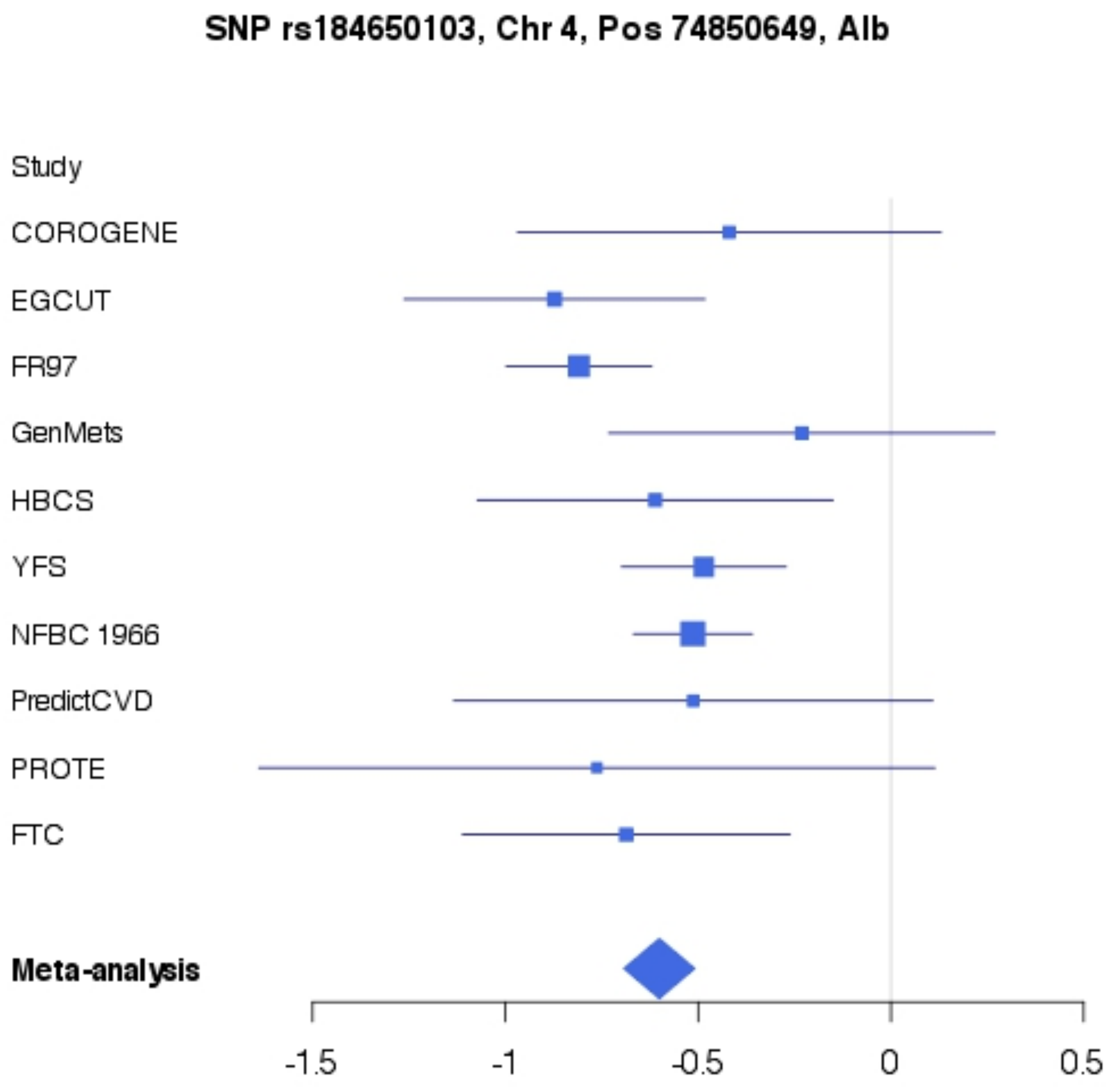
SNP rs184650103, Chr 4, Pos 74850649, Alb
Study
COROGENE
EGCUT
FR97
GenMets
HBCS
YFS
NFBC 1966
PredictCVD
PROTE
FTC
Meta-analysis
-1.5
-1
-0.5
0
0.5

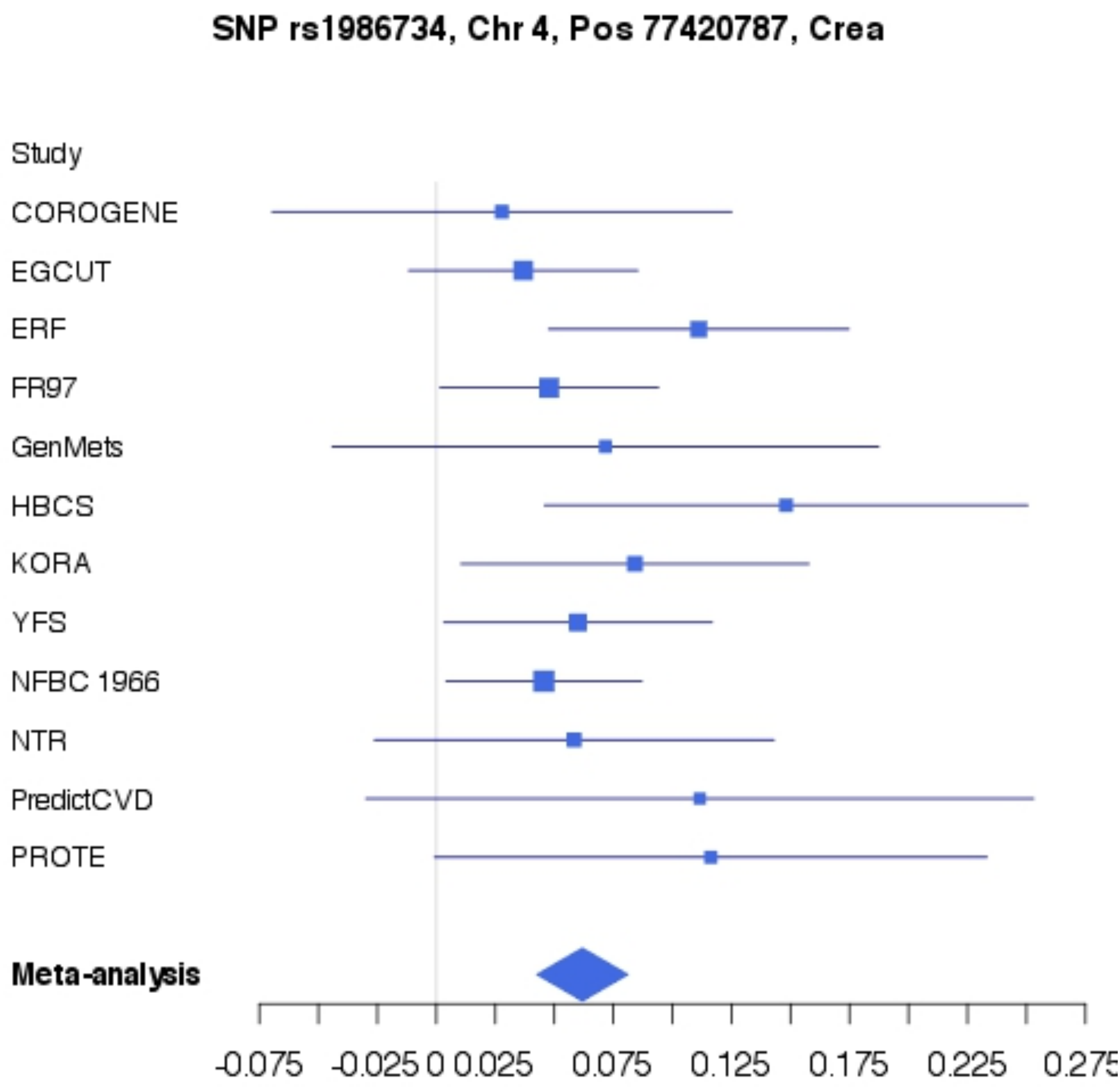
SNP rs1986734, Chr 4, Pos 77420787, Crea
Study
COROGENE
EGCUT
ERF
FR97
GenMets
HBCS
KORA
YFS
NFBC 1966
NTR
PredictCVD
PROTE
Meta-analysis
-0.075
-0.025
0
0.025
0.075
0.125
0.175
0.225

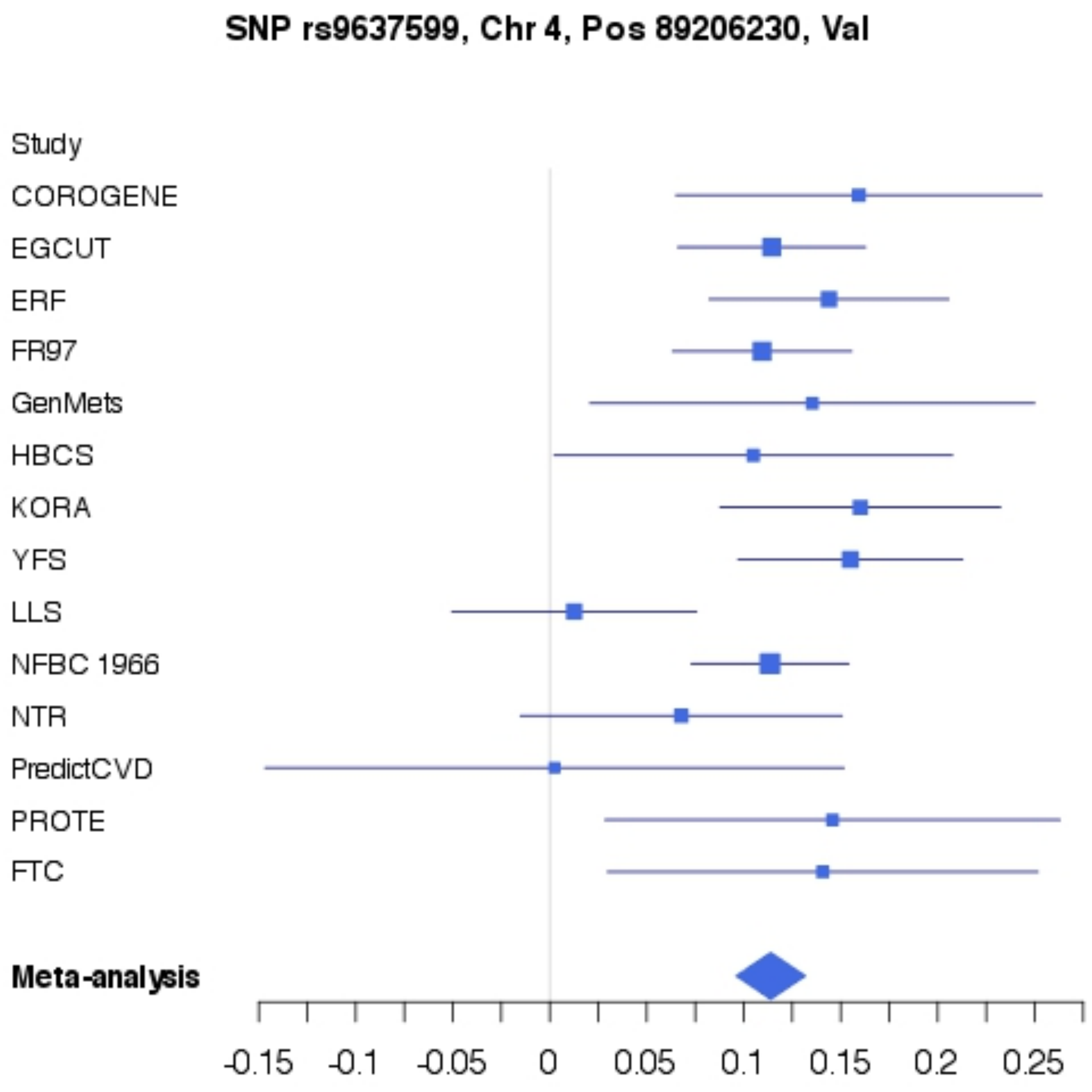
SNP rs9637599, Chr 4, Pos 89206230, Val
Study
COROGENE
EGCUT
ERF
FR97
GenMets
HBCS
KORA
YFS
LLS
NFBC 1966
NTR
PredictCVD
PROTE
FTC
Meta-analysis
-0.15
-0.1
-0.05
0
0.05
0.1
0.15
0.2
0.25

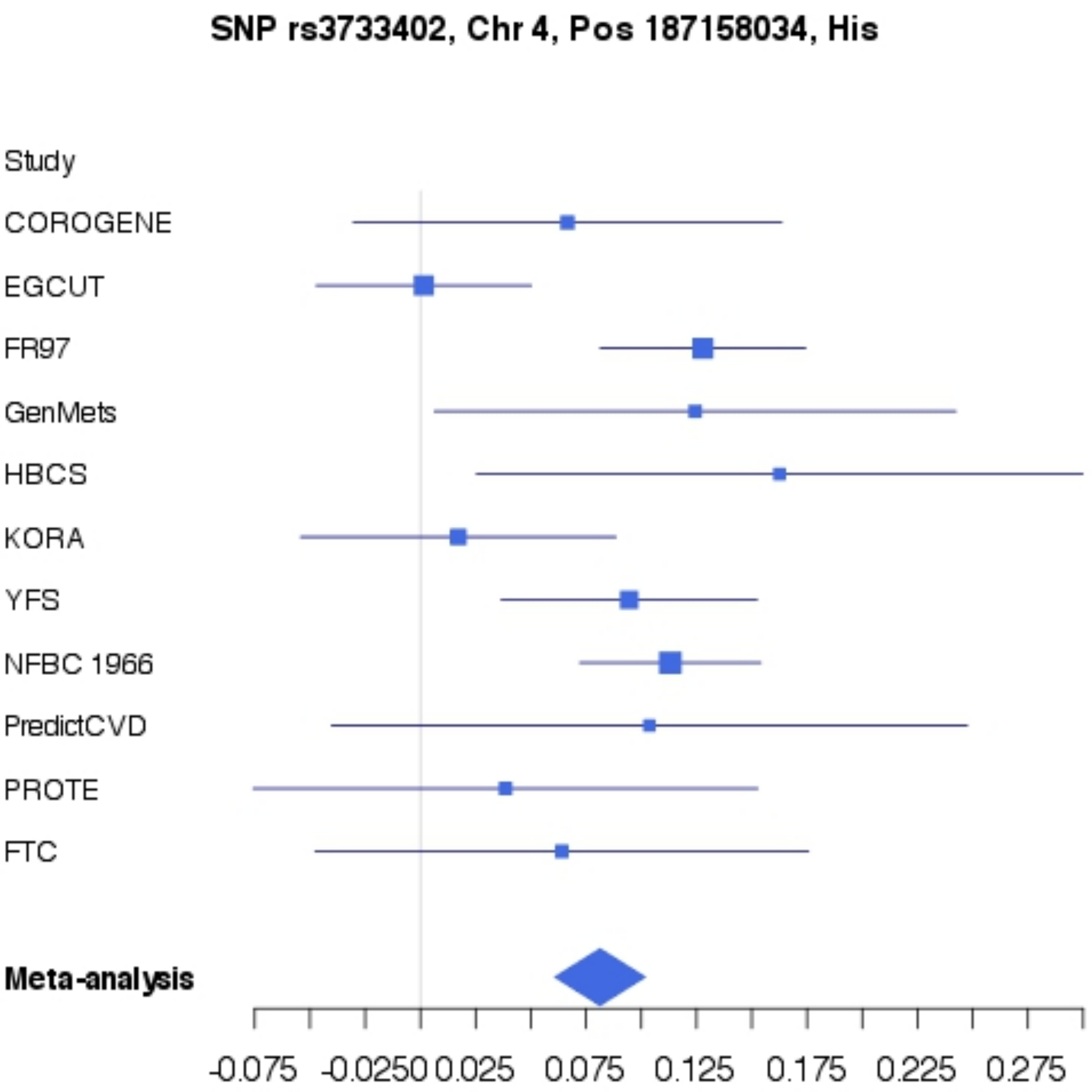
SNP rs3733402, Chr 4, Pos 187158034, His
Study
COROGENE
EGCUT
FR97
GenMets
HBCS
KORA
YFS
NFBC 1966
PredictCVD
PROTE
FTC
Meta-analysis
-0.075
-0.025
0
0.025
0.075
0.125
0.175
0.225
0.275

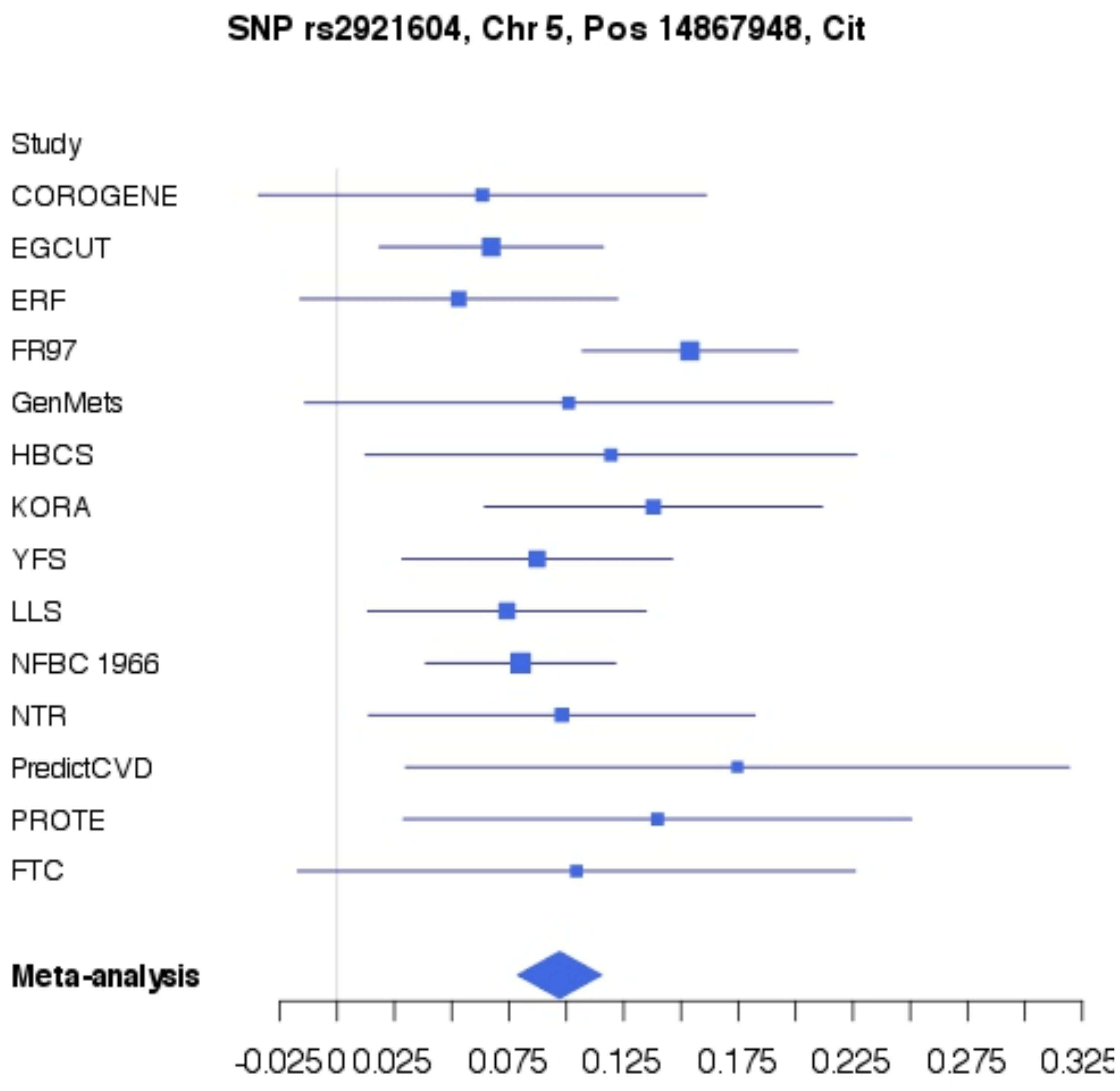
SNP rs2921604, Chr 5, Pos 14867948, Cit
Study
COROGENE
EGCUT
ERF
FR97
GenMets
HBCS
KORA
YFS
LLS
NFBC 1966
NTR
PredictCVD
PROTE
FTC
Meta-analysis
-0.025
0
0.025
0.075
0.125
0.175
0.225
0.275
0.325

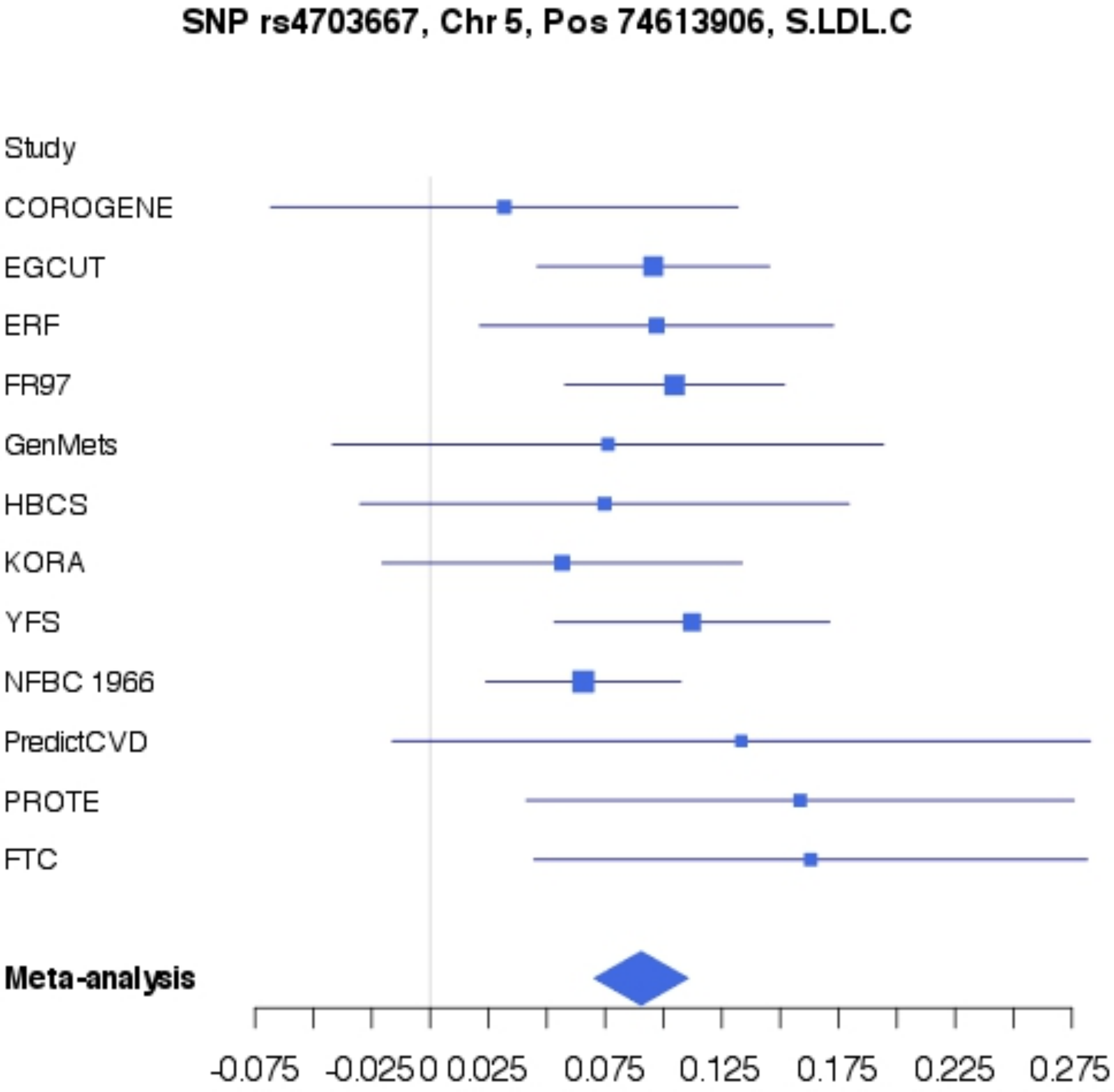

SNP rs4703667, Chr 5, Pos 74613906, S.LDL.C
Study
COROGENE
EGCUT
ERF
FR97
GenMets
HBCS
KORA
YFS
NFBC 1966
PredictCVD
PROTE
FTC
Meta-analysis
-0.075
-0.025
0
0.025
0.075
0.125
0.175
0.225
0.275

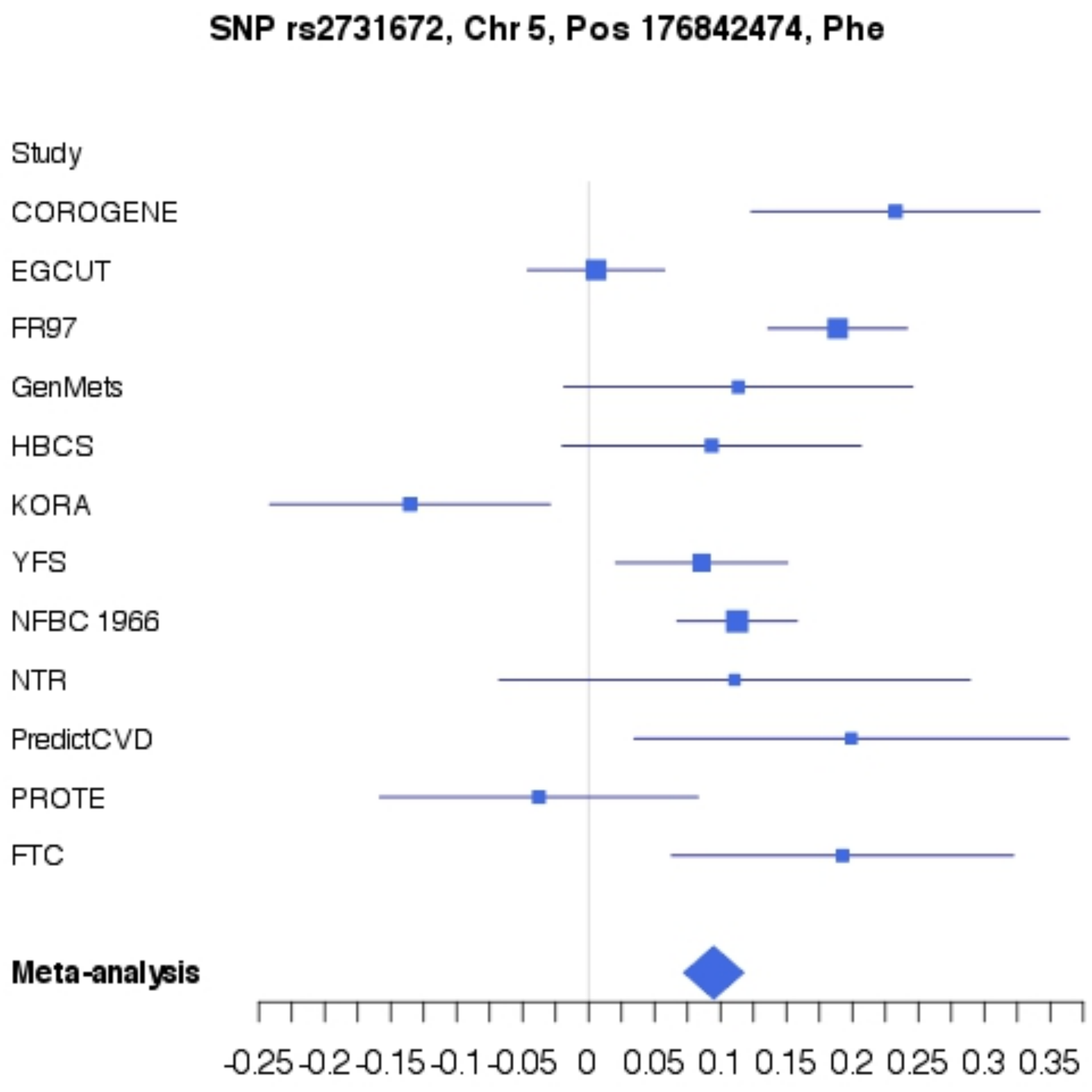
SNP rs2731672, Chr 5, Pos 176842474, Phe
Study
COROGENE
EGCUT
FR97
GenMets
HBCS
KORA
YFS
NFBC 1966
NTR
PredictCVD
PROTE
FTC
Meta-analysis
-0.25 -0.2 -0.15 -0.1 -0.05 0 0.05 0.1 0.15 0.2 0.25 0.3 0.35

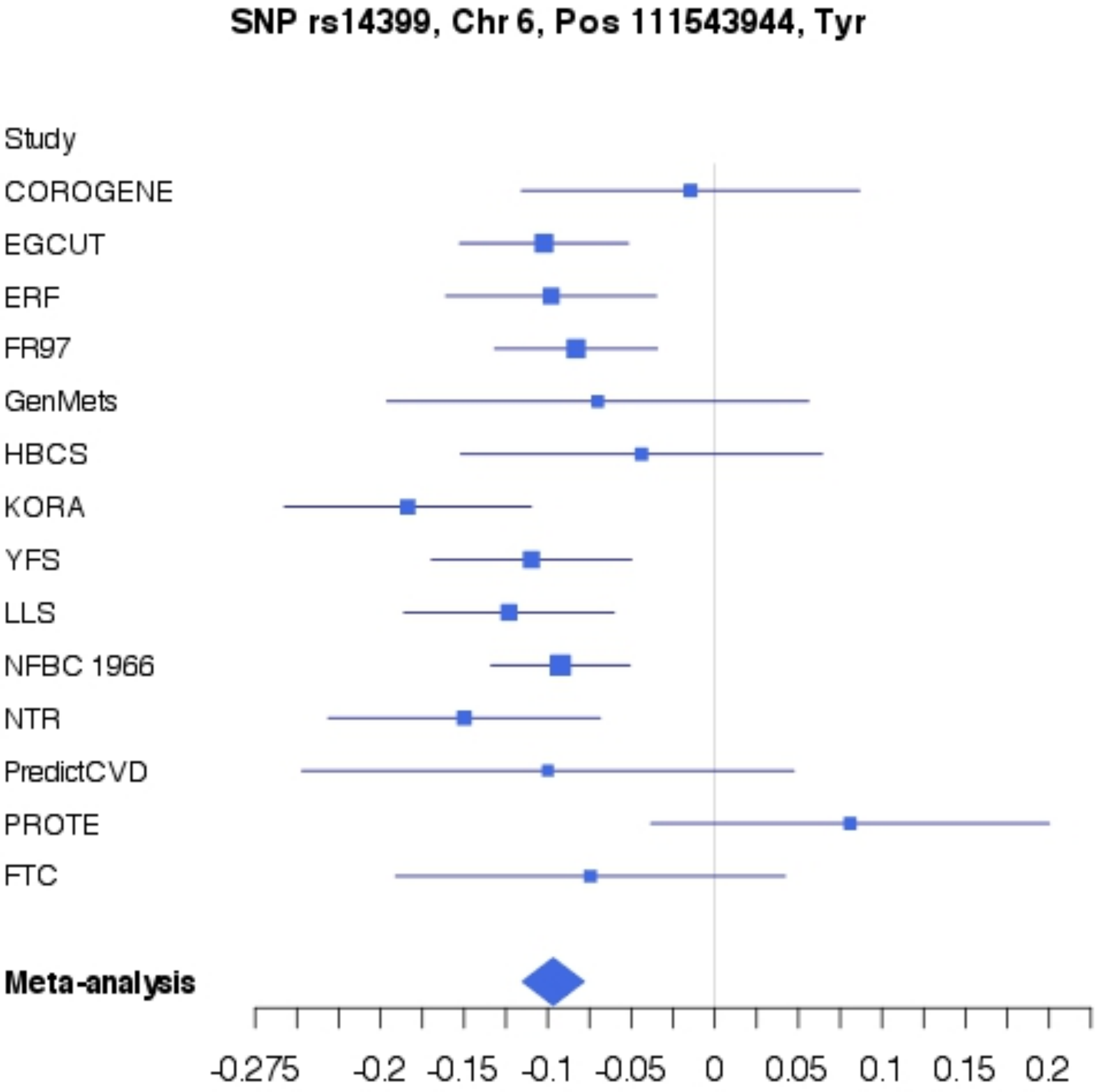
SNP rs14399, Chr 6, Pos 111543944, Tyr
Study
COROGENE
EGCUT
ERF
FR97
GenMets
HBCS
KORA
YFS
LLS
NFBC 1966
NTR
PredictCVD
PROTE
FTC
Meta-analysis
-0.275
-0.2
-0.15
-0.1
-0.05
0
0.05
0.1
0.15
0.2

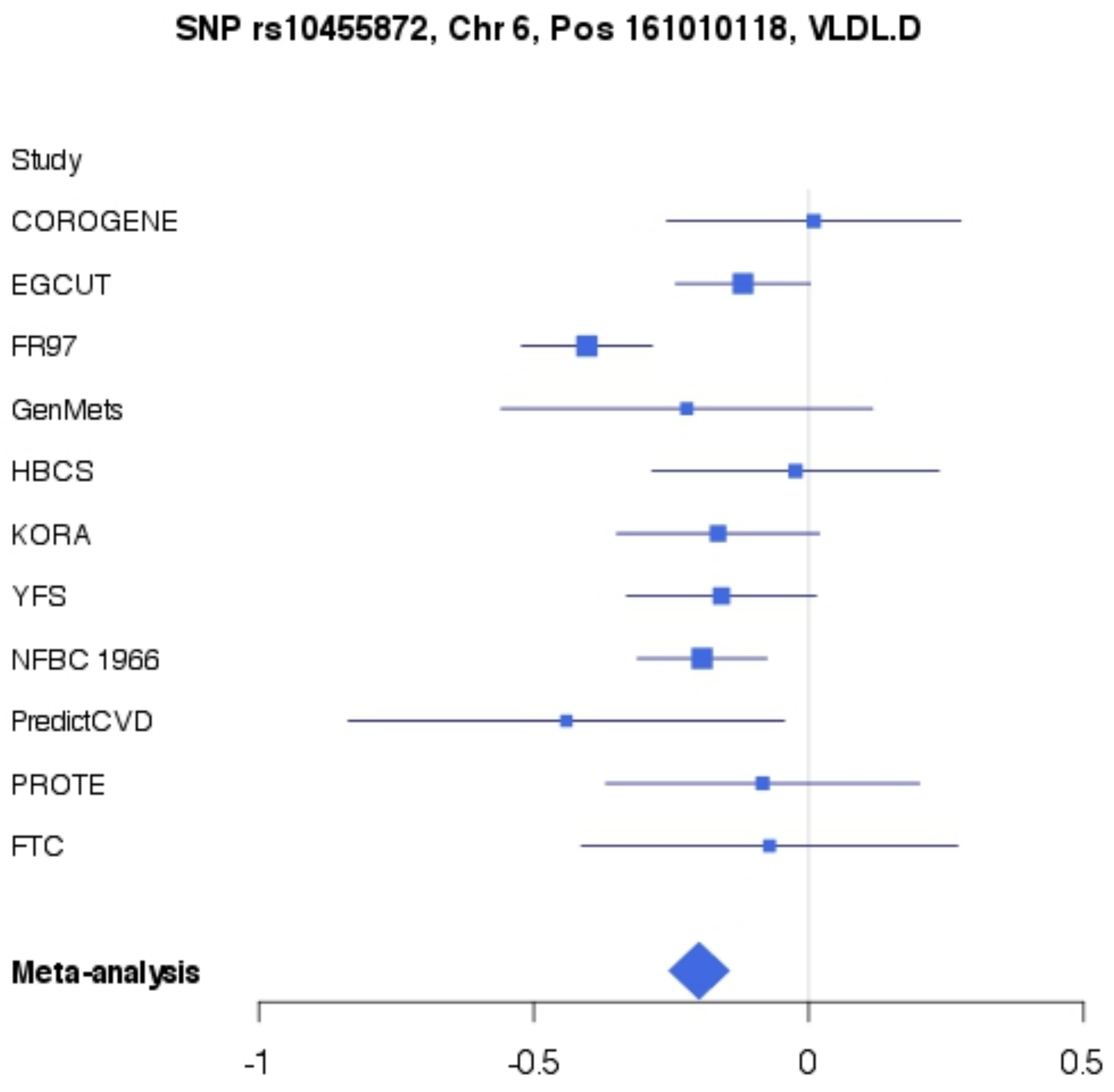

SNP rs10455872, Chr 6, Pos 161010118, VLDL.D
Study
COROGENE
EGCUT
FR97
GenMets
HBCS
KORA
YFS
NFBC 1966
PredictCVD
PROTE
FTC
Meta-analysis
-1
-0.5
0
0.5

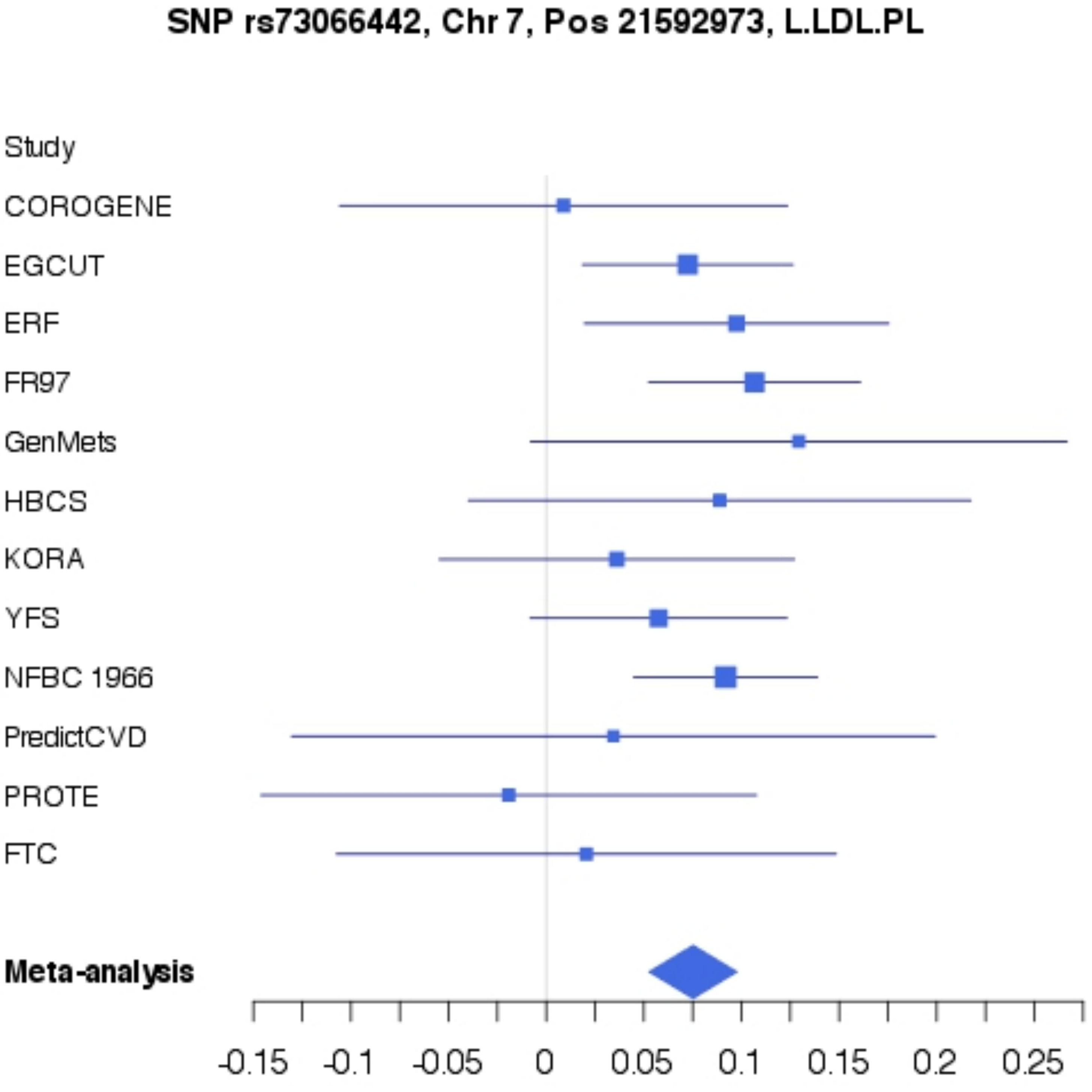
SNP rs73066442, Chr 7, Pos 21592973, L.LDL.PL
Study
COROGENE
EGCUT
ERF
FR97
GenMets
HBCS
KORA
YFS
NFBC 1966
PredictCVD
PROTE
FTC
Meta-analysis
-0.15
-0.1
-0.05
0
0.05
0.1
0.15
0.2
0.25

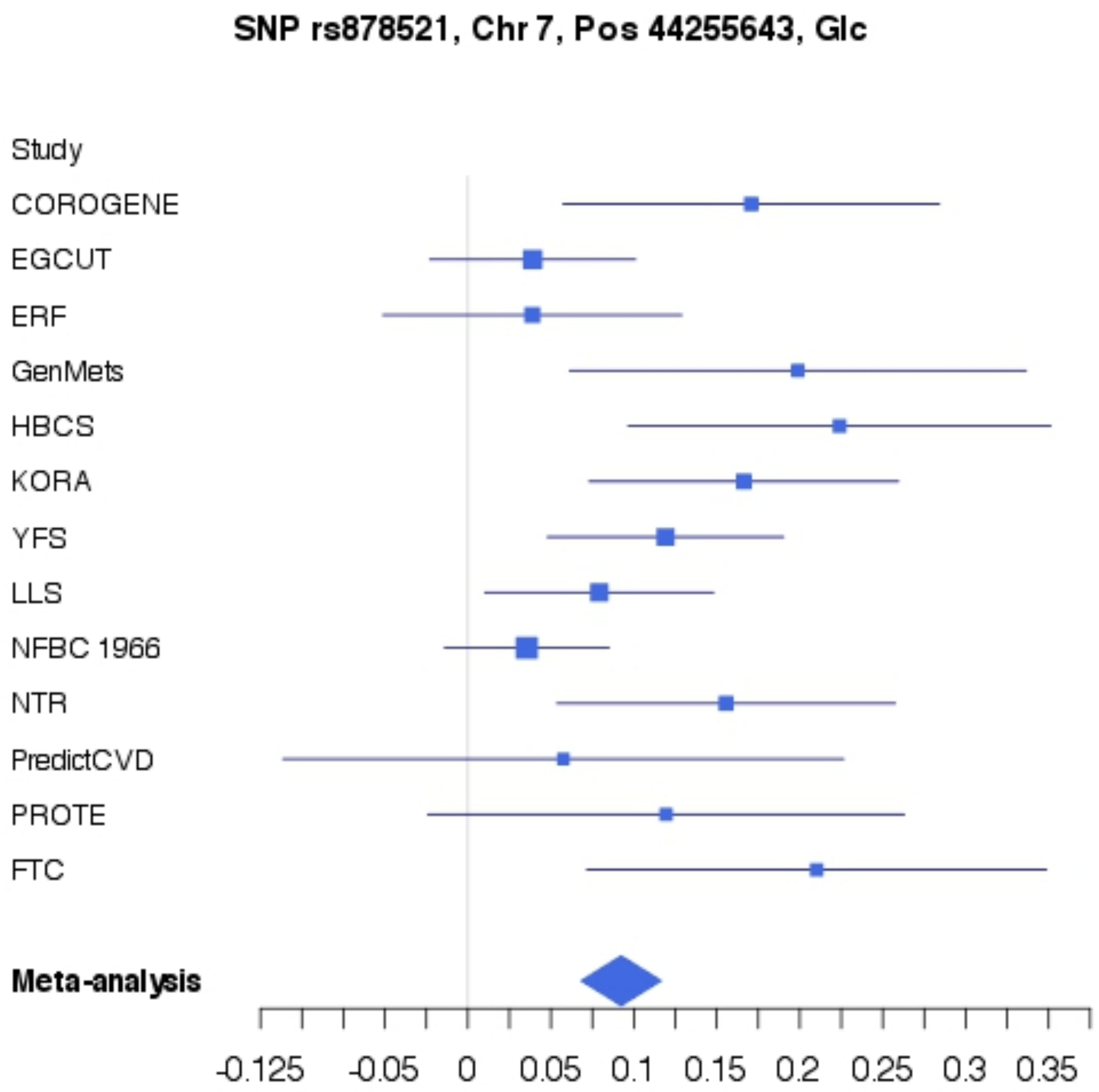
SNP rs878521, Chr 7, Pos 44255643, Glc
Study
COROGENE
EGCUT
ERF
GenMets
HBCS
KORA
YFS
LLS
NFBC 1966
NTR
PredictCVD
PROTE
FTC
Meta-analysis
-0.125
-0.05
0
0.05
0.1
0.15
0.2
0.25
0.3
0.35

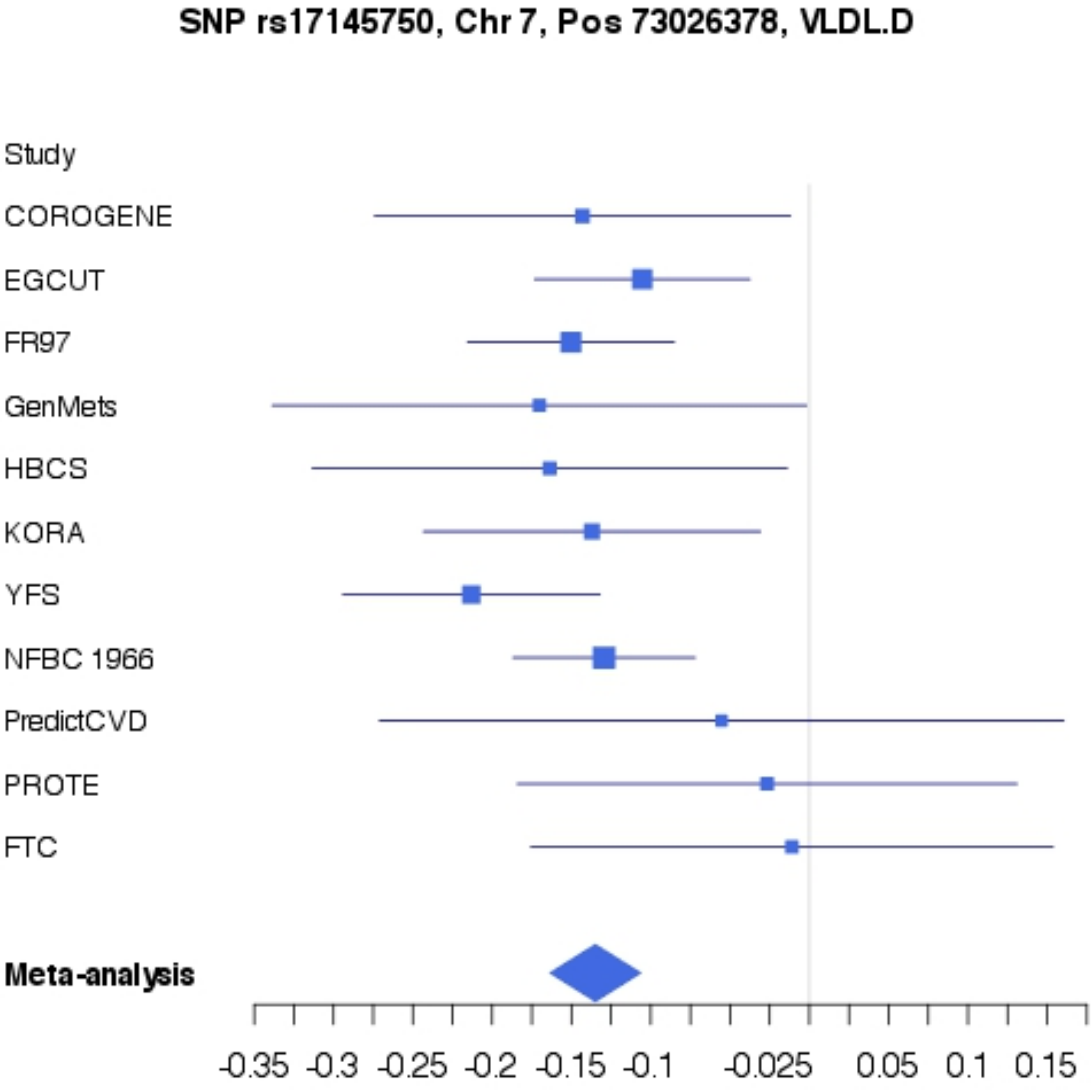
SNP rs17145750, Chr 7, Pos 73026378, VLDL.D
Study
COROGENE
EGCUT
FR97
GenMets
HBCS
KORA
YFS
NFBC 1966
PredictCVD
PROTE
FTC
Meta-analysis
-0.35
-0.3
-0.25
-0.2
-0.15
-0.1
-0.025
0.05
0.1
0.15

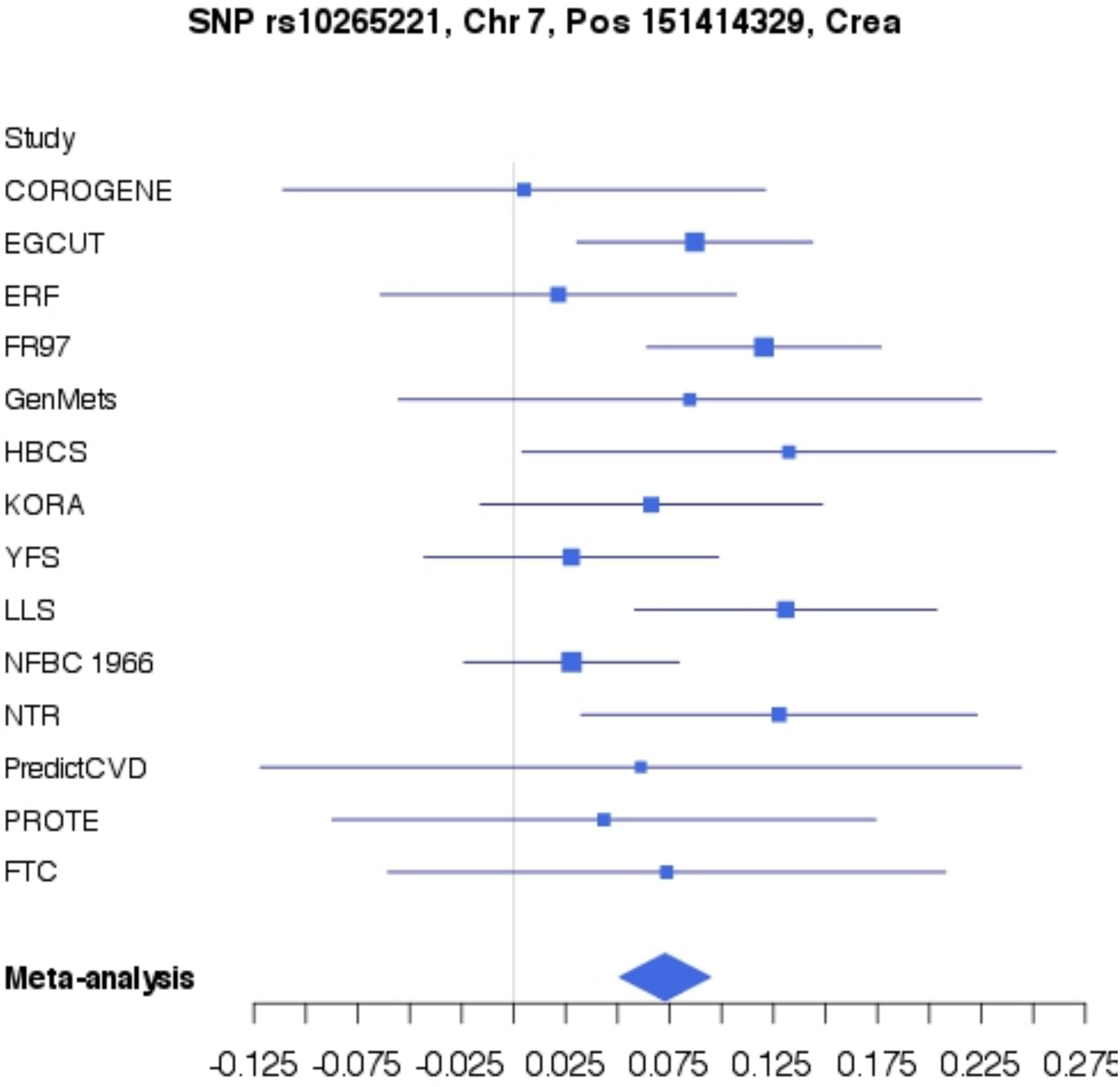

SNP rs10265221, Chr 7, Pos 151414329, Crea
Study
COROGENE
EGCUT
ERF
FR97
GenMets
HBCS
KORA
YFS
LLS
NFBC 1966
NTR
PredictCVD
PROTE
FTC
Meta-analysis
-0.125
-0.075
-0.025
0.025
0.075
0.125
0.175
0.225
0.275

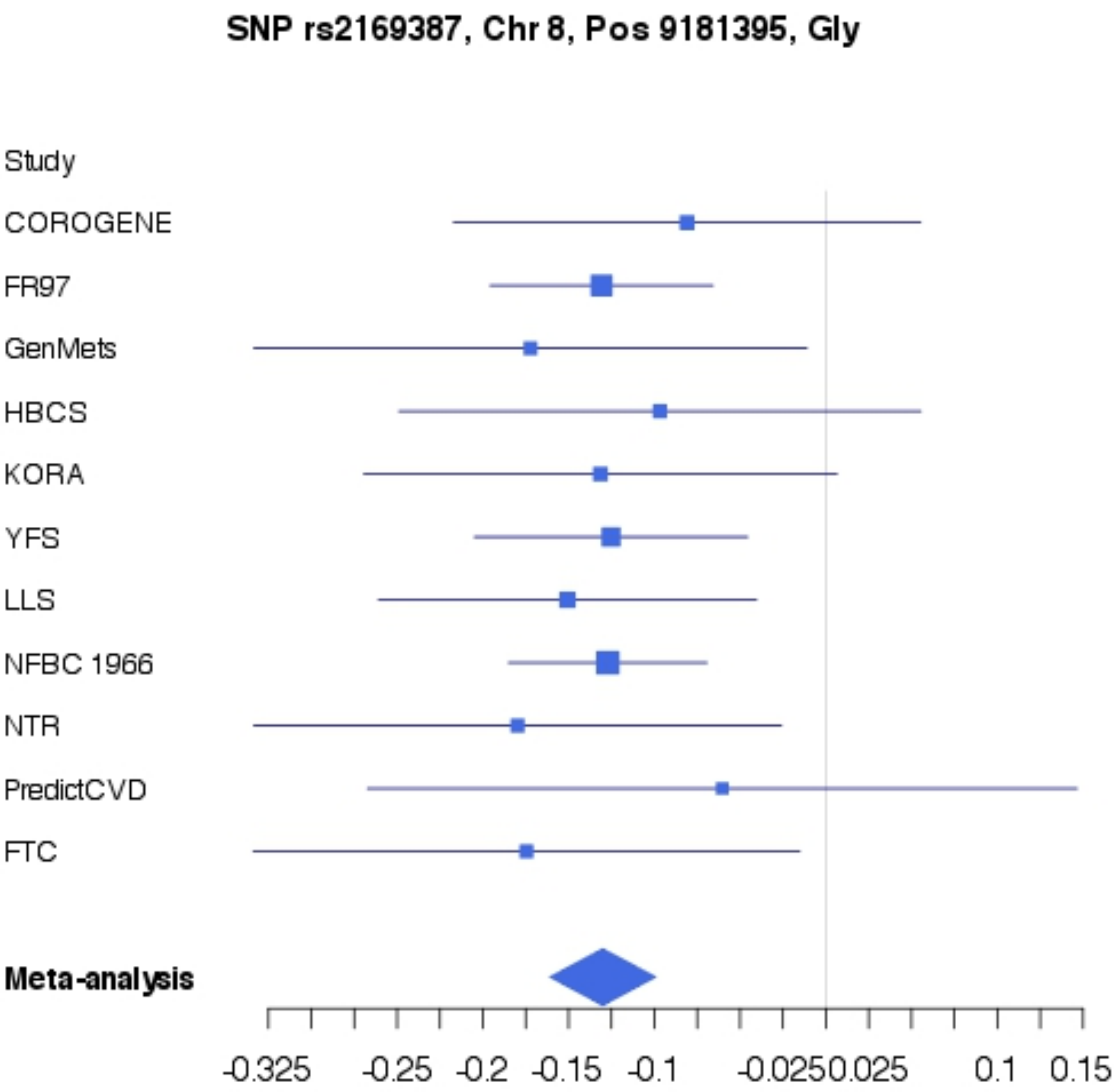

SNP rs2169387, Chr 8, Pos 9181395, Gly
Study
COROGENE
FR97
GenMets
HBCS
KORA
YFS
LLS
NFBC 1966
NTR
PredictCVD
FTC
Meta-analysis
-0.325
-0.25
-0.2
-0.15
-0.1
-0.025
0.025
0.1
0.15

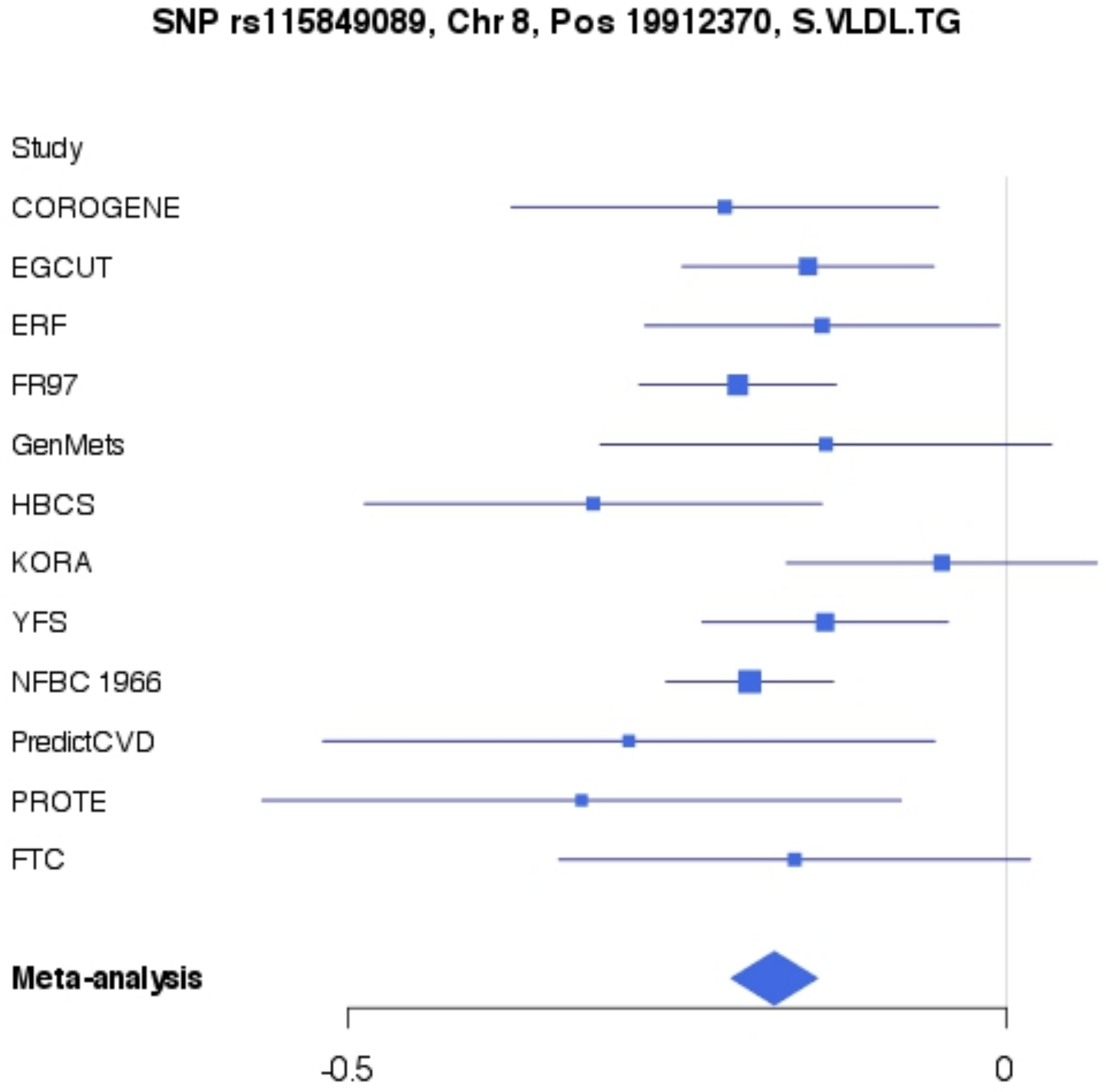
SNP rs115849089, Chr 8, Pos 19912370, S.VLDL.TG
Study
COROGENE
EGCUT
ERF
FR97
GenMets
HBCS
KORA
YFS
NFBC 1966
PredictCVD
PROTE
FTC
Meta-analysis
-0.5
0

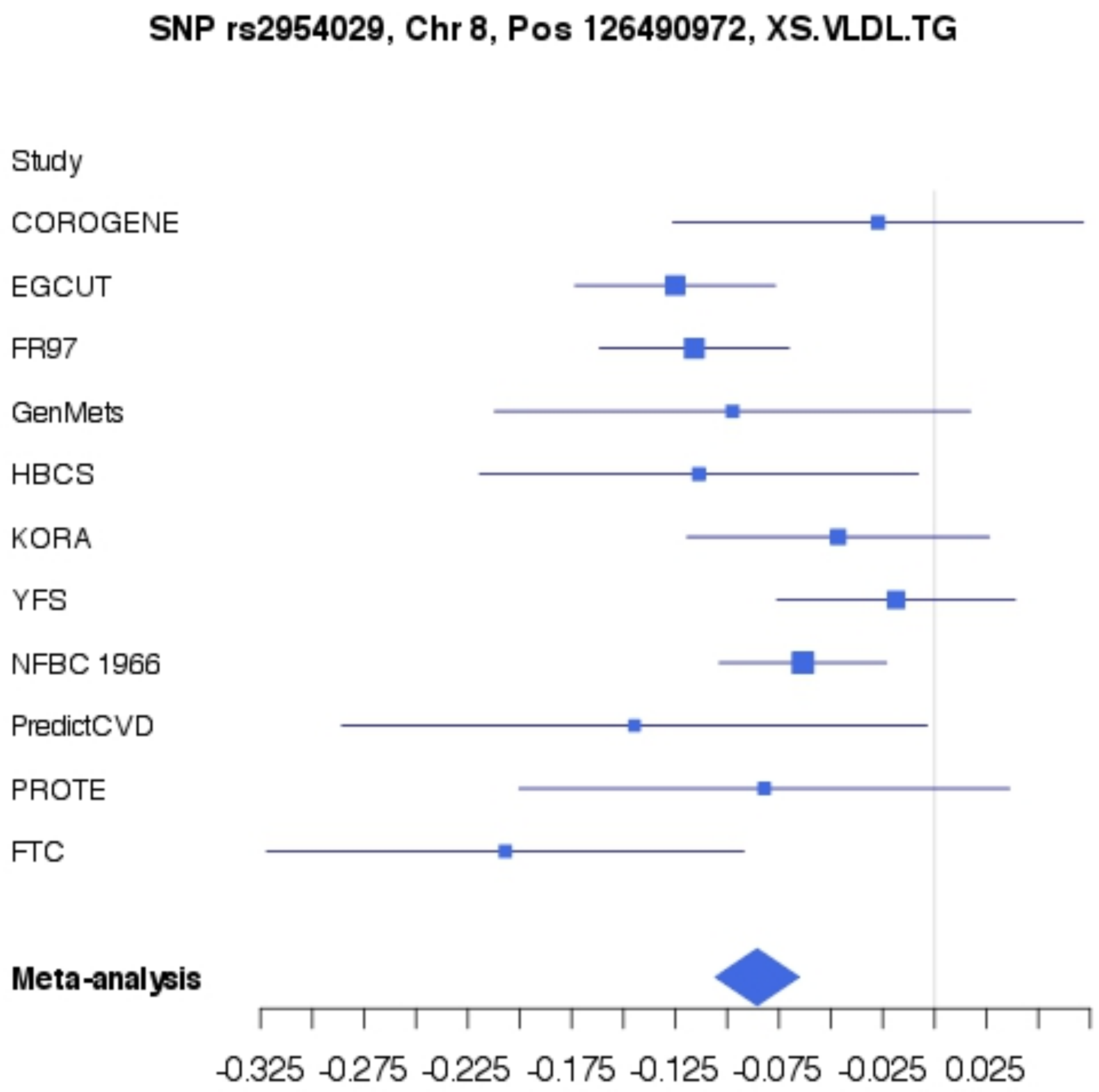
SNP rs2954029, Chr 8, Pos 126490972, XS.VLDL.TG
Study
COROGENE
EGCUT
FR97
GenMets
HBCS
KORA
YFS
NFBC 1966
PredictCVD
PROTE
FTC
Meta-analysis
-0.325
-0.275
-0.225
-0.175
-0.125
-0.075
-0.025
0.025

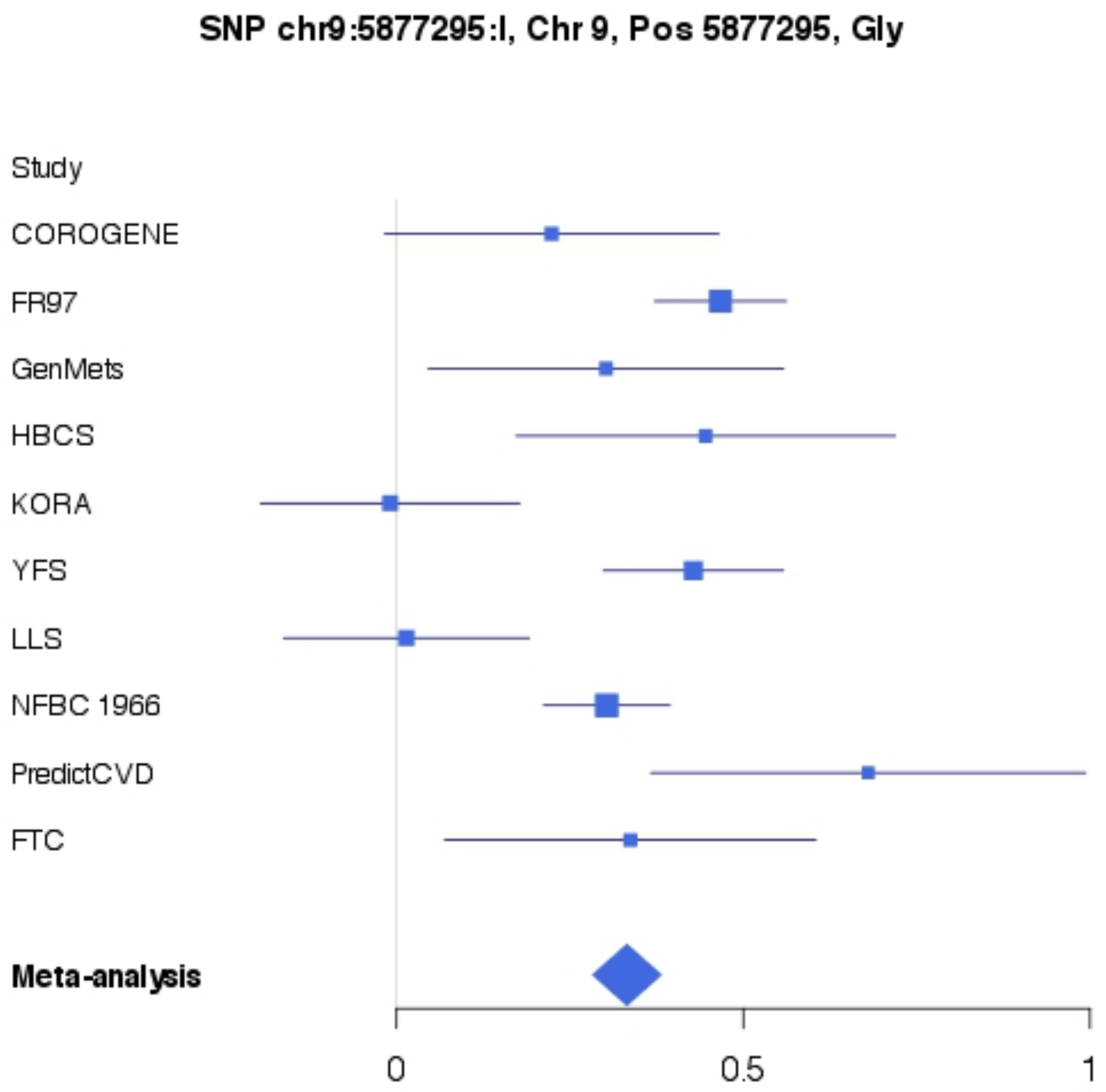
SNP chr9:5877295:I, Chr 9, Pos 5877295, Gly
Study
COROGENE
FR97
GenMets
HBCS
KORA
YFS
LLS
NFBC 1966
PredictCVD
FTC
Meta-analysis
0
0.5
1

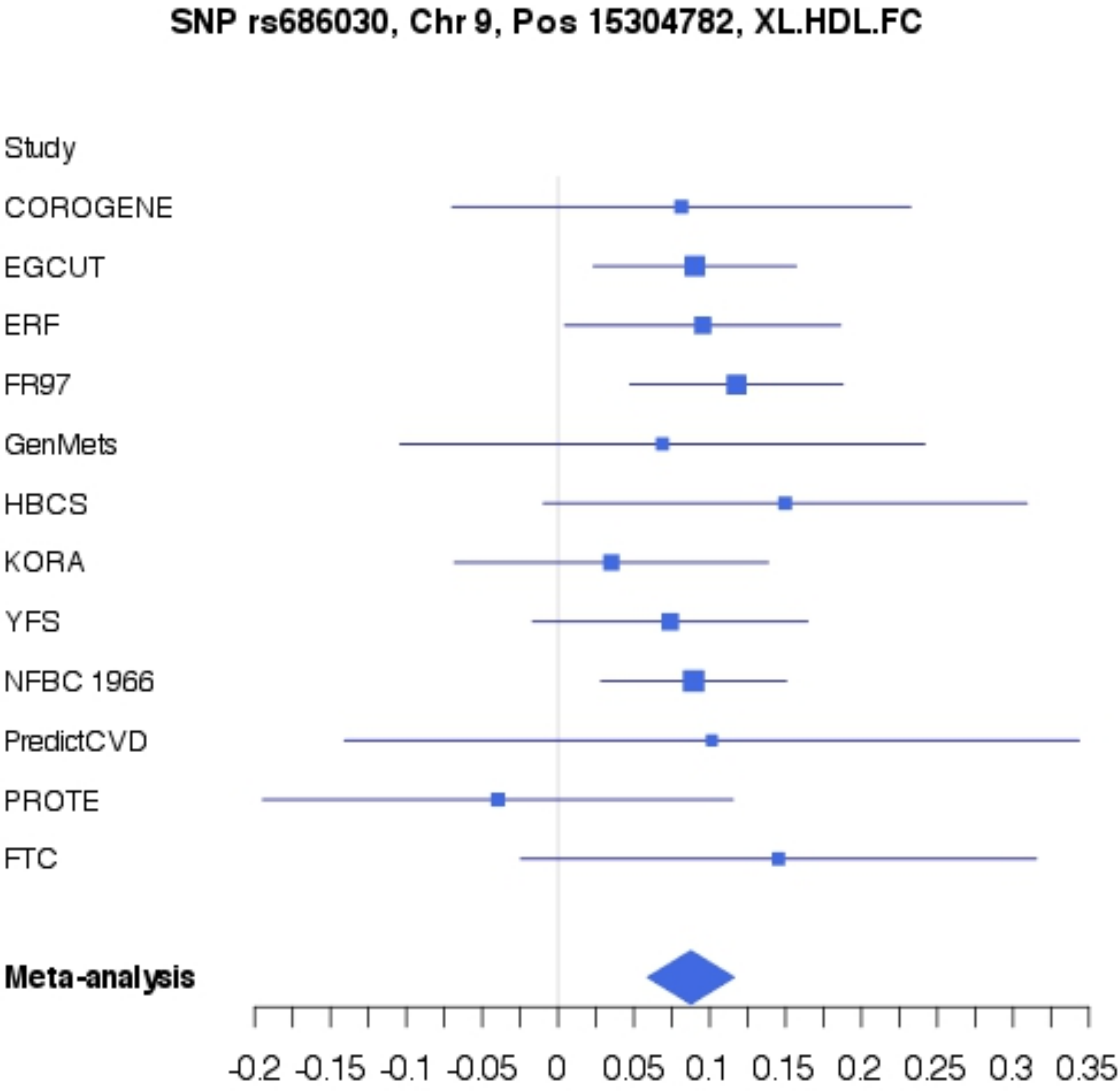

SNP rs686030, Chr 9, Pos 15304782, XL.HDL.FC
Study
COROGENE
EGCUT
ERF
FR97
GenMets
HBCS
KORA
YFS
NFBC 1966
PredictCVD
PROTE
FTC
Meta-analysis
-0.2
-0.15
-0.1
-0.05
0
0.05
0.1
0.15
0.2
0.25
0.3
0.35

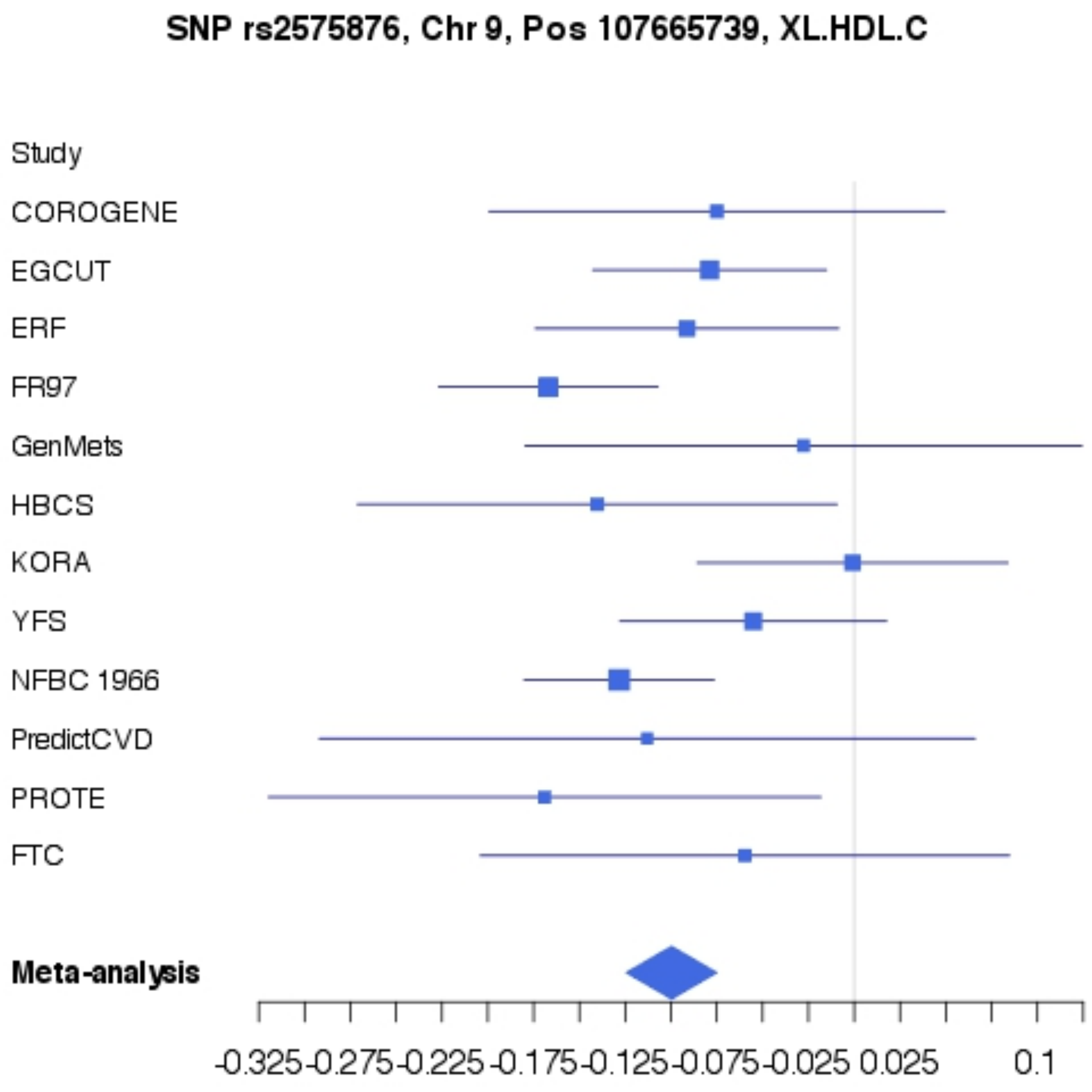
SNP rs2575876, Chr 9, Pos 107665739, XL.HDL.C
Study
COROGENE
EGCUT
ERF
FR97
GenMets
HBCS
KORA
YFS
NFBC 1966
PredictCVD
PROTE
FTC
Meta-analysis
-0.325
-0.275
-0.225
-0.175
-0.125
-0.075
-0.025
0.025
0.1

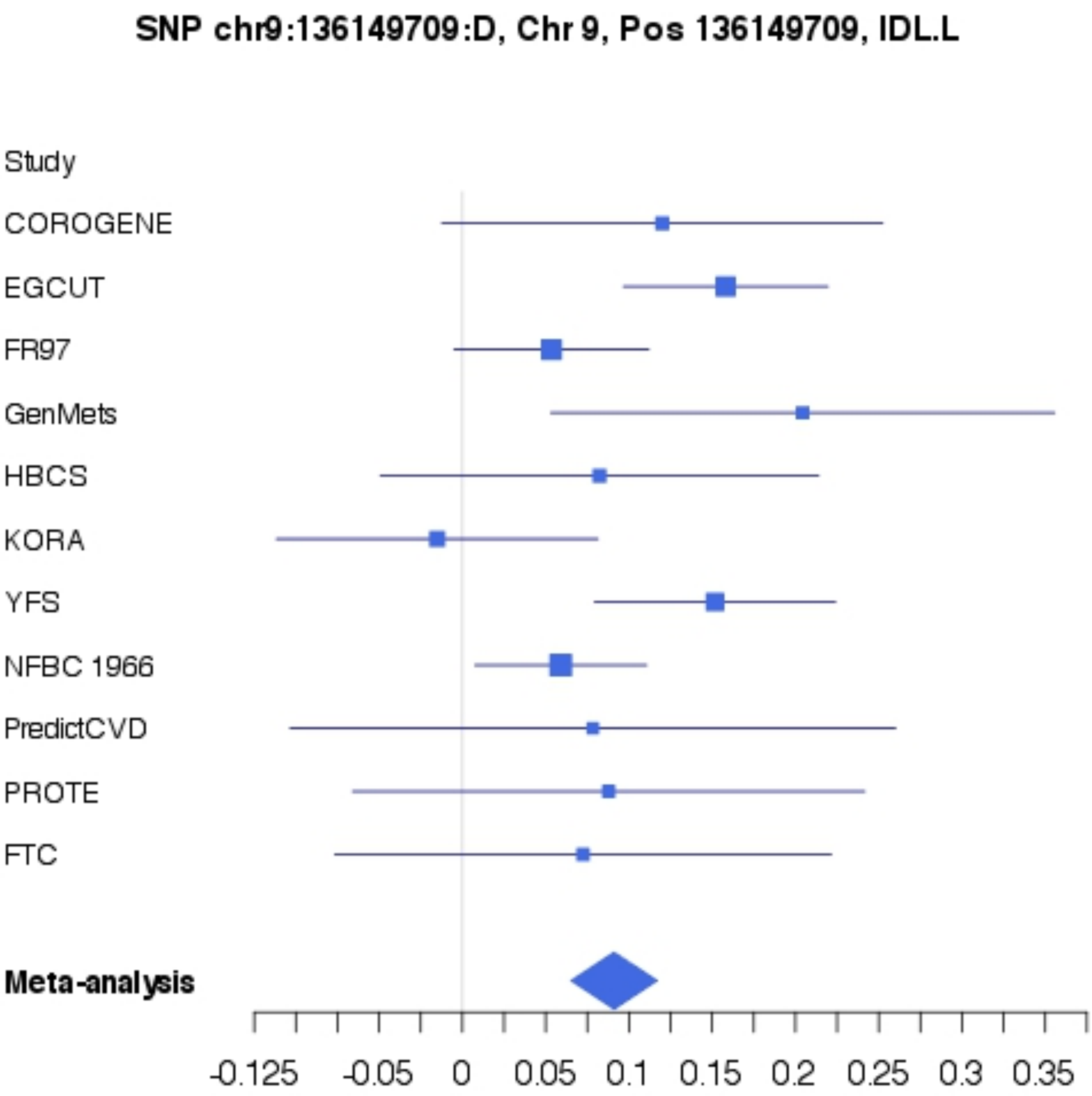
SNP chr9:136149709:D, Chr 9, Pos 136149709, IDL.L
Study
COROGENE
EGCUT
FR97
GenMets
HBCS
KORA
YFS
NFBC 1966
PredictCVD
PROTE
FTC
Meta-analysis
-0.125
-0.05
0
0.05
0.1
0.15
0.2
0.25
0.3
0.35

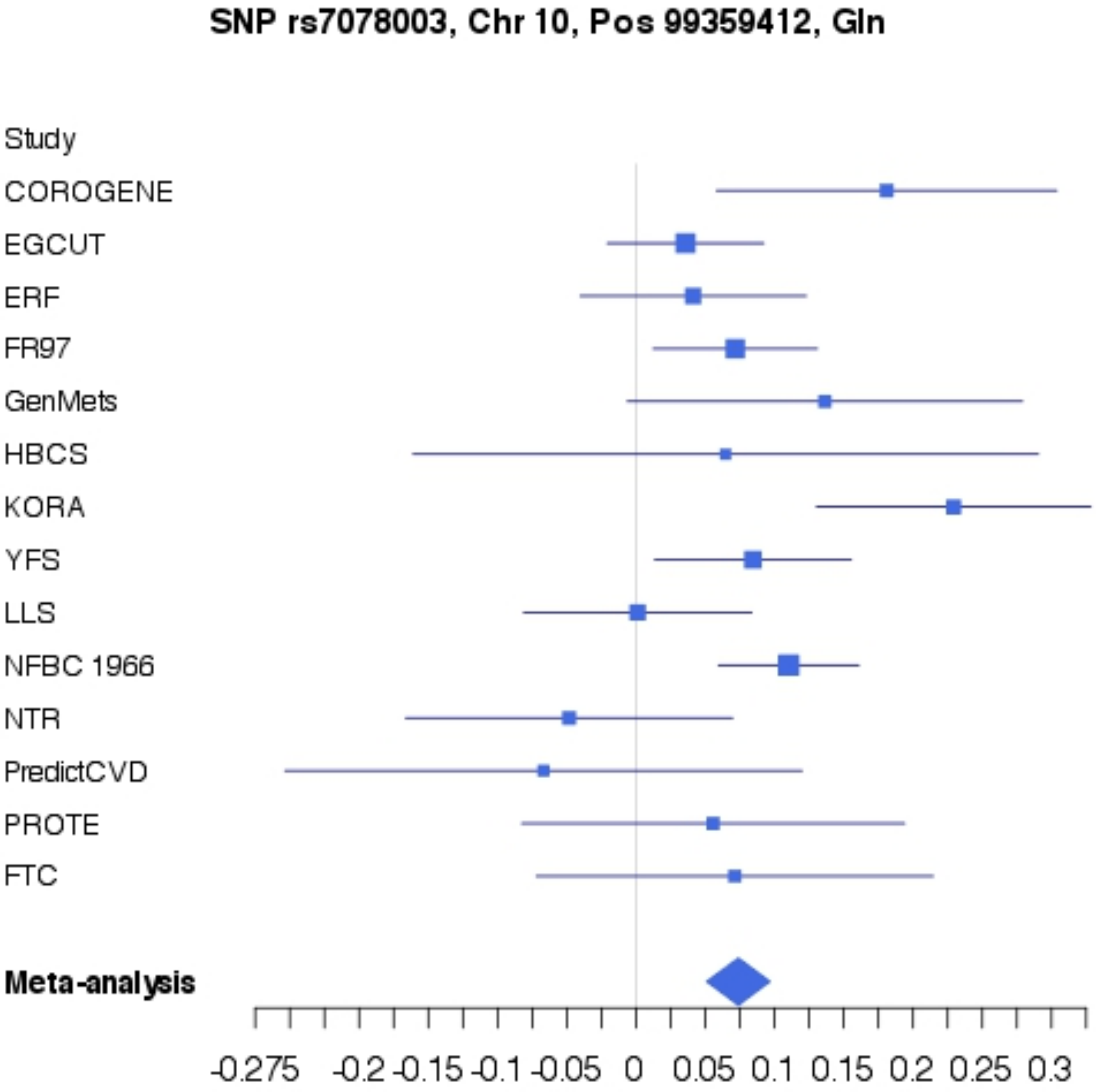

SNP rs7078003, Chr 10, Pos 99359412, Gln
Study
COROGENE
EGCUT
ERF
FR97
GenMets
HBCS
KORA
YFS
LLS
NFBC 1966
NTR
PredictCVD
PROTE
FTC
Meta-analysis
-0.275
-0.2
-0.15
-0.1
-0.05
0
0.05
0.1
0.15
0.2
0.25
0.3

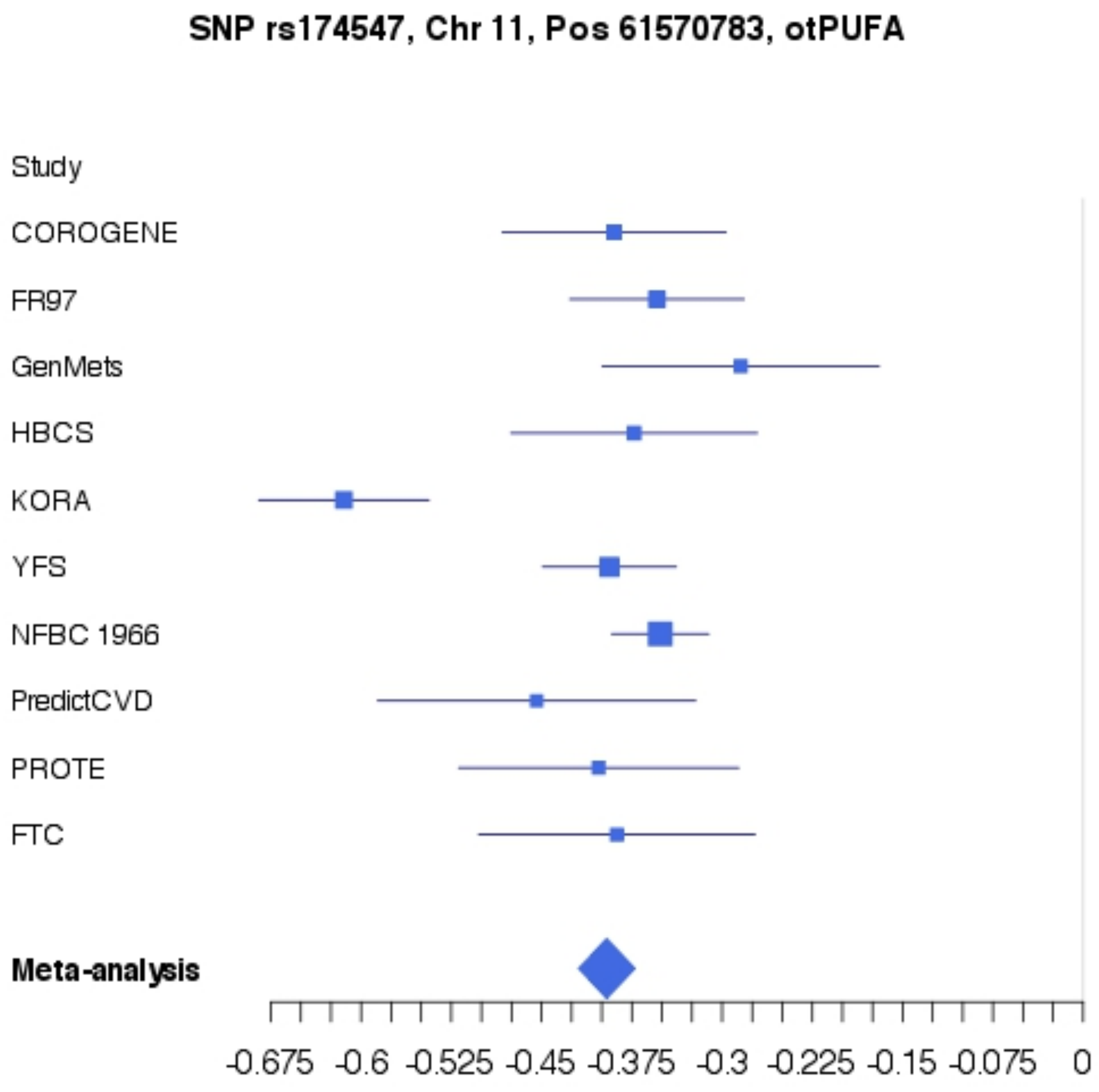

SNP rs174547, Chr 11, Pos 61570783, otPUFA
Study
COROGENE
FR97
GenMets
HBCS
KORA
YFS
NFBC 1966
PredictCVD
PROTE
FTC
Meta-analysis
-0.675
-0.6
-0.525
-0.45
-0.375
-0.3
-0.225
-0.15
-0.075
0

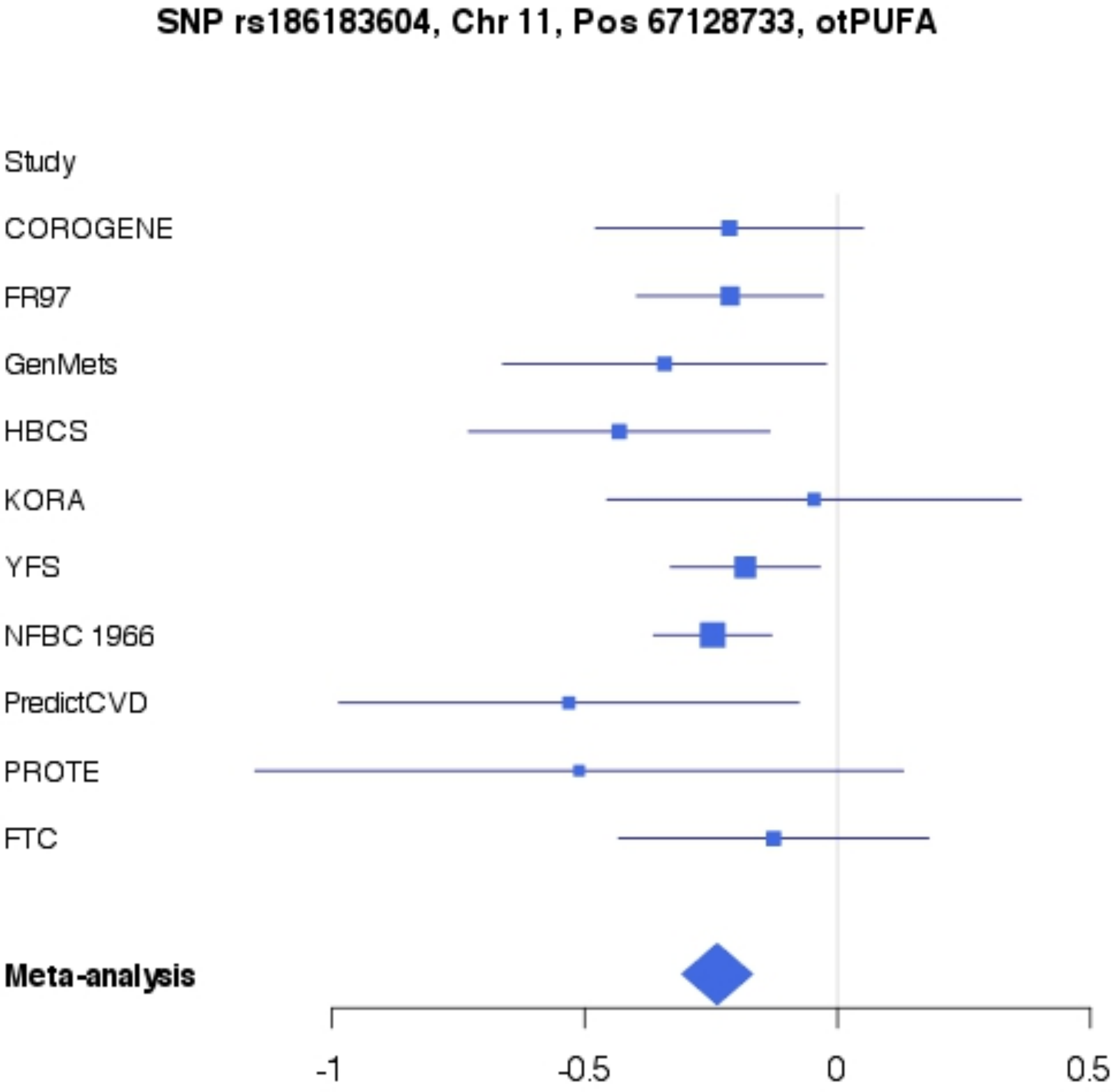
SNP rs186183604, Chr 11, Pos 67128733, otPUFA
Study
COROGENE
FR97
GenMets
HBCS
KORA
YFS
NFBC 1966
PredictCVD
PROTE
FTC
Meta-analysis
-1
-0.5
0
0.5

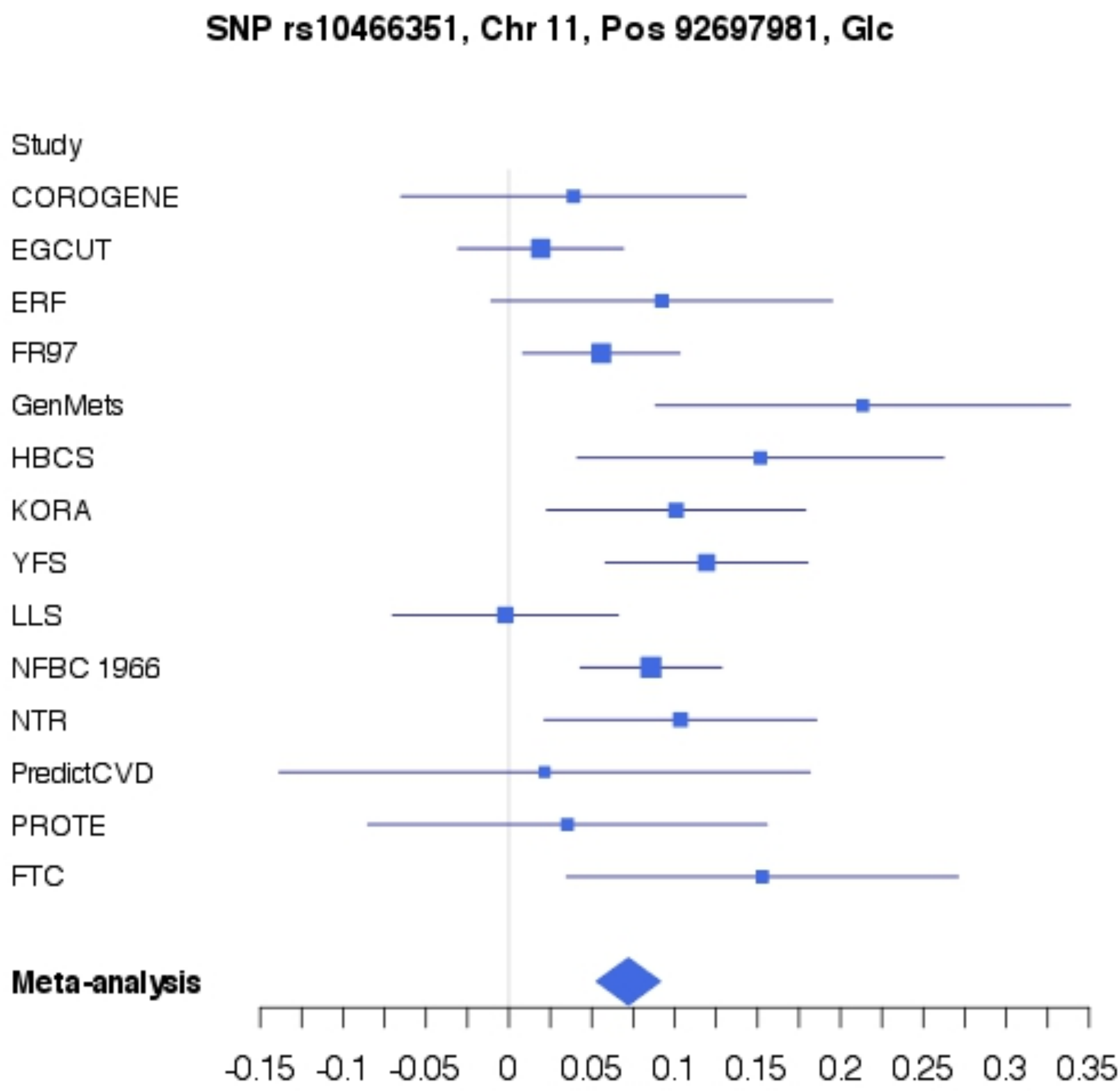
SNP rs10466351, Chr 11, Pos 92697981, Glc
Study
COROGENE
EGCUT
ERF
FR97
GenMets
HBCS
KORA
YFS
LLS
NFBC 1966
NTR
PredictCVD
PROTE
FTC
Meta-analysis
-0.15
-0.1
-0.05
0
0.05
0.1
0.15
0.2
0.25
0.3
0.35

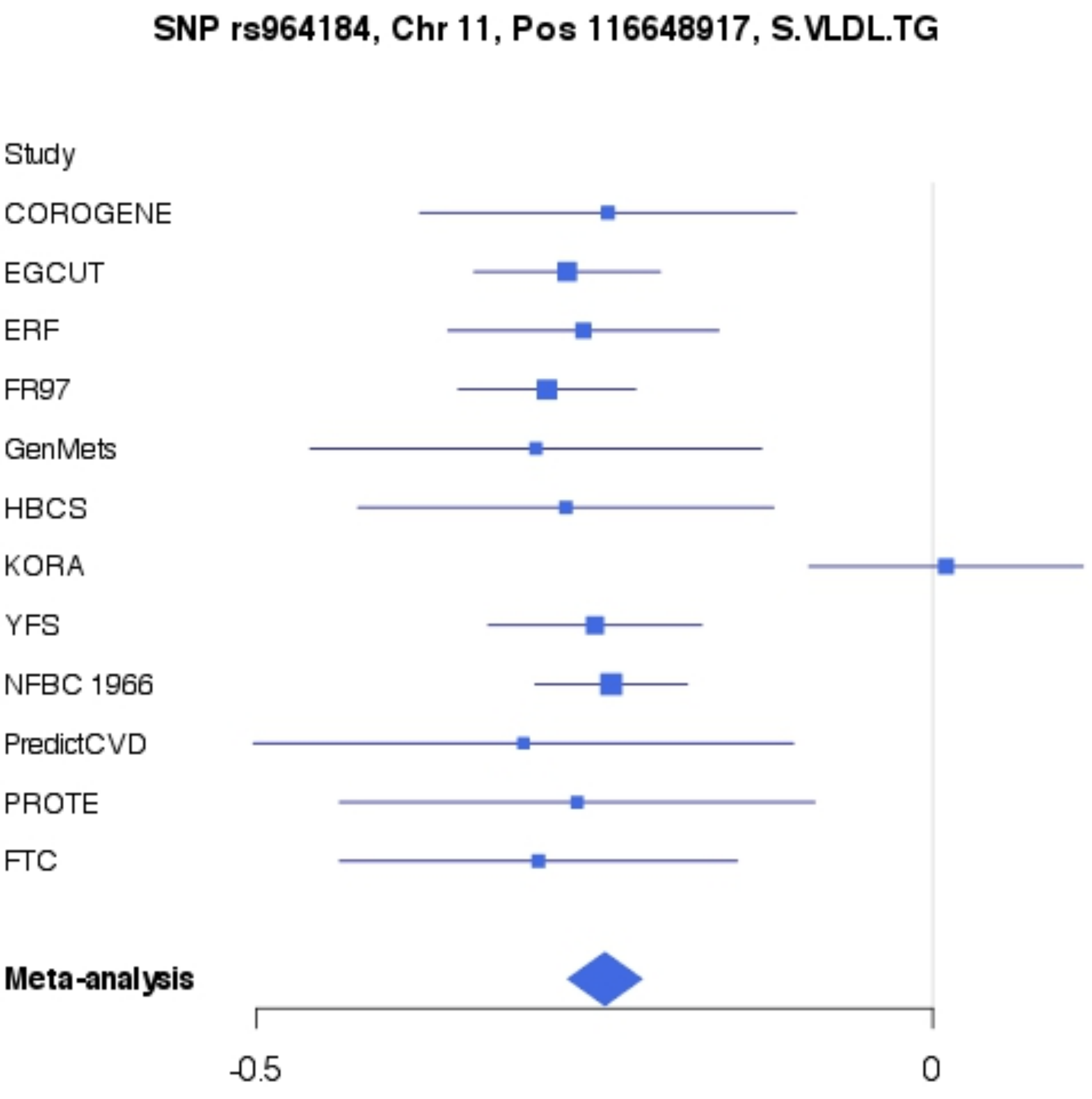

SNP rs964184, Chr 11, Pos 116648917, S.VLDL.TG
Study
COROGENE
EGCUT
ERF
FR97
GenMets
HBCS
KORA
YFS
NFBC 1966
PredictCVD
PROTE
FTC
Meta-analysis
-0.5
0

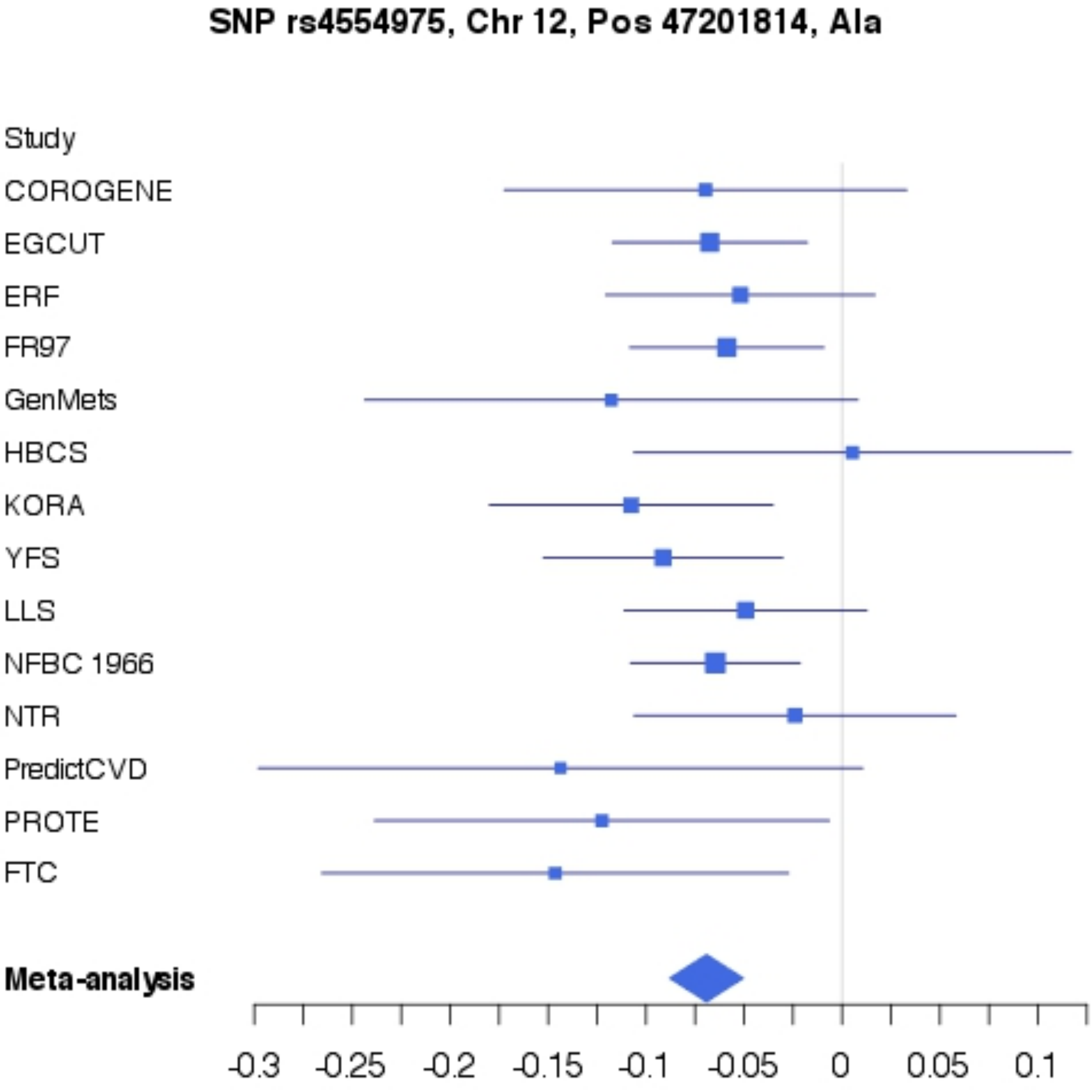
SNP rs4554975, Chr 12, Pos 47201814, Ala
Study
COROGENE
EGCUT
ERF
FR97
GenMets
HBCS
KORA
YFS
LLS
NFBC 1966
NTR
PredictCVD
PROTE
FTC
Meta-analysis
-0.3
-0.25
-0.2
-0.15
-0.1
-0.05
0
0.05
0.1

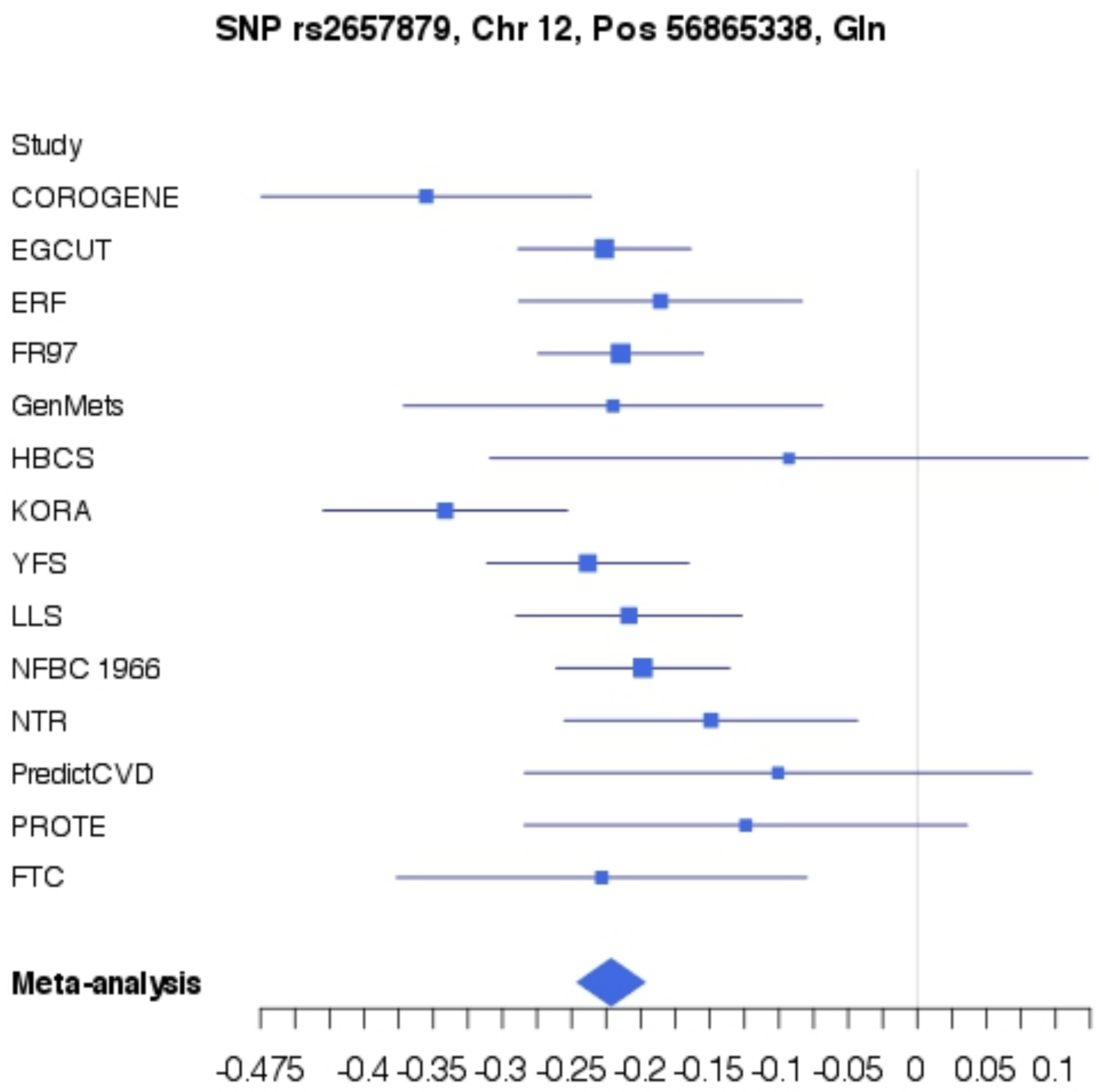
SNP rs2657879, Chr 12, Pos 56865338, Gln
Study
COROGENE
EGCUT
ERF
FR97
GenMets
HBCS
KORA
YFS
LLS
NFBC 1966
NTR
PredictCVD
PROTE
FTC
Meta-analysis
-0.475
-0.4
-0.35
-0.3
-0.25
-0.2
-0.15
-0.1
-0.05
0
0.05
0.1

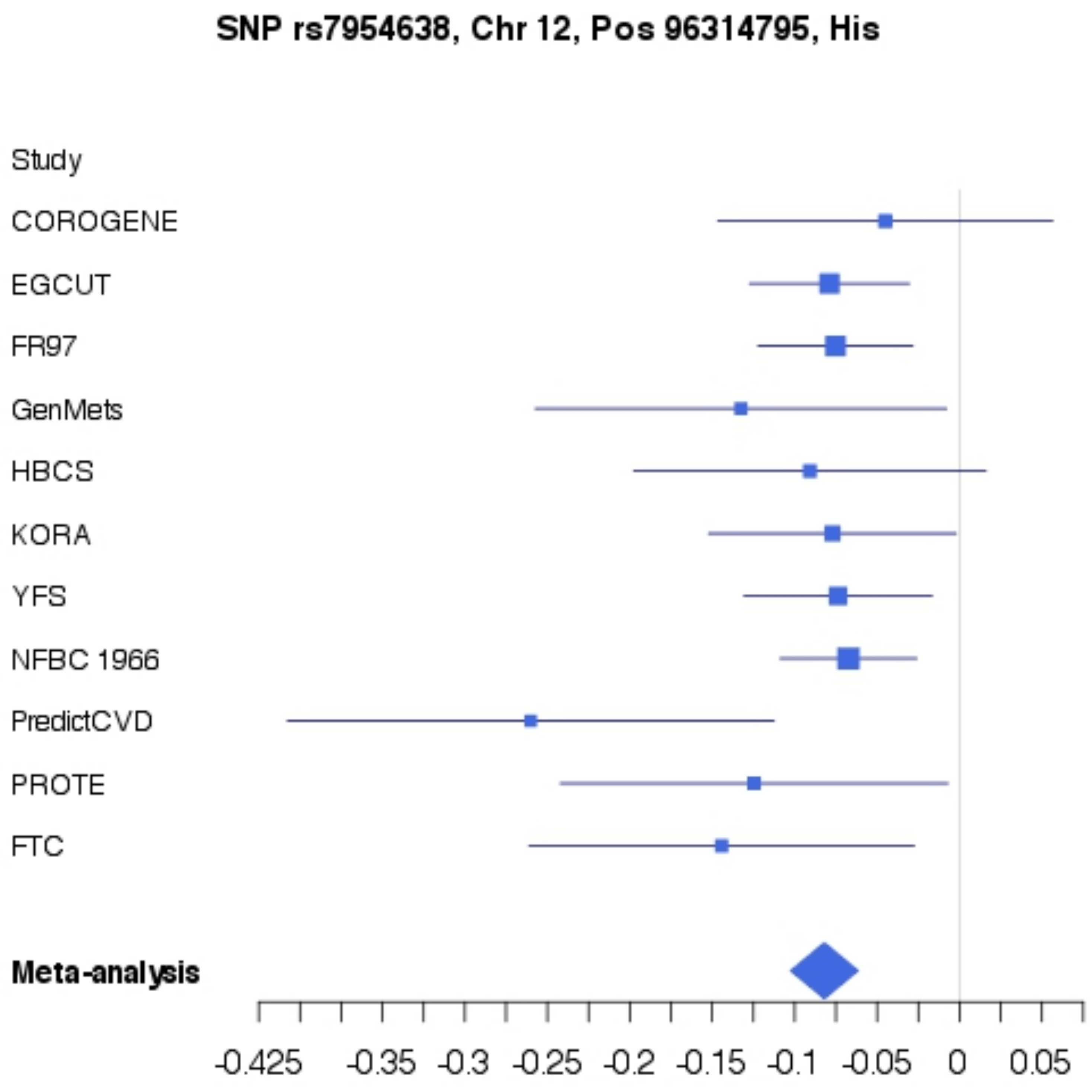

SNP rs7954638, Chr 12, Pos 96314795, His
Study
COROGENE
EGCUT
FR97
GenMets
HBCS
KORA
YFS
NFBC 1966
PredictCVD
PROTE
FTC
Meta-analysis
-0.425
-0.35
-0.3
-0.25
-0.2
-0.15
-0.1
-0.05
0
0.05

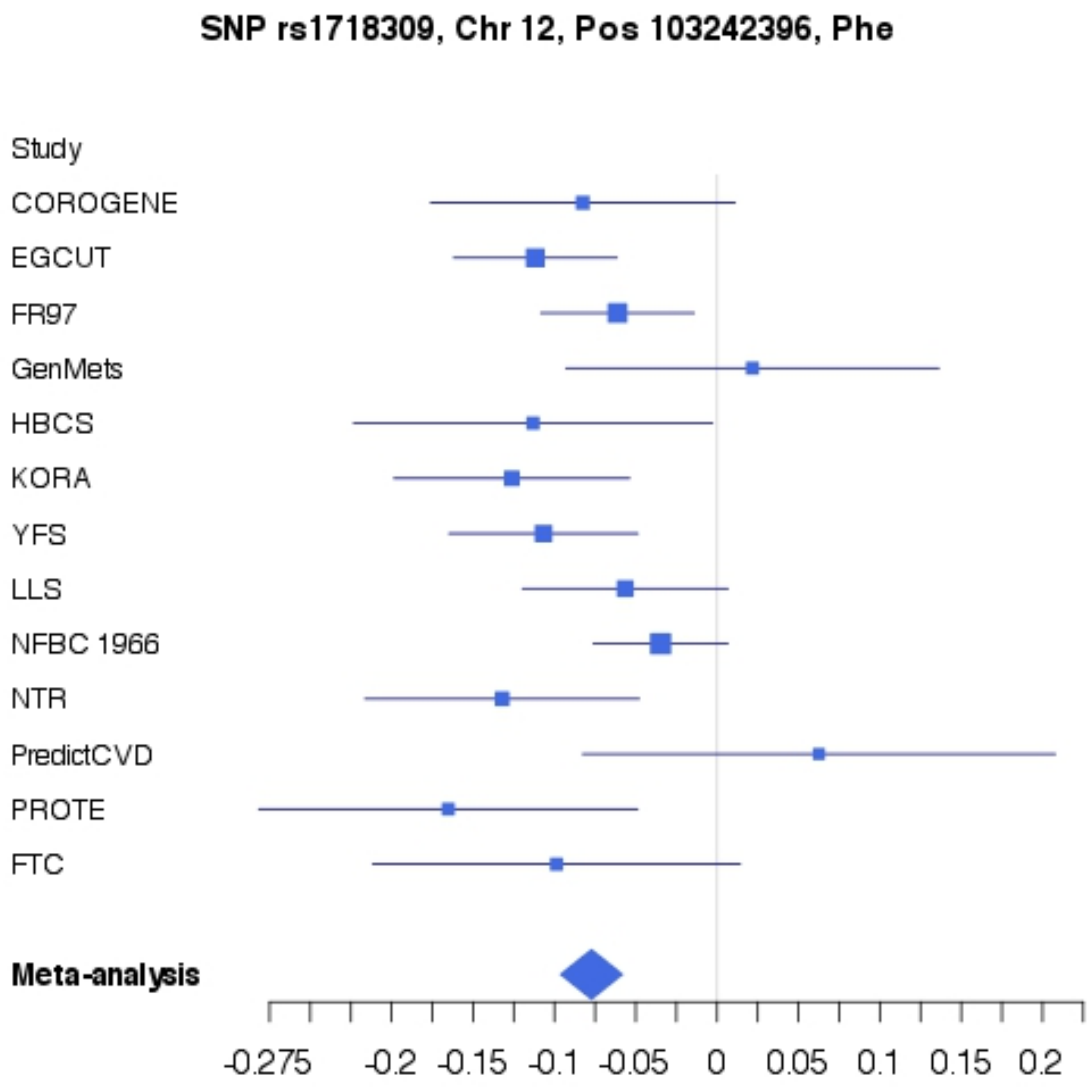
SNP rs1718309, Chr 12, Pos 103242396, Phe
Study
COROGENE
EGCUT
FR97
GenMets
HBCS
KORA
YFS
LLS
NFBC 1966
NTR
PredictCVD
PROTE
FTC
Meta-analysis
-0.275
-0.2
-0.15
-0.1
-0.05
0
0.05
0.1
0.15
0.2

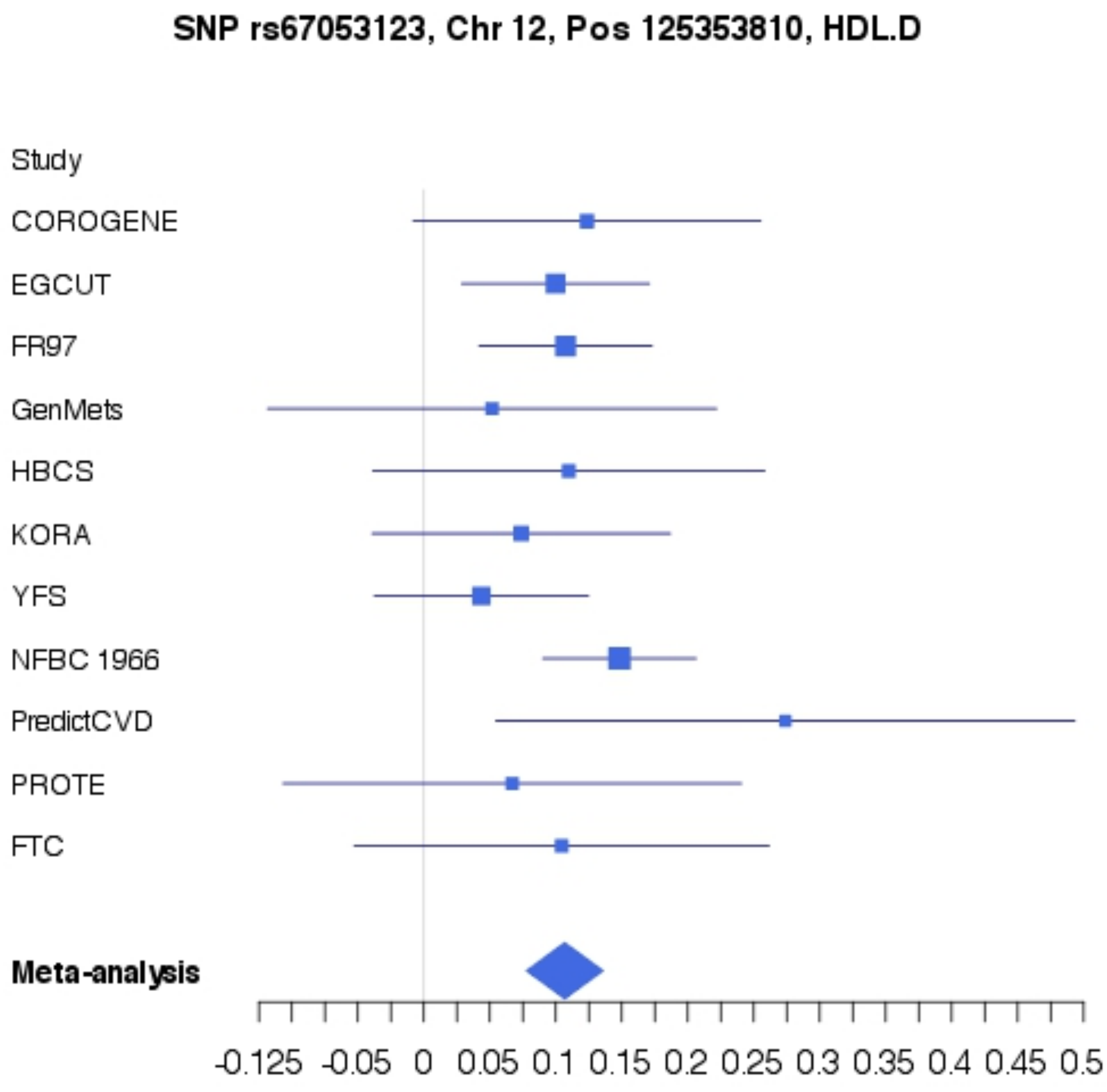
SNP rs67053123, Chr 12, Pos 125353810, HDL.D
Study
COROGENE
EGCUT
FR97
GenMets
HBCS
KORA
YFS
NFBC 1966
PredictCVD
PROTE
FTC
Meta-analysis
-0.125 -0.05 0 0.05 0.1 0.15 0.2 0.25 0.3 0.35 0.4 0.45 0.5

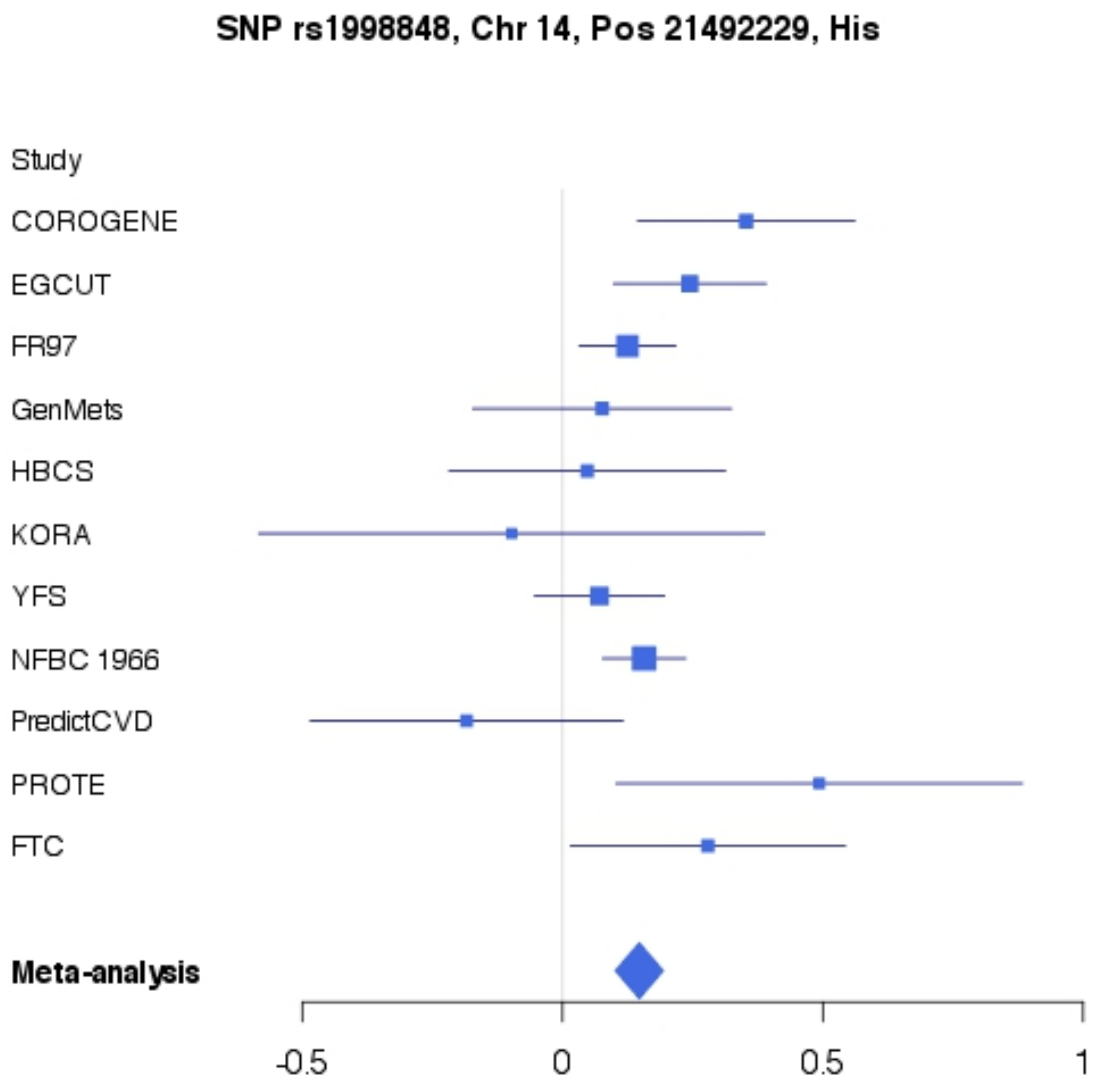
SNP rs1998848, Chr 14, Pos 21492229, His
Study
COROGENE
EGCUT
FR97
GenMets
HBCS
KORA
YFS
NFBC 1966
PredictCVD
PROTE
FTC
Meta-analysis
-0.5
0
0.5
1

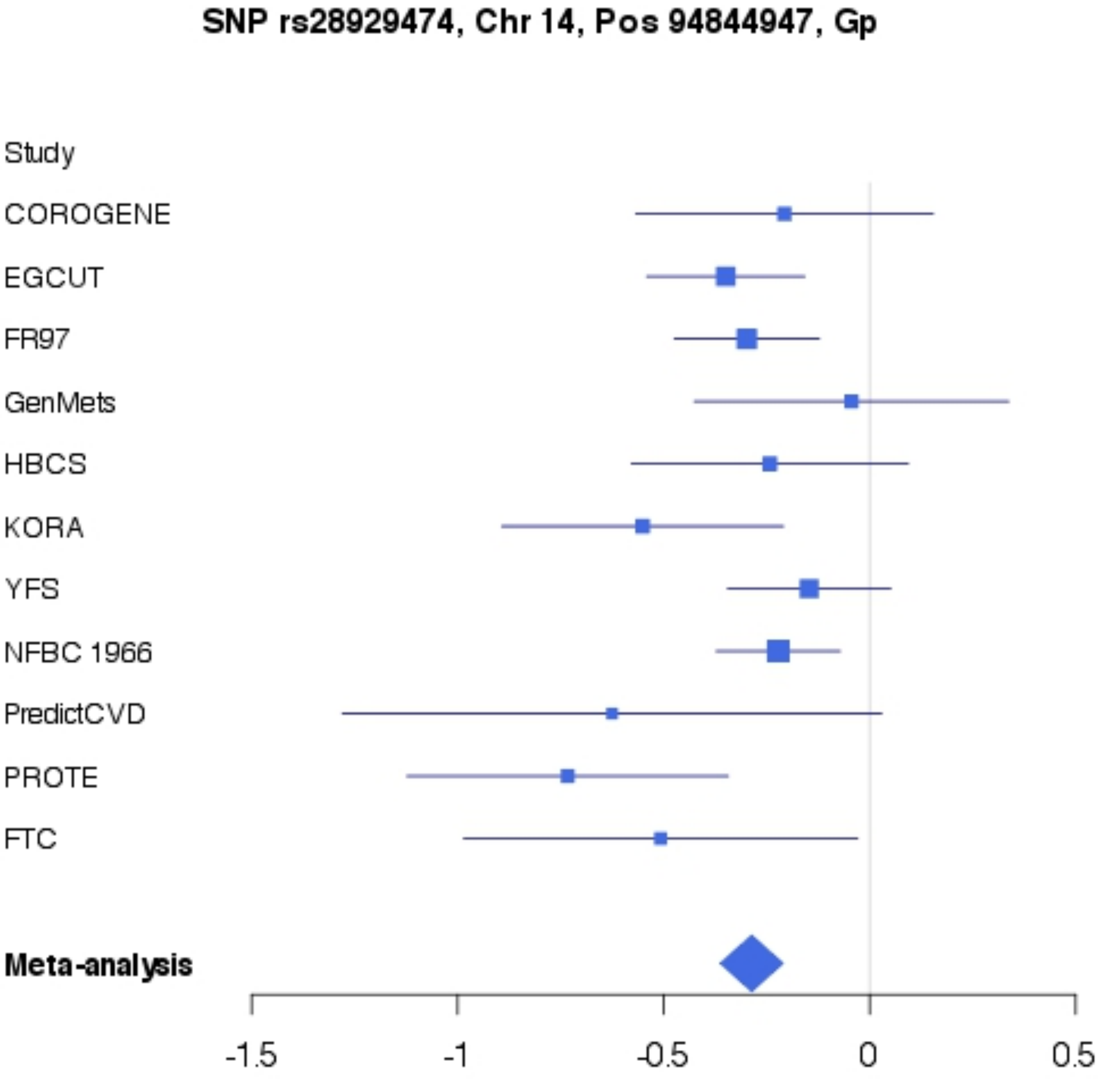
SNP rs28929474, Chr 14, Pos 94844947, Gp
Study
COROGENE
EGCUT
FR97
GenMets
HBCS
KORA
YFS
NFBC 1966
PredictCVD
PROTE
FTC
Meta-analysis
-1.5
-1
-0.5
0
0.5

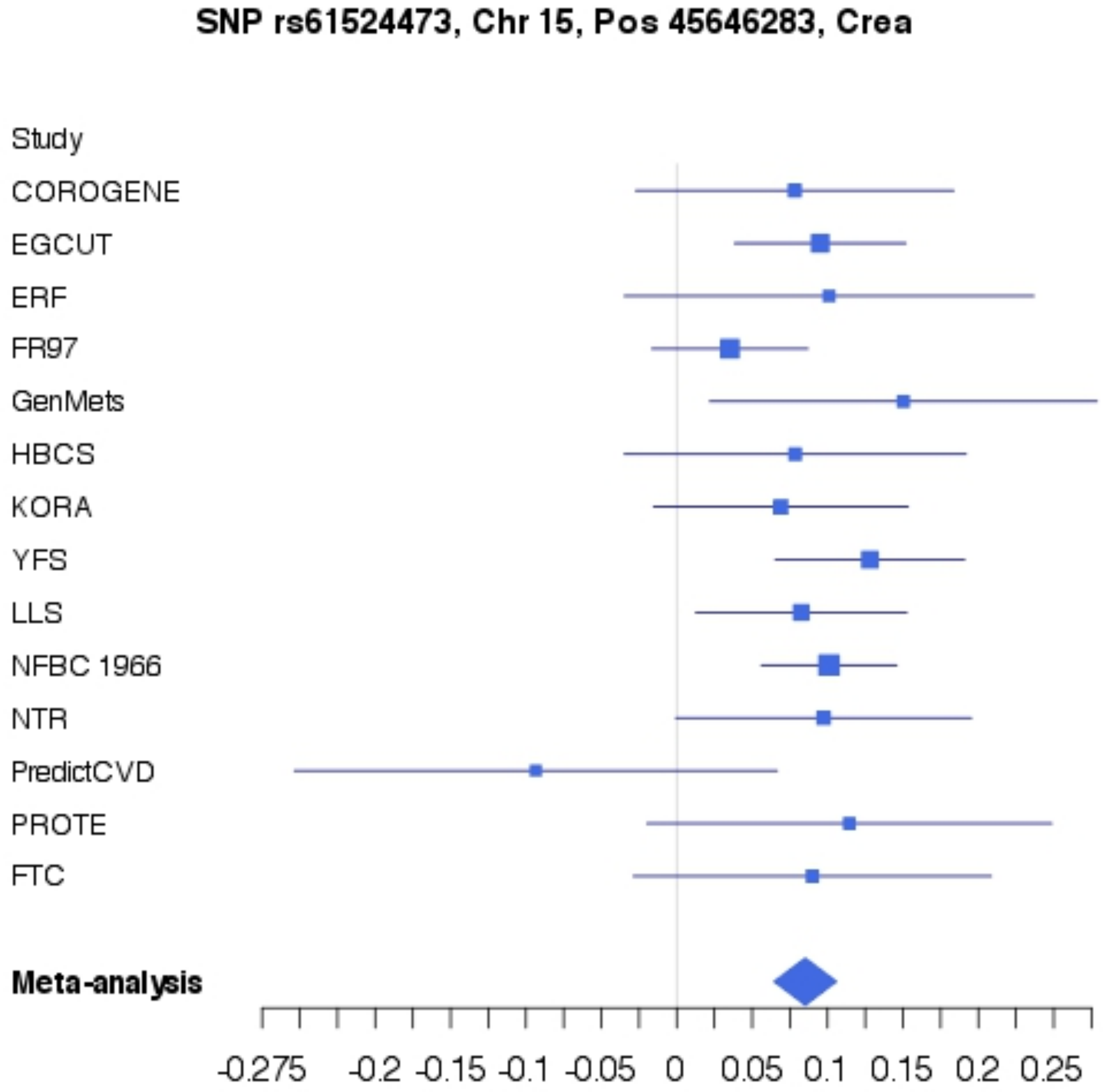
SNP rs61524473, Chr 15, Pos 45646283, Crea
Study
COROGENE
EGCUT
ERF
FR97
GenMets
HBCS
KORA
YFS
LLS
NFBC 1966
NTR
PredictCVD
PROTE
FTC
Meta-analysis
-0.275
-0.2
-0.15
-0.1
-0.05
0
0.05
0.1
0.15
0.2
0.25

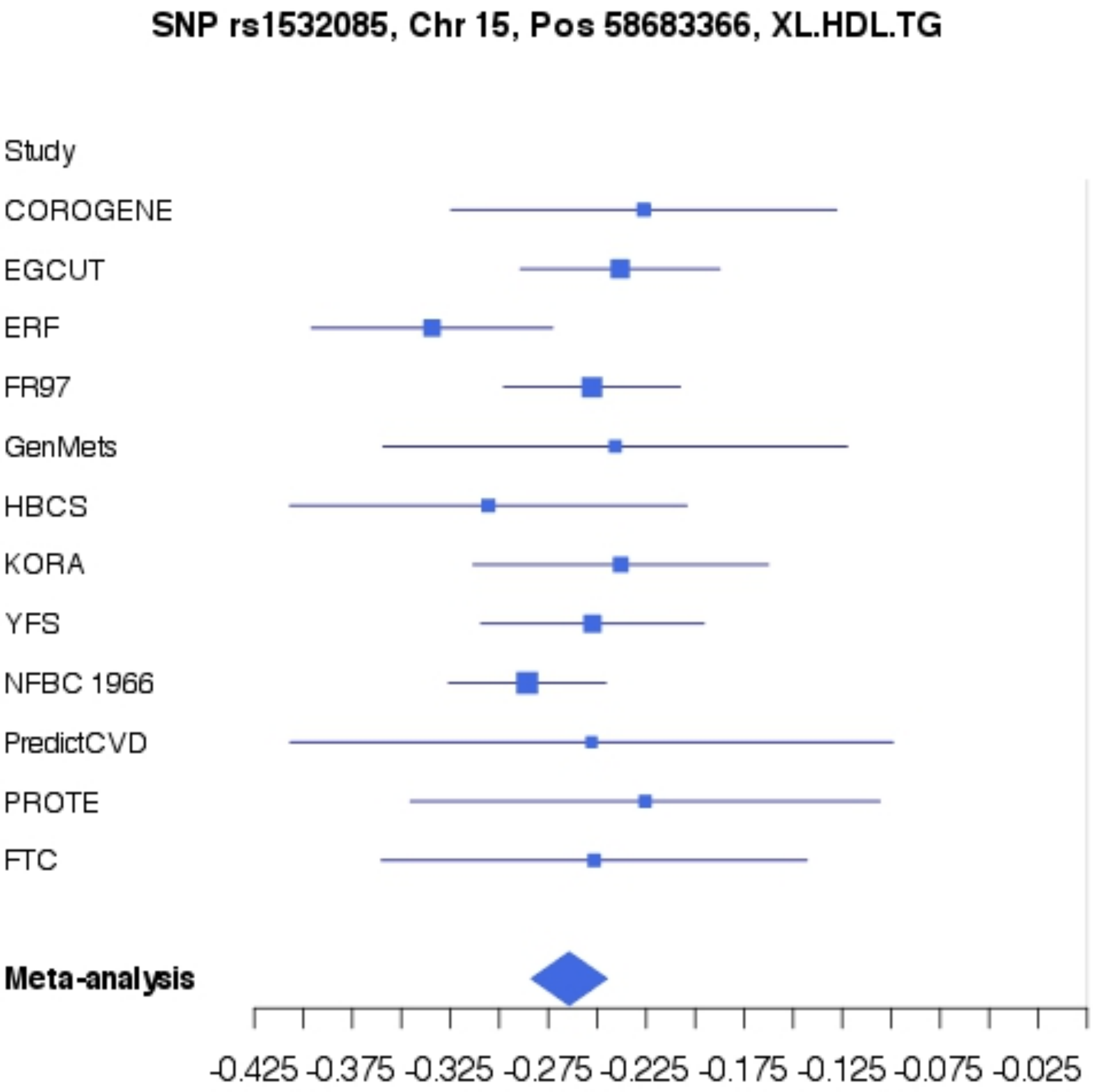
SNP rs1532085, Chr 15, Pos 58683366, XL.HDL.TG
Study
COROGENE
EGCUT
ERF
FR97
GenMets
HBCS
KORA
YFS
NFBC 1966
PredictCVD
PROTE
FTC
Meta-analysis
-0.425
-0.375
-0.325
-0.275
-0.225
-0.175
-0.125
-0.075
-0.025

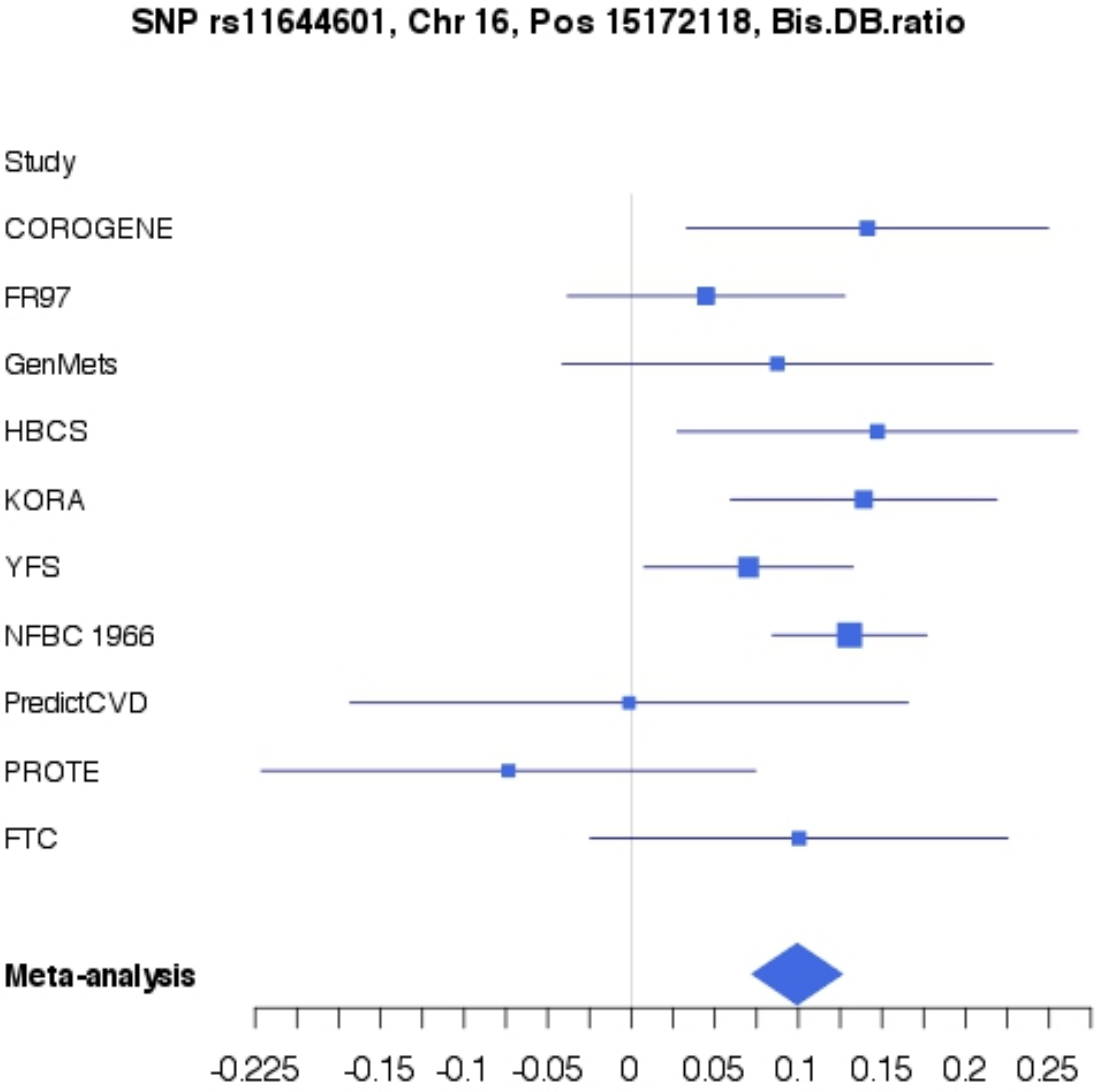
SNP rs11644601, Chr 16, Pos 15172118, Bis.DB.ratio
Study
COROGENE
FR97
GenMets
HBCS
KORA
YFS
NFBC 1966
PredictCVD
PROTE
FTC
Meta-analysis
-0.225
-0.15
-0.1
-0.05
0
0.05
0.1
0.15
0.2
0.25

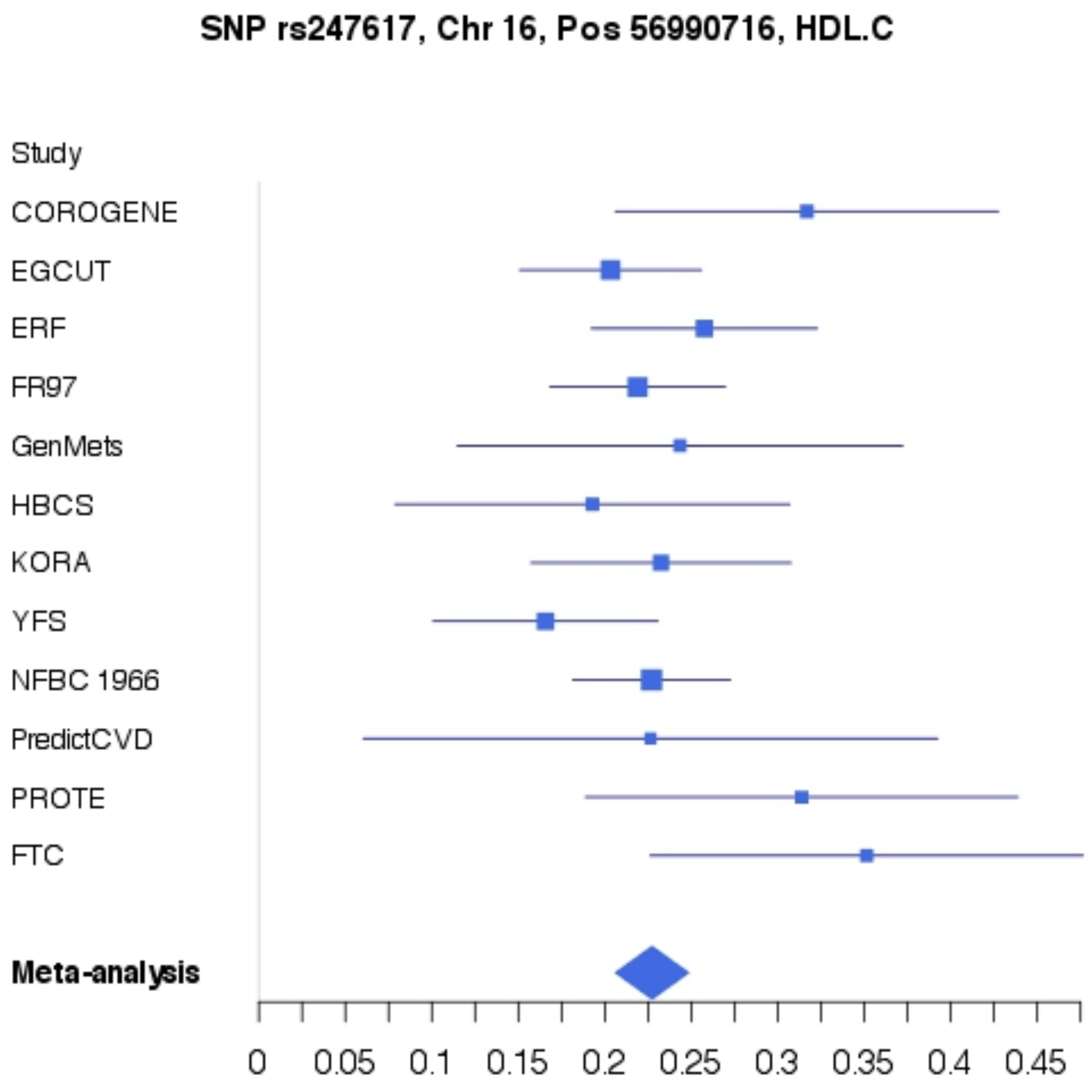

SNP rs247617, Chr 16, Pos 56990716, HDL.C
Study
COROGENE
EGCUT
ERF
FR97
GenMets
HBCS
KORA
YFS
NFBC 1966
PredictCVD
PROTE
FTC
Meta-analysis
0
0.05
0.1
0.15
0.2
0.25
0.3
0.35
0.4
0.45

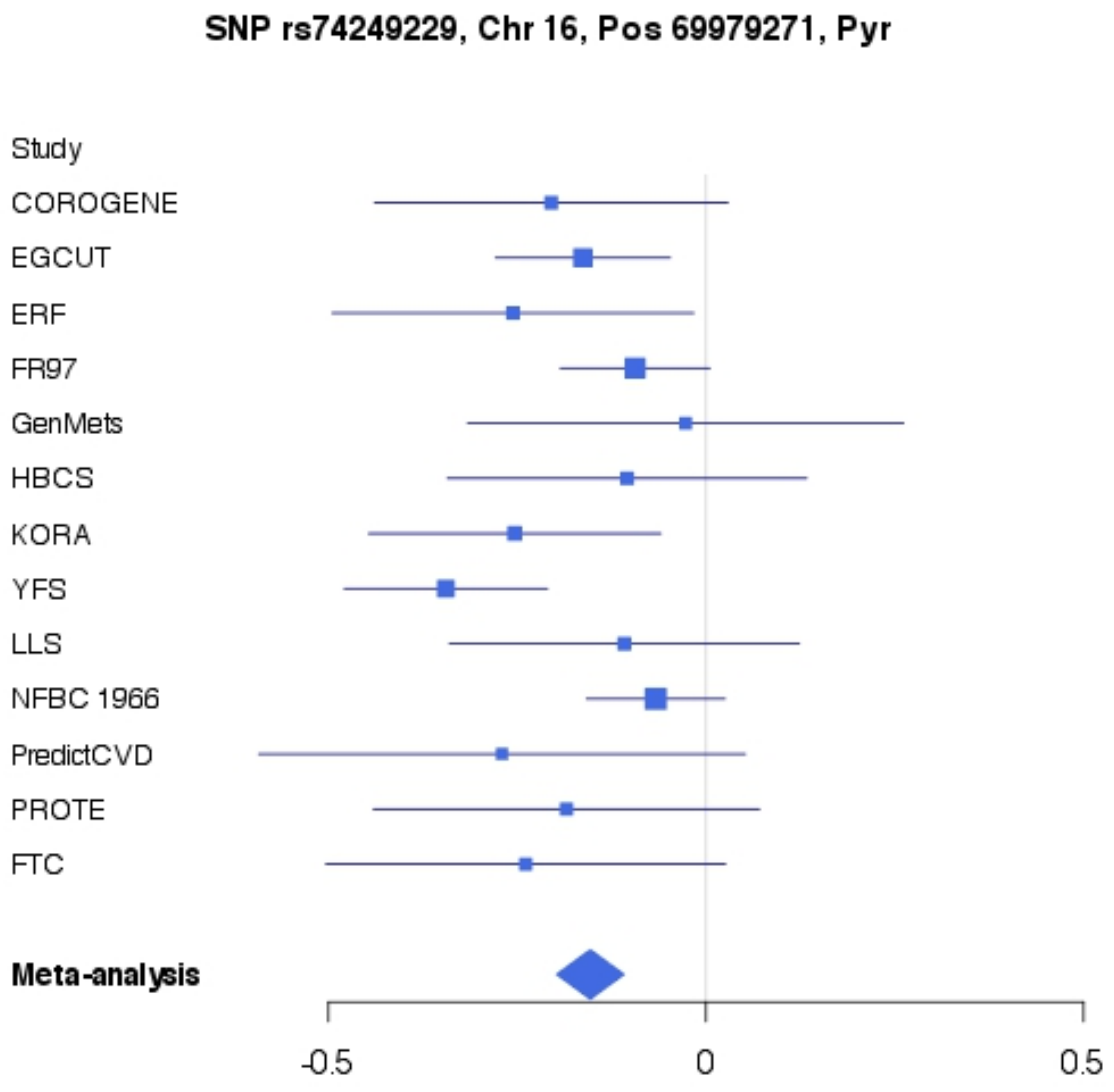
SNP rs74249229, Chr 16, Pos 69979271, Pyr
Study
COROGENE
EGCUT
ERF
FR97
GenMets
HBCS
KORA
YFS
LLS
NFBC 1966
PredictCVD
PROTE
FTC
Meta-analysis
-0.5
0
0.5

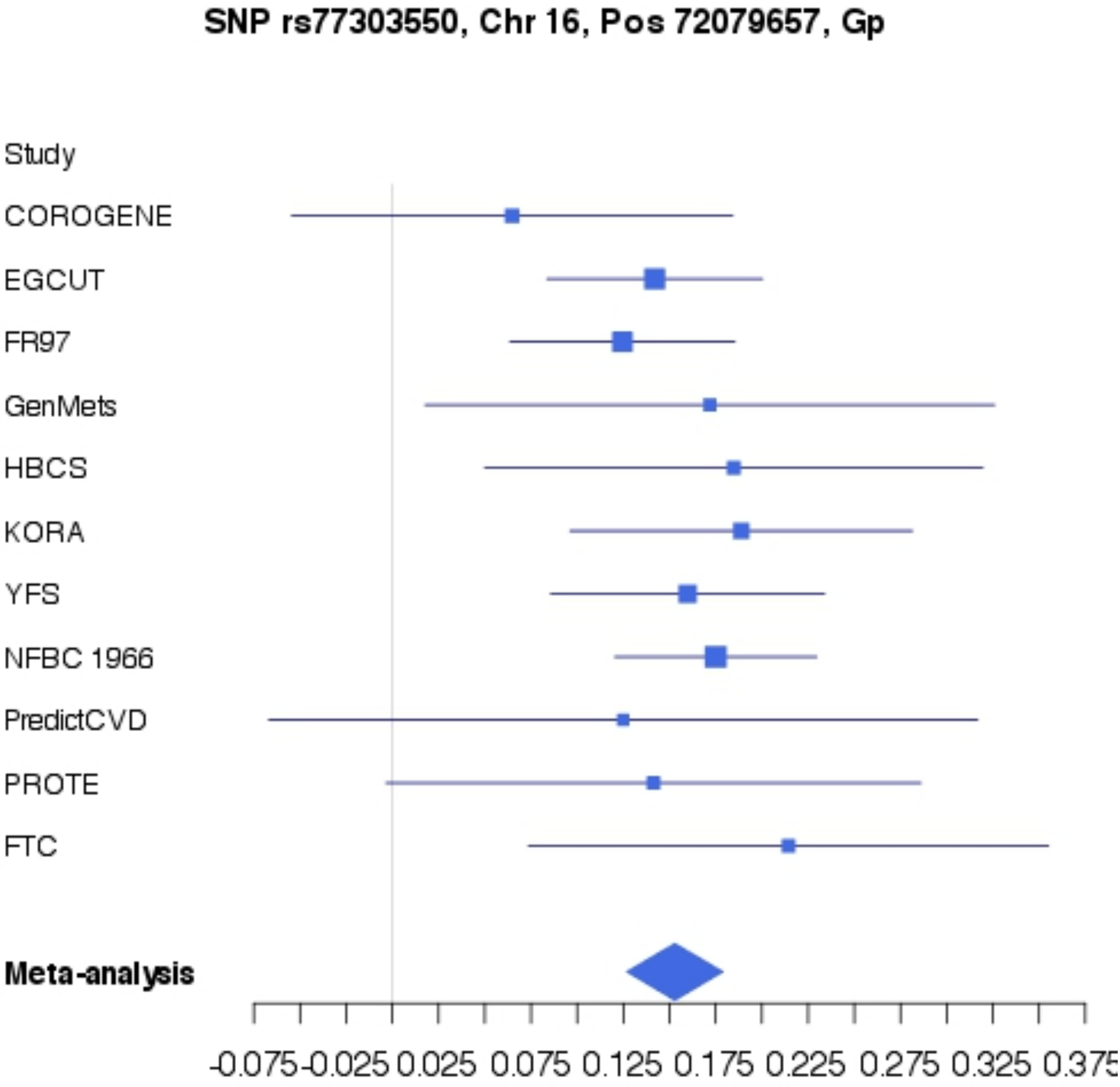
SNP rs77303550, Chr 16, Pos 72079657, Gp
Study
COROGENE
EGCUT
FR97
GenMets
HBCS
KORA
YFS
NFBC 1966
PredictCVD
PROTE
FTC
Meta-analysis
-0.075
-0.025
0.025
0.075
0.125
0.175
0.225
0.275
0.325
0.375

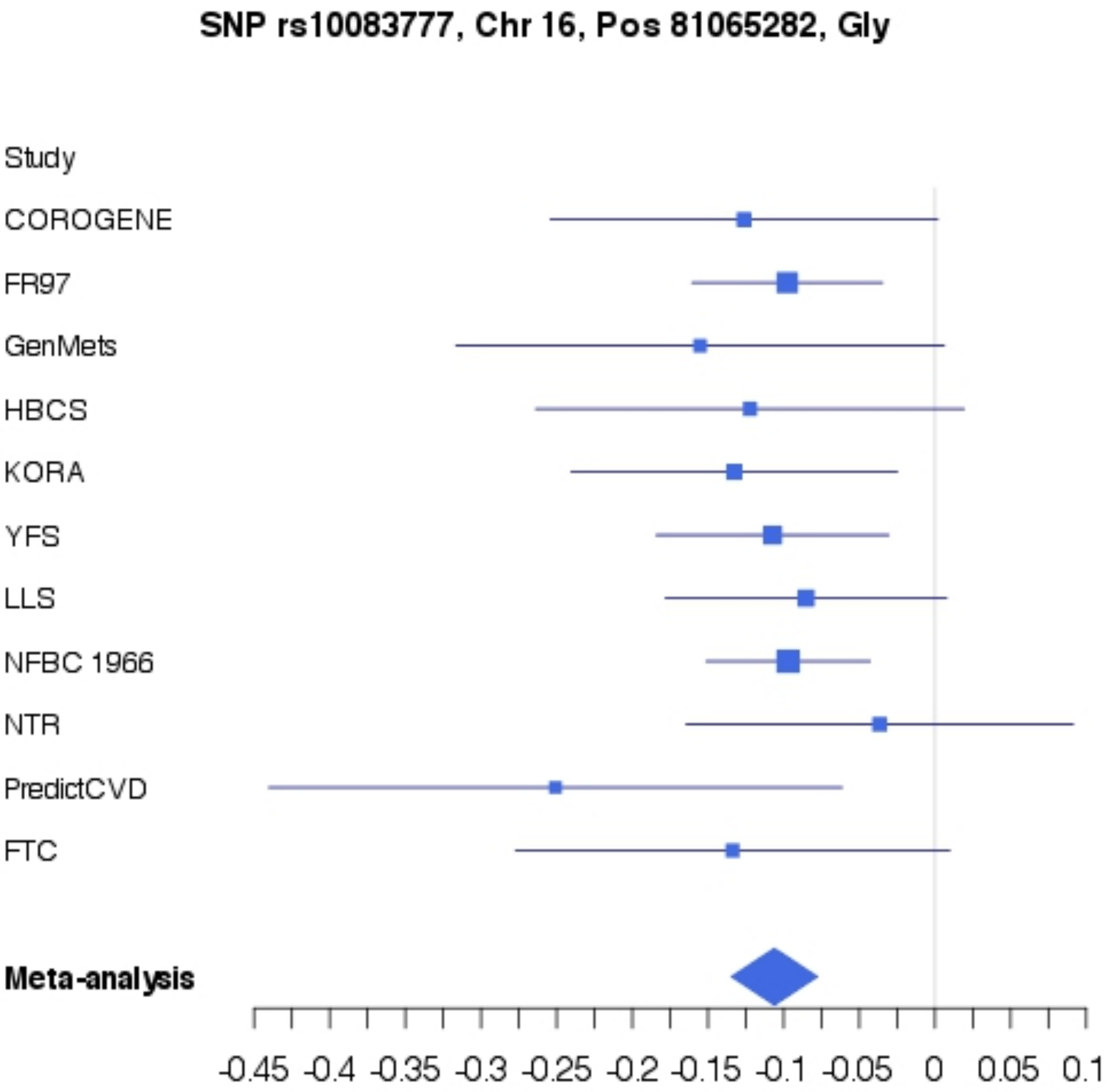
SNP rs10083777, Chr 16, Pos 81065282, Gly
Study
COROGENE
FR97
GenMets
HBCS
KORA
YFS
LLS
NFBC 1966
NTR
PredictCVD
FTC
Meta-analysis
-0.45 -0.4 -0.35 -0.3 -0.25 -0.2 -0.15 -0.1 -0.05 0 0.05 0.1

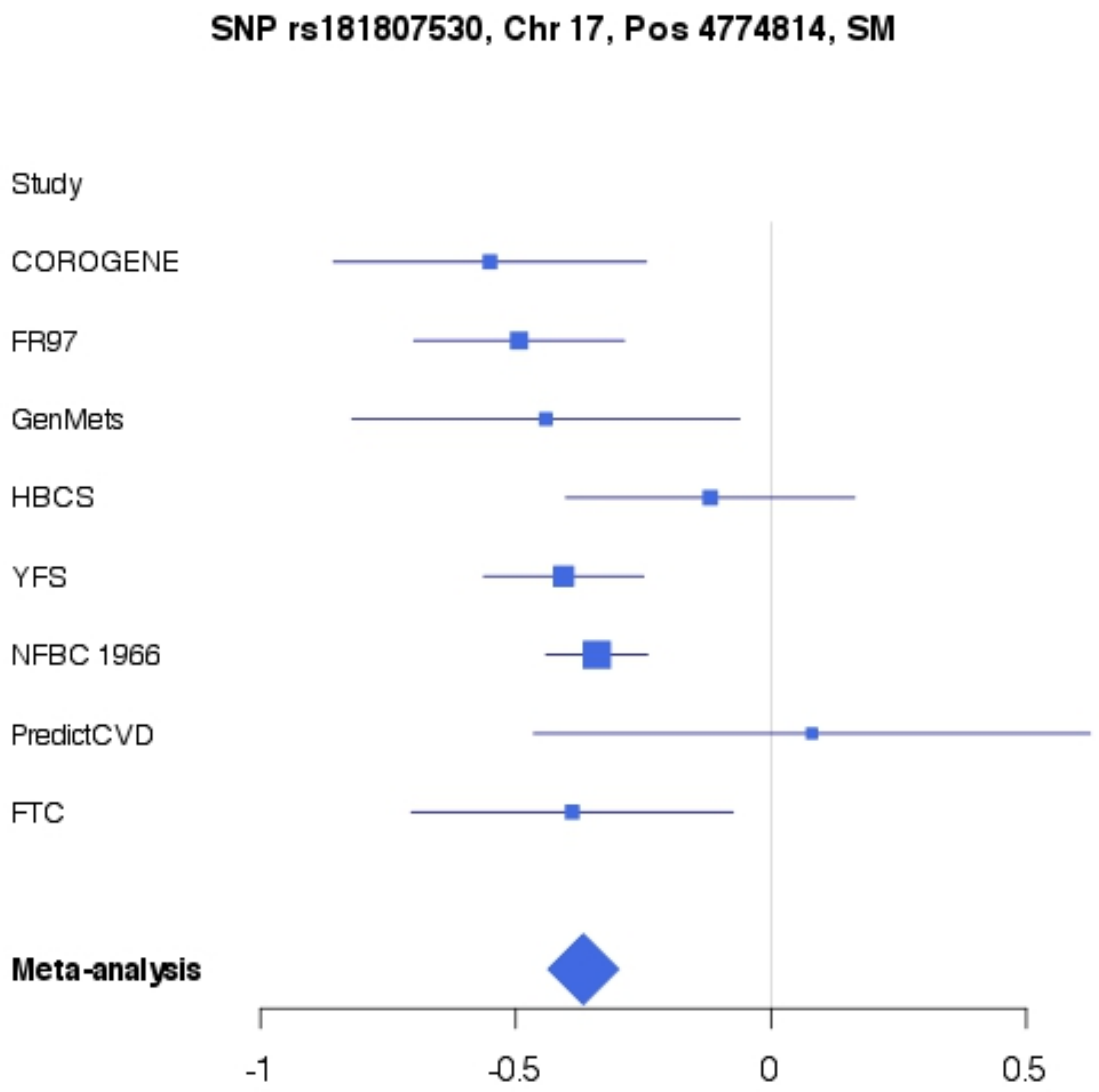

SNP rs181807530, Chr 17, Pos 4774814, SM
Study
COROGENE
FR97
GenMets
HBCS
YFS
NFBC 1966
PredictCVD
FTC
Meta-analysis
-1
-0.5
0
0.5

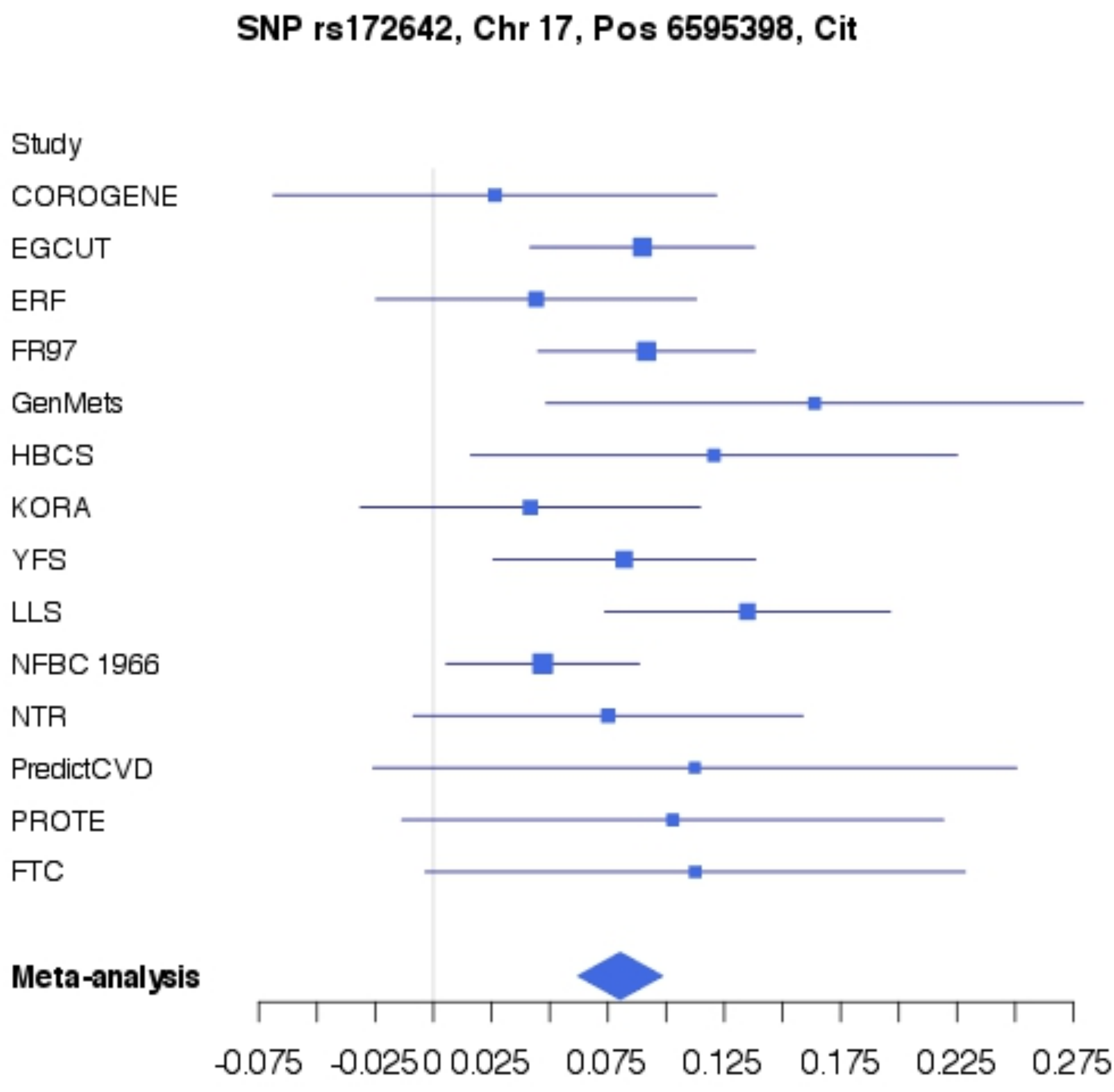

SNP rs172642, Chr 17, Pos 6595398, Cit
Study
COROGENE
EGCUT
ERF
FR97
GenMets
HBCS
KORA
YFS
LLS
NFBC 1966
NTR
PredictCVD
PROTE
FTC
Meta-analysis
-0.075
-0.025
0
0.025
0.075
0.125
0.175
0.225
0.275

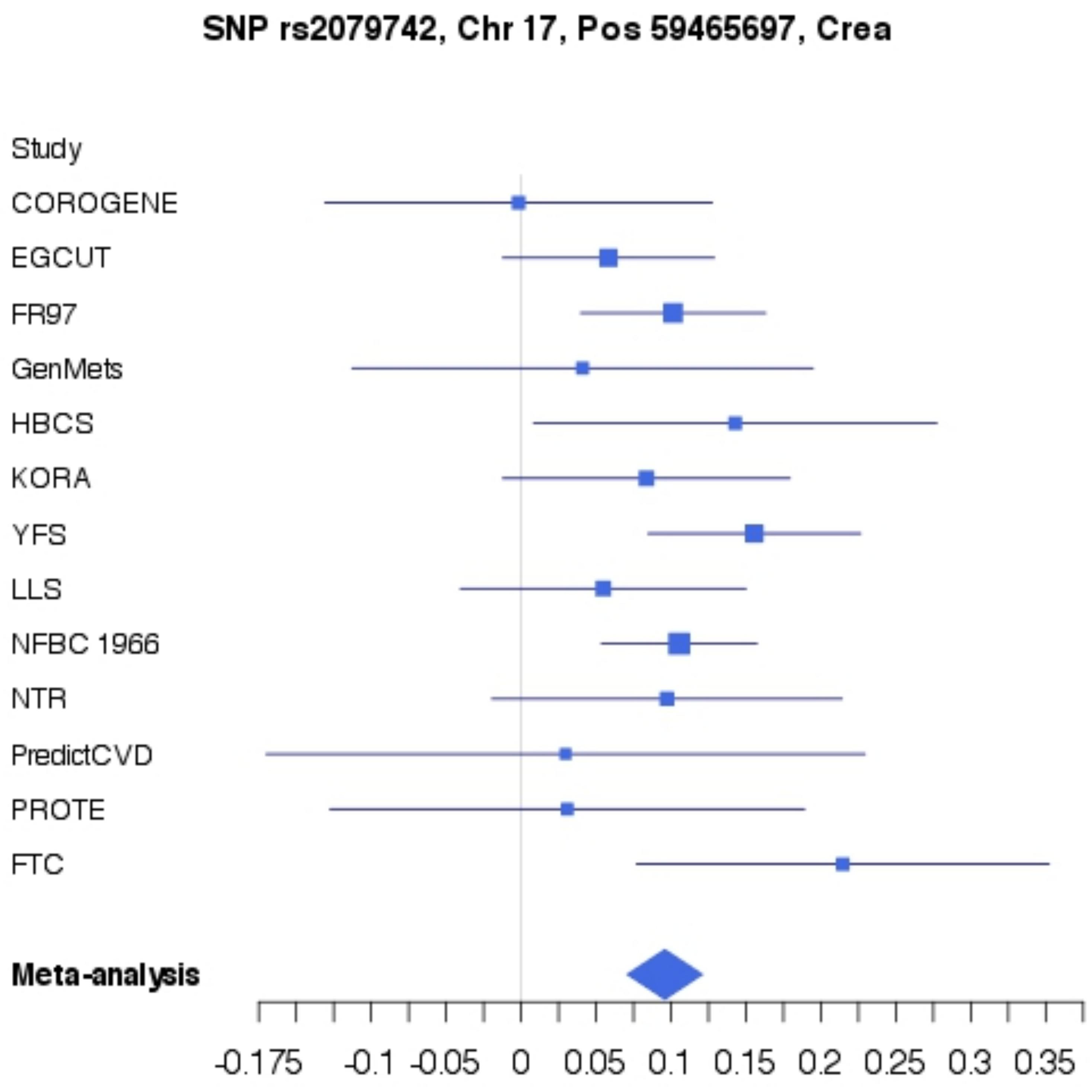
SNP rs2079742, Chr 17, Pos 59465697, Crea
Study
COROGENE
EGCUT
FR97
GenMets
HBCS
KORA
YFS
LLS
NFBC 1966
NTR
PredictCVD
PROTE
FTC
Meta-analysis
-0.175
-0.1
-0.05
0
0.05
0.1
0.15
0.2
0.25
0.3
0.35

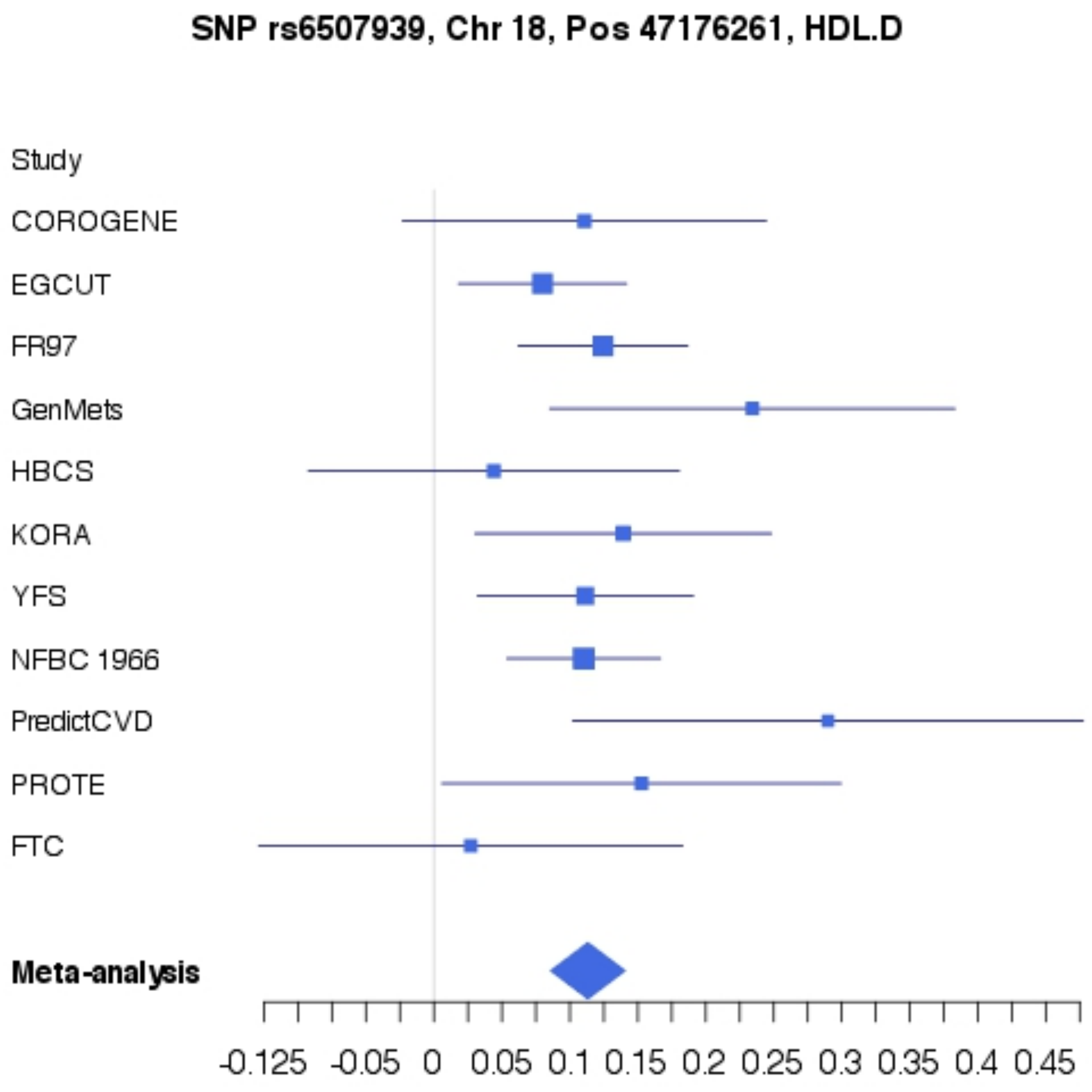
SNP rs6507939, Chr 18, Pos 47176261, HDL.D
Study
COROGENE
EGCUT
FR97
GenMets
HBCS
KORA
YFS
NFBC 1966
PredictCVD
PROTE
FTC
Meta-analysis
-0.125
-0.05
0
0.05
0.1
0.15
0.2
0.25
0.3
0.35
0.4
0.45

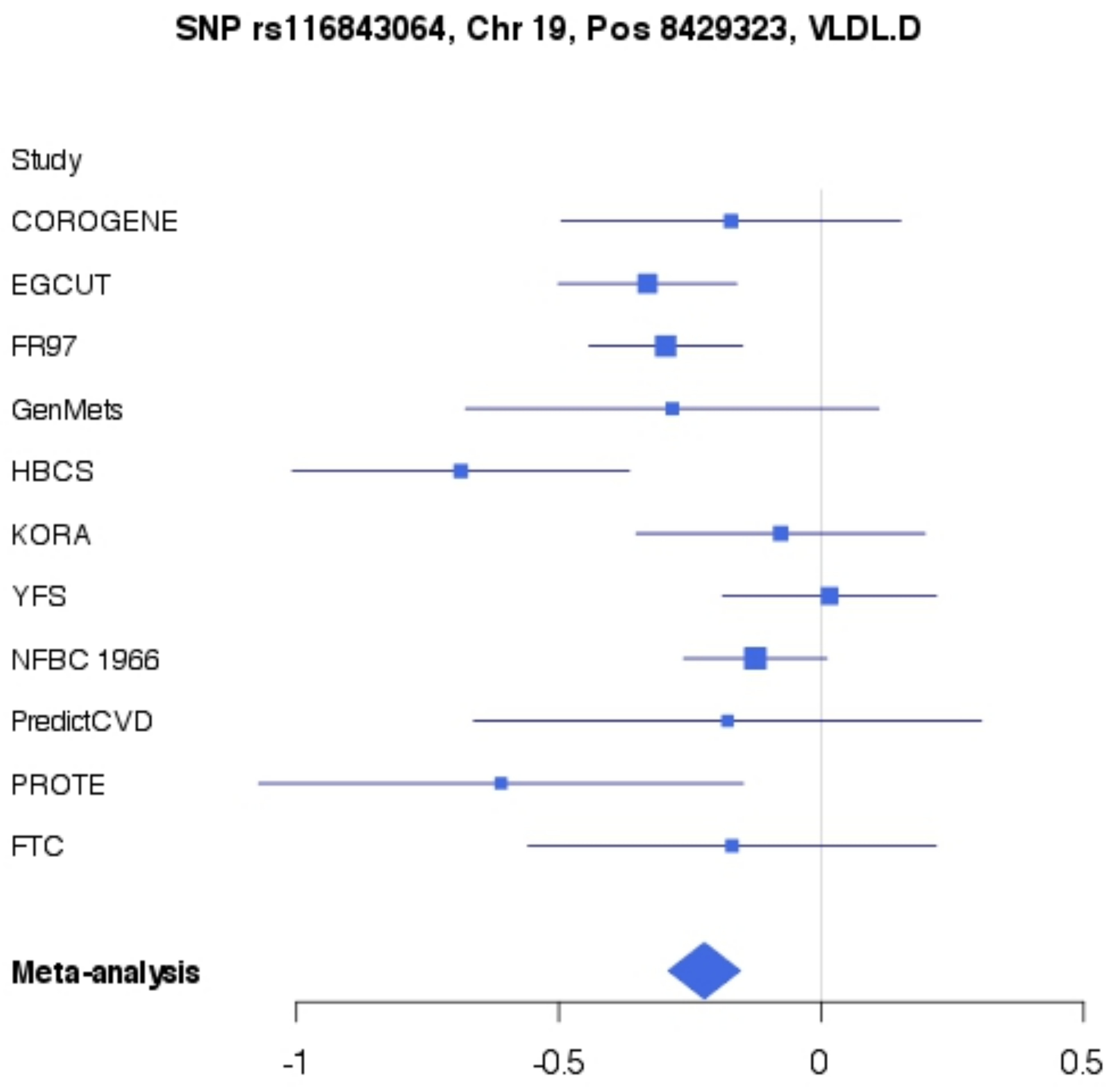
SNP rs116843064, Chr 19, Pos 8429323, VLDL.D
Study
COROGENE
EGCUT
FR97
GenMets
HBCS
KORA
YFS
NFBC 1966
PredictCVD
PROTE
FTC
Meta-analysis
-1
-0.5
0
0.5

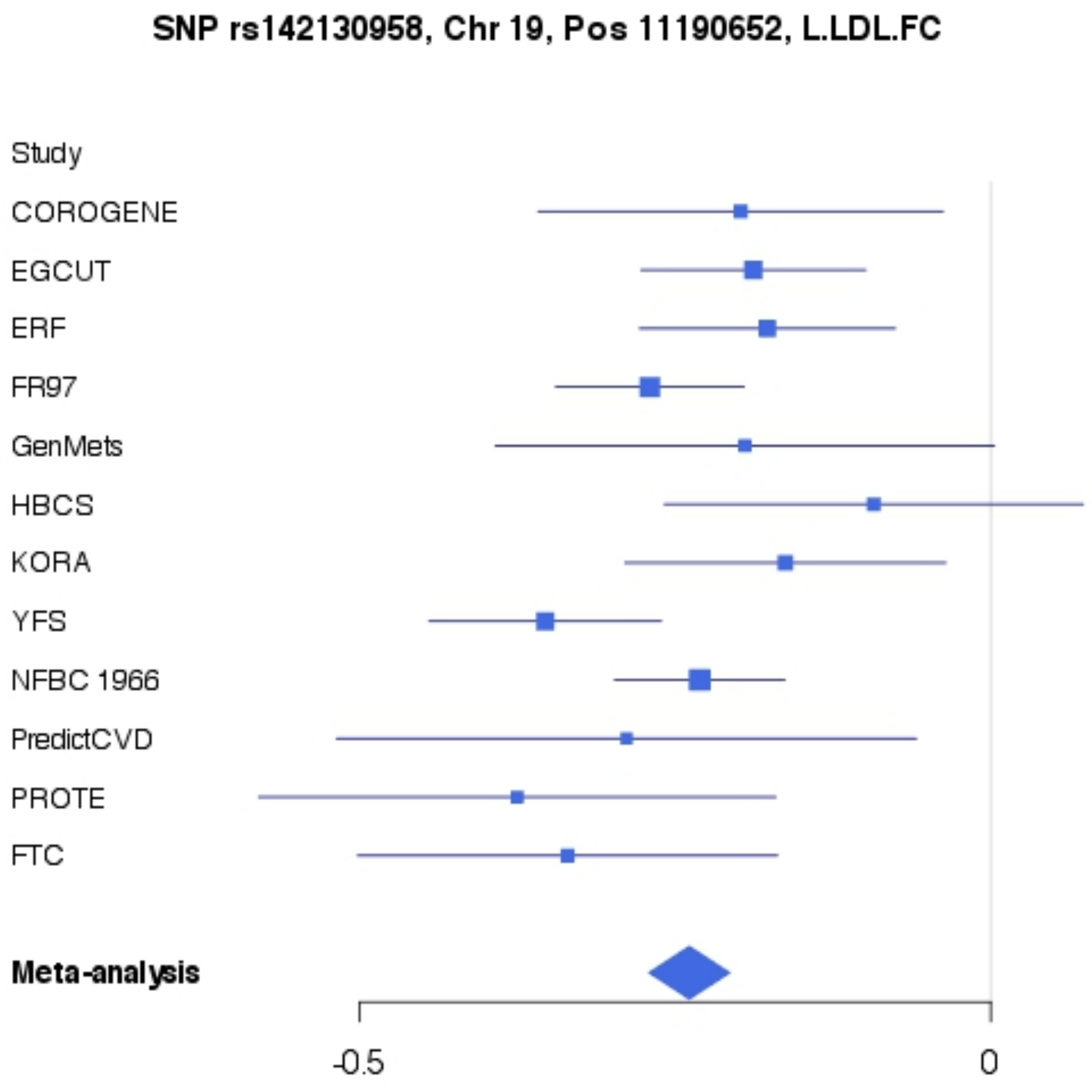

SNP rs142130958, Chr 19, Pos 11190652, L.LDL.FC
Study
COROGENE
EGCUT
ERF
FR97
GenMets
HBCS
KORA
YFS
NFBC 1966
PredictCVD
PROTE
FTC
Meta-analysis
-0.5
0

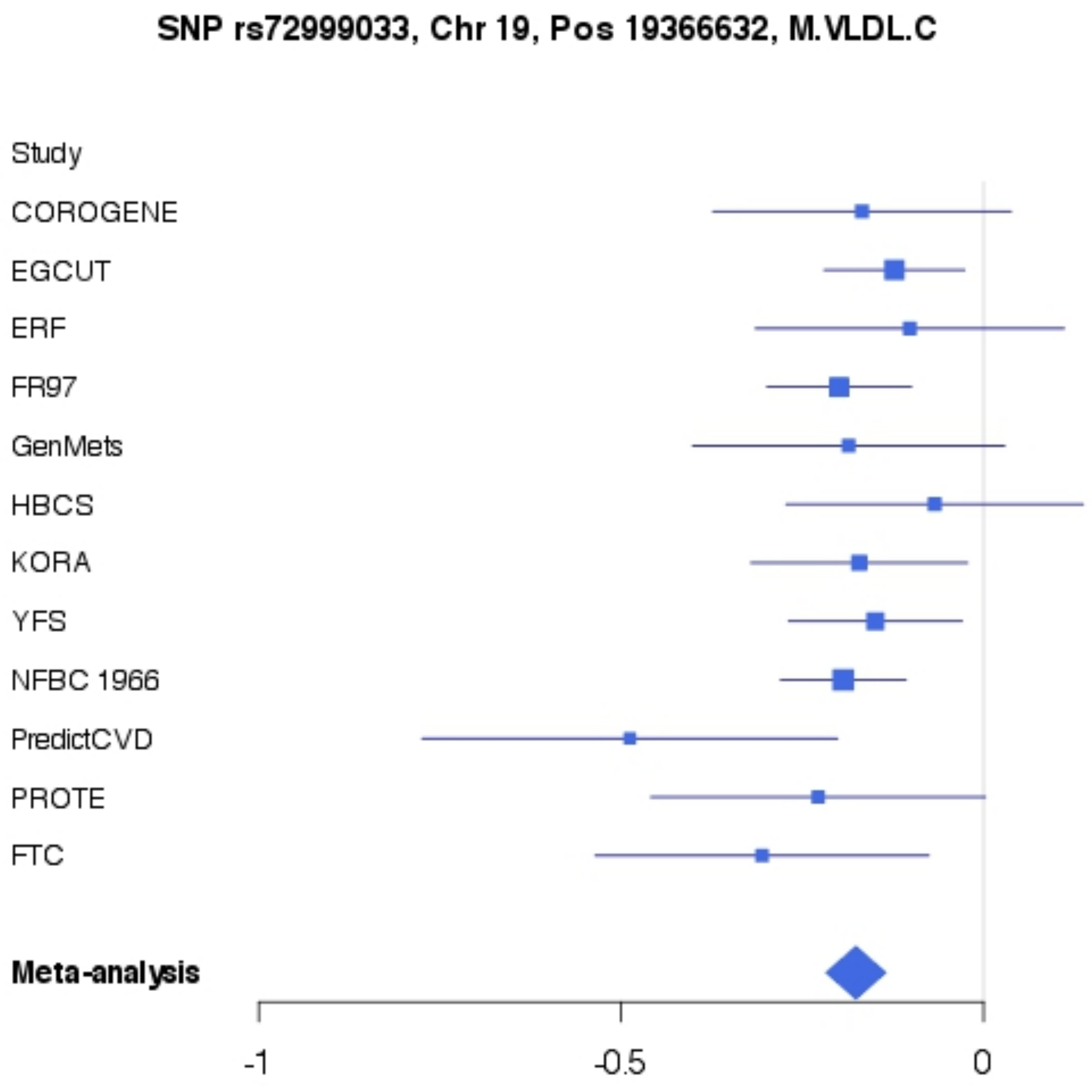
SNP rs72999033, Chr 19, Pos 19366632, M.VLDL.C
Study
COROGENE
EGCUT
ERF
FR97
GenMets
HBCS
KORA
YFS
NFBC 1966
PredictCVD
PROTE
FTC
Meta-analysis
-1
-0.5
0

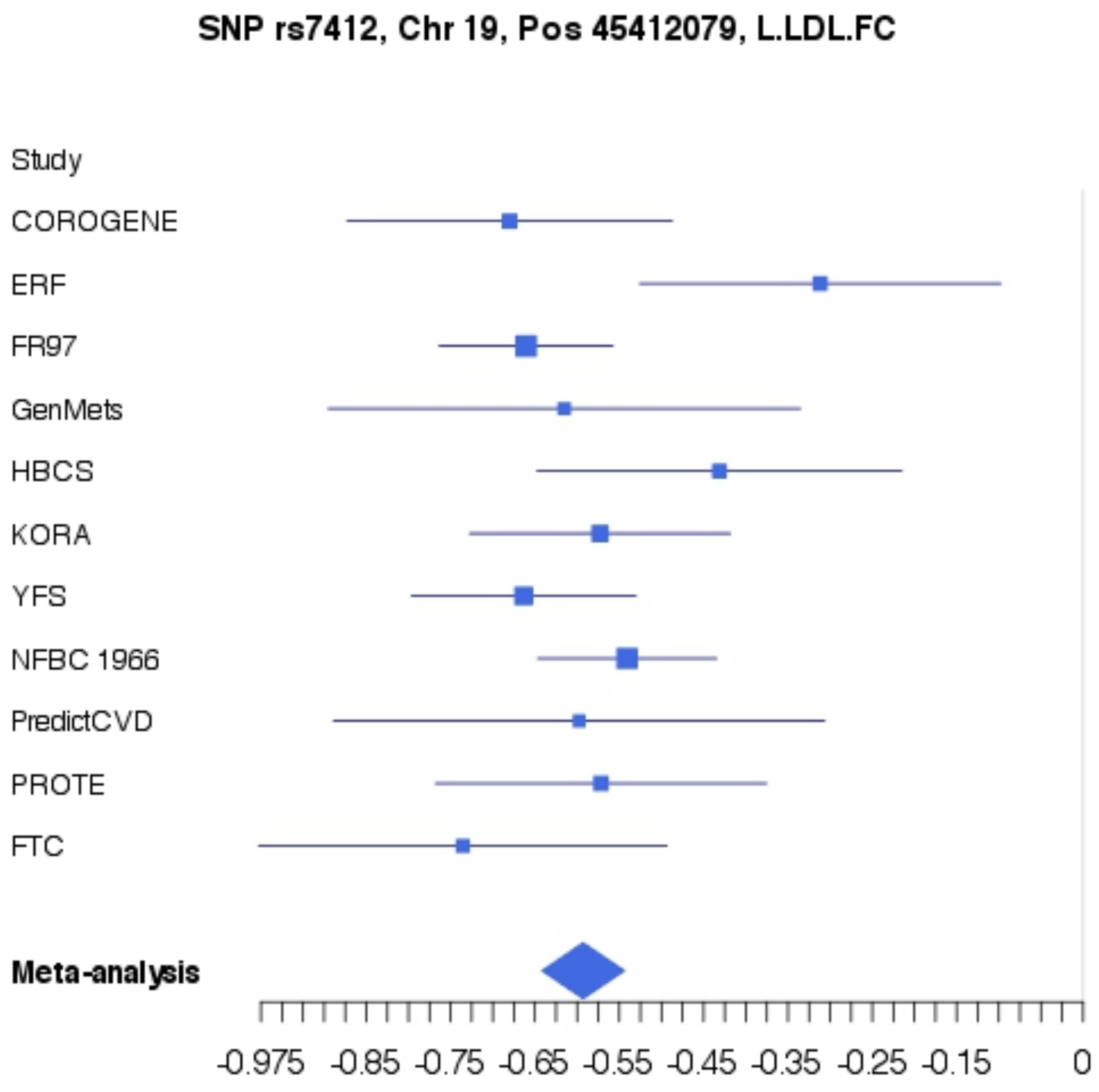
SNP rs7412, Chr 19, Pos 45412079, L.LDL.FC
Study
COROGENE
ERF
FR97
GenMets
HBCS
KORA
YFS
NFBC 1966
PredictCVD
PROTE
FTC
Meta-analysis
-0.975
-0.85
-0.75
-0.65
-0.55
-0.45
-0.35
-0.25
-0.15
0

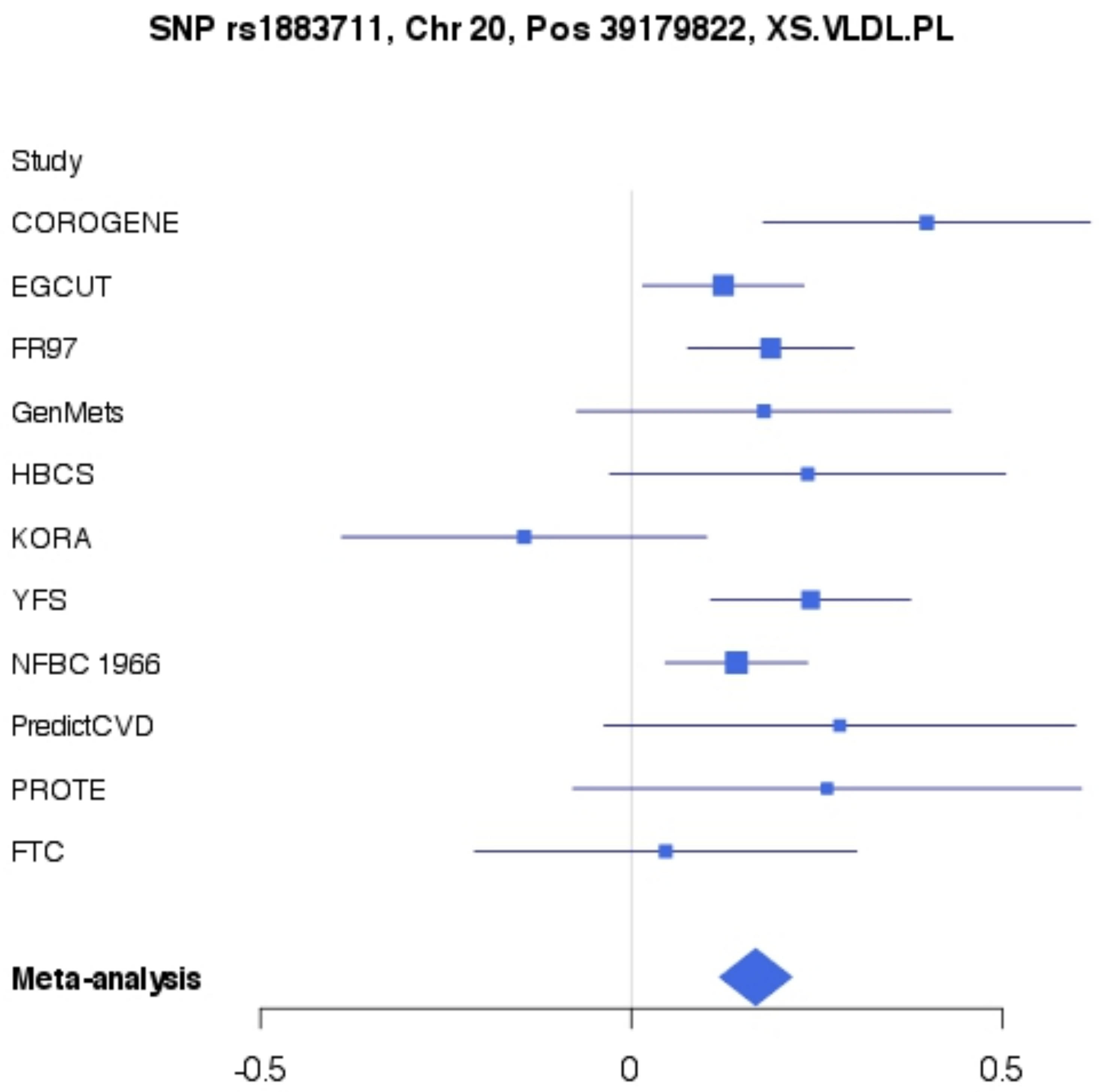
SNP rs1883711, Chr 20, Pos 39179822, XS.VLDL.PL
Study
COROGENE
EGCUT
FR97
GenMets
HBCS
KORA
YFS
NFBC 1966
PredictCVD
PROTE
FTC
Meta-analysis
-0.5
0
0.5

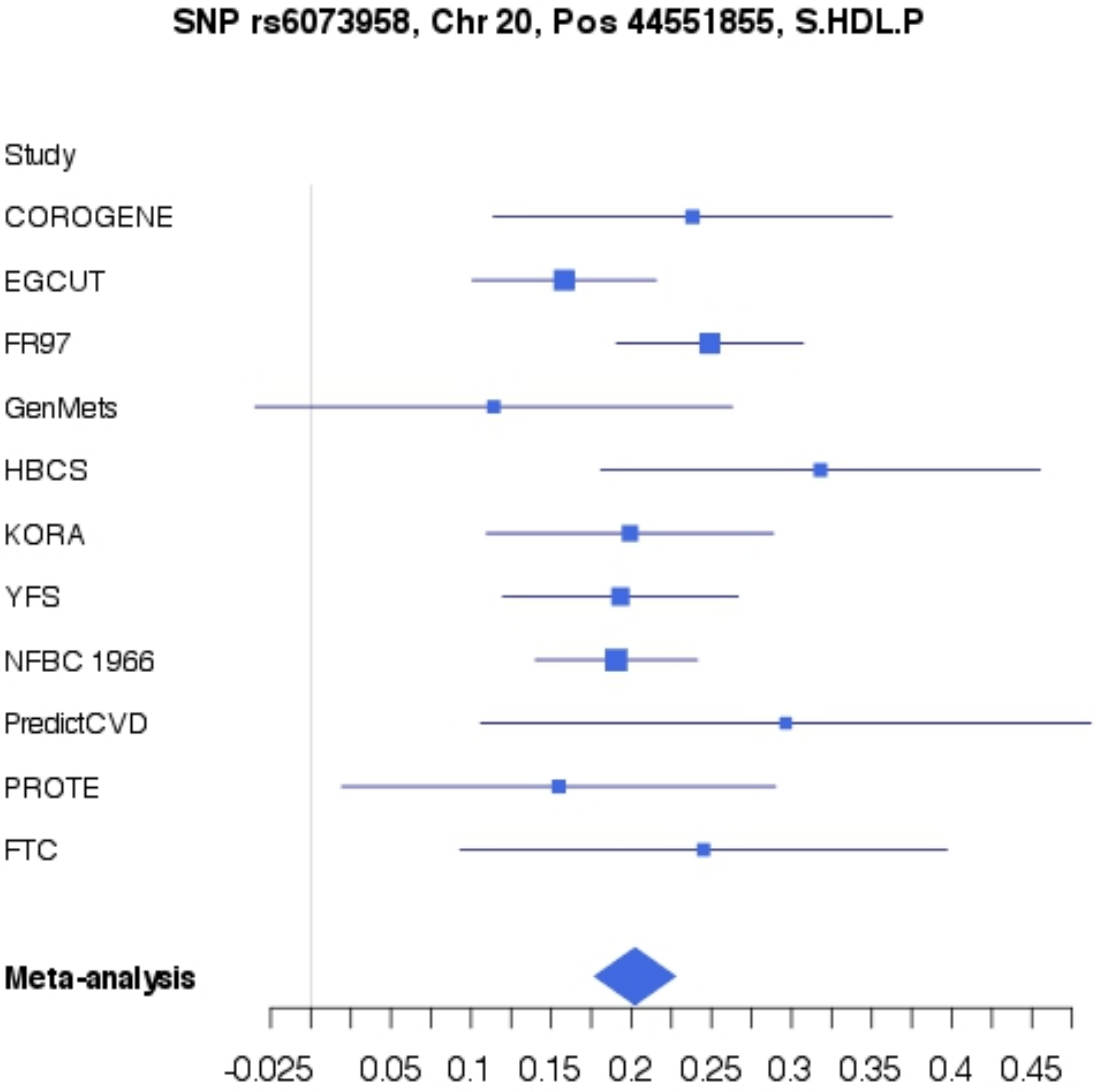
SNP rs6073958, Chr 20, Pos 44551855, S.HDL.P
Study
COROGENE
EGCUT
FR97
GenMets
HBCS
KORA
YFS
NFBC 1966
PredictCVD
PROTE
FTC
Meta-analysis
-0.025
0.05
0.1
0.15
0.2
0.25
0.3
0.35
0.4
0.45

Supplementary Figure 3. Representative metabolite-specific original (untransformed) distributions and descriptive statistics (range, mean, median and skewness). The distributions drawn are from the largest cohort NFBC1966 with n = 5923.

NFBC66 measures summary

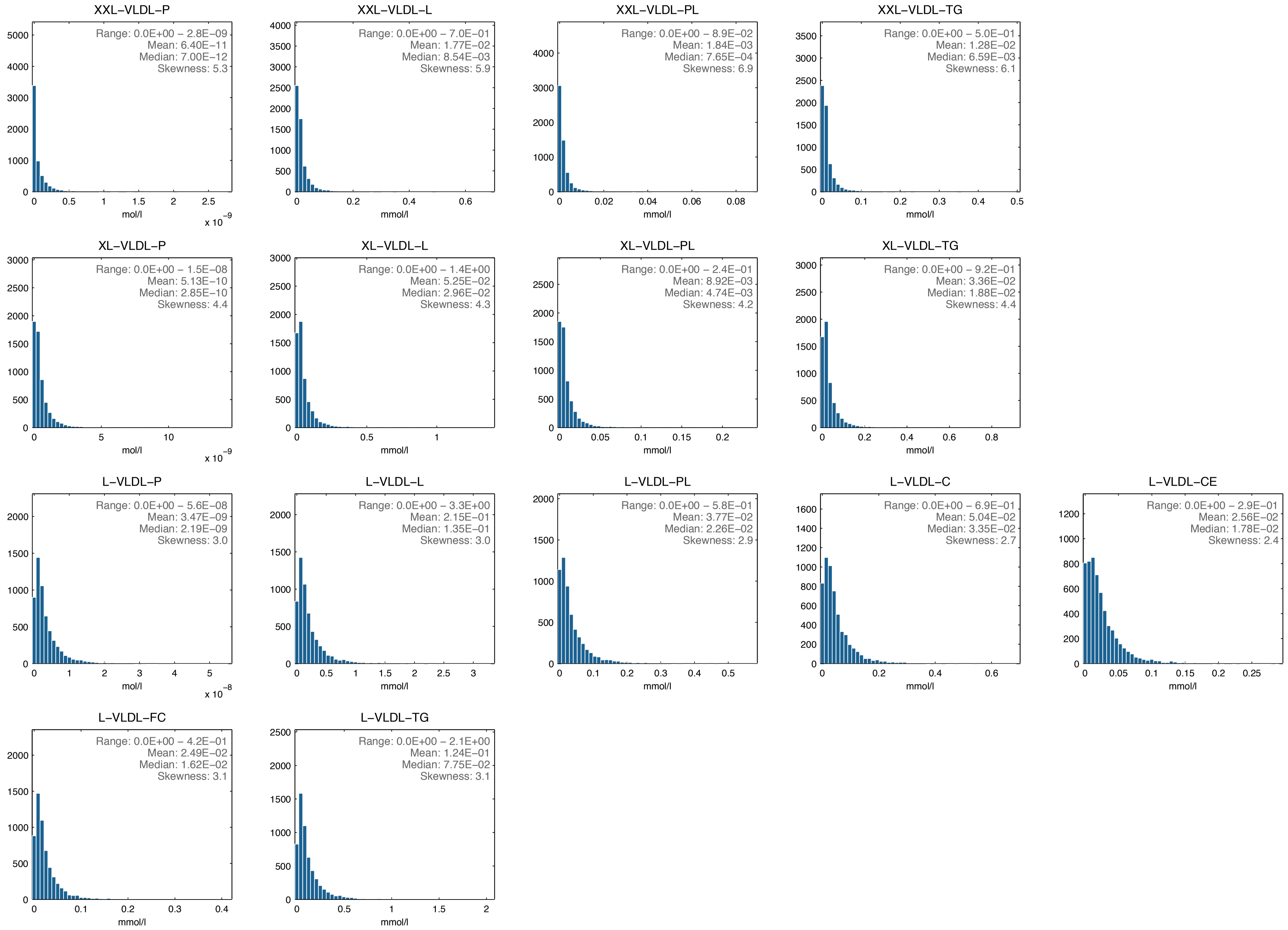

NFBC66 measures summary

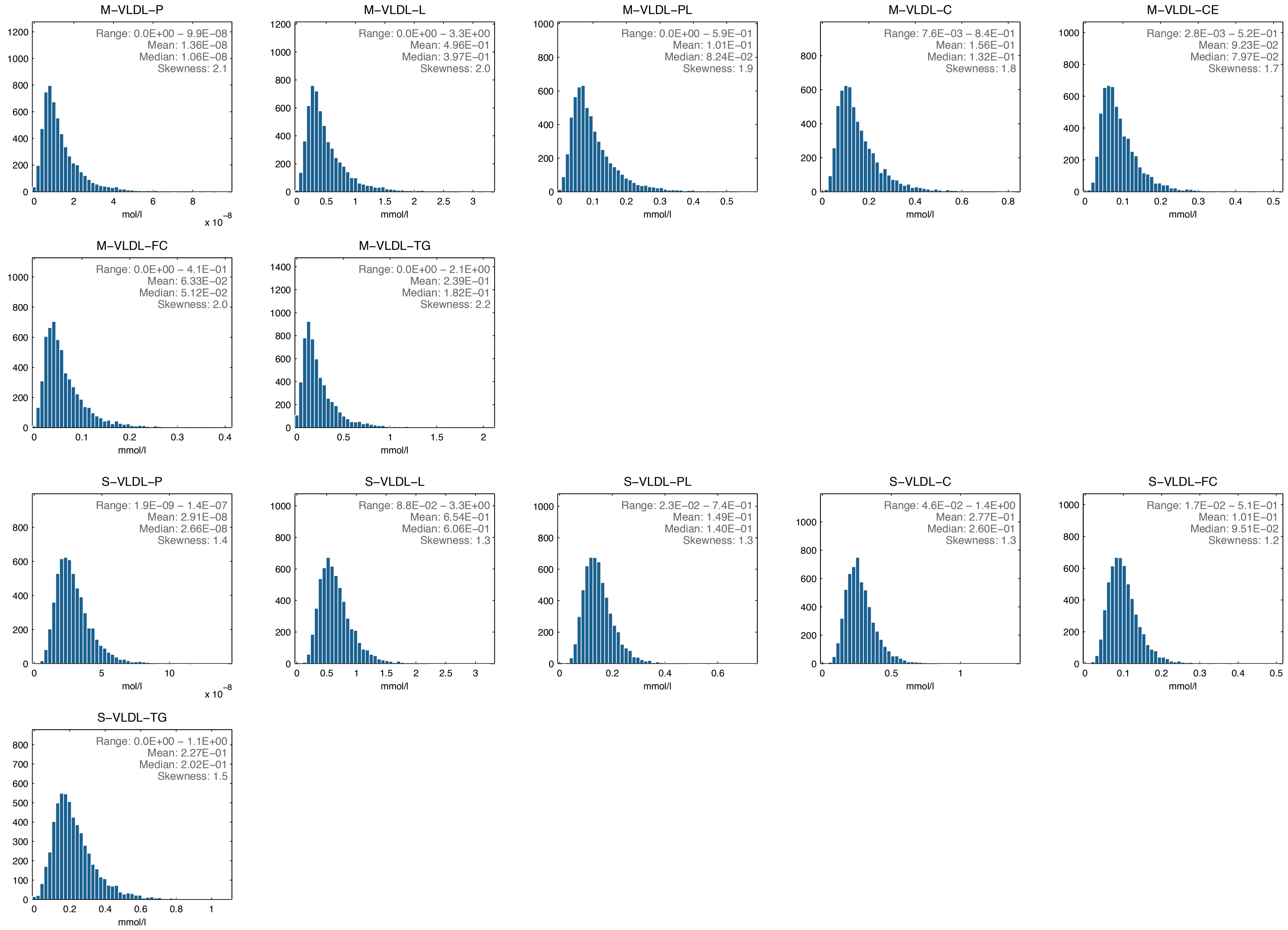

NFBC66 measures summary

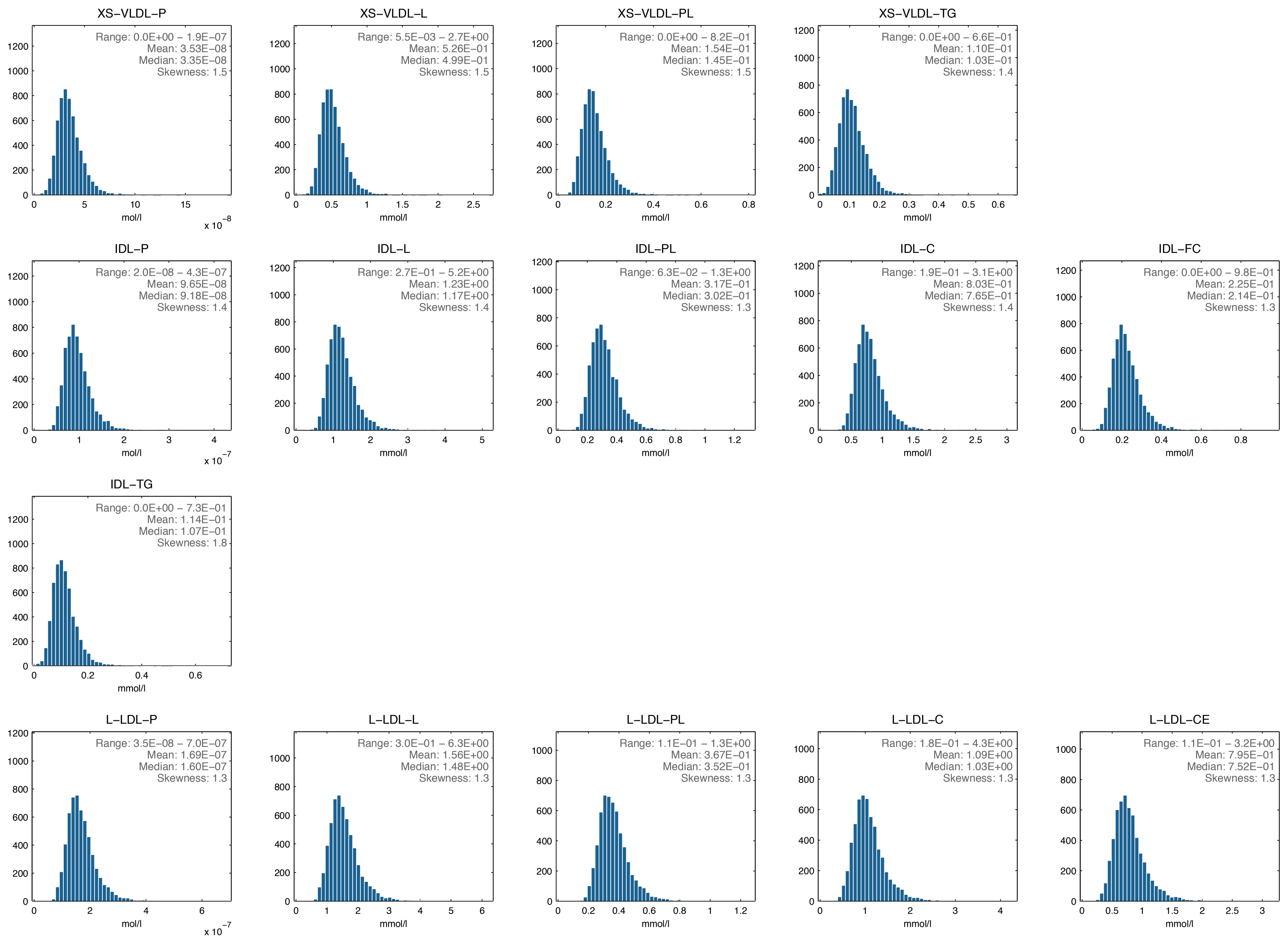

NFBC66 measures summary

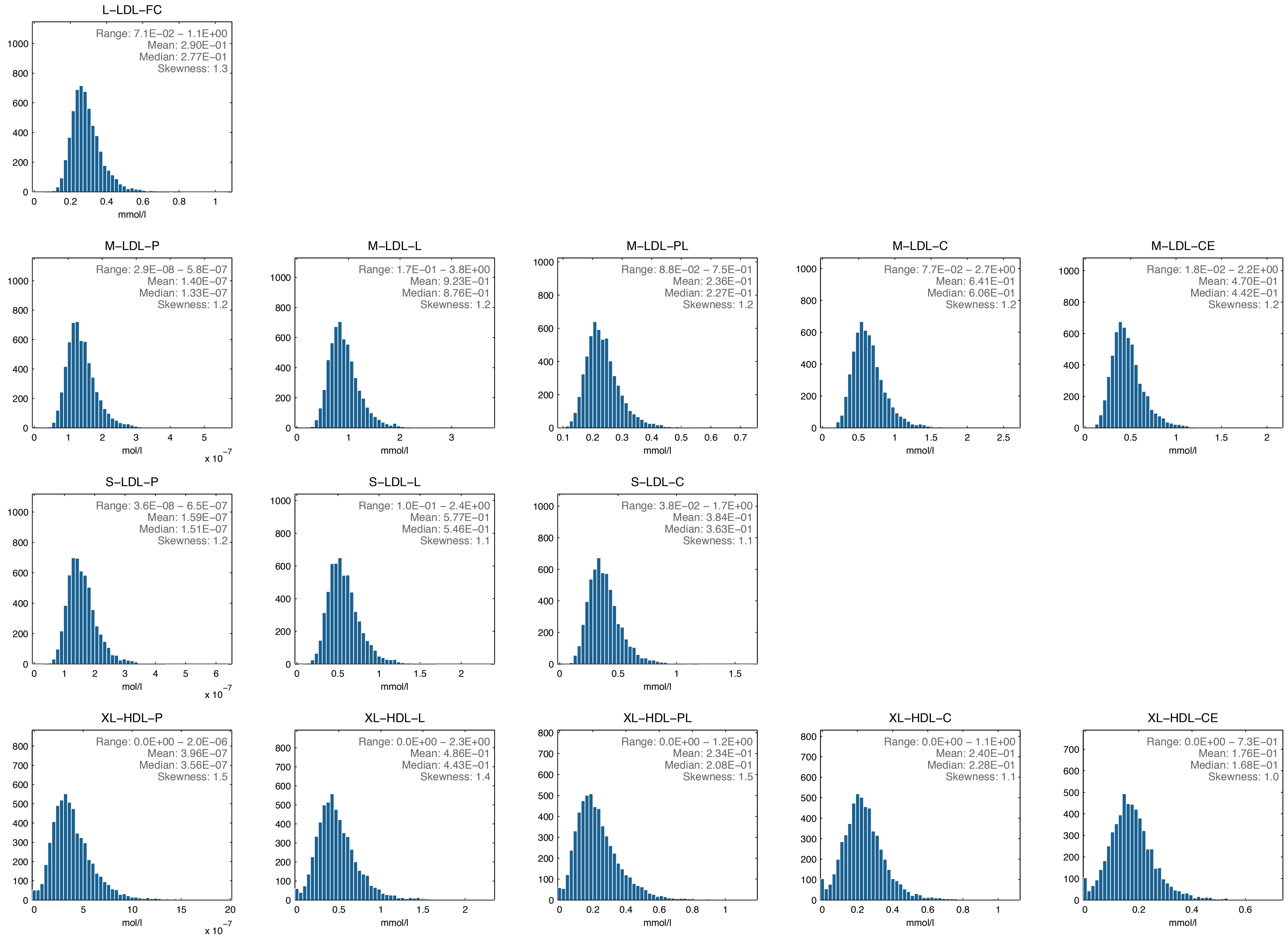

NFBC66 measures summary

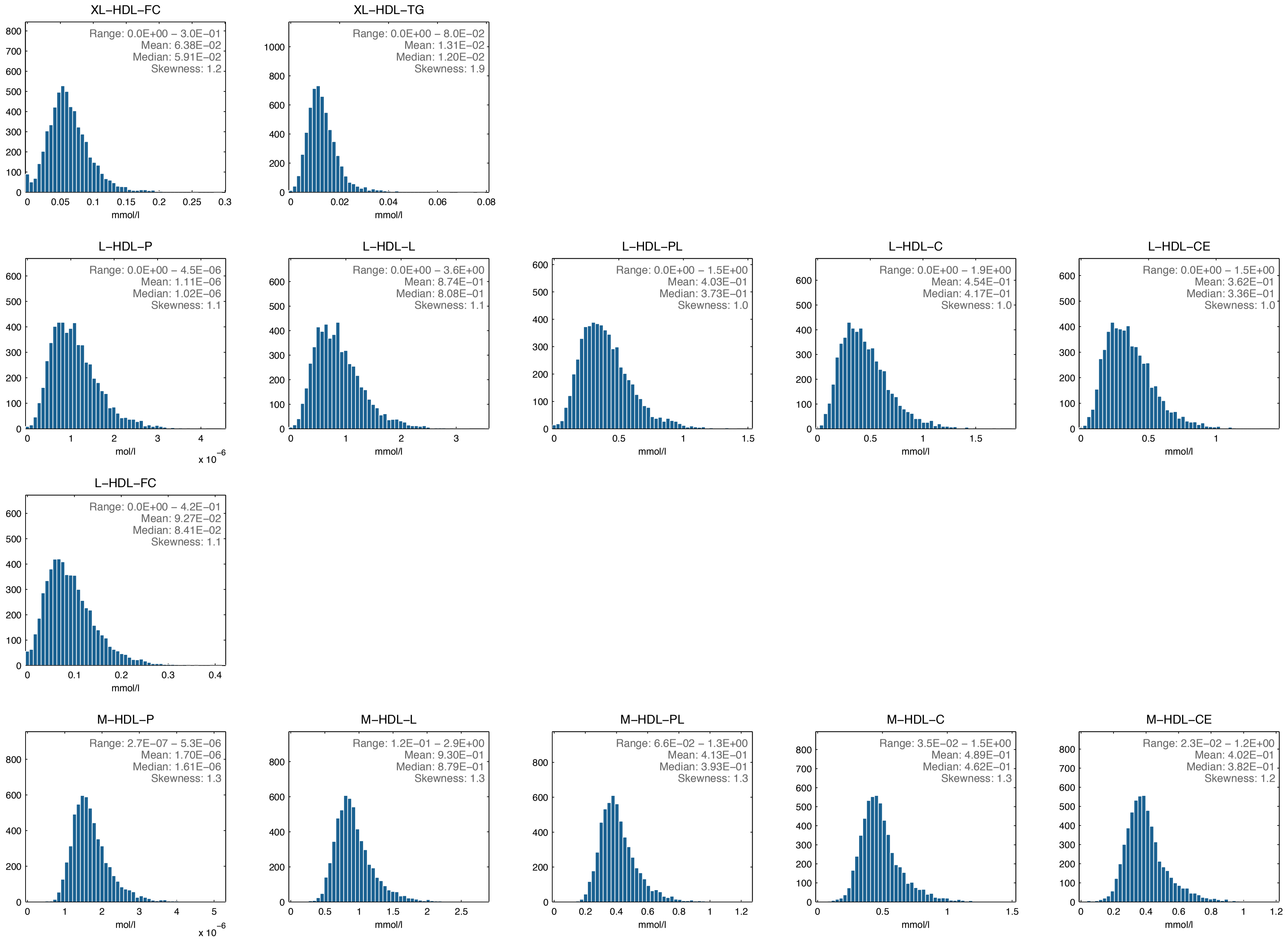

NFBC66 measures summary

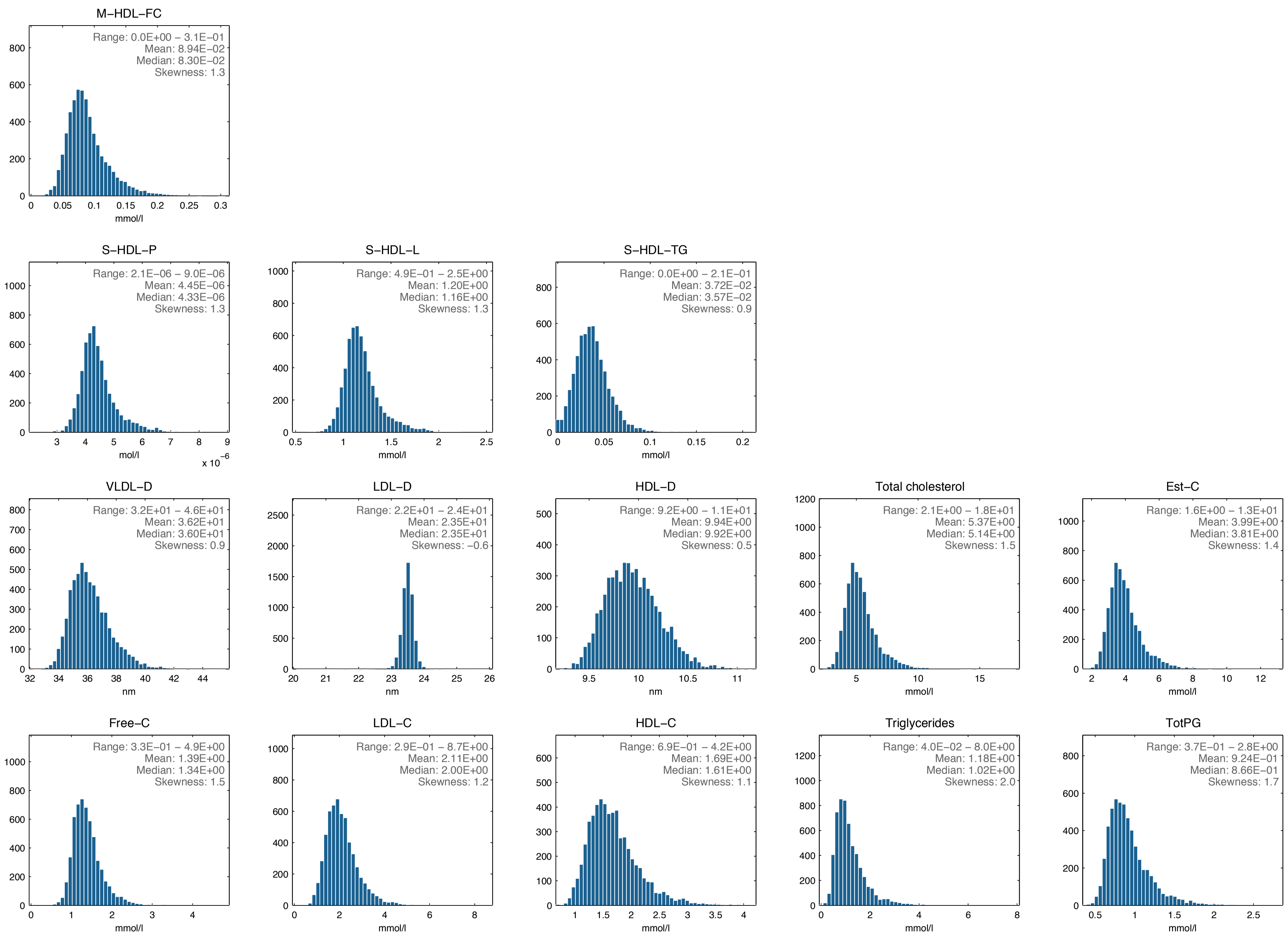

NFBC66 measures summary

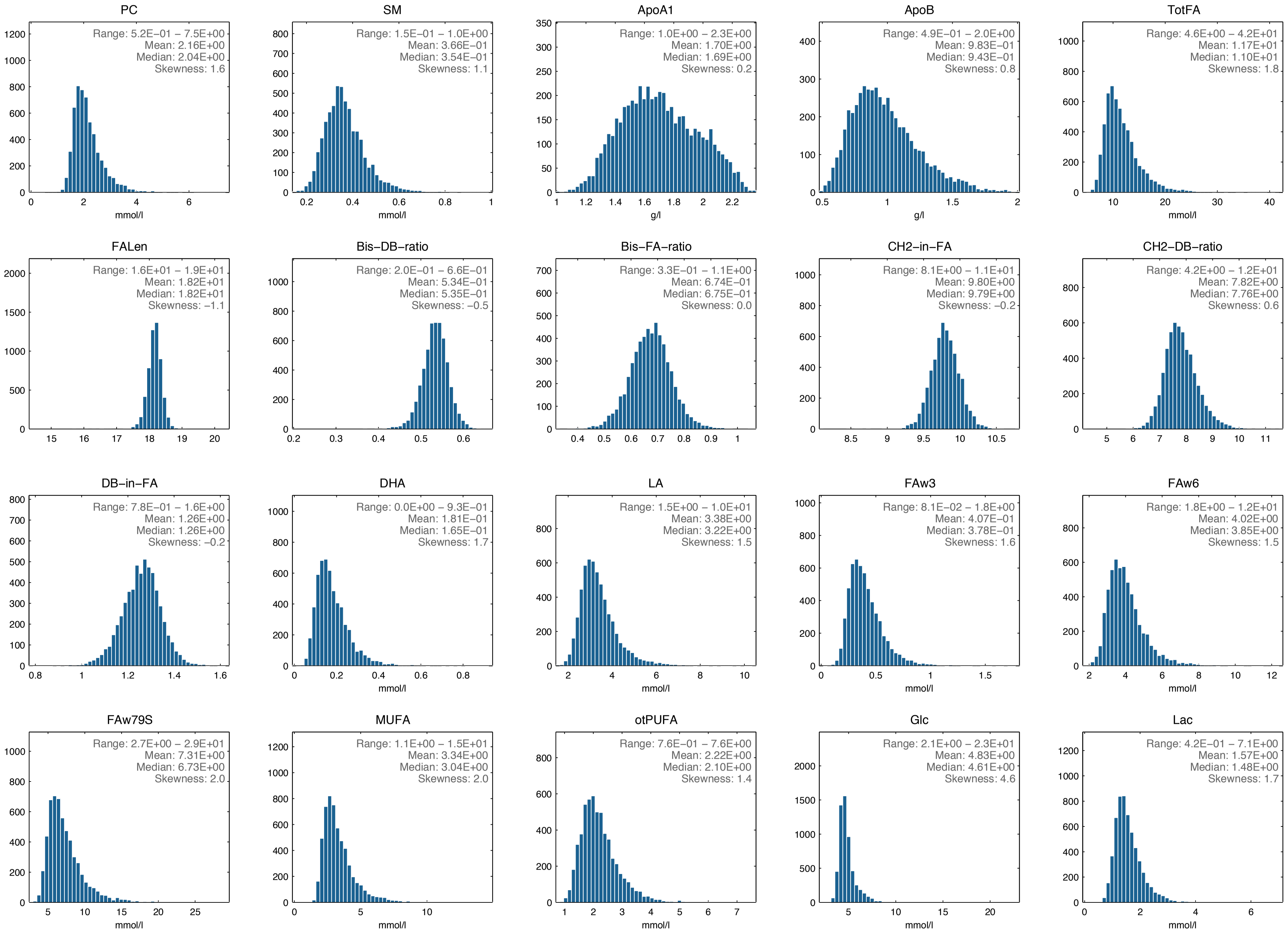

NFBC66 measures summary

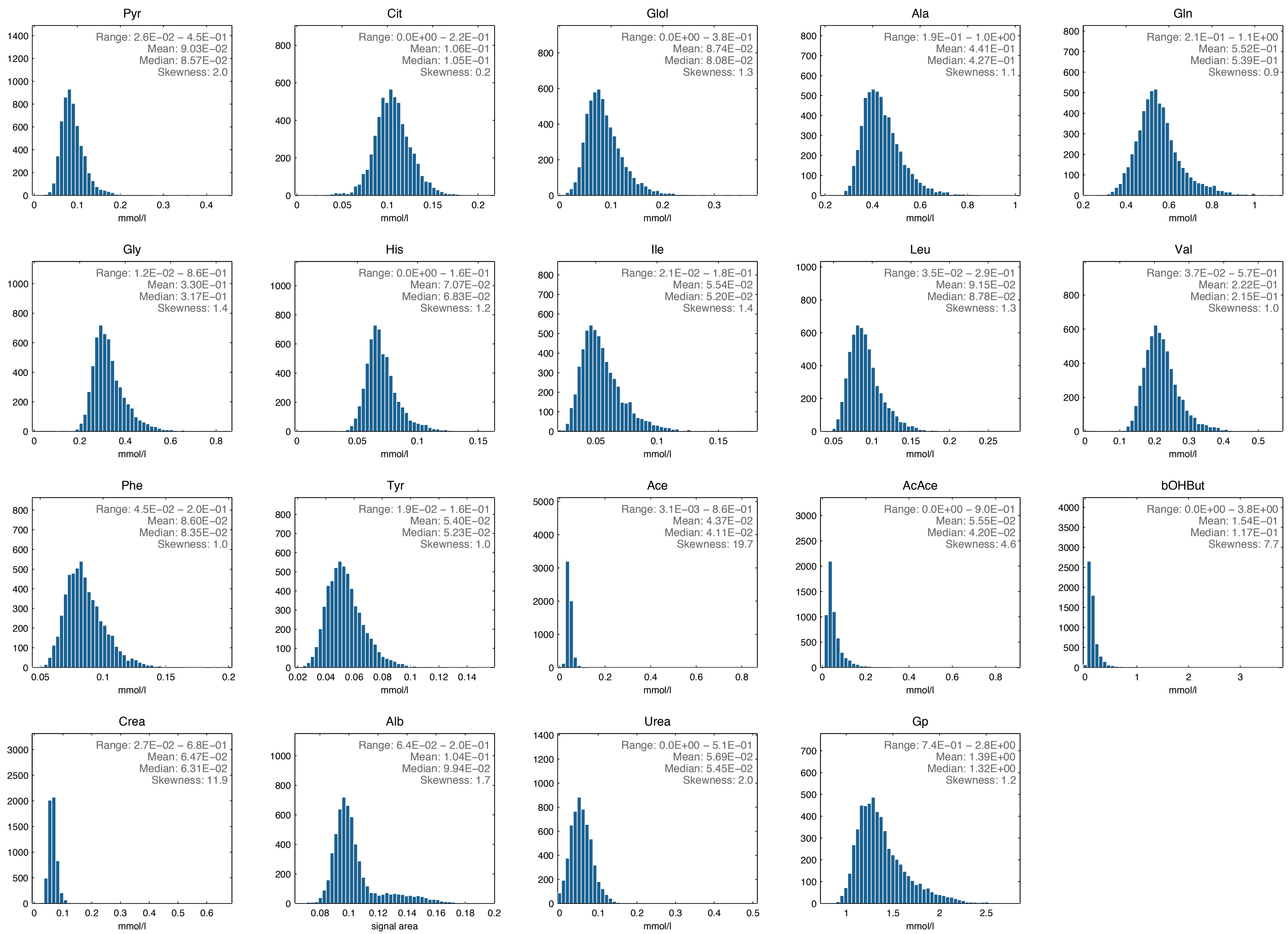

Supplementary Figure 4. Metabolite-specific original (untransformed) distributions and descriptive statistics (for each metabolite; range, mean, median, relative standard deviation and skewness) from ERF cohort (N = 2118) for available metabolic measures.

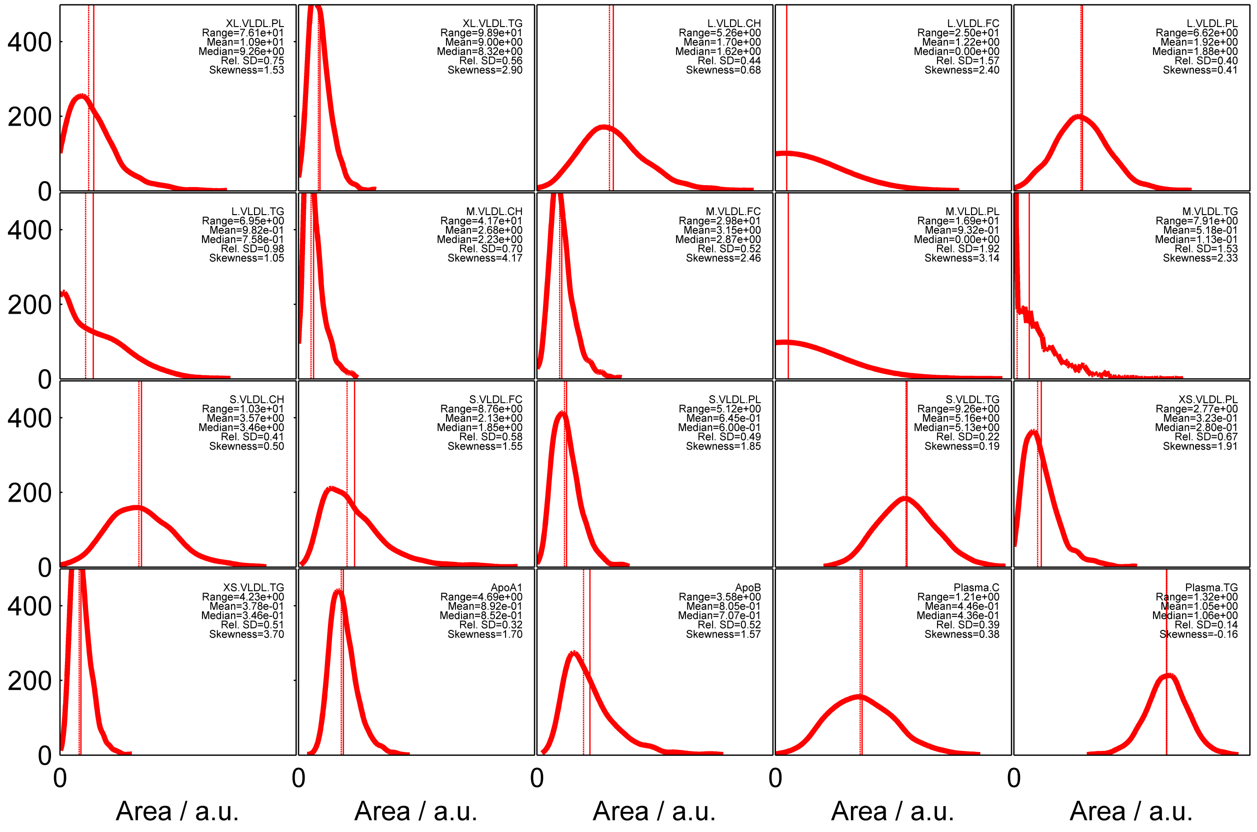

XL.VLDL.PL
Range=7.61e+01
Mean=1.09e+01
Median=9.26e+00
Rel. SD=0.75
Skewness=1.53
XL.VLDL.TG
Range=9.89e+01
Mean=9.00e+00
Median=8.32e+00
Rel. SD=0.56
Skewness=2.90
L.VLDL.CH
Range=5.26e+00
Mean=1.70e+00
Median=1.62e+00
Rel. SD=0.44
Skewness=0.68
L.VLDL.FC
Range=2.50e+01
Mean=1.22e+00
Median=0.00e+00
Rel. SD=1.57
Skewness=2.40
L.VLDL.PL
Range=6.62e+00
Mean=1.92e+00
Median=1.88e+00
Rel. SD=0.40
Skewness=0.41
L.VLDL.TG
Range=6.95e+00
Mean=9.82e-01
Median=7.58e-01
Rel. SD=0.98
Skewness=1.05
M.VLDL.CH
Range=4.17e+01
Mean=2.68e+00
Median=2.23e+00
Rel. SD=0.70
Skewness=4.17
M.VLDL.FC
Range=2.98e+01
Mean=3.15e+00
Median=2.87e+00
Rel. SD=0.52
Skewness=2.46
M.VLDL.PL
Range=1.69e+01
Mean=9.32e-01
Median=0.00e+00
Rel. SD=1.92
Skewness=3.14
M.VLDL.TG
Range=7.91e+00
Mean=5.18e-01
Median=1.13e-01
Rel. SD=1.53
Skewness=2.33
S.VLDL.CH
Range=1.03e+01
Mean=3.57e+00
Median=3.46e+00
Rel. SD=0.41
Skewness=0.50
S.VLDL.FC
Range=8.76e+00
Mean=2.13e+00
Median=1.85e+00
Rel. SD=0.58
Skewness=1.55
S.VLDL.PL
Range=5.12e+00
Mean=6.45e-01
Median=6.00e-01
Rel. SD=0.49
Skewness=1.85
S.VLDL.TG
Range=9.26e+00
Mean=5.16e+00
Median=5.13e+00
Rel. SD=0.22
Skewness=0.19
XS.VLDL.PL
Range=2.77e+00
Mean=3.23e-01
Median=2.80e-01
Rel. SD=0.67
Skewness=1.91
XS.VLDL.TG
Range=4.23e+00
Mean=3.78e-01
Median=3.46e-01
Rel. SD=0.51
Skewness=3.70
ApoA1
Range=4.69e+00
Mean=8.92e-01
Median=8.52e-01
Rel. SD=0.32
Skewness=1.70
ApoB
Range=3.58e+00
Mean=8.05e-01
Median=7.07e-01
Rel. SD=0.52
Skewness=1.57
Plasma.C
Range=1.21e+00
Mean=4.46e-01
Median=4.36e-01
Rel. SD=0.39
Skewness=0.38
Plasma.TG
Range=1.32e+00
Mean=1.05e+00
Median=1.06e+00
Rel. SD=0.14
Skewness=-0.16
400
200
0
Area / a.u.

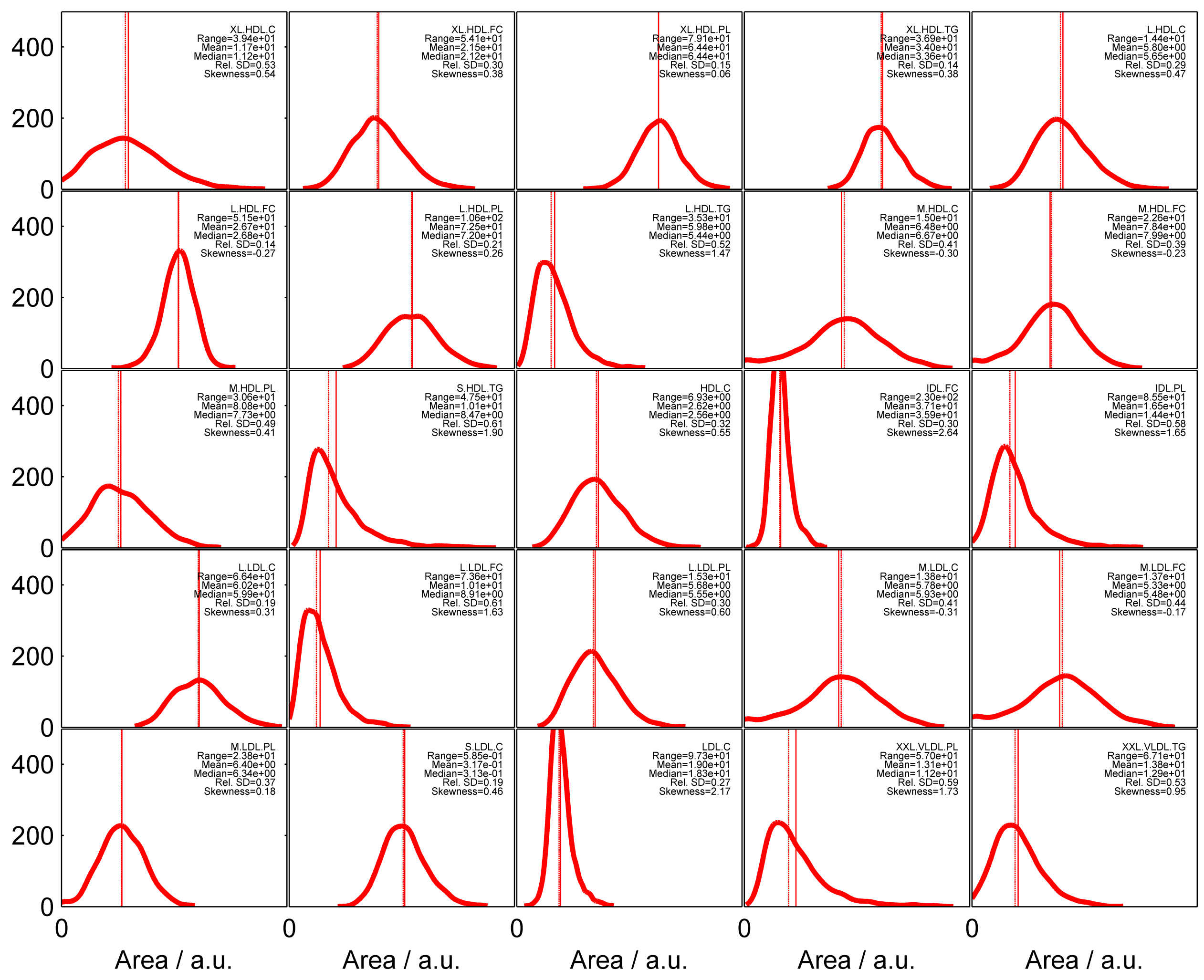
XL.HDL.C
Range=3.94e+01
Mean=1.17e+01
Median=1.12e+01
Rel. SD=0.53
Skewness=0.54
XL.HDL.FC
Range=5.41e+01
Mean=2.15e+01
Median=2.12e+01
Rel. SD=0.30
Skewness=0.38
XL.HDL.PL
Range=7.91e+01
Mean=6.44e+01
Median=6.44e+01
Rel. SD=0.15
Skewness=0.06
XL.HDL.TG
Range=3.69e+01
Mean=3.40e+01
Median=3.36e+01
Rel. SD=0.14
Skewness=0.38
L.HDL.C
Range=1.44e+01
Mean=5.80e+00
Median=5.65e+00
Rel. SD=0.29
Skewness=0.47
L.HDL.FC
Range=5.15e+01
Mean=2.67e+01
Median=2.68e+01
Rel. SD=0.14
Skewness=-0.27
L.HDL.PL
Range=1.06e+02
Mean=7.25e+01
Median=7.20e+01
Rel. SD=0.21
Skewness=0.26
L.HDL.TG
Range=3.53e+01
Mean=5.98e+00
Median=5.44e+00
Rel. SD=0.52
Skewness=1.47
M.HDL.C
Range=1.50e+01
Mean=6.48e+00
Median=6.67e+00
Rel. SD=0.41
Skewness=-0.30
M.HDL.FC
Range=2.26e+01
Mean=7.84e+00
Median=7.99e+00
Rel. SD=0.39
Skewness=-0.23
M.HDL.PL
Range=3.06e+01
Mean=8.08e+00
Median=7.73e+00
Rel. SD=0.49
Skewness=0.41
S.HDL.TG
Range=4.75e+01
Mean=1.01e+01
Median=8.47e+00
Rel. SD=0.61
Skewness=1.90
HDL.C
Range=6.93e+00
Mean=2.62e+00
Median=2.56e+00
Rel. SD=0.32
Skewness=0.55
IDL.FC
Range=2.30e+02
Mean=3.71e+01
Median=3.59e+01
Rel. SD=0.30
Skewness=2.64
IDL.PL
Range=8.55e+01
Mean=1.65e+01
Median=1.44e+01
Rel. SD=0.58
Skewness=1.65
L.LDL.C
Range=6.64e+01
Mean=6.02e+01
Median=5.99e+01
Rel. SD=0.19
Skewness=0.31
L.LDL.FC
Range=7.36e+01
Mean=1.01e+01
Median=8.91e+00
Rel. SD=0.61
Skewness=1.63
L.LDL.PL
Range=1.53e+01
Mean=5.68e+00
Median=5.55e+00
Rel. SD=0.30
Skewness=0.60
M.LDL.C
Range=1.38e+01
Mean=5.78e+00
Median=5.93e+00
Rel. SD=0.41
Skewness=-0.31
M.LDL.FC
Range=1.37e+01
Mean=5.33e+00
Median=5.48e+00
Rel. SD=0.44
Skewness=-0.17
M.LDL.PL
Range=2.38e+01
Mean=6.40e+00
Median=6.34e+00
Rel. SD=0.37
Skewness=0.18
S.LDL.C
Range=5.85e-01
Mean=3.17e-01
Median=3.13e-01
Rel. SD=0.19
Skewness=0.46
LDL.C
Range=9.73e+01
Mean=1.90e+01
Median=1.83e+01
Rel. SD=0.27
Skewness=2.17
XXL.VLDL.PL
Range=5.70e+01
Mean=1.31e+01
Median=1.12e+01
Rel. SD=0.59
Skewness=1.73
XXL.VLDL.TG
Range=6.71e+01
Mean=1.38e+01
Median=1.29e+01
Rel. SD=0.53
Skewness=0.95
400
200
0
Area / a.u.

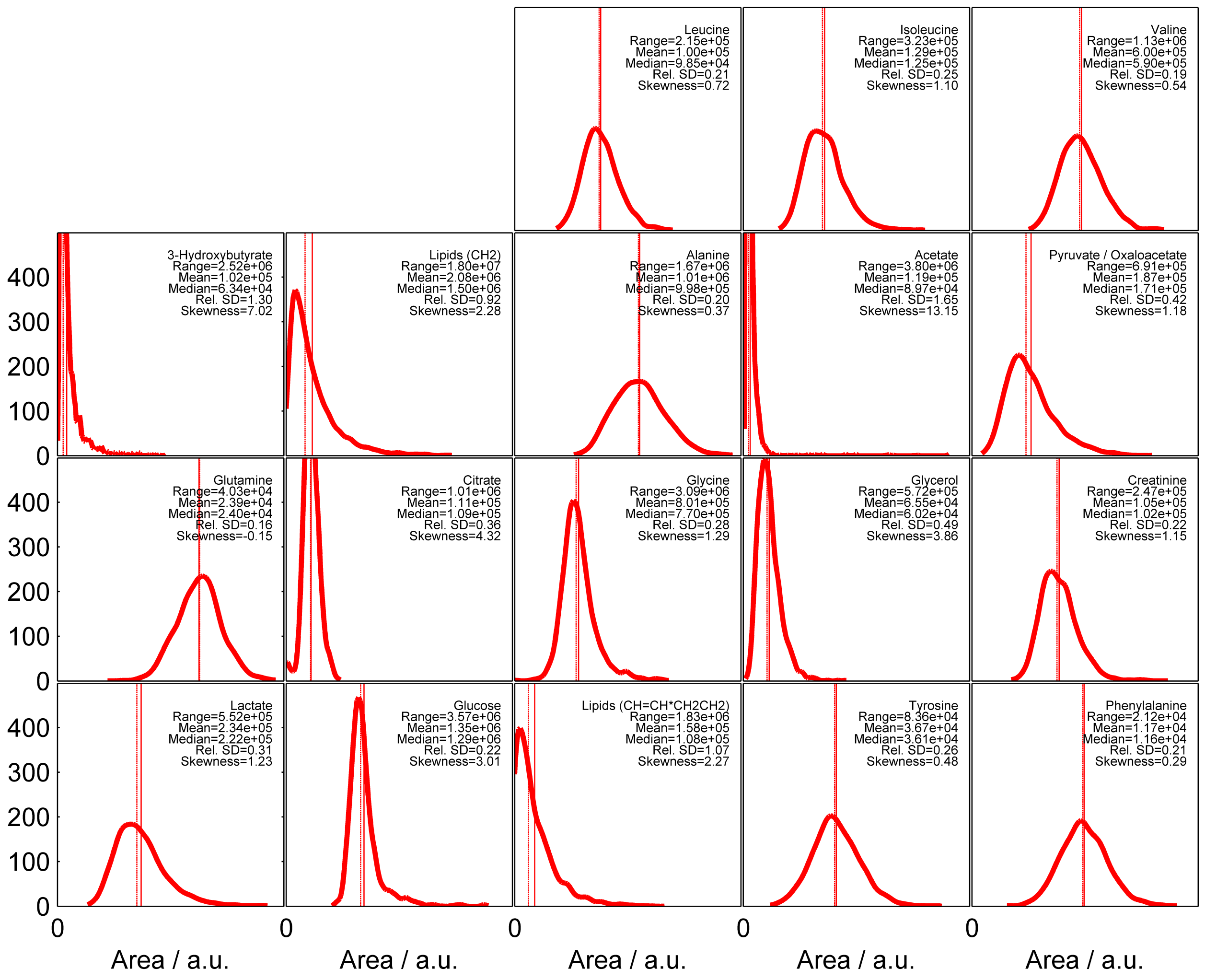

Leucine
Range=2.15e+05
Mean=1.00e+05
Median=9.85e+04
Rel. SD=0.21
Skewness=0.72
Isoleucine
Range=3.23e+05
Mean=1.29e+05
Median=1.25e+05
Rel. SD=0.25
Skewness=1.10
Valine
Range=1.13e+06
Mean=6.00e+05
Median=5.90e+05
Rel. SD=0.19
Skewness=0.54
3-Hydroxybutyrate
Range=2.52e+06
Mean=1.02e+05
Median=6.34e+04
Rel. SD=1.30
Skewness=7.02
Lipids (CH2)
Range=1.80e+07
Mean=2.08e+06
Median=1.50e+06
Rel. SD=0.92
Skewness=2.28
Alanine
Range=1.67e+06
Mean=1.01e+06
Median=9.98e+05
Rel. SD=0.20
Skewness=0.37
Acetate
Range=3.80e+06
Mean=1.19e+05
Median=8.97e+04
Rel. SD=1.65
Skewness=13.15
Pyruvate / Oxaloacetate
Range=6.91e+05
Mean=1.87e+05
Median=1.71e+05
Rel. SD=0.42
Skewness=1.18
Glutamine
Range=4.03e+04
Mean=2.39e+04
Median=2.40e+04
Rel. SD=0.16
Skewness=-0.15
Citrate
Range=1.01e+06
Mean=1.11e+05
Median=1.09e+05
Rel. SD=0.36
Skewness=4.32
Glycine
Range=3.09e+06
Mean=8.01e+05
Median=7.70e+05
Rel. SD=0.28
Skewness=1.29
Glycerol
Range=5.72e+05
Mean=6.55e+04
Median=6.02e+04
Rel. SD=0.49
Skewness=3.86
Creatinine
Range=2.47e+05
Mean=1.05e+05
Median=1.02e+05
Rel. SD=0.22
Skewness=1.15
Lactate
Range=5.52e+05
Mean=2.34e+05
Median=2.22e+05
Rel. SD=0.31
Skewness=1.23
Glucose
Range=3.57e+06
Mean=1.35e+06
Median=1.29e+06
Rel. SD=0.22
Skewness=3.01
Lipids (CH=CH*CH2CH2)
Range=1.83e+06
Mean=1.58e+05
Median=1.08e+05
Rel. SD=1.07
Skewness=2.27
Tyrosine
Range=8.36e+04
Mean=3.67e+04
Median=3.61e+04
Rel. SD=0.26
Skewness=0.48
Phenylalanine
Range=2.12e+04
Mean=1.17e+04
Median=1.16e+04
Rel. SD=0.21
Skewness=0.29
0
100
200
300
400
Area / a.u.

Supplementary Figure 5. Quantile plots for all traits. The p-value has been truncated at 1 x 10^{-14} to allow the visualization of over all inflation, i.e. observed distribution departing from expected already at large p-value distribution. The smallest p-value of the distribution is given in the upper part of each figure.

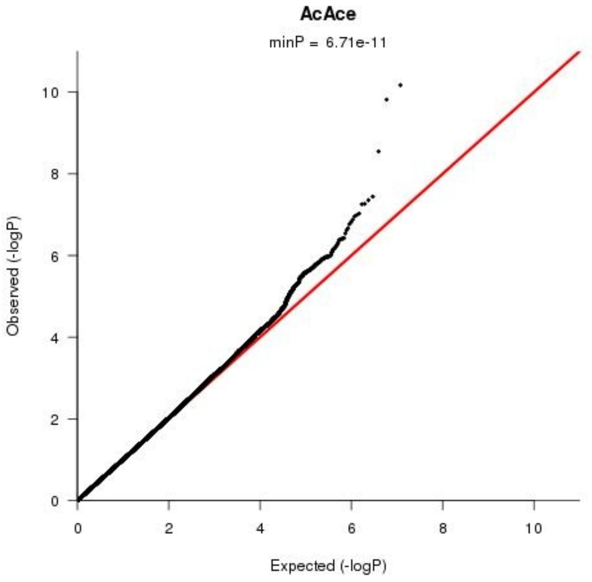
AcAce
minP = 6.71e-11
Observed (-logP)
Expected (-logP)

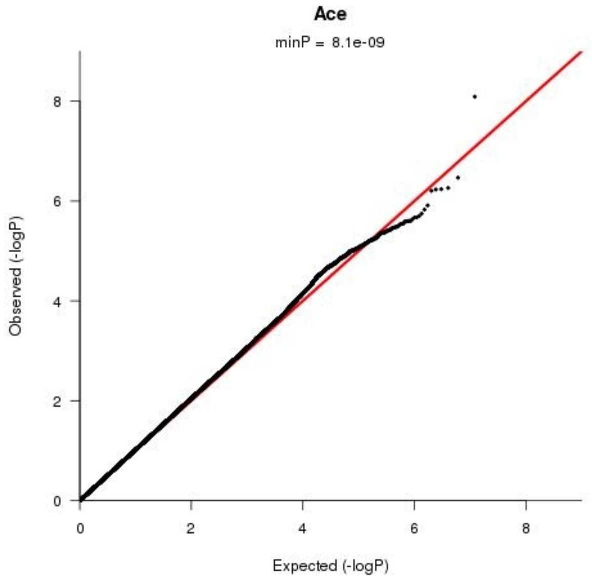
Ace
minP = 8.1e-09
Observed (-logP)
Expected (-logP)

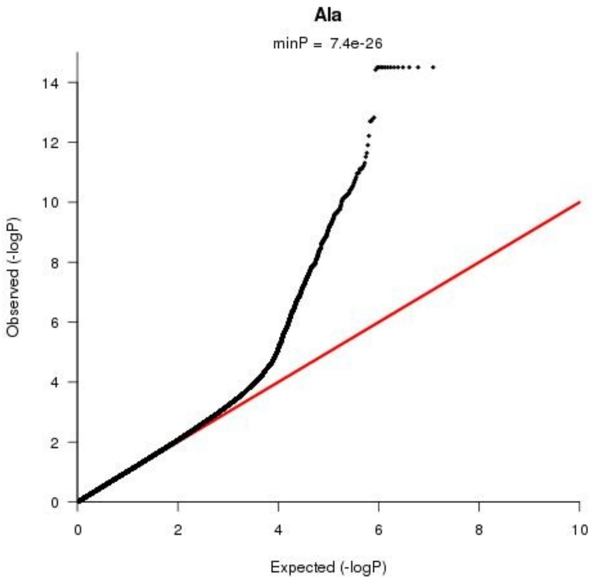
Ala
minP = 7.4e-26
Observed (-logP)
Expected (-logP)

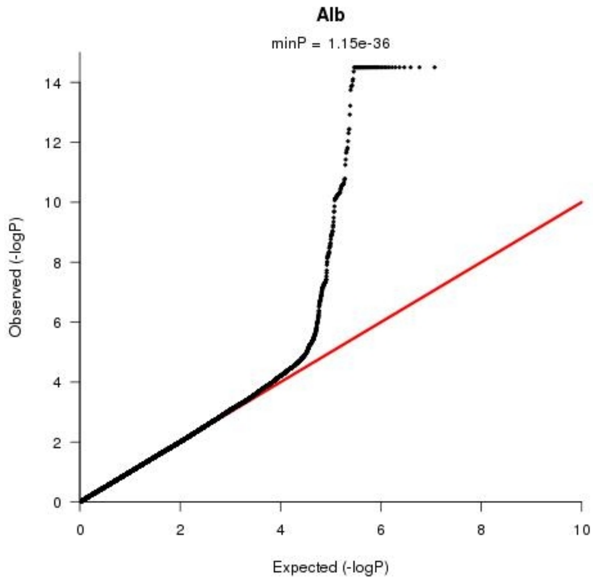
Alb
minP = 1.15e-36
Observed (-logP)
Expected (-logP)

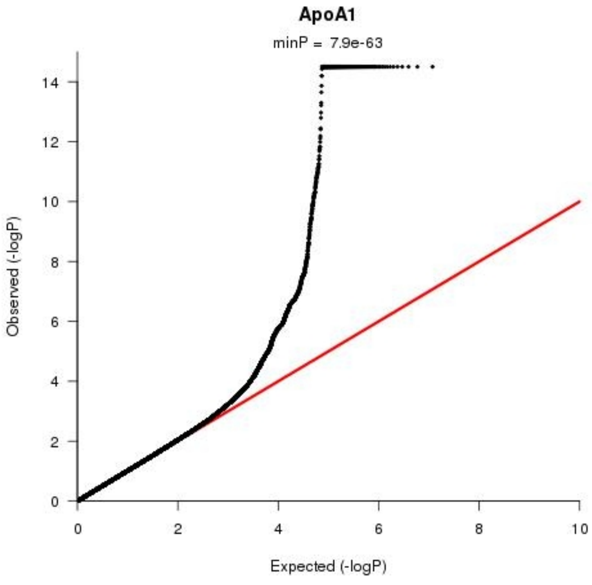
ApoA1
minP = 7.9e-63
Observed (-logP)
Expected (-logP)

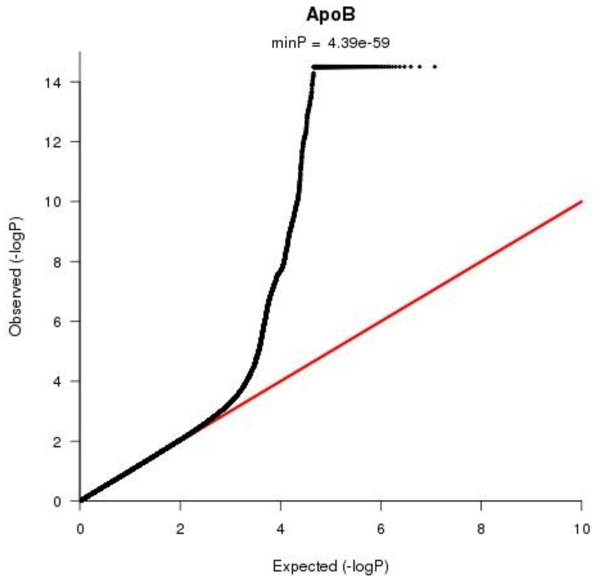
ApoB
minP = 4.39e-59
Observed (-logP)
Expected (-logP)

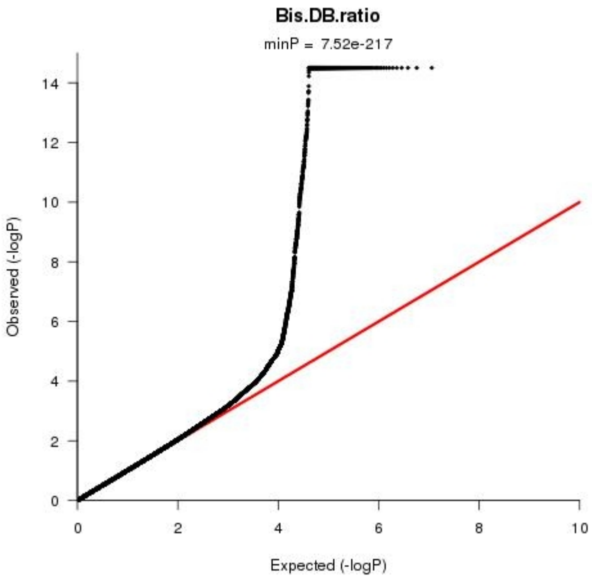
Bis.DB.ratio
minP = 7.52e-217
Observed (-logP)
Expected (-logP)

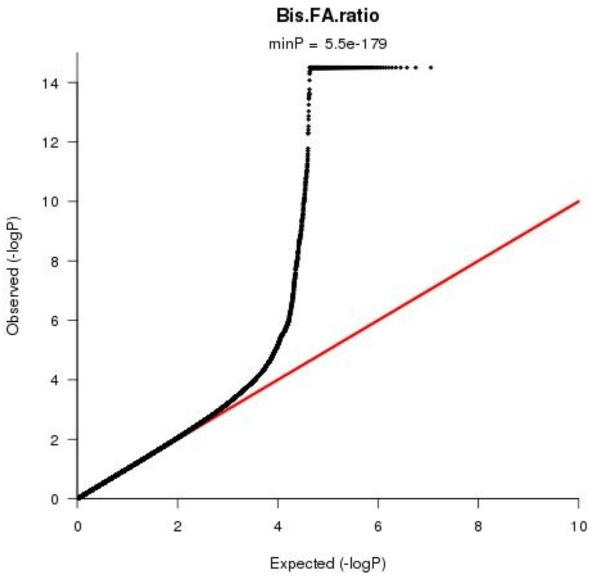
Bis.FA.ratio
minP = 5.5e-179
Observed (-logP)
Expected (-logP)

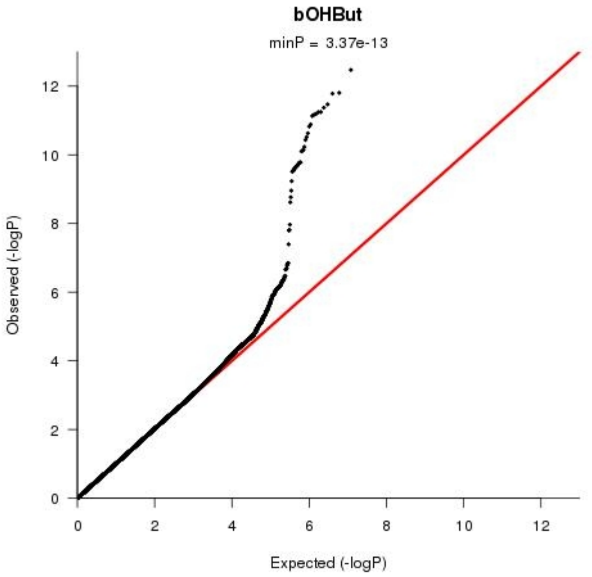
bOHBut
minP = 3.37e-13
Observed (-logP)
Expected (-logP)

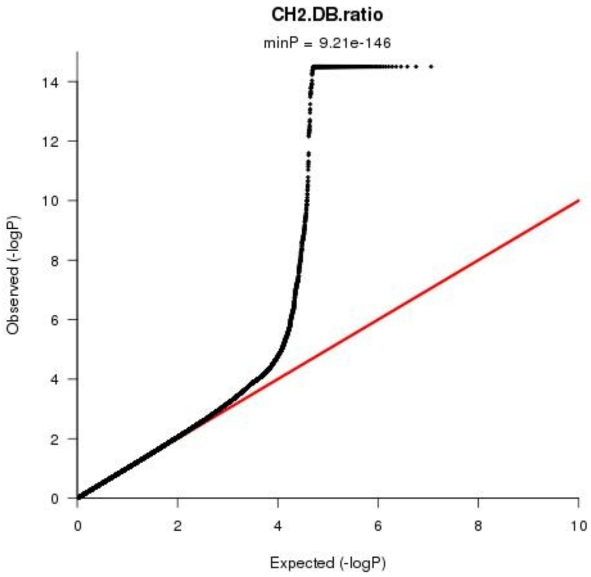
CH2.DB.ratio
minP = 9.21e-146
Observed (-logP)
Expected (-logP)

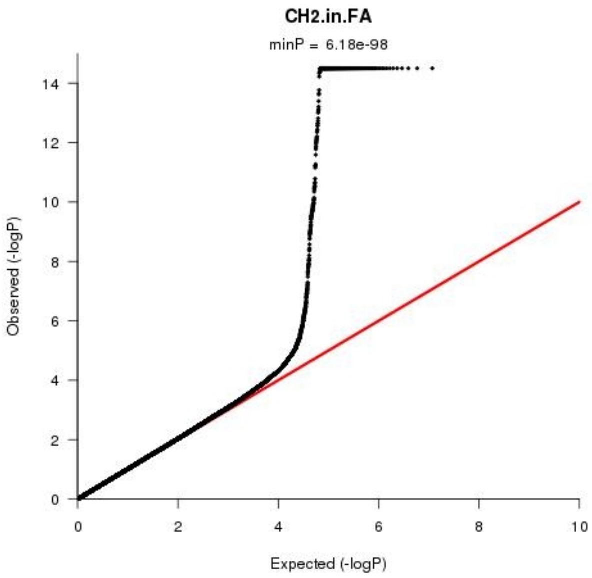
CH2.in.FA
minP = 6.18e-98
Observed (-logP)
Expected (-logP)

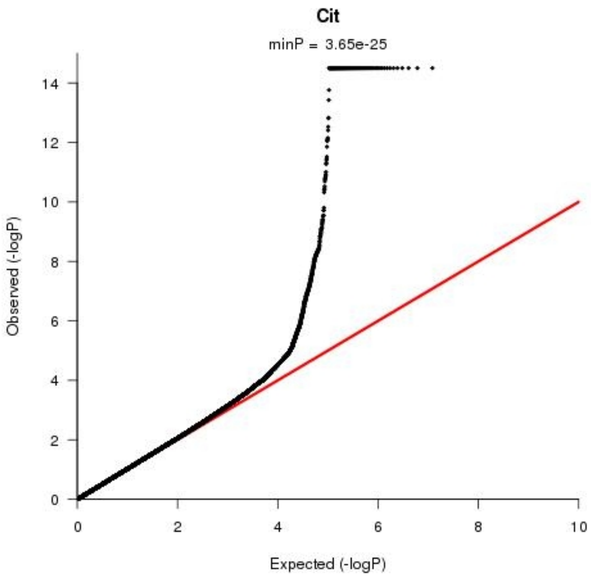
Cit
minP = 3.65e-25
Observed (-logP)
Expected (-logP)

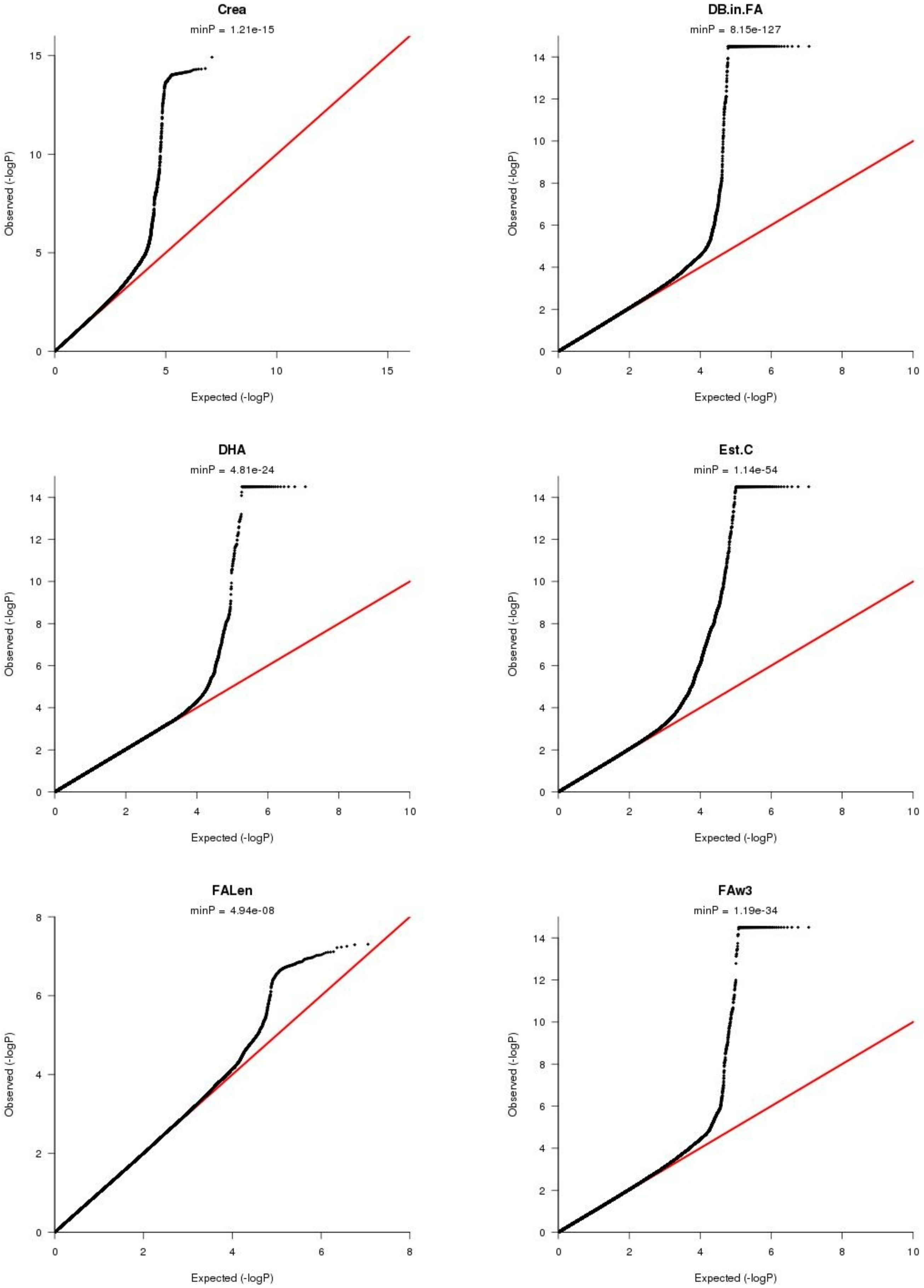
Crea
minP = 1.21e-15
DB.in.FA
minP = 8.15e-127
DHA
minP = 4.81e-24
Est.C
minP = 1.14e-54
FALen
minP = 4.94e-08
FAw3
minP = 1.19e-34
Observed (-logP)
Expected (-logP)

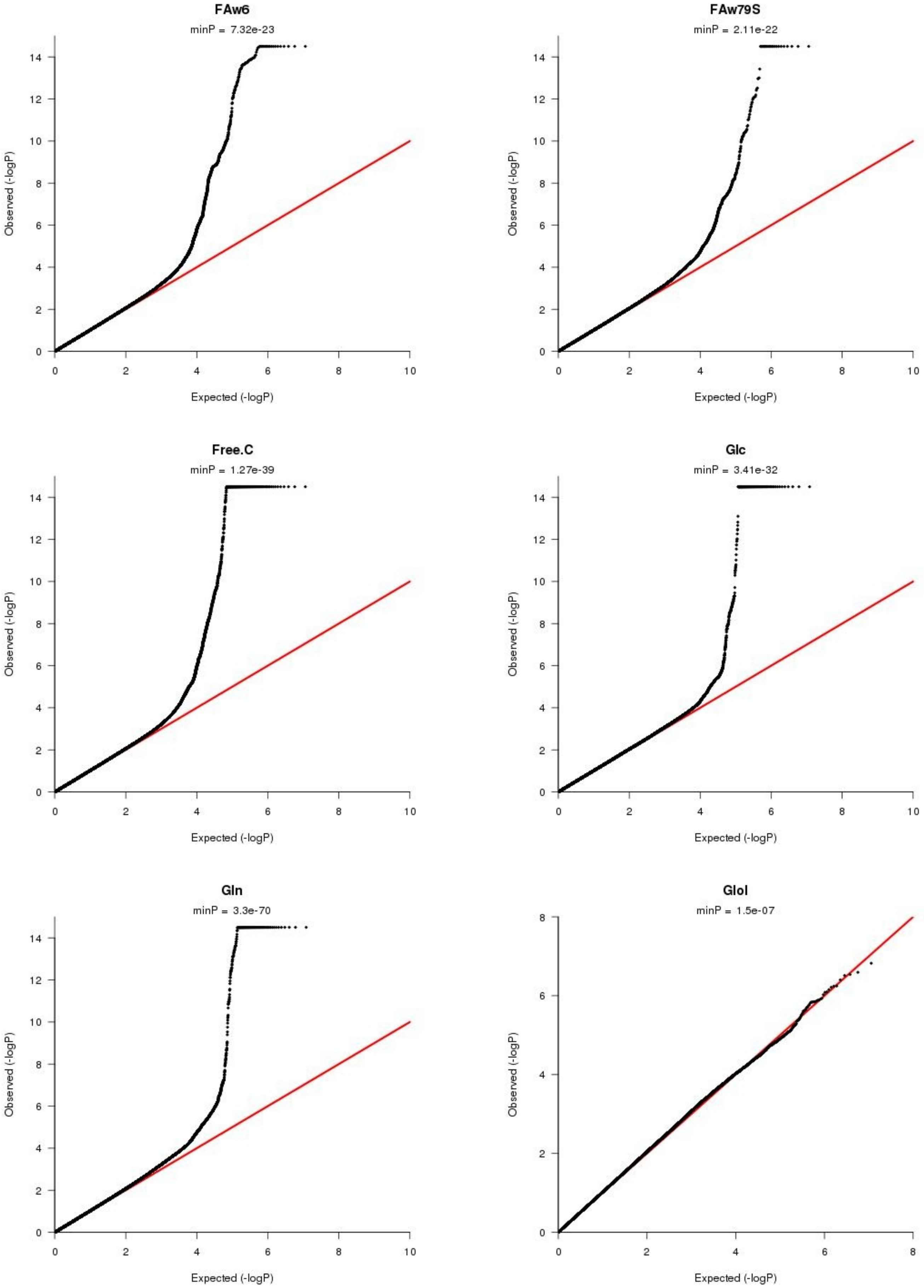
FAw6
minP = 7.32e-23
FAw79S
minP = 2.11e-22
Free.C
minP = 1.27e-39
Glc
minP = 3.41e-32
Gln
minP = 3.3e-70
Glol
minP = 1.5e-07
Observed (-logP)
Expected (-logP)

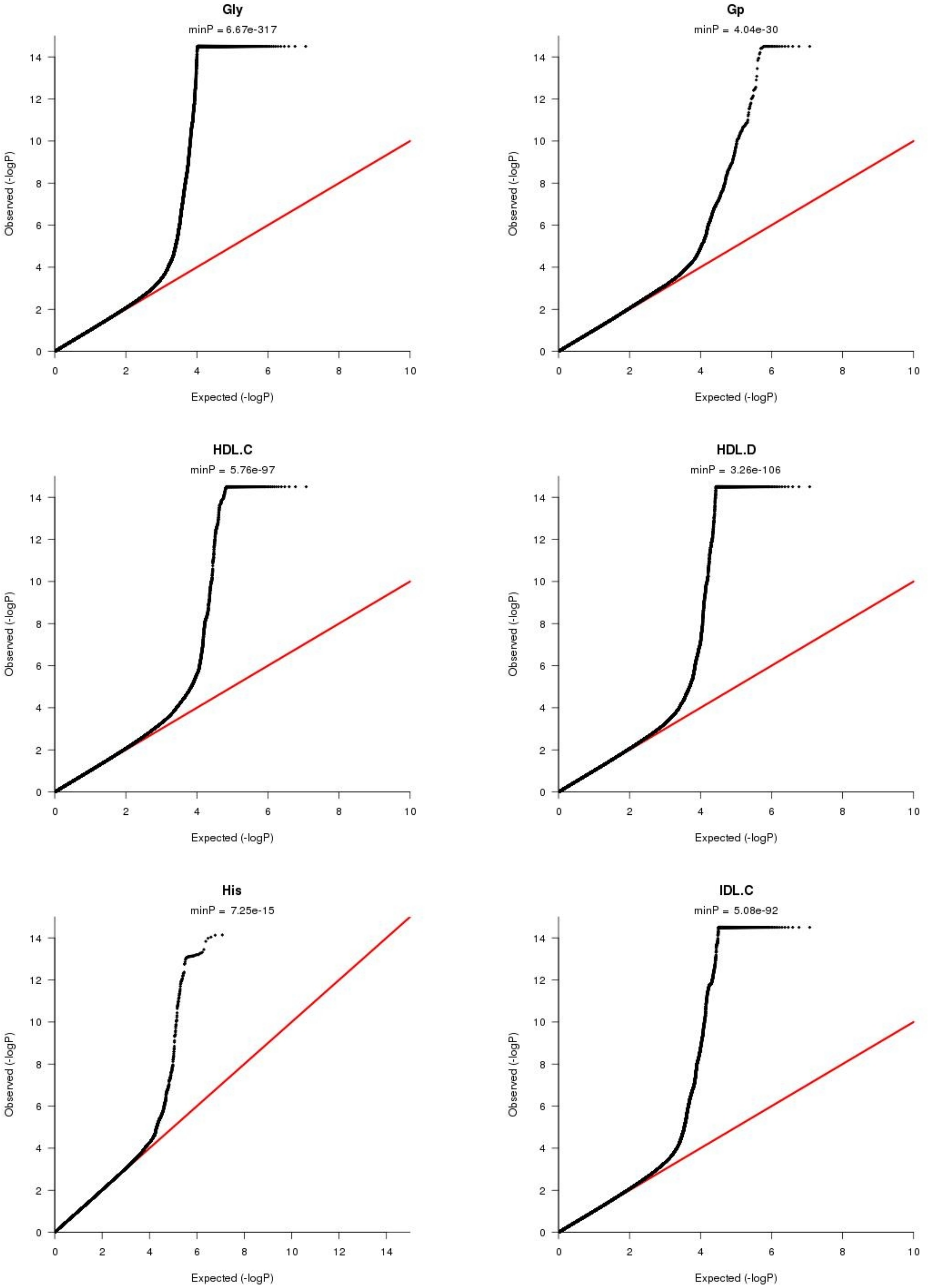
Gly
minP = 6.67e-317
Gp
minP = 4.04e-30
HDL.C
minP = 5.76e-97
HDL.D
minP = 3.26e-106
His
minP = 7.25e-15
IDL.C
minP = 5.08e-92
Observed (-logP)
Expected (-logP)

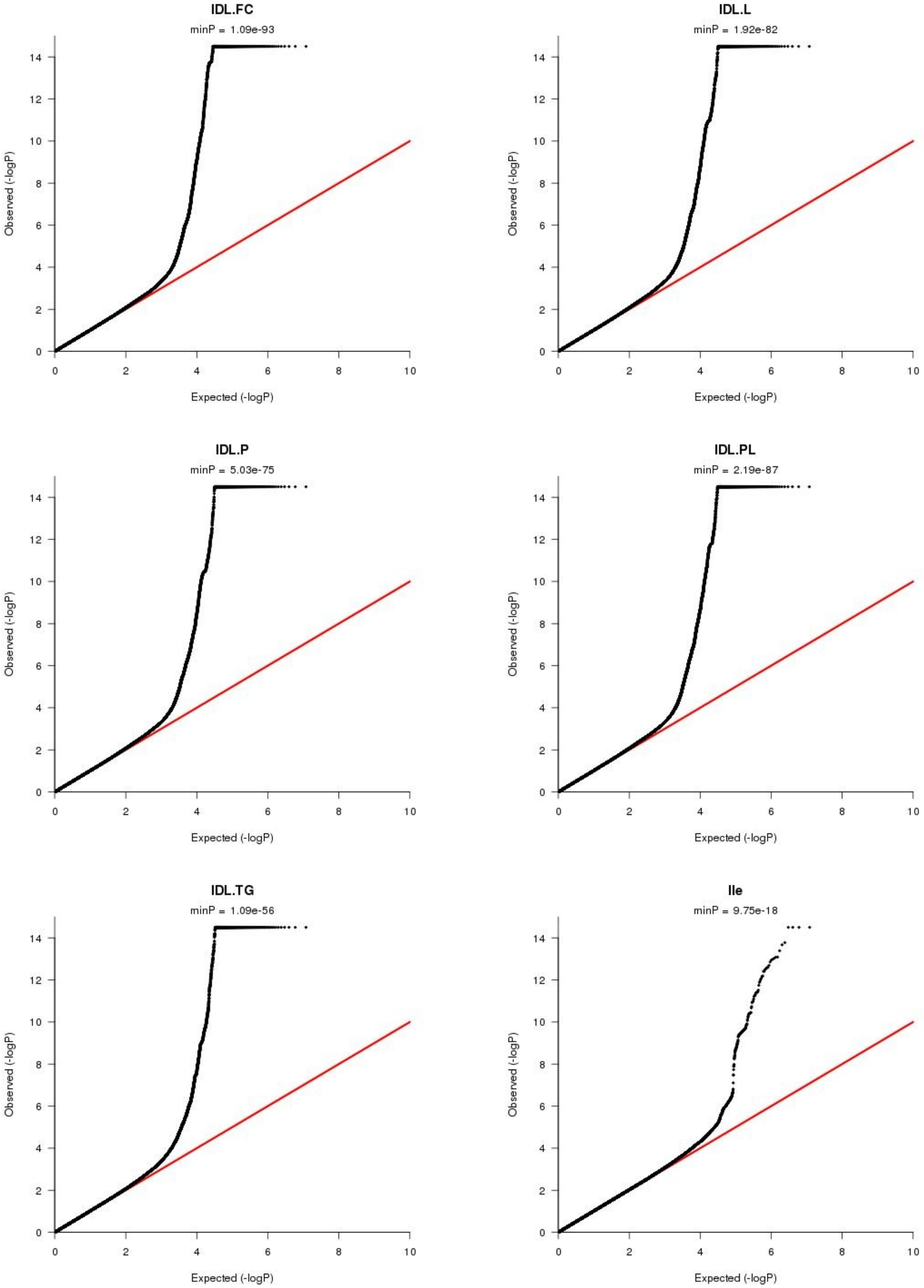
IDL.FC
minP = 1.09e-93
IDL.L
minP = 1.92e-82
IDL.P
minP = 5.03e-75
IDL.PL
minP = 2.19e-87
IDL.TG
minP = 1.09e-56
Ile
minP = 9.75e-18
Observed (-logP)
Expected (-logP)

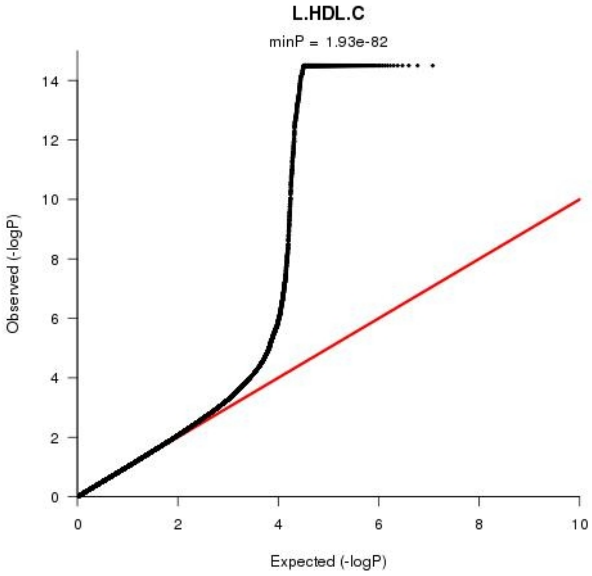
L.HDL.C
minP = 1.93e-82
Observed (-logP)
Expected (-logP)

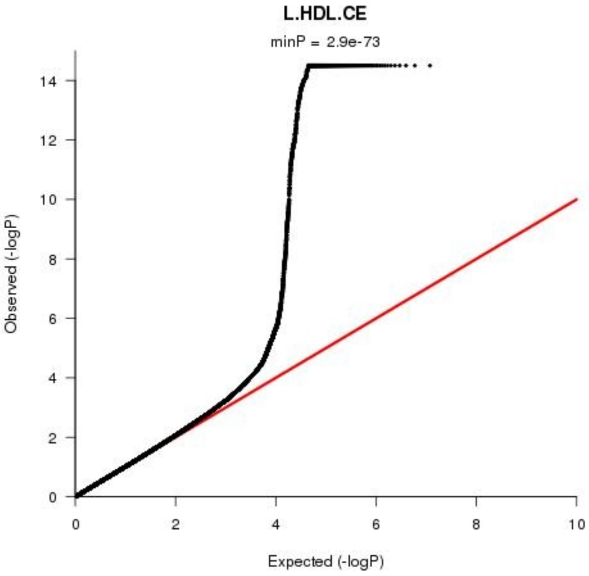
L.HDL.CE
minP = 2.9e-73
Observed (-logP)
Expected (-logP)

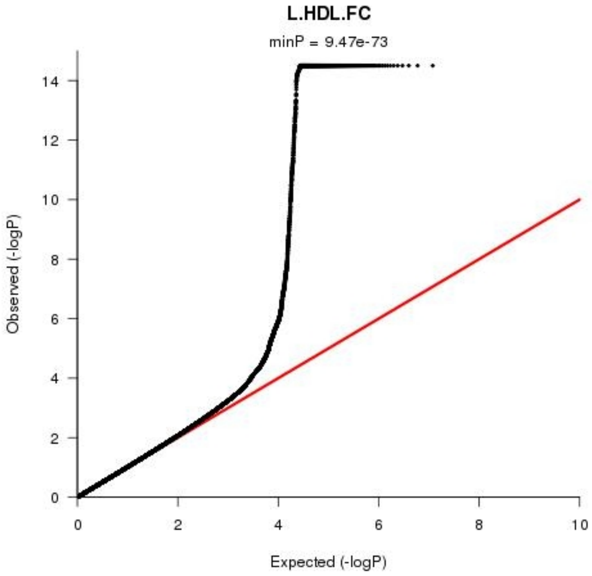
L.HDL.FC
minP = 9.47e-73
Observed (-logP)
Expected (-logP)

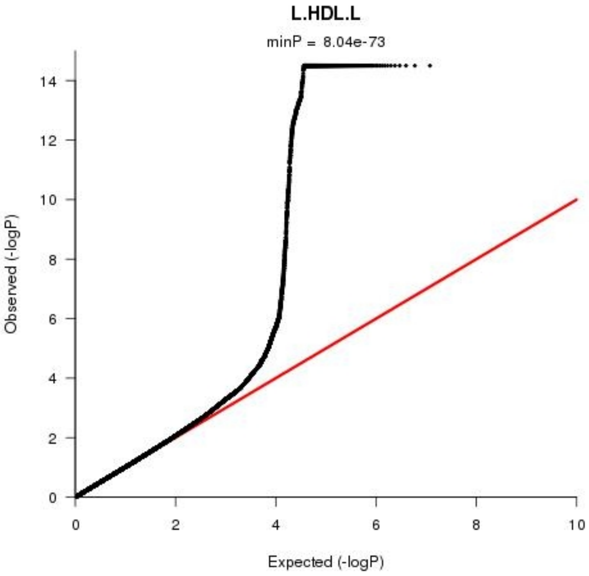
L.HDL.L
minP = 8.04e-73
Observed (-logP)
Expected (-logP)

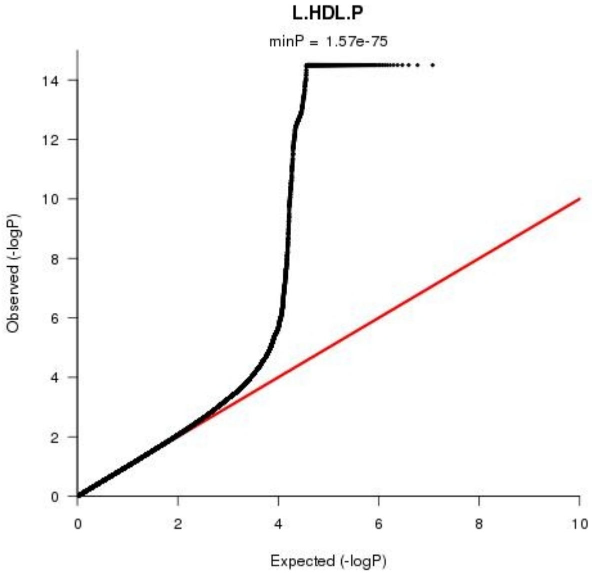
L.HDL.P
minP = 1.57e-75
Observed (-logP)
Expected (-logP)

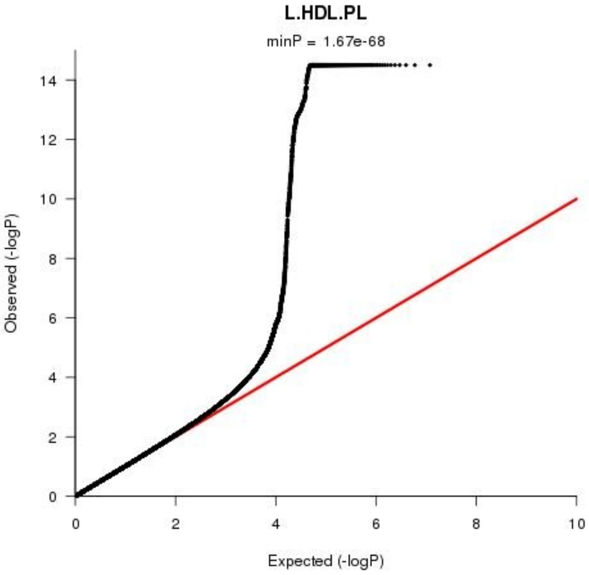
L.HDL.PL
minP = 1.67e-68
Observed (-logP)
Expected (-logP)

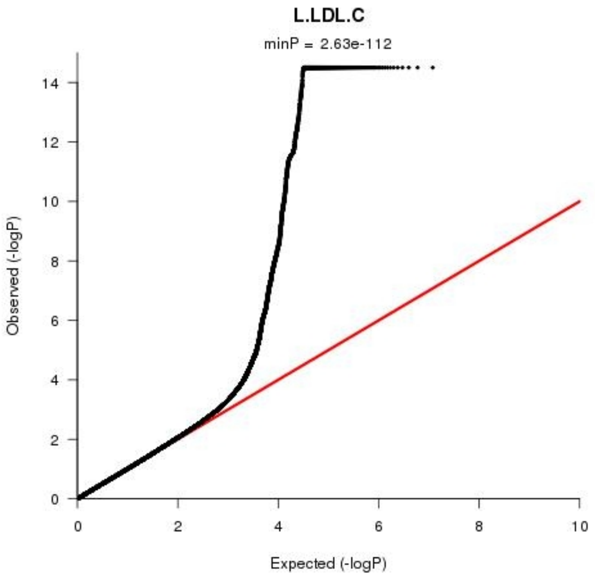
L.LDL.C
minP = 2.63e-112
Observed (-logP)
Expected (-logP)

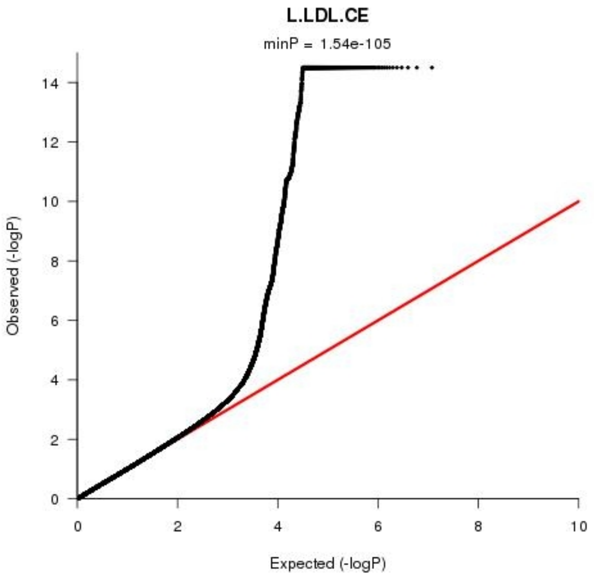
L.LDL.CE
minP = 1.54e-105
Observed (-logP)
Expected (-logP)

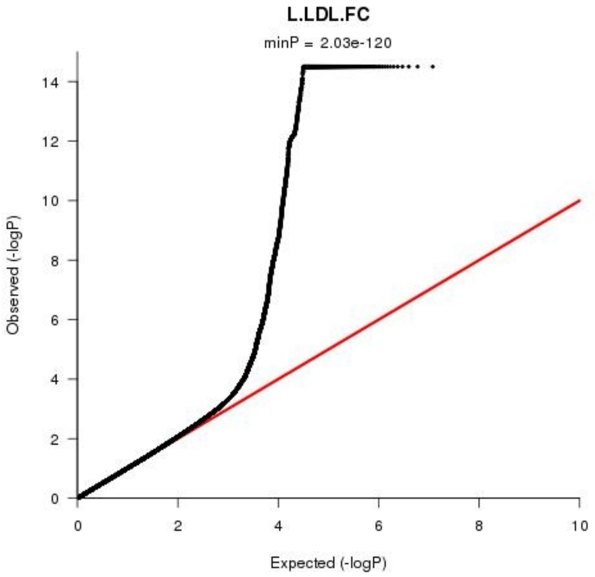
L.LDL.FC
minP = 2.03e-120
Observed (-logP)
Expected (-logP)

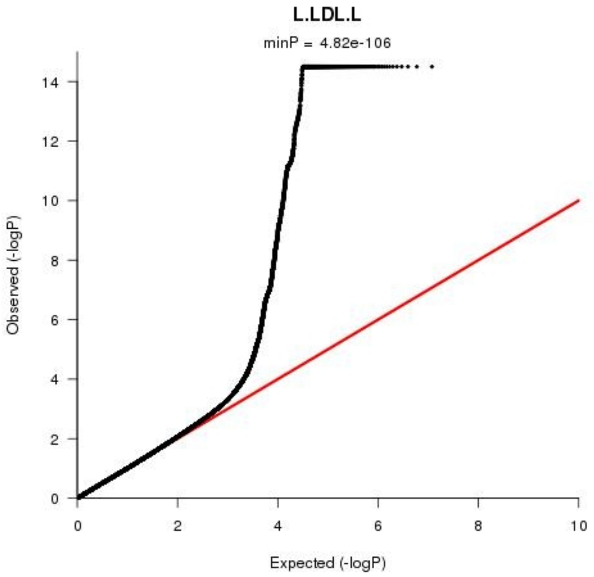
L.LDL.L
minP = 4.82e-106
Observed (-logP)
Expected (-logP)

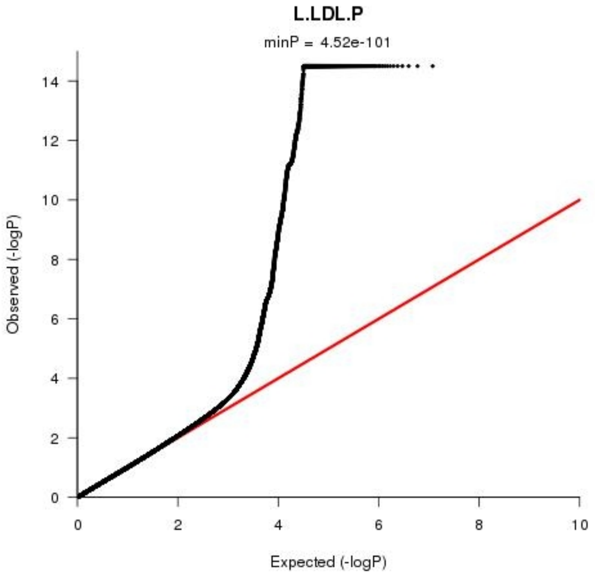
L.LDL.P
minP = 4.52e-101
Observed (-logP)
Expected (-logP)

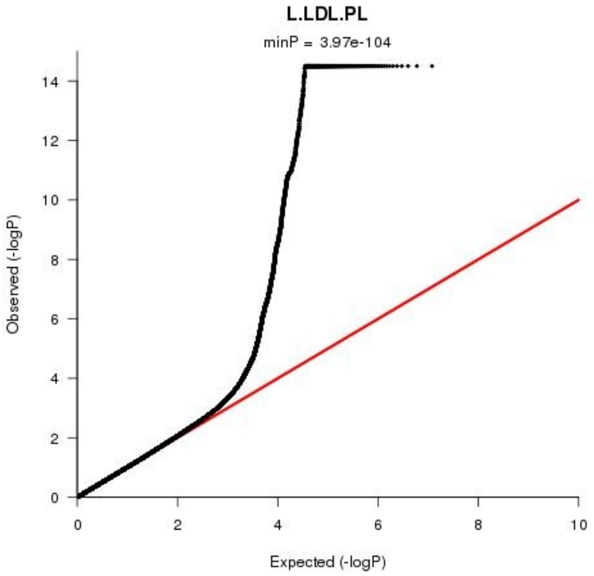
L.LDL.PL
minP = 3.97e-104
Observed (-logP)
Expected (-logP)

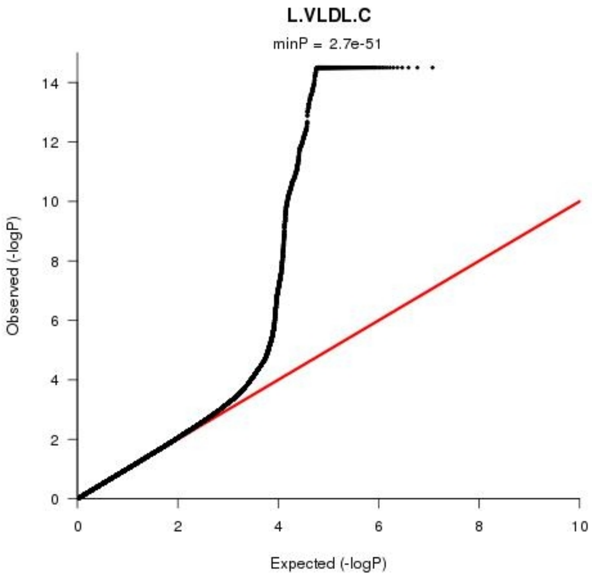
L.VLDL.C
minP = 2.7e-51
Observed (-logP)
Expected (-logP)
0
2
4
6
8
10
12
14

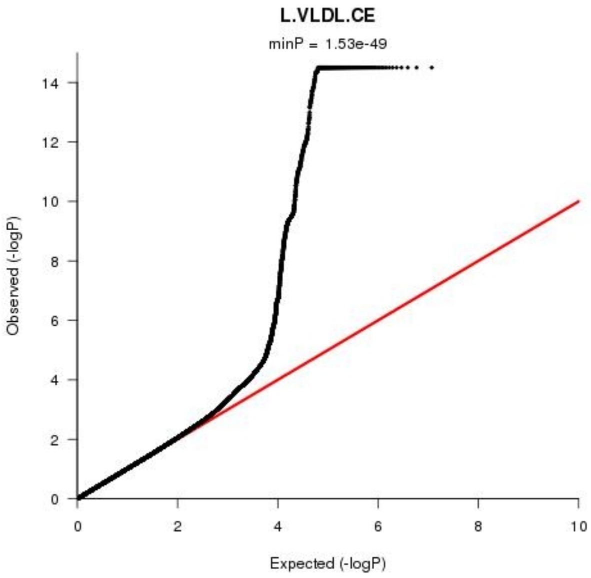
L.VLDL.CE
minP = 1.53e-49
Observed (-logP)
Expected (-logP)
0
2
4
6
8
10
12
14

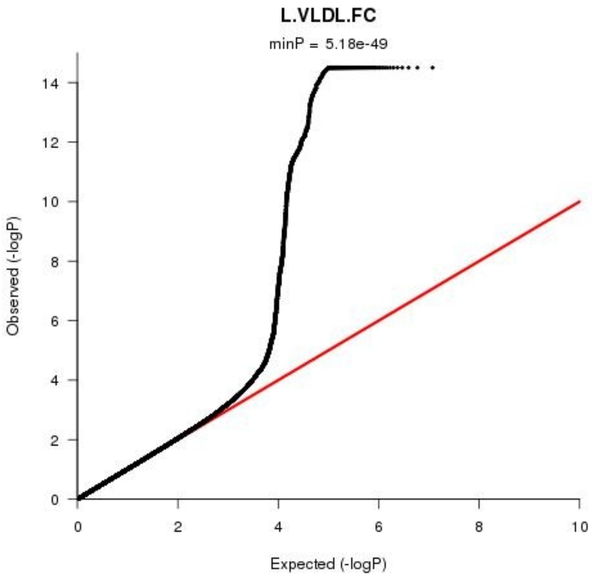
L.VLDL.FC
minP = 5.18e-49
Observed (-logP)
Expected (-logP)
0
2
4
6
8
10
12
14

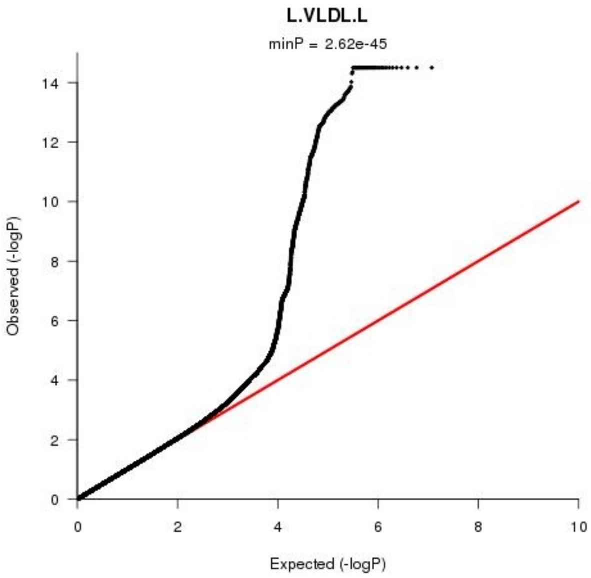
L.VLDL.L
minP = 2.62e-45
Observed (-logP)
Expected (-logP)
0
2
4
6
8
10
12
14

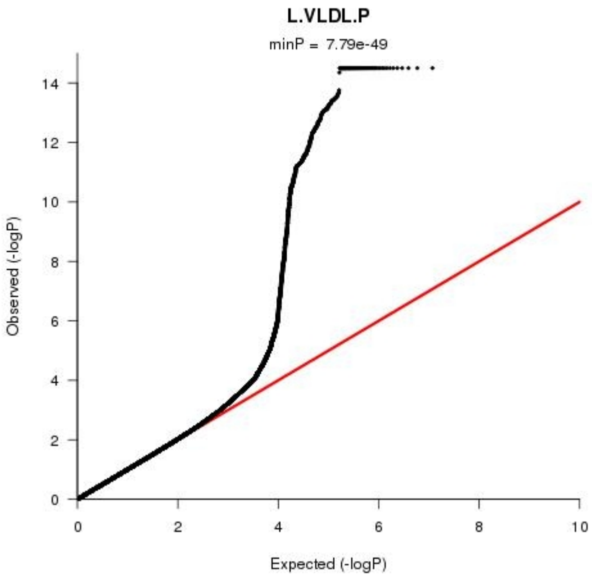
L.VLDL.P
minP = 7.79e-49
Observed (-logP)
Expected (-logP)
0
2
4
6
8
10
12
14

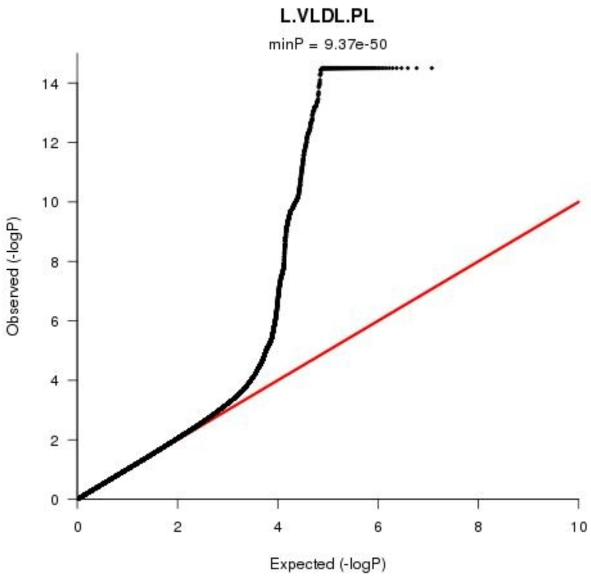
L.VLDL.PL
minP = 9.37e-50
Observed (-logP)
Expected (-logP)
0
2
4
6
8
10
12
14

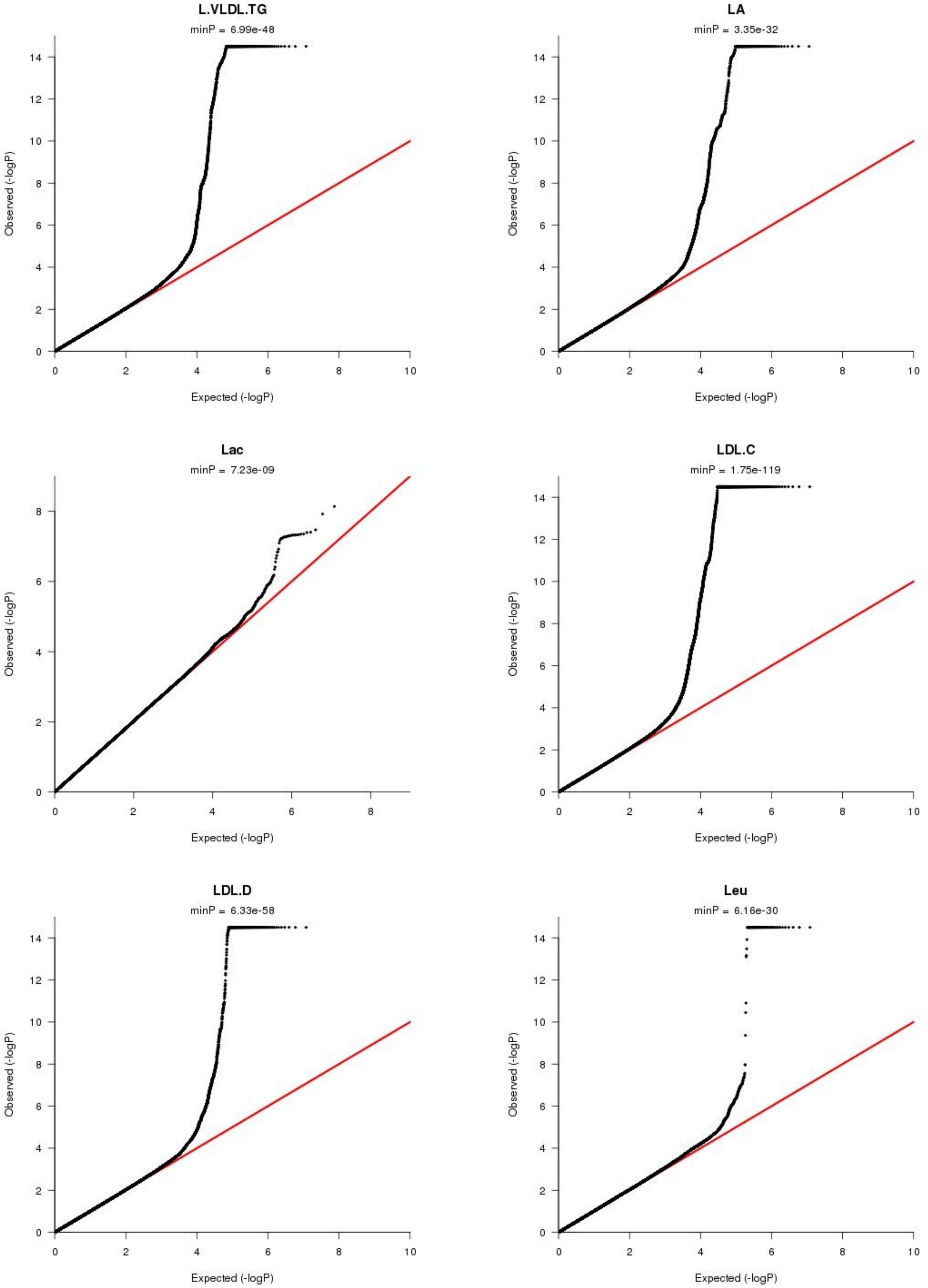
L.VLDL.TG
minP = 6.99e-48
LA
minP = 3.35e-32
Lac
minP = 7.23e-09
LDL.C
minP = 1.75e-119
LDL.D
minP = 6.33e-58
Leu
minP = 6.16e-30
Observed (-logP)
Expected (-logP)

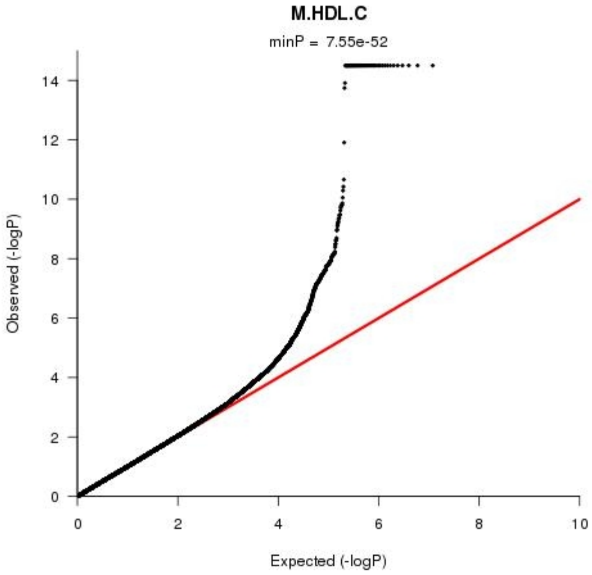
M.HDL.C
minP = 7.55e-52
Observed (-logP)
Expected (-logP)

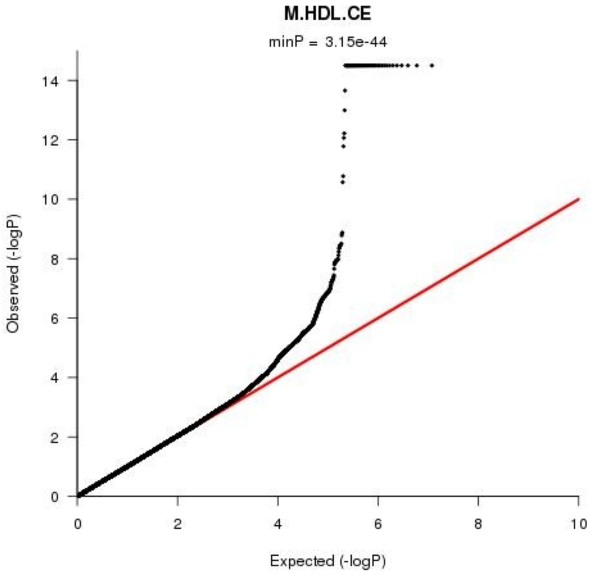
M.HDL.CE
minP = 3.15e-44
Observed (-logP)
Expected (-logP)

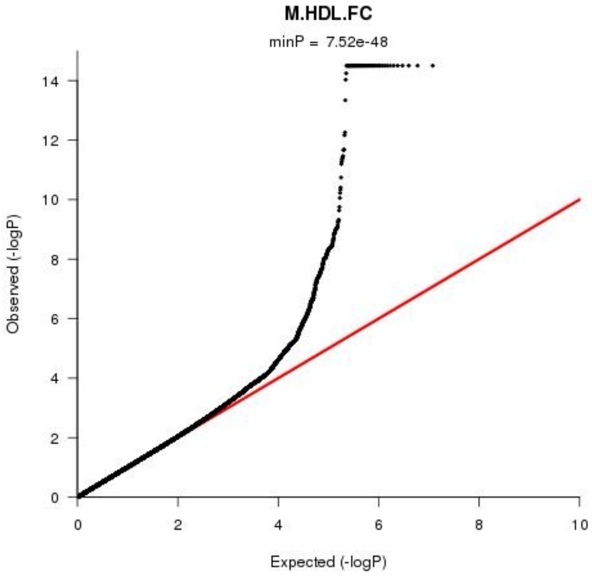
M.HDL.FC
minP = 7.52e-48
Observed (-logP)
Expected (-logP)

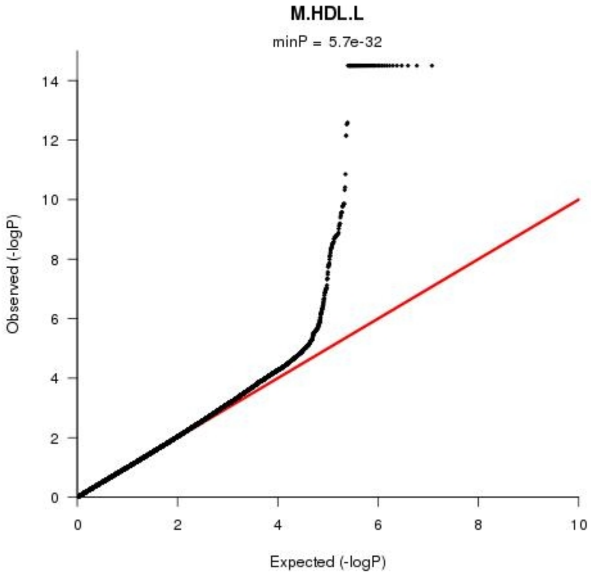
M.HDL.L
minP = 5.7e-32
Observed (-logP)
Expected (-logP)

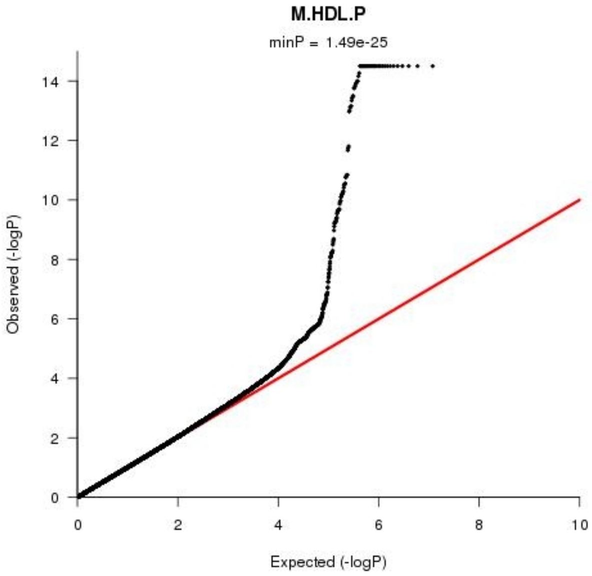
M.HDL.P
minP = 1.49e-25
Observed (-logP)
Expected (-logP)

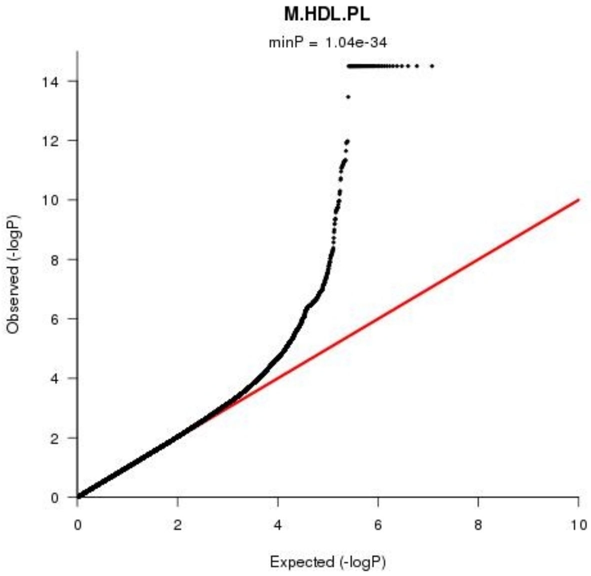
M.HDL.PL
minP = 1.04e-34
Observed (-logP)
Expected (-logP)

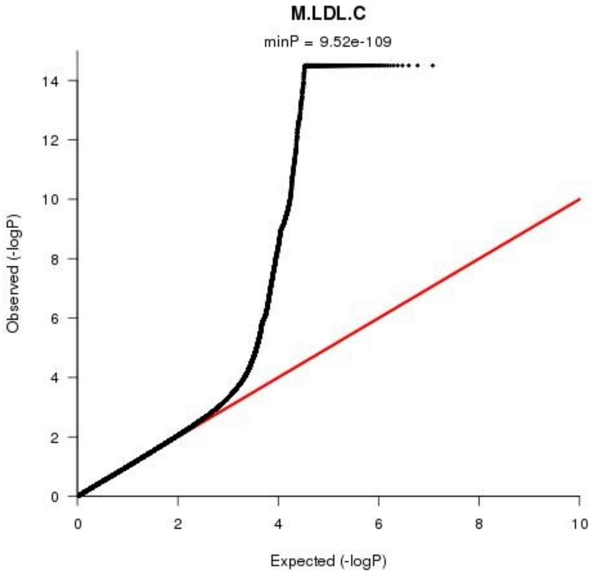
M.LDL.C
minP = 9.52e-109
Observed (-logP)
Expected (-logP)

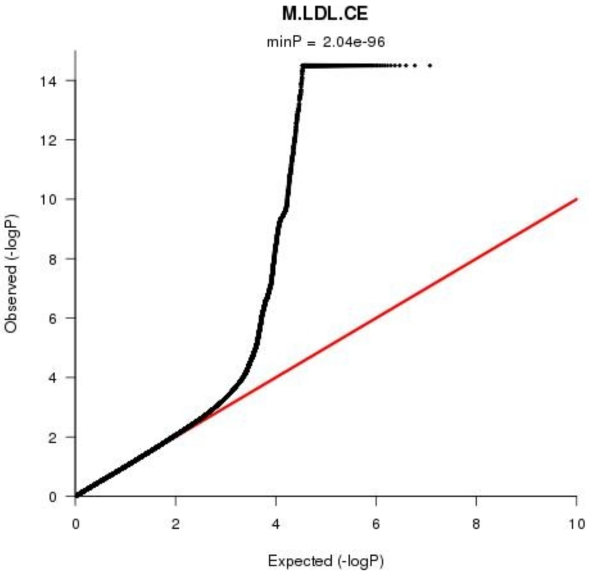
M.LDL.CE
minP = 2.04e-96
Observed (-logP)
Expected (-logP)

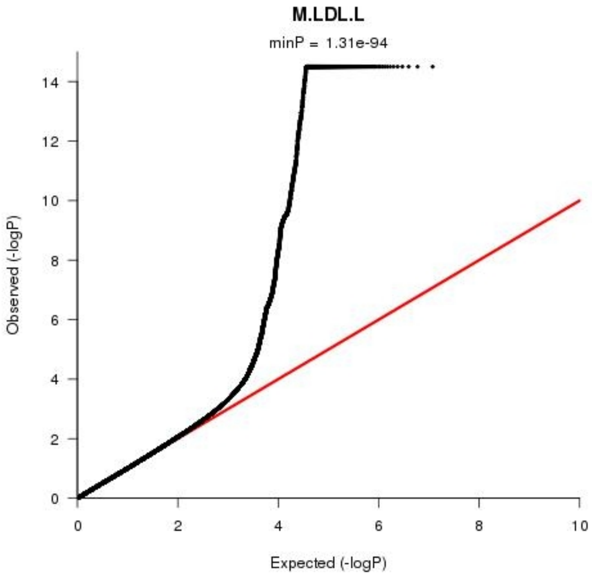
M.LDL.L
minP = 1.31e-94
Observed (-logP)
Expected (-logP)

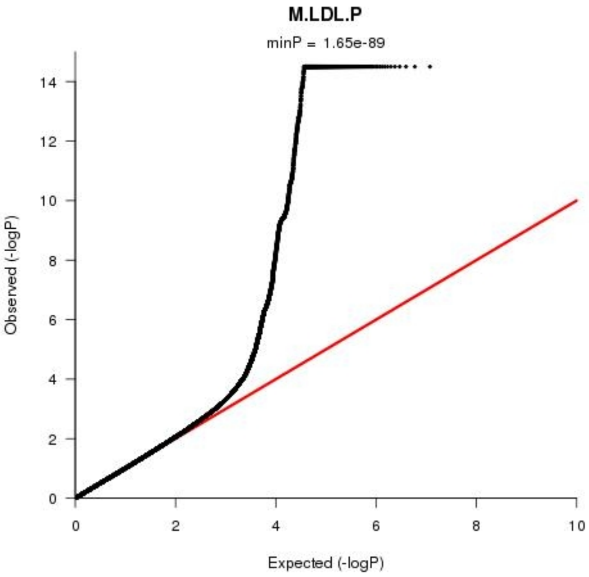
M.LDL.P
minP = 1.65e-89
Observed (-logP)
Expected (-logP)

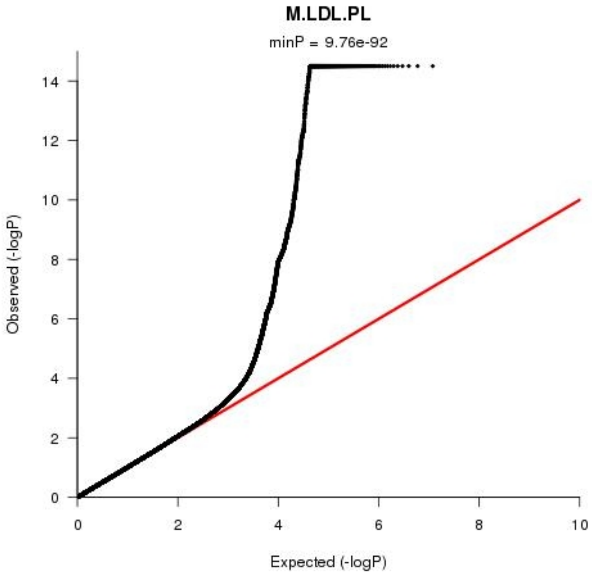
M.LDL.PL
minP = 9.76e-92
Observed (-logP)
Expected (-logP)

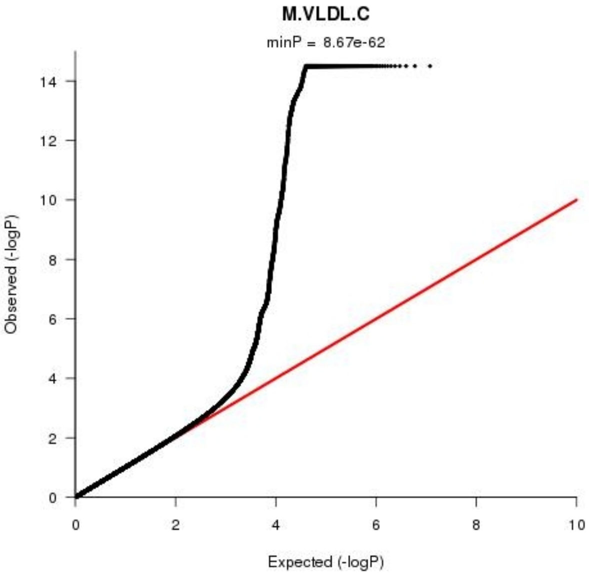
M.VLDL.C
minP = 8.67e-62
Observed (-logP)
Expected (-logP)

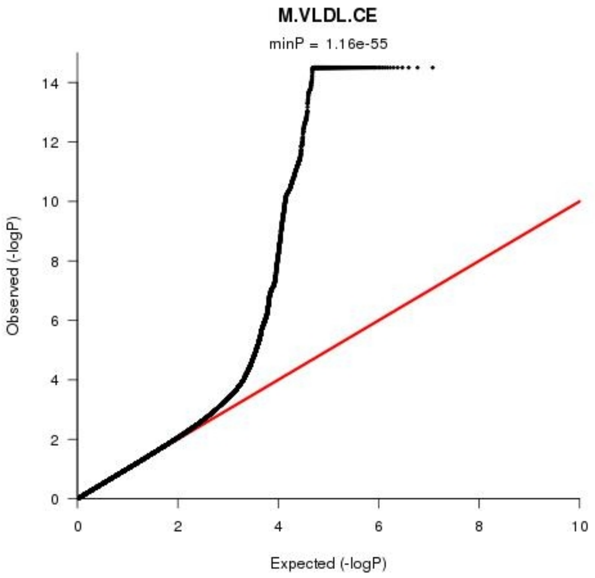
M.VLDL.CE
minP = 1.16e-55
Observed (-logP)
Expected (-logP)
0 2 4 6 8 10 12 14
0 2 4 6 8 10

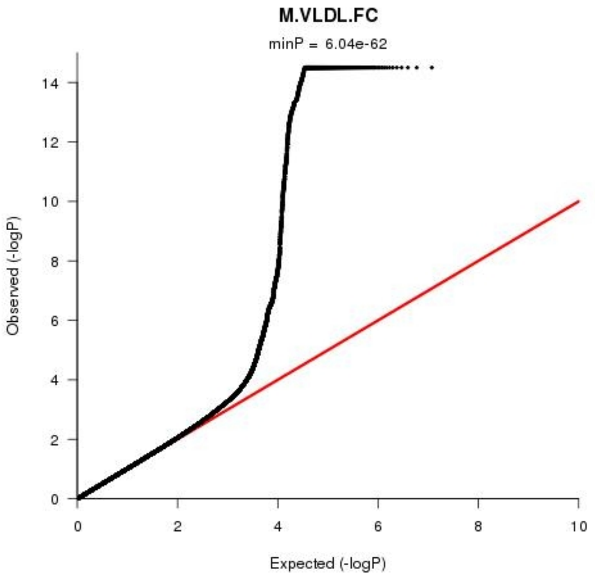
M.VLDL.FC
minP = 6.04e-62
Observed (-logP)
Expected (-logP)
0 2 4 6 8 10 12 14
0 2 4 6 8 10

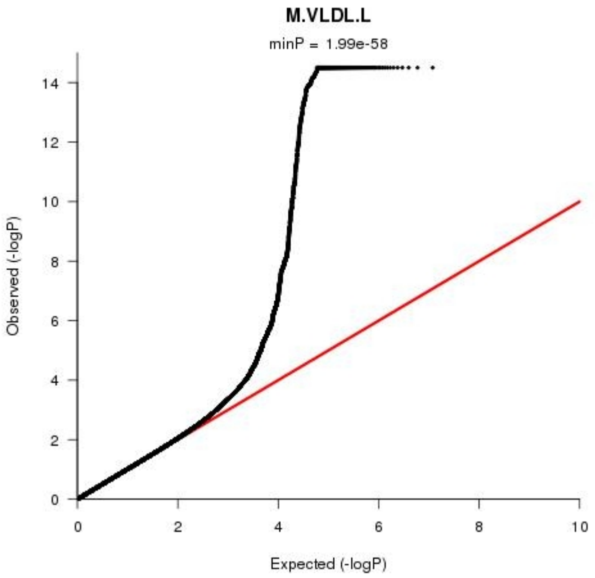
M.VLDL.L
minP = 1.99e-58
Observed (-logP)
Expected (-logP)
0 2 4 6 8 10 12 14
0 2 4 6 8 10

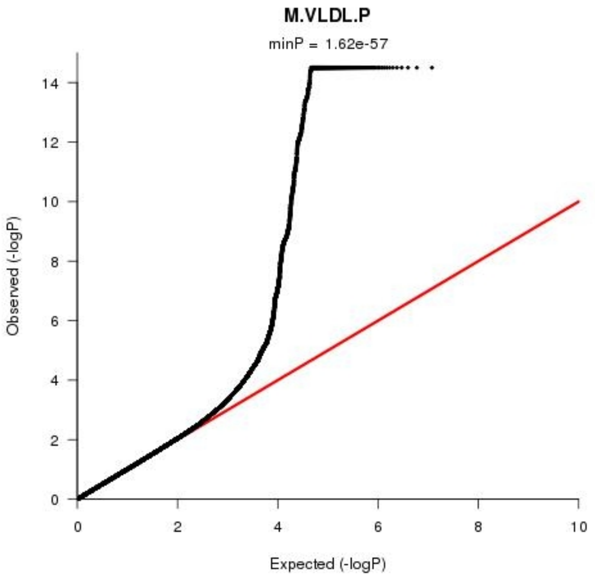
M.VLDL.P
minP = 1.62e-57
Observed (-logP)
Expected (-logP)
0 2 4 6 8 10 12 14
0 2 4 6 8 10

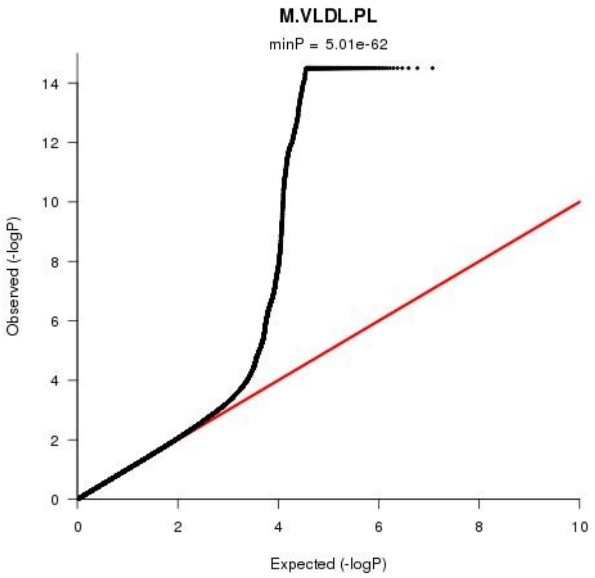
M.VLDL.PL
minP = 5.01e-62
Observed (-logP)
Expected (-logP)
0 2 4 6 8 10 12 14
0 2 4 6 8 10

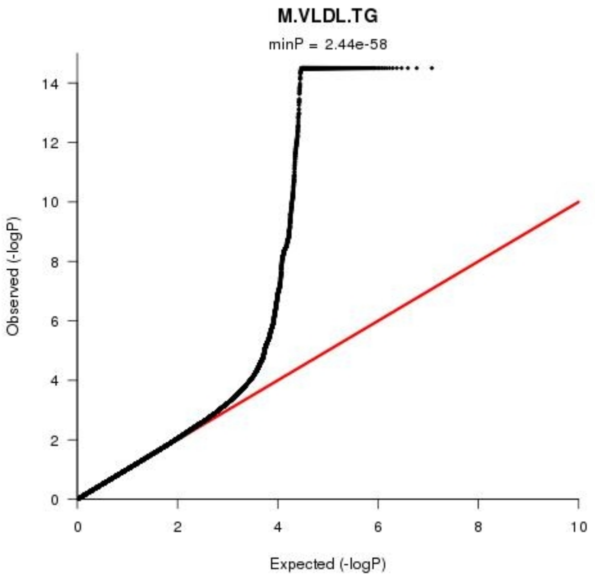
M.VLDL.TG
minP = 2.44e-58
Observed (-logP)
Expected (-logP)
0 2 4 6 8 10 12 14
0 2 4 6 8 10

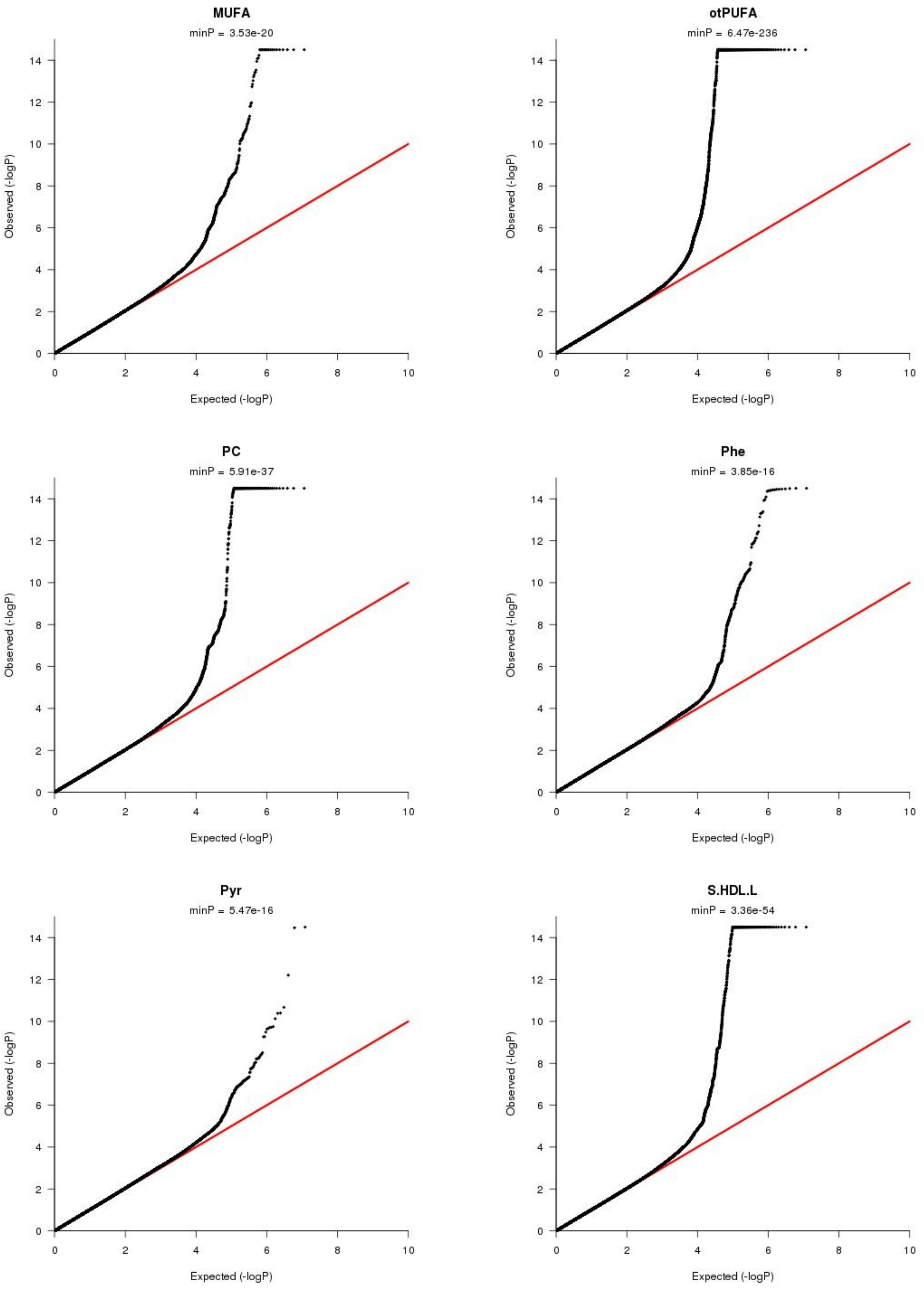
MUFA
minP = 3.53e-20
otPUFA
minP = 6.47e-236
PC
minP = 5.91e-37
Phe
minP = 3.85e-16
Pyr
minP = 5.47e-16
S.HDL.L
minP = 3.36e-54
Observed (-logP)
Expected (-logP)
0
2
4
6
8
10
12
14

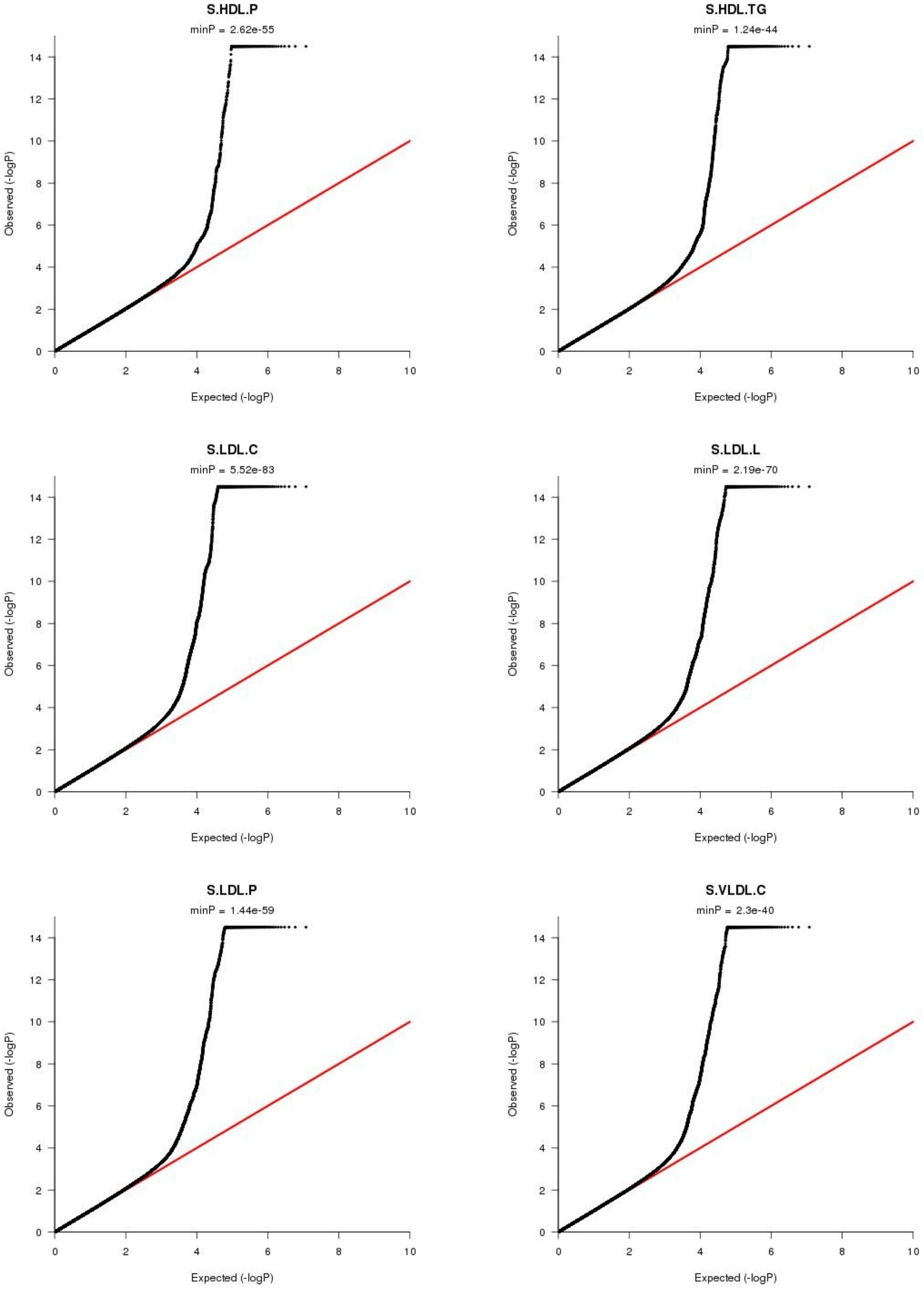
S.HDL.P
minP = 2.62e-55
S.HDL.TG
minP = 1.24e-44
S.LDL.C
minP = 5.52e-83
S.LDL.L
minP = 2.19e-70
S.LDL.P
minP = 1.44e-59
S.VLDL.C
minP = 2.3e-40
Observed (-logP)
Expected (-logP)

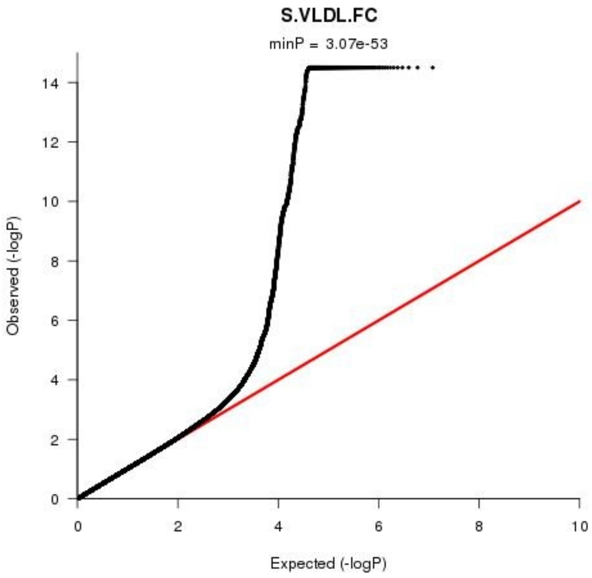
S.VLDL.FC
minP = 3.07e-53
Observed (-logP)
Expected (-logP)

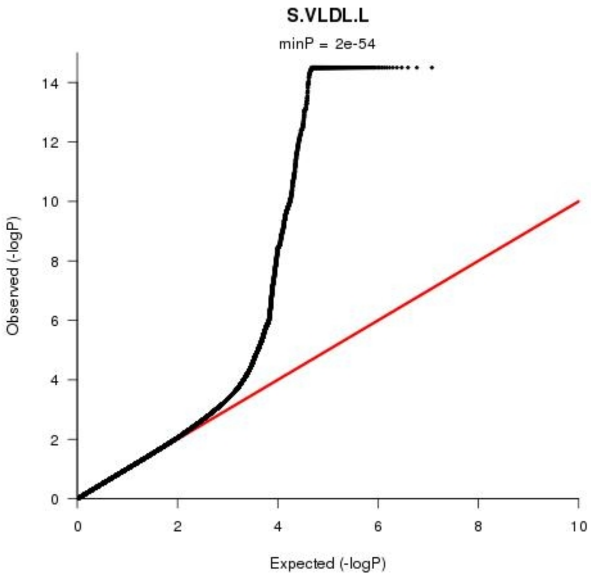
S.VLDL.L
minP = 2e-54
Observed (-logP)
Expected (-logP)

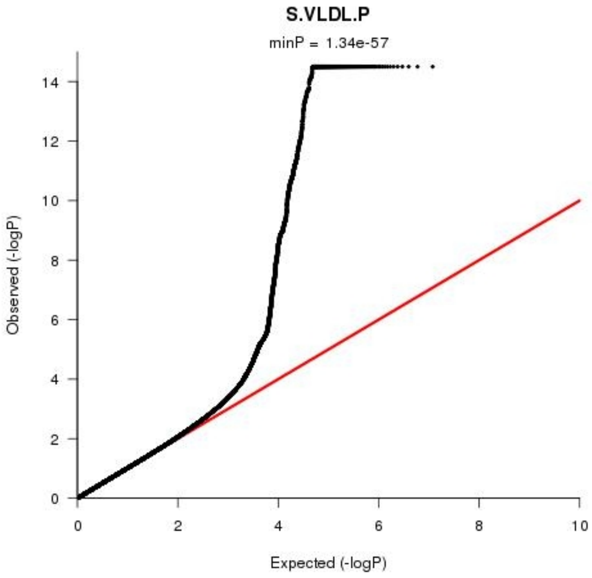
S.VLDL.P
minP = 1.34e-57
Observed (-logP)
Expected (-logP)

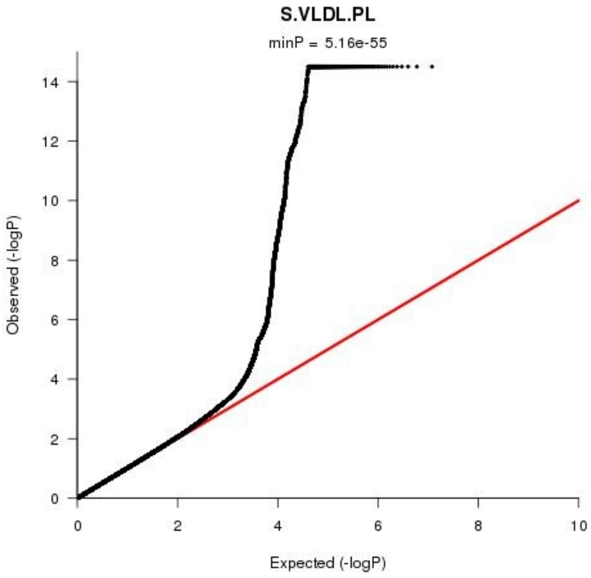
S.VLDL.PL
minP = 5.16e-55
Observed (-logP)
Expected (-logP)

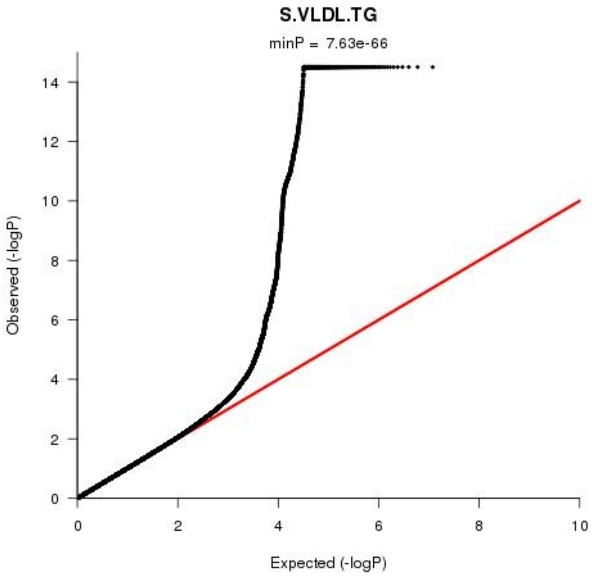
S.VLDL.TG
minP = 7.63e-66
Observed (-logP)
Expected (-logP)

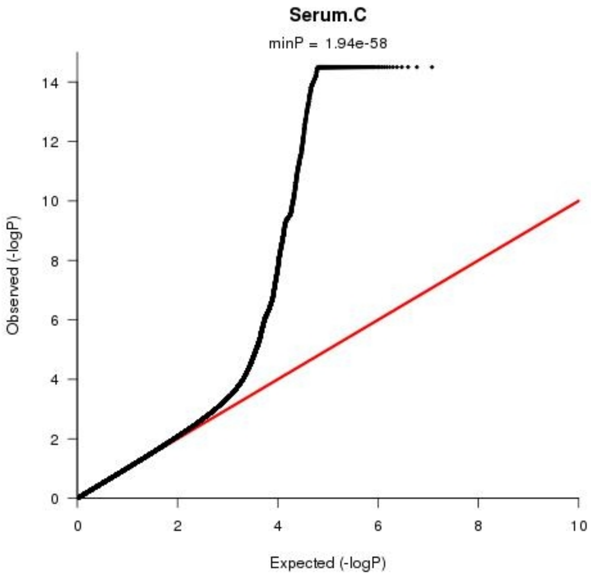
Serum.C
minP = 1.94e-58
Observed (-logP)
Expected (-logP)

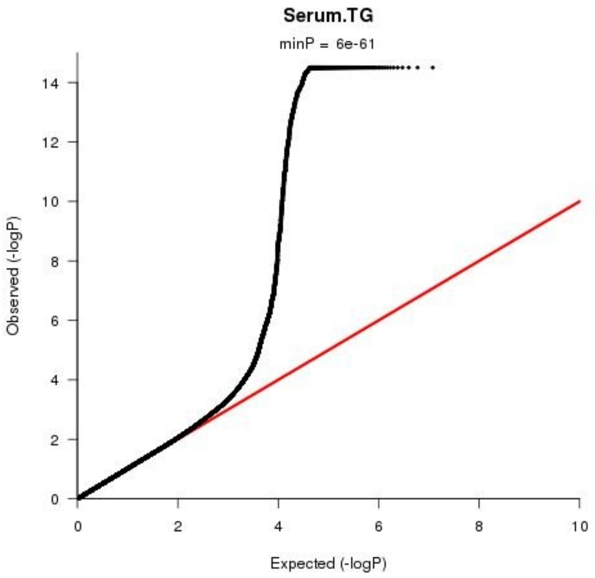
Serum.TG
minP = 6e-61
Observed (-logP)
Expected (-logP)

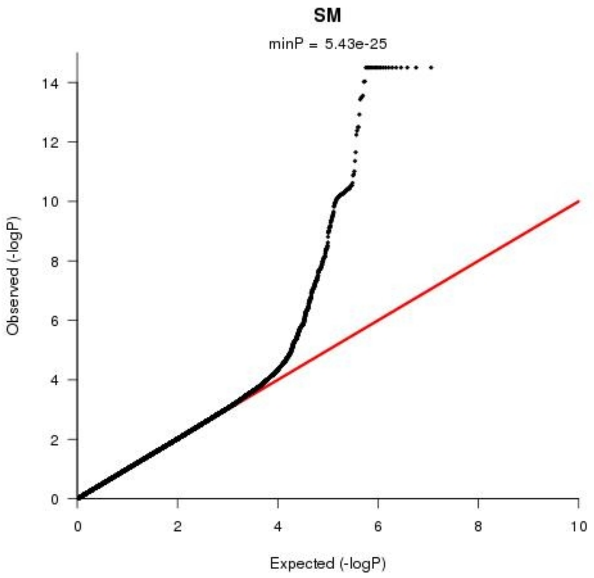
SM
minP = 5.43e-25
Observed (-logP)
Expected (-logP)

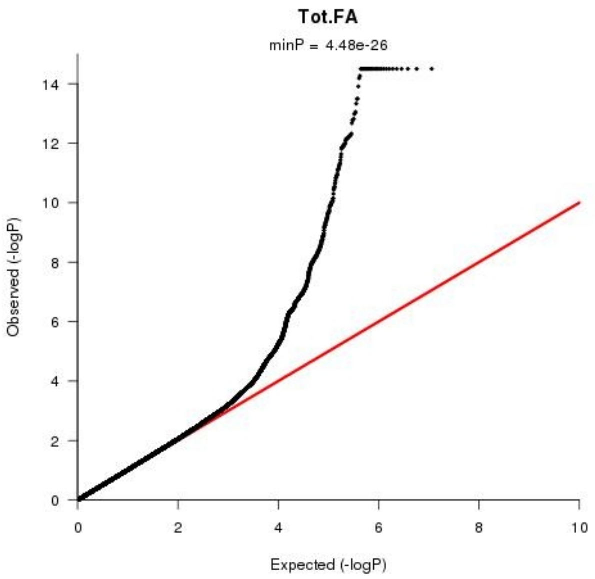
Tot.FA
minP = 4.48e-26
Observed (-logP)
Expected (-logP)

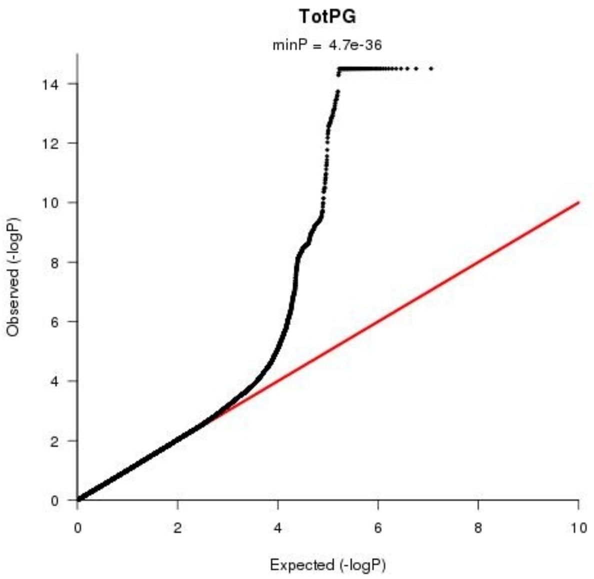
TotPG
minP = 4.7e-36
Observed (-logP)
Expected (-logP)

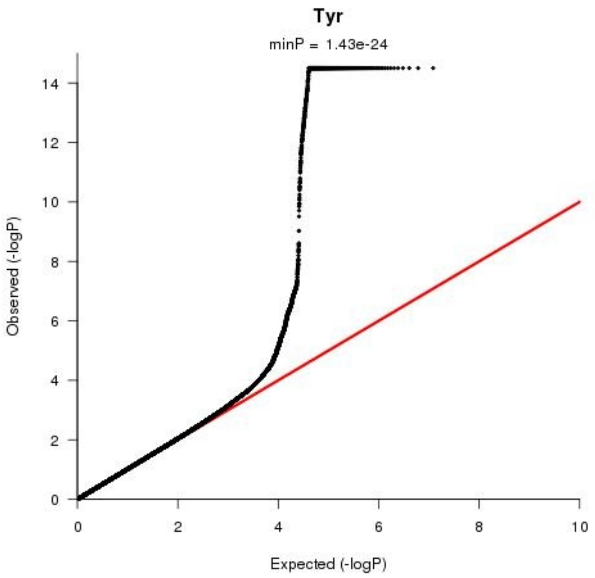
Tyr
minP = 1.43e-24
Observed (-logP)
Expected (-logP)

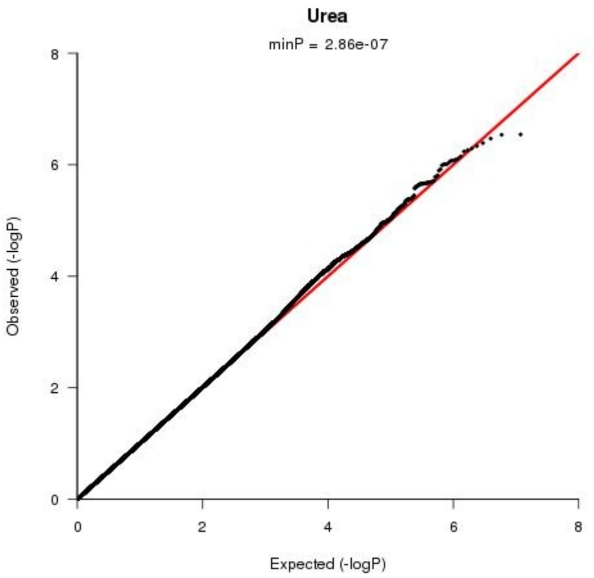
Urea
minP = 2.86e-07
Observed (-logP)
Expected (-logP)

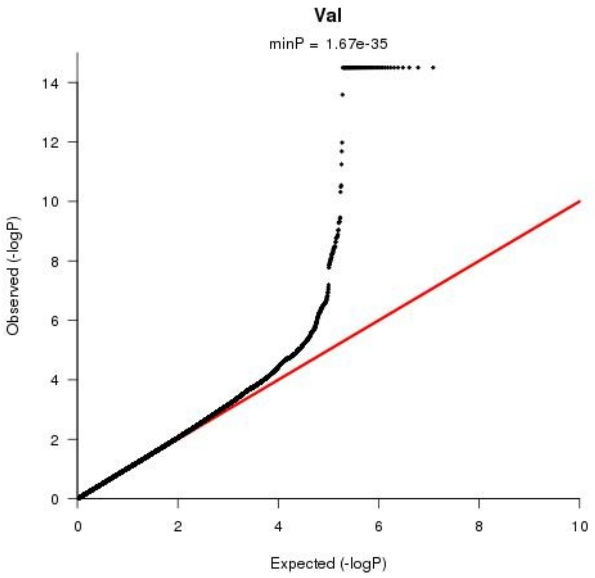
Val
minP = 1.67e-35
Observed (-logP)
Expected (-logP)

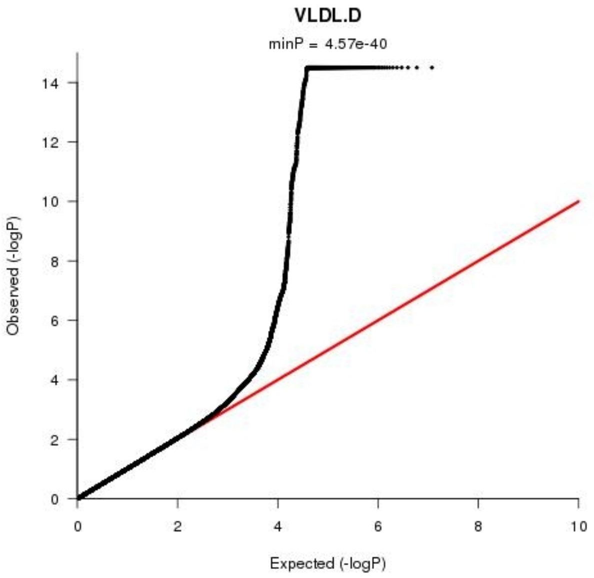
VLDL.D
minP = 4.57e-40
Observed (-logP)
Expected (-logP)

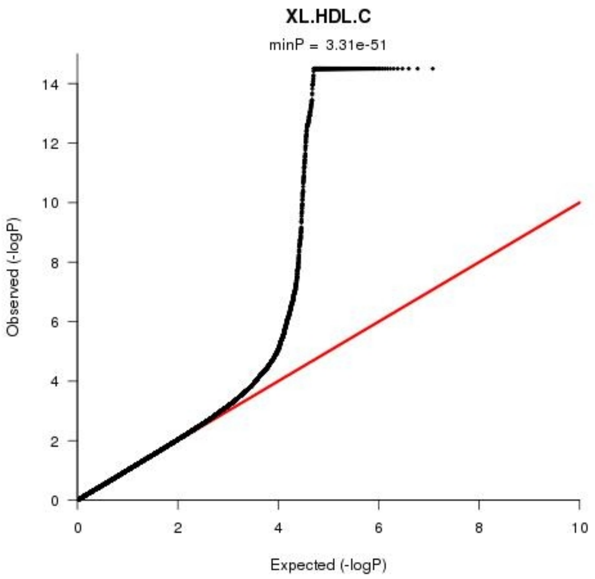
XL.HDL.C
minP = 3.31e-51
Observed (-logP)
Expected (-logP)

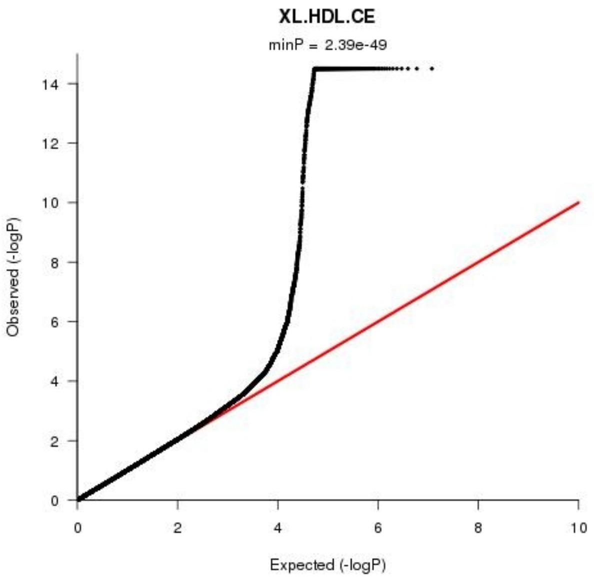
XL.HDL.CE
minP = 2.39e-49
Observed (-logP)
Expected (-logP)

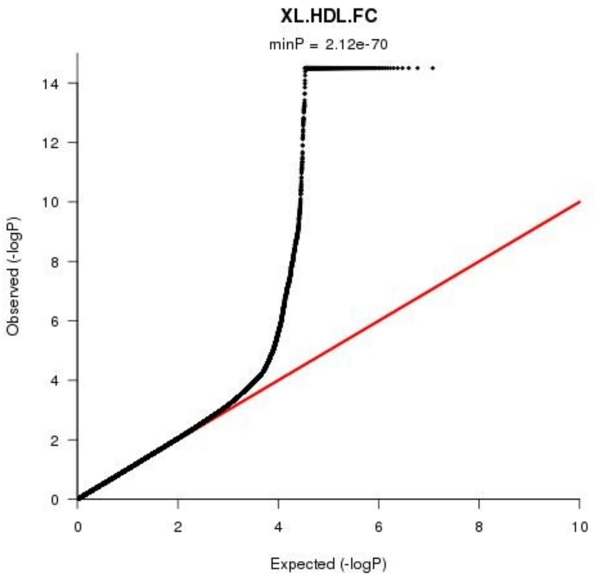
XL.HDL.FC
minP = 2.12e-70
Observed (-logP)
Expected (-logP)

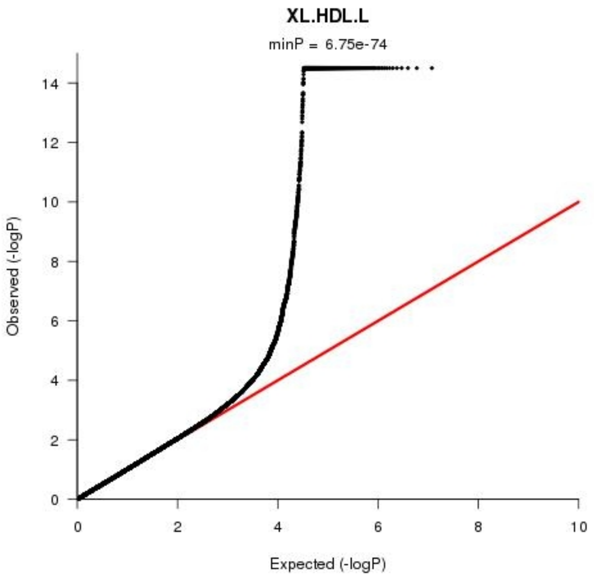
XL.HDL.L
minP = 6.75e-74
Observed (-logP)
Expected (-logP)

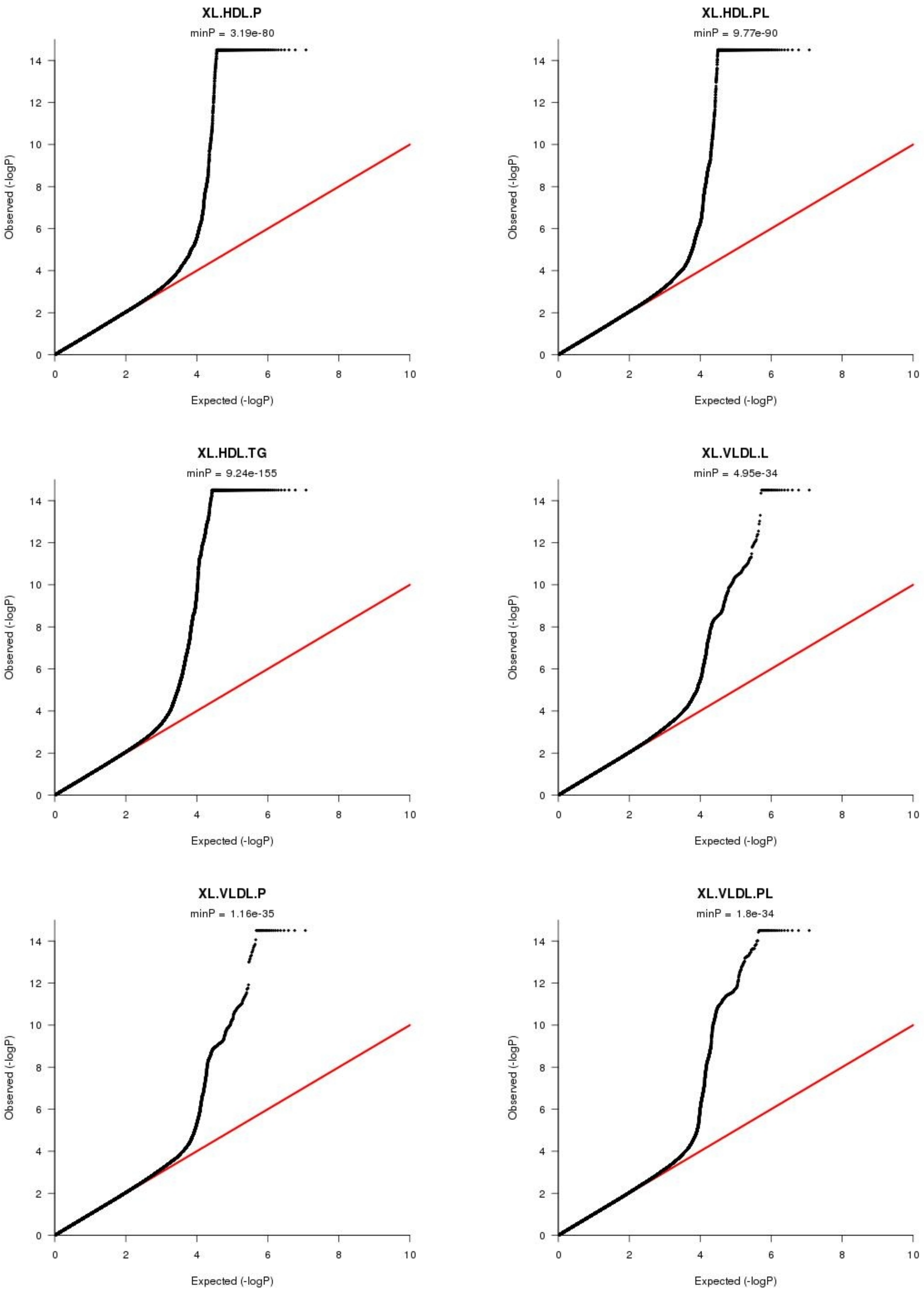
XL.HDL.P
minP = 3.19e-80
Observed (-logP)
Expected (-logP)
XL.HDL.PL
minP = 9.77e-90
Observed (-logP)
Expected (-logP)
XL.HDL.TG
minP = 9.24e-155
Observed (-logP)
Expected (-logP)
XL.VLDL.L
minP = 4.95e-34
Observed (-logP)
Expected (-logP)
XL.VLDL.P
minP = 1.16e-35
Observed (-logP)
Expected (-logP)
XL.VLDL.PL
minP = 1.8e-34
Observed (-logP)
Expected (-logP)

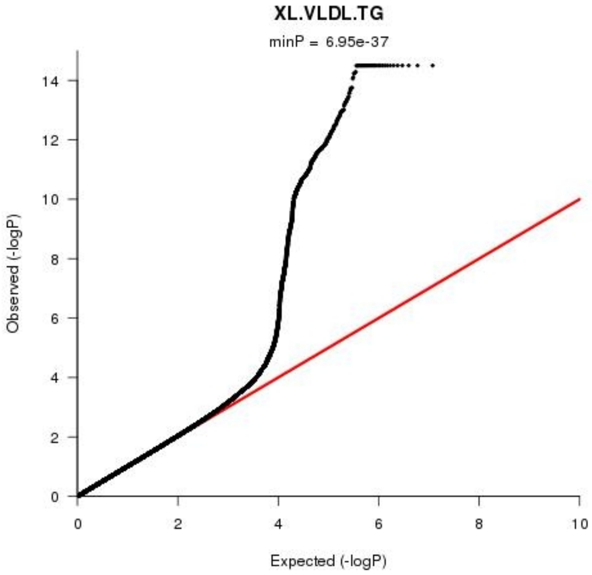
XL.VLDL.TG
minP = 6.95e-37
Observed (-logP)
Expected (-logP)

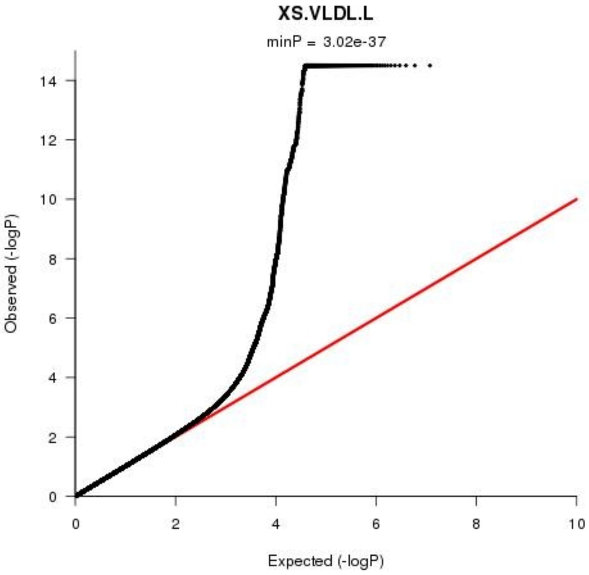
XS.VLDL.L
minP = 3.02e-37
Observed (-logP)
Expected (-logP)

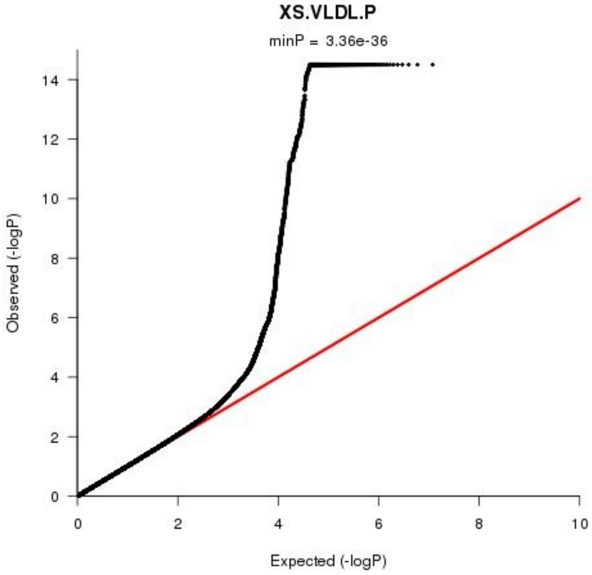
XS.VLDL.P
minP = 3.36e-36
Observed (-logP)
Expected (-logP)

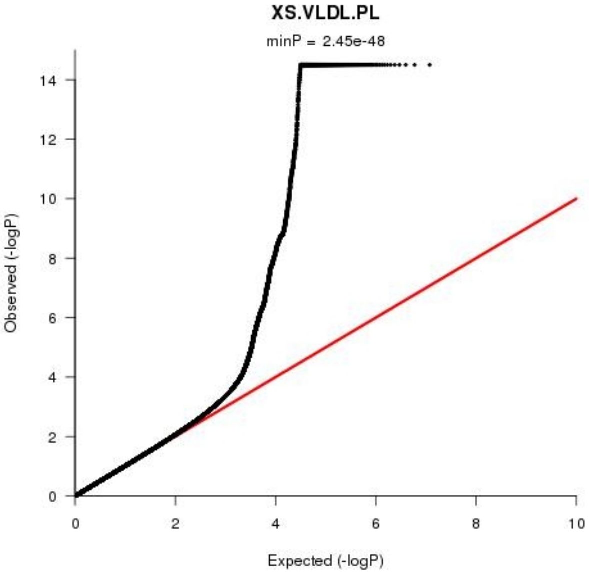
XS.VLDL.PL
minP = 2.45e-48
Observed (-logP)
Expected (-logP)

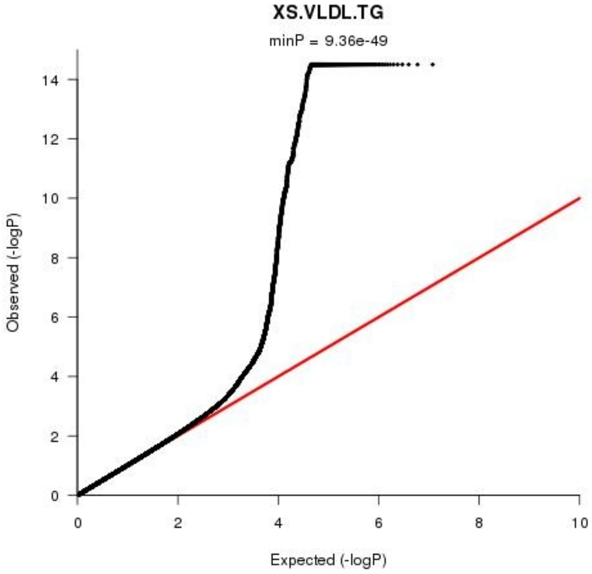
XS.VLDL.TG
minP = 9.36e-49
Observed (-logP)
Expected (-logP)

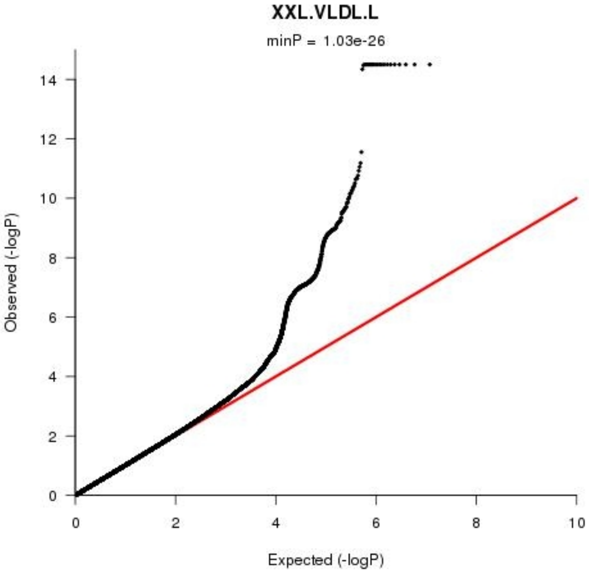
XXL.VLDL.L
minP = 1.03e-26
Observed (-logP)
Expected (-logP)

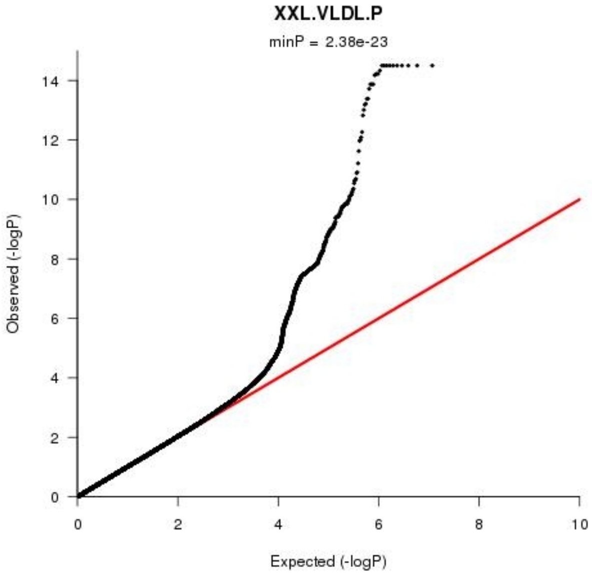
XXL.VLDL.P
minP = 2.38e-23
Observed (-logP)
Expected (-logP)

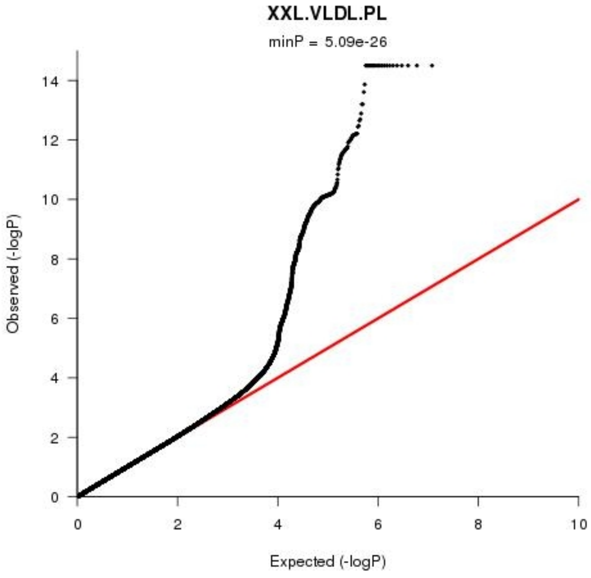
XXL.VLDL.PL
minP = 5.09e-26
Observed (-logP)
Expected (-logP)

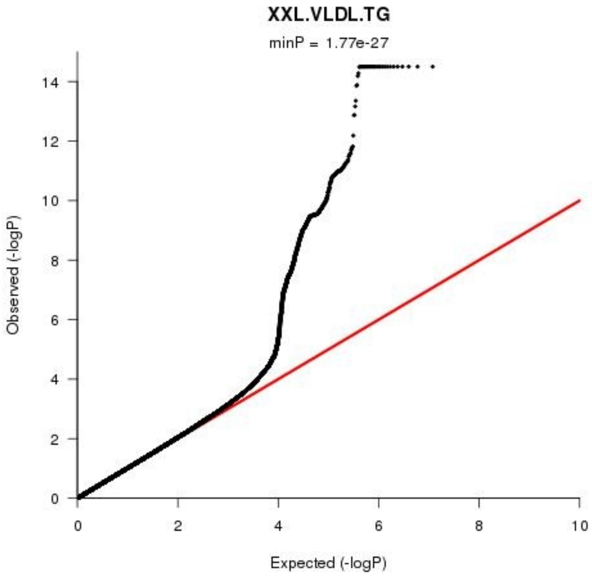
XXL.VLDL.TG
minP = 1.77e-27
Observed (-logP)
Expected (-logP)

Supplementary Table 1. Metabolite abbreviations and names, number of individuals analyzed and proportion of variance explained.

Abbreviation	Name	N	Proportion of variance explained
AcAce	Acetoacetate	19262	0.225 %
Ace	Acetate	24748	NA
Ala	Alanine	24796	1.326 %
Alb	Albumin	18960	0.984 %
ApoA1	ApoA1	20687	5.005 %
ApoB	ApoB	20690	8.609 %
Bis.DB.ratio	Ratio of bis-allylic bonds to double bonds in lipids	13524	7.392 %
Bis.FA.ratio	Ratio of bis-allylic bonds to total fatty acids in lipids	13171	6.324 %
bOHBut	3-hydroxybutyrate	24154	NA
CH2.in.FA	CH2 groups in fatty acids	19021	2.697 %
CH2.DB.ratio	CH2 groups to double bonds ratio	13532	5.122 %
Cit	Citrate	24770	1.197 %
Crea	Creatinine	24810	0.930 %
DB.in.FA	Double bonds in fatty acids	15728	3.066 %
DHA	22:6, docosahexaenoic acid (DHA)	13499	1.677 %
Est.C	Esterified cholesterol	13497	7.582 %
FALen	Fatty acid length	13476	NA
FAw3	Omega-3 fatty acids	13544	2.873 %
FAw6	Omega-6 fatty acids	13506	5.036 %
FAw79S	Omega-7 and -9 and saturated fatty acids	13506	2.500 %
Free.C	Free cholesterol	13497	8.347 %
Glc	Glucose	24679	1.182 %
Gln	Glutamine	24462	1.772 %
Glol	Glycerol	20235	NA
Gly	Glycine	18734	12.488 %
Gp	Glycoprotein acetyls, mainly a1-acid glycoprotein	19270	2.410 %
HDL.C	Total cholesterol in HDL	21555	6.115 %
HDL.D	HDL diameter	19273	10.323 %
His	Histidine	19244	1.108 %
IDL.C	Total cholesterol in IDL	19273	10.892 %
IDL.FC	Free cholesterol in IDL	21559	10.894 %
IDL.L	Total lipids in IDL	19273	11.267 %
IDL.P	Concentration of IDL particles	19273	11.173 %
IDL.PL	Phospholipids in IDL	21559	11.205 %
IDL.TG	Triglycerides in IDL	19273	9.943 %
Ile	Isoleucine	24776	0.557 %
L.HDL.C	Total cholesterol in large HDL	21558	8.227 %
L.HDL.CE	Cholesterol esters in large HDL	19273	8.386 %
L.HDL.FC	Free cholesterol in large HDL	21559	7.686 %
L.HDL.L	Total lipids in large HDL	19273	8.425 %
L.HDL.P	Concentration of large HDL particles	19273	8.436 %
L.HDL.PL	Phospholipids in large HDL	19273	8.030 %
L.LDL.C	Total cholesterol in large LDL	21552	10.442 %
L.LDL.CE	Cholesterol esters in large LDL	19273	10.559 %
L.LDL.FC	Free cholesterol in large LDL	21555	11.078 %
L.LDL.L	Total lipids in large LDL	19273	10.715 %
L.LDL.P	Concentration of large LDL particles	19273	11.131 %
L.LDL.PL	Phospholipids in large LDL	21550	10.506 %
L.VLDL.C	Total cholesterol in large VLDL	21235	3.239 %
L.VLDL.CE	Cholesterol esters in large VLDL	18960	3.391 %
L.VLDL.FC	Free cholesterol in large VLDL	21238	2.907 %
L.VLDL.L	Total lipids in large VLDL	18960	2.441 %
L.VLDL.P	Concentration of large VLDL particles	18960	2.841 %
L.VLDL.PL	Phospholipids in large VLDL	21239	2.393 %
L.VLDL.TG	Triglycerides in large VLDL	21239	2.586 %
LA	18:2, linoleic acid (LA)	13527	6.584 %
Lac	Lactate	24871	NA
LDL.C	Total cholesterol in LDL	21559	10.720 %
LDL.D	LDL diameter	19273	3.124 %
Leu	Leucine	24728	0.797 %
M.HDL.C	Total cholesterol in medium HDL	21558	2.378 %
M.HDL.CE	Cholesterol esters in medium HDL	19273	2.066 %
M.HDL.FC	Free cholesterol in medium HDL	21559	2.380 %
M.HDL.L	Total lipids in medium HDL	19273	1.764 %
M.HDL.P	Concentration of medium HDL particles	19273	1.537 %

M.HDL.PL	Phospholipids in medium HDL	21558	1.901 %
M.LDL.C	Total cholesterol in medium LDL	21559	9.903 %
M.LDL.CE	Cholesterol esters in medium LDL	19273	10.090 %
M.LDL.L	Total lipids in medium LDL	19273	9.933 %
M.LDL.P	Concentration of medium LDL particles	19273	9.708 %
M.LDL.PL	Phospholipids in medium LDL	21558	8.684 %
M.VLDL.C	Total cholesterol in medium VLDL	21551	5.119 %
M.VLDL.CE	Cholesterol esters in medium VLDL	19273	5.926 %
M.VLDL.FC	Free cholesterol in medium VLDL	21240	4.285 %
M.VLDL.L	Total lipids in medium VLDL	19273	3.649 %
M.VLDL.P	Concentration of medium VLDL particles	19273	3.387 %
M.VLDL.PL	Phospholipids in medium VLDL	21240	4.283 %
M.VLDL.TG	Triglycerides in medium VLDL	21241	3.190 %
MUFA	Mono-unsaturated fatty acids	13535	2.067 %
otPUFA	Other polyunsaturated fatty acids than 18:2	13549	10.162 %
PC	Phosphatidylcholine and other cholines	13542	4.258 %
Phe	Phenylalanine	22663	1.105 %
Pyr	Pyruvate	24756	0.523 %
S.HDL.L	Total lipids in small HDL	19273	3.063 %
S.HDL.P	Concentration of small HDL particles	19273	2.494 %
S.HDL.TG	Triglycerides in small HDL	21558	4.242 %
S.LDL.C	Total cholesterol in small LDL	21556	8.964 %
S.LDL.L	Total lipids in small LDL	19273	8.689 %
S.LDL.P	Concentration of small LDL particles	19273	8.504 %
S.VLDL.C	Total cholesterol in small VLDL	21557	6.716 %
S.VLDL.FC	Free cholesterol in small VLDL	21559	6.426 %
S.VLDL.L	Total lipids in small VLDL	19273	6.258 %
S.VLDL.P	Concentration of small VLDL particles	19273	5.495 %
S.VLDL.PL	Phospholipids in small VLDL	21551	6.322 %
S.VLDL.TG	Triglycerides in small VLDL	21558	4.910 %
Serum.C	Serum total cholesterol	21491	8.671 %
Serum.TG	Serum total triglycerides	21545	4.331 %
SM	Sphingomyelins	13476	3.918 %
Tot.FA	Total fatty acids	13505	3.346 %
TotPG	Total phosphoglycerides	13519	4.242 %
Tyr	Tyrosine	24925	0.444 %
Urea	Urea	18814	NA
Val	Valine	24900	1.008 %
VLDL.D	VLDL diameter	19273	4.652 %
XL.HDL.C	Total cholesterol in very large HDL	21540	5.051 %
XL.HDL.CE	Cholesterol esters in very large HDL	19273	5.174 %
XL.HDL.FC	Free cholesterol in very large HDL	21542	6.875 %
XL.HDL.L	Total lipids in very large HDL	19273	7.718 %
XL.HDL.P	Concentration of very large HDL particles	19273	7.258 %
XL.HDL.PL	Phospholipids in very large HDL	19273	9.376 %
XL.HDL.TG	Triglycerides in very large HDL	21536	10.760 %
XL.VLDL.L	Total lipids in very large VLDL	19273	2.047 %
XL.VLDL.P	Concentration of very large VLDL particles	18960	2.009 %
XL.VLDL.PL	Phospholipids in very large VLDL	21237	2.109 %
XL.VLDL.TG	Triglycerides in very large VLDL	21548	2.409 %
XS.VLDL.L	Total lipids in very small VLDL	19273	9.862 %
XS.VLDL.P	Concentration of very small VLDL particles	19273	9.447 %
XS.VLDL.PL	Phospholipids in very small VLDL	19273	10.552 %
XS.VLDL.TG	Triglycerides in very small VLDL	19273	7.125 %
XXL.VLDL.L	Total lipids in chylomicrons and extremely large VLDL	18960	1.567 %
XXL.VLDL.P	Concentration of chylomicrons and extremely large VLDL particles	18960	1.401 %
XXL.VLDL.PL	Phospholipids in chylomicrons and extremely large VLDL	21542	1.572 %
XXL.VLDL.TG	Triglycerides in chylomicrons and extremely large VLDL	21540	1.710 %

NA Those metabolic measures where no genome-wide associations were found

Supplementary Table 2. Genotyping details, genotype quality control and metabolomics measurement details.

Study	Short name	COROGENE	EGCUT	ERF	FR97	GenMets	HBCS	KORA-F4	LLS	NFBC 1966	NTR	Predict CVD	Prote	Twins	YFS
	**Full name**	Genetic Predisposition of Coronary Heart Disease in Patients Verified with Coronary Angiogram	Estonian Genome Center, University of Tartu	Erasmus Rucphen Family Study	FINRISK 1997	Genetics of metabolic syndrome	Helsinki birth cohort study	kooperative gesundheitsforschung in der region augsburg	Leiden Longevity Study	Norther Finland Birth Cohort Study 1966	Netherlands Twin Register	Predict CVD	EGCUT 2nd cohort	Finnish Twin Cohort	The Cardiovascular Risk in Young Finns Study
**Study design**		Case-control study, only controls used	Population-based cohort	Family-based	Population-based cohort	Case-control study	Birth cohort	Population based cohort	Family-based	Birth cohort	Population based twin study	Cohort study	Population-based	Population based twin study	Follow up study in children
**Ethnicity**		Finnish	Estonian	Dutch	Finnish	Finnish	Finnish	German	Dutch	Finnish	Dutch	Finnish	Estonian	Finnish	Finnish
**Total genotyped sample size (N)**		4122	7734	2917	5288	2118	1676	1996	2415	5104	14003	1881	1397	664	2443
**Sample QC**	**Call rate***	≥ 95%	≥95%	≥ 95%	≥ 95%	≥ 95%	≥ 95%	≥ 95%	≥ 95%	≥ 95%	SNP missing rate < 10% in subsets	≥ 95%	≥95%	≥ 95%	≥ 95%
	**other exclusions**	IBD > 0.1, sex mismatches, duplicates, heterozygosity outliers by eye from distribution	Additional exclusions: IBD > 0.1, sex mismatches, duplicates	Mendelian inconsistencies	IBD > 0.1, sex mismatches, duplicates, heterozygosity outliers by eye from distribution	IBD > 0.1, sex mismatches, duplicates, heterozygosity outliers by eye from distribution	IBD > 0.1, sex mismatches, duplicates, heterozygosity outliers by eye from distribution	IBD > 0.1, sex mismatches, duplicates, heterozygosity outliers by eye from distribution	Relatedness, Mendelian inconsistencies	IBD > 0.1, sex mismatches, duplicates, heterozygosity outliers by eye from distribution	Heterozygosity F < -0.10 or > 0.10; mismatch between X-chromosomal genotype and phenotypic sex; failure of expected IBS / IBD relations between samples; Mendel errors > 2%; different DNA for the same sample or between confirmed MZ co-twins over merged SNP genotype-imputed subsets	IBD > 0.1, sex mismatches, duplicates, heterozygosity outliers by eye from distribution	Additional exclusions: IBD > 0.1, sex mismatches, duplicates	IBD > 0.1, sex mismatches, duplicates, heterozygosity outliers by eye from distribution	IBD > 0.1, sex mismatches, duplicates, heterozygosity outliers by eye from distribution
**Assesment NMR**	**Machine**	500 MHz Bruker AVANCE III spectrometer	500 MHz Bruker AVANCE III spectrometer	600 MHz Bruker AVANCE II spectrometer	500 MHz Bruker AVANCE III spectrometer	500 MHz Bruker AVANCE III spectrometer	500 MHz Bruker AVANCE III spectrometer	500 MHz Bruker AVANCE III spectrometer	600 MHz Bruker AVANCE II spectrometer	500 MHz Bruker AVANCE III spectrometer	600 MHz Bruker AVANCE II spectrometer	500 MHz Bruker AVANCE III spectrometer	500 MHz Bruker AVANCE III spectrometer	500 MHz Bruker AVANCE III spectrometer	500 MHz Bruker AVANCE III spectrometer
	**Sample material**	Serum	Plasma	Serum	Serum	Serum	Serum	Serum	Plasma	Serum	Plasma	Serum	Plasma	Serum	Serum
	**Processing**	In-house algorithm	In-house algorithm	in-house deconvolution algorithm	In-house algorithm	In-house algorithm	In-house algorithm	In-house algorithm	in-house deconvolution algorithm	In-house algorithm	in-house deconvolution algorithm	In-house algorithm	In-house algorithm	In-house algorithm	In-house algorithm
**Genotyping**		illumina 610k	Illumina HumanOmniExpress, Illumina HumanCNV370	Illumina 318K, Illumina 370K and Affymetrix 250K	Illumina core-exome	Illumina 610k	Illumina 670k	Affemetrix 6.0	Illumina Human660W, Illumina OmniExpress	Illumina 370k	Perlegen-Affymetrix 500K; Illumina 660K; Illumina 370K; Affymetrix 6.0 907K; Illumina Omni 1M	Illumina omni express	Illumina HumanOmniExpress, Illumina HumanCNV370	Illumina 670k	Illumina 670k
	**Genotype calling algorithm**	Illuminus	Genome Studio	Illumina Bead Studio, BRLMM	Illuminus	Illuminus	Illuminus	Birdseed2	GenomeStudio	Illuminus	Genotyper, Beadstudio, Birdseed	Illuminus	Genome Studio	Illuminus	Illuminus
	**MAF**	≥ 1%	≥ 1%	≥ 1%	≥ 1%	≥ 1%	≥ 1%	≥ 1%	≥ 1%	≥ 1%	> 1%	≥ 1%	≥ 1%	≥ 1%	≥ 1%
	**Call rate***	≥ 95%	≥ 95%	≥ 98%	≥ 95%	≥ 95%	≥ 95%	≥ 95%	≥ 95%	≥ 95%	> 0.95	≥ 95%	≥ 95%	≥ 95%	≥ 95%
	**p for HWE**	> 10-6	> 10-6	> 10-6	> 10-6	> 10-6	> 10-6	> 10-7	> 10-4	> 10-6	> 1E-05	> 10-6	> 10-6	> 10-6	> 10-6
	**SNPs that met QC criteria**	554988	38.02 M in final list	659174	273113	555388	546814	692637	522801-645804	335118	289598-1139672	645088	38.02 M in final list	552894	546677
	**Imputation software**	IMPUTE2	IMPUTE2	Mach/Minimac	IMPUTE2	IMPUTE2	IMPUTE2	IMPUTE	IMPUTE version 2.2 (beta)	IMPUTE2	Mach phasing, Minimac Imputation	IMPUTE2	IMPUTE2	IMPUTE2	IMPUTE2
**Association analyses**	**Analysis software**	SNPTest 2.4.1	SNPTest 2.4.1	ProbABEL	SNPTest 2.4.1	SNPTest 2.4.1	SNPTest 2.4.1	SNPTEST	QT-assoc	SNPTest 2.4.1	PLINK 1.07	SNPTest 2.4.1	SNPTest 2.4.1	GEMMA	SNPTest 2.4.1
	**Cohort specific adjustments**			Family structure					Familial relations		Relatedness; genotyping sample			Family structure	

Supplementary Table 3. Conditional variants

Round 1: conditioned with the lead variant in the locus given in Tables 2 and 3

Phenotype	rsid	Chromosome	Position	Beta	SE	p-value
L.LDL.FC	rs11591147	1	55505647	-0.5335034	0.03230012	2.76E-61
L.LDL.FC	rs13011615	2	21274167	-0.1247424	0.01401459	5.54E-19
Val	rs34226052	4	89163523	-0.0967459	0.01401841	5.15E-12
HDL.C	rs75835816	8	19885513	-0.279236	0.0368885	3.74E-14
His	rs2072510	12	96403199	-0.0689075	0.01048573	4.98E-11
XL.HDL.TG	rs261334	15	58726744	-0.2686627	0.0112669	1.13E-125
HDL.C	rs11076175	16	57006378	-0.1511545	0.0135707	8.17E-29
otPUFA	rs190121281	19	19252779	-0.3228719	0.03264413	4.57E-23
S.HDL.L	rs6032614	20	44609595	-0.1017956	0.01388914	2.32E-13

Round 2: conditioned with the lead variant in the locus given in Tables 2 and 3 as well as round 1 variant above

Phenotype	rsid	Chromosome	Position	Beta	SE	p-value
XS.VLDL.TG	chr2.211488[illegible]	2	21148888	-0.1263201	0.01389021	9.53E-20
XL.HDL.TG	rs56050415	15	58574966	-0.1198553	0.01366415	1.76E-18

Round 3: conditioned with the lead variant in the locus given in Tables 2 and 3 as well as round 1 and 2 variants above

Phenotype	rsid	Chromosome	Position	Beta	SE	p-value
HDL.D	chr15.58927[illegible]	15	58927492	-0.1706685	0.02565108	2.86E-11

Supplementary Table 4. Previously identified loci and SNPs significant in this GWAS. Beta refers to one copy addition of the effect allele in standard deviation units. Metabolic traits are grouped coarsely by phenotypic similarity.

Trait	Variant identifi[illegible]	Chr	Position	ea/nea	Eaf	Beta	SE	P-value	Q P-value	N samples	Candidate gene	eQTL	Function
**Lipoprotein measuress**													
L.LDL.FC	rs191448950	1	55584844	A/G	0.02	-0.5	0.03	4.8×10^{-56}	0.31	21549	*PCSK9*	—	Intron;USP24
L.LDL.FC	chr1:10981815[illegible]	1	109818158	CT/C	0.78	0.14	0.01	1.0×10^{-27}	0.15	19265	*SORT1*	*CELSR2*	5'UTR/TBFS;CELSR2
XL.HDL.PL	rs4503368	1	161190250	C/T	0.53	-0.07	0.01	3.2×10^{-11}	0.34	19270	*USF1*	—	—
M.HDL.FC	rs590820	1	230309619	G/A	0.44	-0.06	0.01	$1.6\times10^{[illegible]}$	0.2	21553	*GALNT2*	—	Intron
IDL.FC	rs952275	2	21221399	G/T	0.43	0.11	0.01	1.5×10^{-27}	0.18	21556	*APOB*	—	TFBS
LDL.C	rs6756629#	2	44065090	A/G	0.08	-0.14	0.02	1.2×10^{-14}	0.15	21559	*ABCG5*	—	Missense
S.LDL.C	rs4703667	5	74613906	C/G	0.39	0.09	0.01	2.6×10^{-18}	0.76	21551	*HMGCR*	—	—
VLDL.D	rs10455872	6	161010118	G/A	0.04	-0.2	0.03	1.3×10^{-12}	0.03	19272	*LPA*	—	Intron
L.LDL.PL	rs73066442	7	21592973	G/A	0.25	0.08	0.01	9.7×10^{-11}	0.71	21546	*DNAH11*	*DNAH11*	Intron
VLDL.D	rs17145750	7	73026378	T/C	0.15	-0.13	0.01	4.3×10^{-20}	0.5	19268	*MLX1PL*	*MLX1PL*	Intron
S.VLDL.TG	rs115849089	8	19912370	A/G	0.11	-0.18	0.02	1.6×10^{-25}	0.4	21554	*LPL*	—	—
XS.VLDL.TG	rs2954029	8	126490972	T/A	0.48	-0.09	0.01	2.8×10^{-16}	0.051	19270	*TRIB1*	—	—
XL.HDL.FC	rs686030	9	15304782	A/C	0.87	0.09	0.01	$2.0\times10^{[illegible]}$	0.91	21541	*TTC39B*	—	TFBS
XL.HDL.C	rs2575876	9	107665739	A/G	0.19	-0.1	0.01	2.7×10^{-15}	0.16	21540	*ABCA1*	—	TFBS/Intron
IDL.L	chr9:13614970[illegible]	9	136149709	A/AC	0.19	0.09	0.01	1.0×10^{-11}	0.068	19269	*ABO*	—	Intron
S.VLDL.TG	rs964184	11	116648917	C/G	0.86	-0.24	0.01	7.6×10^{-66}	$4.0\times10^{[illegible]}$	21556	*ZNF259*	—	TFBS
HDL.D	rs67053123	12	125353810	A/T	0.15	0.11	0.01	9.8×10^{-13}	0.67	19270	*SCARB1*	—	TFBS
XL.HDL.TG	rs1532085	15	58683366	G/A	0.6	-0.26	0.01	$9.2\times10^{-15[illegible]}$	0.59	21536	*LIPC*	—	—
HDL.C	rs247617	16	56990716	A/C	0.3	0.23	0.01	5.8×10^{-97}	0.26	21550	*CETP*	—	TFBS
HDL.D	rs6507939	18	47176261	C/A	0.84	0.11	0.01	8.3×10^{-16}	0.46	19268	*LIPG*	—	—
VLDL.D	rs116843064#	19	8429323	A/G	0.03	-0.22	0.03	2.8×10^{-10}	0.016	19268	*ANGPTL4*	—	Missense
L.LDL.FC	rs142130958	19	11190652	A/G	0.11	-0.24	0.02	6.5×10^{-47}	0.11	21554	*LDLR*	—	—
M.VLDL.C	rs72999033	19	19366632	T/C	0.06	-0.18	0.02	1.0×10^{-16}	0.6	21548	*CILP*	—	TFBS/UTR;HAPLN4
L.LDL.FC	rs7412	19	45412079	T/C	0.06	-0.59	0.03	$2.0\times10^{-12[illegible]}$	0.15	18263	*APOE*	—	Missense
XS.VLDL.PL	rs1883711	20	39179822	C/G	0.06	0.17	0.02	2.3×10^{-11}	0.16	19268	*MAFB*	—	TFBS
S.HDL.P	rs6073958	20	44551855	C/T	0.2	0.2	0.01	2.6×10^{-55}	0.35	19269	*PLTP*	—	—
**Lipid measures**													
Serum.TG	rs1168041	1	62960250	C/T	0.72	0.1	0.01	3.4×10^{-17}	0.62	21539	*ANGPTL3*	—	Intron;DOCK7
otPUFA	rs174547	11	61570783	C/T	0.4	-0.4	0.01	$6.5\times10^{-23[illegible]}$	$3.8\times10^{[illegible]}$	13547	*FADS1*	*FADS2*	Intron
Bis.DB.ratio	rs11644601	16	15172118	C/T	0.28	0.1	0.01	7.7×10^{-13}	0.16	13517	*PDXC1*	*NPIPA5*	Intron
Sphingomyelin	rs181807530	17	4774814	G/C	0.04	-0.37	0.04	5.4×10^{-25}	0.25	11439	*MINK1*	—	Intron
**Small molecules and protein measures**													
Alanine	rs1260326#	2	27730940	C/T	0.64	-0.1	0.01	7.4×10^{-26}	0.19	22569	*GCKR*	—	Missense
Valine	rs10211524	2	65208074	A/G	0.41	0.09	0.01	5.2×10^{-20}	0.1	24898	*SLC1A4*	—	—
Glucose	rs560887	2	169763148	C/T	0.7	0.12	0.01	3.4×10^{-32}	0.0016	22452	*G6PC2*	—	Intron
Glycine	rs1047891#	2	211540507	A/C	0.33	0.49	0.01	$<1\times10^{-30[illegible]}$	$5.3\times10^{[illegible]}$	18730	*CPS1*	—	Missense
Albumin	rs184650103	4	74850649	T/C	0.01	-0.6	0.05	1.2×10^{-36}	0.23	17461	*ALB*	—	TFBS
Creatinine	rs1986734	4	77420787	T/C	0.56	0.06	0.01	3.0×10^{-10}	0.62	21918	*SHROOM3*	—	TFBS
Valine	rs9637599	4	89206230	C/A	0.47	0.11	0.01	1.7×10^{-35}	0.12	24899	*PPM1K*	*lincRNA*	TFBS
Histidine	rs3733402#	4	187158034	A/G	0.57	0.08	0.01	9.1×10^{-15}	0.013	19241	*KLKB1*	—	Missense
Citrate	rs2921604	5	14867948	C/T	0.44	0.1	0.01	3.7×10^{-25}	0.43	24765	*ANKH*	*ANHK*	Intron
Phenylalanine	rs2731672	5	176842474	C/T	0.74	0.09	0.01	3.9×10^{-16}	$4.0\times10^{[illegible]}$	20436	*F12*	*LMAN2*	—
Tyrosine	rs14399	6	111543944	A/C	0.39	-0.1	0.01	1.4×10^{-24}	0.086	24918	*SLC16A10*	—	3'UTR
Glucose	rs878521	7	44255643	A/G	0.22	0.09	0.01	8.0×10^{-14}	0.016	21020	*GCK*	—	TFBS
Creatinine	rs10265221	7	151414329	C/T	0.24	0.07	0.01	7.5×10^{-11}	0.32	24805	*PRKAG2*	—	Intron
Glycine	rs2169387	8	9181395	G/A	0.87	-0.13	0.02	1.3×10^{-16}	0.99	18729	*PPP1R3B*	*Processed pseudogene*	—
Glutamine	rs7078003	10	99359412	T/C	0.2	0.07	0.01	3.0×10^{-10}	0.014	24459	*HOGA1*	—	Intron/3'UTR
Glucose	rs10466351	11	92697981	T/C	0.38	0.07	0.01	3.3×10^{-13}	0.042	24676	*MTNR1B*	—	—
Glutamine	rs2657879#	12	56865338	G/A	0.18	-0.22	0.01	3.3×10^{-70}	0.11	24458	*GLS2*	—	Missense
Phenylalanine	rs1718309	12	103242396	G/A	0.6	-0.08	0.01	2.5×10^{-15}	0.07	22660	*PAH*	—	Intron
Glycoprotein acetyls	rs28929474#	14	94844947	T/C	0.02	-0.29	0.04	2.7×10^{-13}	0.17	19267	*SERPINA1*	—	Missense
Creatinine	rs61524473	15	45646283	C/T	0.26	0.09	0.01	1.2×10^{-15}	0.51	24805	*GATM*	*GATM, SPAT5L1, LOC101928414*	—
Glycoprotein acetyls	rs77303550	16	72079657	T/C	0.19	0.15	0.01	4.0×10^{-30}	0.86	19267	*HP*	—	Intron;TXNL4B
Citrate	rs172642	17	6595398	C/A	0.48	0.08	0.01	4.8×10^{-18}	0.5	24767	*SLC13A5*	*DVL2*	Intron
Creatinine	rs2079742	17	59465697	C/T	0.18	0.1	0.01	8.6×10^{-14}	0.46	22575	*BCAS3*	—	Intron
Citrate	rs2040771	22	19161935	T/C	0.48	-0.09	0.01	1.3×10^{-22}	0.36	24769	*SLC25A1*	*SLC25A1*	TFBS

In lipoprotein abbreviations, first is given the size category if applicable: XL=extra large, L=large, M=medium, S=small and XS=extra small. Then the lipoprotein particle is given: VLDL=very-low-density lipoprotein particle, IDL= intermediate-density lipoprotein particle, LDL=low-density lipoprotein particles and HDL=high-density lipoprotein particle and finally the lipid measure of the particle: C=total cholesterol, D=the mean diameter of the particle, FC=free cholesterol, L=total lipids, P=particle concentration, PL=phospholipids, TG= triglycerides. In lipids and small molecules: Bis.DB.ratio = Ratio of bis-allylic bonds to double bonds in lipids, otPUFA= Other polyunsaturated fatty acids than 18:2, ea= effect allele, nea=non-effect allele, Eaf=effect allele frequency, Beta=effect estimate, SE=standard error, eQTL=expression quantitative trait locus from GTEx, # = missense variant. TFBS: transcription factor binding site and Q: Heterogeneity statistics. If the SNP is located in an intron of a different gene than the candidate, then the gene is presented in the Function column after semicolon. Full list of abbreviations can be found from **Supplementary Table 1**

Supplementary Table 5. GTEx eQTLs

Tissue	SNP	Ensembl Gene ID	Gene type	UniProt Gene Name	beta	t-stat	p-value	FDR	pheno	chromosome	rs_number	position	gene	novelty	function	MAF
Muscle_skeletal	chr1:109818158:I	ENSG00000143126	protein_coding	CELSR2	-0.4642675	-5.624193	1.29E-07	0.000128	L.LDL.FC	1	chr1:109818	109818158	SORT1	old	TFBS_ins	0.215503
Heart_Left_Ventricle	rs9637599	ENSG00000246375	lincRNA	101929118	0.92322468	9.58844052	7.38E-14	1.43E-10	Val	4	rs9637599	89206230	PPM1K	old	TFBS	0.46958
Heart_Left_Ventricle	rs9637599	ENSG00000207480	misc_RNA		0.62357991	5.83980989	2.07E-07	0.00020037	Val	4	rs9637599	89206230	PPM1K	old	TFBS	0.46958
Thyroid	rs9637599	ENSG00000246375	lincRNA	101929118	0.56862444	4.95380022	3.72E-06	0.00140644	Val	4	rs9637599	89206230	PPM1K	old	TFBS	0.46958
Artery_Tibial	rs9637599	ENSG00000246375	lincRNA	101929118	0.6217407	6.17461482	1.82E-08	1.33E-05	Val	4	rs9637599	89206230	PPM1K	old	TFBS	0.46958
Nerve_Tibial	rs9637599	ENSG00000246375	lincRNA	101929118	1.02111658	10.7500861	3.16E-16	6.81E-13	Val	4	rs9637599	89206230	PPM1K	old	TFBS	0.46958
Nerve_Tibial	rs9637599	ENSG00000207480	misc_RNA		0.57966837	4.98798945	4.58E-06	0.00197816	Val	4	rs9637599	89206230	PPM1K	old	TFBS	0.46958
Whole_blood	rs2921604	ENSG00000154122	protein_coding	ANKH	-0.2541829	-4.280844	3.51E-05	0.01774489	Cit	5	rs2921604	14867948	ANKH	old	intron	0.444373
Heart_Left_Ventricle	rs2731672	ENSG00000169223	protein_coding	LMAN2	0.25785696	4.18379027	9.19E-05	0.02537323	Phe	5	rs2731672	176842474	F12	old	NA	0.259219
Thyroid	rs73066442	ENSG00000105877	protein_coding	DNAH11	-0.8147978	-6.2407472	1.70E-08	1.28E-05	L.LDL.PL	7	rs73066442	21592973	DNAH11	old	intron	0.245779
Heart_Left_Ventricle	rs17145750	ENSG00000009950	protein_coding	MLXIPL	0.79120012	3.97243976	0.00018794	0.04036603	VLDL.D	7	rs17145750	73026378	MLX1PL	old	intron	0.148692
Thyroid	rs17145750	ENSG00000009950	protein_coding	MLXIPL	1.0881624	6.86526984	1.07E-09	1.21E-06	VLDL.D	7	rs17145750	73026378	MLX1PL	old	intron	0.148692
Skin_sun_exposed_lower_leg	rs17145750	ENSG00000009950	protein_coding	MLXIPL	0.77159537	4.99376257	3.75E-06	0.00412804	VLDL.D	7	rs17145750	73026378	MLX1PL	old	intron	0.148692
Thyroid	rs2169387	ENSG00000253697	processed_pseudogene		-0.484505	-5.2509556	1.12E-06	0.00050933	Gly	8	rs2169387	9181395	PPP1R3B	old	NA	0.13332
Heart_Left_Ventricle	rs174547	ENSG00000134824	protein_coding	FADS2	0.63735921	5.51966948	7.09E-07	0.00045697	otPUFA	11	rs174547	61570783	FADS1	old	Intron-FADS1	0.402707
Heart_Left_Ventricle	rs174547	ENSG00000256443	lincRNA		0.60464182	4.25482322	7.20E-05	0.02318245	otPUFA	11	rs174547	61570783	FADS1	old	Intron-FADS1	0.402707
Muscle_skeletal	rs174547	ENSG00000134824	protein_coding	FADS2	0.3522459	4.66225052	8.36E-06	0.00331788	otPUFA	11	rs174547	61570783	FADS1	old	Intron-FADS1	0.402707
Nerve_Tibial	rs174547	ENSG00000134824	protein_coding	FADS2	0.49602567	4.93549184	5.58E-06	0.00200845	otPUFA	11	rs174547	61570783	FADS1	old	Intron-FADS1	0.402707
Whole_blood	rs174547	ENSG00000134824	protein_coding	FADS2	0.5078831	6.33037234	3.36E-09	6.80E-06	otPUFA	11	rs174547	61570783	FADS1	old	Intron-FADS1	0.402707
Heart_Left_Ventricle	rs7954638	ENSG00000139344	protein_coding	AMDHD1	0.5876378	4.1401397	0.00010668	0.02577536	His	12	rs7954638	96314795	HAL	new	intron-CCDC38	0.482356
Thyroid	rs7954638	ENSG00000139344	protein_coding	AMDHD1	0.55002097	4.52452761	1.97E-05	0.00640156	His	12	rs7954638	96314795	HAL	new	intron-CCDC38	0.482356
Artery_Tibial	rs7954638	ENSG00000139344	protein_coding	AMDHD1	0.48807644	4.28572368	4.52E-05	0.01558644	His	12	rs7954638	96314795	HAL	new	intron-CCDC38	0.482356
Muscle_skeletal	rs7954638	ENSG00000139344	protein_coding	AMDHD1	0.73453433	7.84045274	2.31E-12	4.58E-09	His	12	rs7954638	96314795	HAL	new	intron-CCDC38	0.482356
Heart_Left_Ventricle	rs61524473	ENSG00000259520	antisense	LOC101928414	-0.731142	-4.7842257	1.10E-05	0.00531813	Crea	15	rs61524473	45646283	GATM	old	NA	0.272263
Thyroid	rs61524473	ENSG00000259520	antisense	LOC101928414	-0.853445	-7.8694479	1.11E-11	2.51E-08	Crea	15	rs61524473	45646283	GATM	old	NA	0.272263
Thyroid	rs61524473	ENSG00000171763	protein_coding	SPATA5L1	0.57982168	4.46326719	2.49E-05	0.00706289	Crea	15	rs61524473	45646283	GATM	old	NA	0.272263
Artery_Tibial	rs61524473	ENSG00000259520	antisense	LOC101928414	-0.6890699	-6.1617995	1.93E-08	1.33E-05	Crea	15	rs61524473	45646283	GATM	old	NA	0.272263
Artery_Tibial	rs61524473	ENSG00000259433	processed_pseudogene		-0.494388	-4.8112902	5.92E-06	0.00306431	Crea	15	rs61524473	45646283	GATM	old	NA	0.272263
Artery_Tibial	rs61524473	ENSG00000171763	protein_coding	SPATA5L1	0.45453552	4.62624266	1.23E-05	0.00508763	Crea	15	rs61524473	45646283	GATM	old	NA	0.272263
Lung	rs61524473	ENSG00000259520	antisense	LOC101928414	-0.6693657	-6.0209232	3.02E-08	6.74E-05	Crea	15	rs61524473	45646283	GATM	old	NA	0.272263
Muscle_skeletal	rs61524473	ENSG00000171766	protein_coding	GATM	0.44907279	5.00640568	1.98E-06	0.00130904	Crea	15	rs61524473	45646283	GATM	old	NA	0.272263
Muscle_skeletal	rs61524473	ENSG00000259520	antisense	LOC101928414	-0.5024898	-4.9373017	2.66E-06	0.00131758	Crea	15	rs61524473	45646283	GATM	old	NA	0.272263
Nerve_Tibial	rs61524473	ENSG00000259520	antisense	LOC101928414	-0.7743297	-6.3368806	2.28E-08	2.46E-05	Crea	15	rs61524473	45646283	GATM	old	NA	0.272263
Nerve_Tibial	rs61524473	ENSG00000259433	processed_pseudogene		-0.6584493	-5.5152584	6.04E-07	0.00043483	Crea	15	rs61524473	45646283	GATM	old	NA	0.272263
Whole_blood	rs61524473	ENSG00000171763	protein_coding	SPATA5L1	0.37890372	5.87204221	3.18E-08	3.21E-05	Crea	15	rs61524473	45646283	GATM	old	NA	0.272263
Skin_sun_exposed_lower_leg	rs11644601	ENSG00000183793	protein_coding	NPIPA5	0.50805101	4.72138515	1.07E-05	0.00782577	Bis.DB.ratio	16	rs11644601	15172118	PDXC1	old	intron	0.277297
Muscle_skeletal	rs10083777	ENSG00000166454	protein_coding	ATMIN	0.37275208	4.38231689	2.58E-05	0.00851883	Gly	16	rs10083777	81065282	GCSH	new	TFBS	0.172891
Nerve_Tibial	rs10083777	ENSG00000140905	protein_coding	GCSH	-0.6454039	-4.6893123	1.39E-05	0.00429704	Gly	16	rs10083777	81065282	GCSH	new	TFBS	0.172891
Whole_blood	rs10083777	ENSG00000261061	sense_intronic	LOC102724325	-0.4315857	-4.022807	9.53E-05	0.03856528	Gly	16	rs10083777	81065282	GCSH	new	TFBS	0.172891
Skin_sun_exposed_lower_leg	rs172642	ENSG00000004975	protein_coding	DVL2	-0.3277284	-4.331814	4.52E-05	0.02491013	Cit	17	rs172642	6595398	SLC13A5	new	intron	0.480953
Heart_Left_Ventricle	rs2040771	ENSG00000100075	protein_coding	SLC25A1	-0.3678193	-4.3533879	5.11E-05	0.01975487	Cit	22	rs2040771	19161935	SLC25A1	old	TFBS	0.476266
Thyroid	rs2040771	ENSG00000100075	protein_coding	SLC25A1	-0.5097302	-5.5108741	3.84E-07	0.00021771	Cit	22	rs2040771	19161935	SLC25A1	old	TFBS	0.476266
Artery_Tibial	rs2040771	ENSG00000100075	protein_coding	SLC25A1	-0.4771556	-6.5316593	3.65E-09	7.56E-06	Cit	22	rs2040771	19161935	SLC25A1	old	TFBS	0.476266
Lung	rs2040771	ENSG00000100075	protein_coding	SLC25A1	-0.3534833	-5.3593401	5.55E-07	0.00061845	Cit	22	rs2040771	19161935	SLC25A1	old	TFBS	0.476266
Nerve_Tibial	rs2040771	ENSG00000100075	protein_coding	SLC25A1	-0.5105157	-5.1753537	2.25E-06	0.00121393	Cit	22	rs2040771	19161935	SLC25A1	old	TFBS	0.476266
Skin_sun_exposed_lower_leg	rs2040771	ENSG00000100075	protein_coding	SLC25A1	-0.4374226	-5.4723843	5.63E-07	0.00124038	Cit	22	rs2040771	19161935	SLC25A1	old	TFBS	0.476266
Whole_blood	rs2040771	ENSG00000100075	protein_coding	SLC25A1	-0.1892798	-5.1889422	7.58E-07	0.00051143	Cit	22	rs2040771	19161935	SLC25A1	old	TFBS	0.476266

Supplementary Table 6. Genetic risk score for Lp(a)

log(LPA) SNP associations in FINRISK97

ID	NEA	EA	Estimate	SE	P
rs10455872	A	G	1.29083958	0.03524657	7.66E-260
rs112092923	A	G	0.39071088	0.04693155	1.08E-16
rs117052562	G	A	0.57207416	0.04576285	2.53E-35
rs11756060	C	T	0.316227	0.03716715	2.32E-17
rs117898561	T	A	0.40094462	0.0577513	4.35E-12
rs190981730	G	A	0.75985504	0.07922578	1.35E-21
rs192776464	G	A	-0.5744705	0.04947457	9.07E-31
rs200319086	TA	T	-0.1480954	0.01382495	1.75E-26
rs201673688	G	GA	0.21289994	0.02607088	4.01E-16
rs2504921	T	G	-0.2855298	0.0141451	4.09E-87
rs41266385	A	C	0.5008693	0.03393023	2.76E-48
rs41267809	A	G	-0.3995413	0.04080005	1.94E-22
rs56393506	C	T	0.45768525	0.02010824	4.12E-109
rs71033586	AC	A	2.32579404	0.06692013	7.87E-237
rs783147	G	A	-0.1803628	0.01403455	3.34E-37
rs79018195	T	C	1.48259637	0.10269944	2.68E-46
rs79601080	A	G	-0.6400112	0.0758658	4.27E-17
rs9355839	G	A	0.22495482	0.02378786	4.79E-21
Sex			0.14549432	0.01666602	3.44E-18
Age			0.00313083	0.00063022	7.00E-07

log(LPA) SNP associations in YFS

ID	Estimate	SE	P
rs10455872	1.0902468	0.08211059	1.27E-38
rs112092923	0.67176931	0.10657425	3.57E-10
rs117052562	0.93784151	0.09257205	1.47E-23
rs11756060	0.3144094	0.07943694	7.82E-05
rs117898561	0.61401742	0.13035034	2.64E-06
rs190981730	0.7208449	0.19191886	1.78E-04
rs192776464	-1.0936299	0.10528971	1.19E-24
rs200319086	-0.2222792	0.02979609	1.28E-13
rs201673688	0.32991772	0.05630624	5.42E-09
rs2504921	-0.4711327	0.0298661	1.53E-43
rs41266385	1.01076523	0.07129889	2.54E-09
rs41267809	-0.723179	0.12080486	8.06E-80
rs56393506	0.83603685	0.04222552	3.21E-73
rs71033586	2.59944904	0.13776413	7.67E-29
rs783147	-0.3016177	0.02899995	2.39E-13
rs79018195	1.78164975	0.15738207	3.46E-07
rs79601080	-1.1458333	0.1553635	6.26E-53
rs9355839	0.25186722	0.04925064	1.04E-24
Sex	-0.0123919	0.03570944	7.29E-01
Age	0.00254736	0.00355268	4.73E-01

EA	Effect allele
NEA	Non-effect allele
SE	Standard error
P	P-value

Supplementary Table 7. For the Dutch NMR method the most representative chemical shift values for the metabolic measures in the NMR spectra are shown (many metabolites have a complicated signal with components at multiple chemical shift locations). The representative coefficients of variation (in percent) are calculated for each metabolic measure based on 174 repeated quality control serum samples that are processed identically with the actual study samples; distributed randomly among the boxes represent the averaged situation for the total 7628 samples analysed.

Method Used for: ERF, NTR, LLS

Measure	CS / ppm	CV / %
Leucine	0.95	3.91
Isoleucine	1.00	4.45
Valine	1.03	3.96
3-Hydroxybutyrate	1.19	6.49
Lipids (CH2)	1.27	6.17
Alanine	1.47	4.38
Acetate	1.91	8.26
Pyruvate / Oxaloacetate	2.36	5.52
Glutamine	2.47	11.00
Citrate	2.53	5.88
Glycine	3.55	5.97
Glycerol	3.56	18.67
Creatinine	4.05	5.08
Lactate	4.10	4.68
Glucose	5.23	3.33
Lipids (CH=CH*CH2CH2)	5.30	12.01
Tyrosine	6.89	6.41
Phenylalanine	7.42	10.53

Supplementary Note 1: Sample descriptions

1. FINRISK
FINRISK surveys are cross-sectional, population-based studies conducted every five years since 1972 to monitor the risk of chronic diseases. For each survey, a representative random sample was selected from 25- to 74-year-old inhabitants of different regions in Finland. The survey included a questionnaire and a clinical examination, at which a blood sample was drawn, with linkage to national registers of cardiovascular and other health outcomes. The study protocol has been described elsewhere [1]. Study participants were followed up through December 31, 2012. The current study included eligible individuals from FINRISK surveys conducted in 1992, 1997, 2002, and 2007 (total n=27 838). FINRISK samples were genotyped in three separate batches: DILGOM, PredictCVD and FINRISK. All three batches were analyzed separately to avoid batch effects and meta-analyzed.

2. Helsinki Birth Cohort Study (HBCS)
The primary aim of the Helsinki Birth Cohort Study was to assess how growth and environmental factors acting during the fetal period and childhood are related to health in adult life. A particular focus has been the study of the early life origins of cardiovascular disease and its risk factors. Participants were born between 1934 and 1944 in Helsinki. An essential component of the project was a detailed clinical examination of over 2500 volunteers, parts of which provided data included in this study [2].

3. Health2000 GenMets Study (GenMets)
The GenMets sample has been described in detail previously by Perttilä et al.[3]. Individuals in GenMets are metabolic syndrome cases and matched controls drawn from the Finnish Health2000 study; a population health survey. Persons with known diabetes were excluded. Participants included in the present study had participated in an additional survey, which included drawing fasting serum samples.

4. Northern Finland Birth Cohort 1966 (NFBC1966)
The NFBC1966 has been described in detail previously by Rantakallio[4]. The original study design focused on factors affecting pre-term birth, low birth weight, and subsequent morbidity and mortality. Mothers living in the two northern-most provinces of Finland were invited to participate if they had expected delivery dates during 1966. Individuals still living in the Helsinki area or Northern Finland (N = 5923) were asked at age 31 to participate in a detailed biological and medical examination as well as a questionnaire.

5. Cardiovascular Risk in Young Finns Study (YF)
The Cardiovascular Risk in Young Finns Study (YF) is a population based prospective cohort study. It was conducted at five medical schools in Finland (Turku, Helsinki, Kuopio, Tampere and Oulu), with the aim of studying the levels of cardiovascular risk factors in children and adolescents in different parts of the country. The latest follow-up was conducted in 2007. The serum samples for this metabolomics study were collected at this latest follow up. The study and data collection protocols have been described in detail by Raitakari et al.[5].

6. Finnish twin cohort
Individuals in this study were derived from the FinnTwin12 (FT12) and FinnTwin16 (FT16) cohort studies. In brief, the FT12 is a population-based cohort longitudinal study of five consecutive birth cohorts of Finnish twins born between 1983 and 1987. All twins (and their parents) were initially contacted and invited to participate by mail in the autumn of the year in which their birth cohort

reached 11 years of age. Subsequent follow-up assessments were made when the twins were aged 14, 17 and ~22 years. The FT16 is a population-based longitudinal study of five consecutive birth cohorts of Finnish twins born between 1975 and 1979. Each pair was initially approached and invited to participate by mail in the 1-2 months following the twins' 16th birthday. The baseline data collection started in 1991 and was completed in 1996, with participation of 2733 twin pairs (response rate ~ 88%). Subsequent follow-up assessments were made when the twins were 17, 18.5, and ~25 years, following a similar approach. For both the FT12 and FT16, the baseline and follow-up assessments included surveys of health habits and attitudes, symptom checklists, personality scales, and social relationships [6]. In addition, blood samples were taken from all twins during a visit to the twin research clinic in Helsinki (Finland) at the last follow-up (young adulthood) for DNA and biochemistry analyses. Data collection and analysis were approved by the ethics committees of the Department of Public Health of the University of Helsinki, the Helsinki and Uusimaa Hospital District and the IRB of Indiana University. Written informed consent was obtained from all participating twins.

7. ERF
The Erasmus Rucphen Family (http://www.erasmusmc.nl/klinische_genetica/research/intro/genepi/) genetic isolate study is a family based study in a genetically isolated population in the Southwest of the Netherlands. This young genetic isolate was founded in the mid - 18th century and minimal immigration and marriages have occurred between surrounding settlements due to social and religious reasons. The ERF population includes 3,465 individuals that are living descendants of 22 couples with at least six children baptized[7]. The study protocol was approved by the medical ethics board of the Erasmus MC Rotterdam, the Netherlands.

8. EGCUT
The Estonian Biobank is the population-based biobank of the Estonian Genome Center of the University of Tartu (EGCUT). The project is conducted according to the Estonian Gene Research Act and all participants have signed broad informed consent[8]. The cohort size is currently 51535 people aged 18 years and older. All subjects are volunteers and were recruited randomly by general practitioners (GP) and physicians in hospitals. A Computer Assisted Personal Interview is conducted at the doctor's office to record personal data (place of birth, place(s) of living, nationality etc.), genealogical data (family history spanning four generations), educational and occupational history, lifestyle data (physical activity, dietary habits - FFQ, smoking, alcohol consumption, women's health, quality of life). Metabolite concentrations (over 110; NMR and MS) are available for about 12000 subjects; metabolic follow-up has been conducted for more than 2000 donors. The EGCUT database has been linked with the national registries and hospital databases for obtaining up-to-date phenotypic information. Medical history and current health status are recorded according to the ICD-10, medication according to the ATC. Anthropometric measurements, blood pressure (sitting position at the end of the interview), and resting heart rate are measured; 30-50 mL of venous blood are collected into EDTA Vacutainers. These are transported to the central laboratory of the EGCUT at +4…+6 C (in 6 to 36h) where DNA, plasma and WBC are immediately isolated and kept in aliquots in MAPI straws in liquid N_2. All procedures are run according to ISO 9000-2008 and LIMS.

9. KORA
KORA (Cooperative Health Research in the Region of Augsburg; http://www.helmholtz-muenchen.de/en/kora-en/kora-homepage/index.html) exists since 1996 in the region of Augsburg in the southwest of Germany, and builds on the MONICA (Monitoring of trends and determinants in cardiovascular disease) project initiated in 1984[9]. KORA is a regional research platform for population-based surveys and a cohort of more than 18,000 subjects are actively followed up until

the present date. Four cross-sectional health surveys have been performed in five-year intervals, each containing independent random samples of residents in the city of Augsburg and the two adjacent counties in the age-range between 25 to 74 years at baseline examination. The study followed the recommendations of the Declaration of Helsinki and was approved by the local ethical committees and informed consent was given by all participants. Blood was drawn in the morning between 8 and 10am after a period of overnight fasting. Material was immediately horizontal shaken (10 min), followed by 40 min resting at 4°C to obtain complete coagulation. The material was then centrifuged (2000 × g; 4°C). Serum was aliquoted and stored for 2–4 hours at 4°C, after which it was deep frozen to -80°C until sampling.

10. LLS
In the Leiden Longevity Study (http://www.molepi.nl/research/longevity), nonagenarian sibling pairs were included when aged older than 89 years for men and 91 years for women. Additional inclusion criteria were that participants needed to have at least one sister or a brother fulfilling these age criteria, and who was also willing to participate[10]. Because proper controls are lacking at very high ages, the offspring of the nonagenarian siblings were asked to be included in the study as well. The partners thereof were included in the study to serve as a control group, representing the general population at an age comparable to the offspring. The total study population, excluding the nonagenarian siblings, consisted of 2,415 participants (1,671 offspring; 744 partners). The Medical Ethical Committee of the Leiden University Medical Centre approved the study and informed consent was obtained from all participants. Blood samples were taken at baseline for extraction of DNA and the determination of non-fasted serum parameters. For metabolomics measurements, blood was drawn using a safety lock butterfly needle. Within two hours tubes with clotted blood were centrifuged for 15 minutes at 2,800 x g and serum was obtained and divided into subsamples, snap-frozen and stored at –80° C.

11. NTR
The Netherlands Twin Register (NTR; http://www.tweelingenregister.org/) recruits twins and their family members to study the causes of individual differences in health, behavior and lifestyle. Participants are followed longitudinally; details about the cohort have been published previously[11]. A subsample of unselected twins and their family members has taken part in the NTR-Biobank project. Genotyping was performed on the Affymetrix 6.0 (N=298), Affymetrix Perlegen 5.0 (N=3,697), Illumina 370 (N=290), Illumina 660 (N=1,439) and Illumina Omni Express 1M (N=455) platforms. Calls were made with the platform specific software (Genotyper, Beadstudio). Per platform the quality control thresholds for SNPs were minor allele frequency (MAF) > 1%, Hardy-Weinberg equilibrium (HWE) P value > 0.00001, call rate >95%, and 0.30 < heterozygosity < 0.35. Samples were excluded from the data if their expected sex and identity by descent (IBD) status did not match, or if the genotype missing rate was above 10%. For each platform all SNPs were aligned to the positive strand of the Hapmap 2 Build 36 release 24 CEU reference set. The alignment was checked using individuals and family members tested on multiple platforms. SNPs were excluded per platform if allele frequencies differed more than 15% with the reference set and/or the other platforms. The data of the platforms were subsequently merged into a single dataset (N=5,856). This merged set was imputed against the reference set using IMPUTE v2. After imputation, genotype dosage was calculated if the highest genotype probability was above 90%. Badly imputed SNPs were removed based on HWE P value < 0.00001, proper info < 0.40, MAF < 1%, allele frequency difference > 0.15 against reference. From the total set of 5,856 individuals with imputed GWA data, a subset of unrelated participants were included in the present study. For metabolomics measurements, blood was collected in the morning using a safety lock butterfly needle.

Supplementary Note 2: Additional acknowledgements

The Estonian Genome Center of University of Tartu (EGCUT) received support from EU FP7 grant BBMRI-LPC 313010, targeted financing from Estonian Government IUT20-60, IUT24-6, Estonian Research Roadmap through the Estonian Ministry of Education and Research (3.2.0304.11-0312), Center of Excellence in Genomics (EXCEGEN), Development Fund from the University of Tartu (SP1GVARENG) and from an EFSD New Horizons grant. EGCUT thanks Viljo Soo Estonian Genome Center for his help on this project. This work was also supported by the US National Institute of Health [R01DK075787].

The Leiden Longevity Study (LLS) has received funding from the European Union's Seventh Framework Programme (FP7/2007-2011) under grant agreement n° 259679. This study was supported by a grant from the Innovation-Oriented Research Program on Genomics (SenterNovem IGE05007), the Centre for Medical Systems Biology, and the Netherlands Consortium for Healthy Ageing (grant 050-060-810), all in the framework of the Netherlands Genomics Initiative, Netherlands Organization for Scientific Research (NWO), Unilever Colworth and by BBMRI-NL, a Research Infrastructure financed by the Dutch government (NWO 184.021.007).

Netherlands Twin Registry (NTR) Research was funded by the Netherlands Organization for Scientific Research (NWO: MagW/ZonMW grants 904-61-090, 985-10-002,904-61-193,480-04-004, 400-05-717, Addiction-31160008 Middelgroot-911-09-032, Spinozapremie 56-464-14192), Center for Medical Systems Biology (CSMB, NWO Genomics), NBIC/BioAssist/RK(2008.024), Biobanking and Biomolecular Resources Research Infrastructure (BBMRI –NL, 184.021.007), the VU University's Institute for Health and Care Research (EMGO+), the European Community's Seventh Framework Program (FP7/2007-2013), ENGAGE (HEALTH-F4-2007-201413) and the European Science Council (ERC - 230374 and ERC-284167). HHMD is supported by an EMGO+ Fellowship as part of the Mental Health research program of the EMGO Institute for Health and

Care Research. NTR thanks all participants in the Netherlands Twin Register. This work was carried out on the Dutch national e-infrastructure with the support of SURF Foundation.

Erasmus Rucphen Family (ERF) was supported by the Consortium for Systems Biology (NCSB), both within the framework of the Netherlands Genomics Initiative (NGI)/Netherlands Organisation for Scientific Research (NWO). ERF study as a part of EUROSPAN (European Special Populations Research Network) was supported by European Commission FP6 STRP grant number 018947 (LSHG-CT-2006-01947) and also received funding from the European Community's Seventh Framework Programme (FP7/2007-2013)/grant agreement HEALTH-F4-2007-201413 by the European Commission under the programme "Quality of Life and Management of the Living Resources" of 5th Framework Programme (no. QLG2-CT-2002-01254) as well as FP7 project EUROHEADPAIN (nr 602633). High-throughput analysis of the ERF data was supported by joint grant from Netherlands Organisation for Scientific Research and the Russian Foundation for Basic Research (NWO-RFBR 047.017.043). Exome sequencing analysis in ERF was supported by the ZonMw grant (2010/31471/ZONMW). The funders had no role in study design, data collection and analysis, decision to publish, or preparation of the manuscripts. ERF study is grateful to all study participants and their relatives, general practitioners and neurologists for their contributions and to P. Veraart for her help in genealogy, J. Vergeer for the supervision of the laboratory work and P. Snijders for his help in data collection.

The Cardiovascular Risk in Young Finns Study (YFS) The Young Finns Study has been financially supported by the Academy of Finland: grants 134309 (Eye), 126925, 121584, 124282, 129378 (Salve), 117787 (Gendi), and 41071 (Skidi); the Social Insurance Institution of Finland; Kuopio, Tampere and Turku University Hospital Medical Funds (grant X51001 for T.L.); Juho Vainio Foundation; Paavo Nurmi Foundation; Finnish Foundation of Cardiovascular Research (T.L.);

Finnish Cultural Foundation; Tampere Tuberculosis Foundation (T.L.); Emil Aaltonen Foundation (T.L.); and Yrjö Jahnsson Foundation (T.L.).

Helsinki Birth Cohort Study (HBCS) has been supported by grants from the Academy of Finland (120386, 125876 and 126775 to J.E.), the Finnish Diabetes Research Society, the Novo Nordisk Foundation, the European Science Foundation (EuroSTRESS), the Wellcome Trust (89061/Z/09/Z and 089062/Z/09/Z), the Samfundet Folkhälsan and the Finska Läkaresällskapet.

Northern Finland Birth Cohort 1966 (NFBC1966) received financial support from the Academy of Finland (104781, 120315, 129269, 1114194, 139900 and SALVE to M.-R.J.) University Hospital Oulu, Biocenter, University of Oulu (75617 to M.-R.J. and M.S.), the European Commission EURO-BLCS Framework 5 award (QLG1-CT-2000-01643 to M.-R.J.), the US National Heart, Lung, and Blood Institute (NHLBI) (5R01HL087679), the US National Institute of Mental Health (NIMH) (1RL1MH083268), European Network for Genetic and Genomic Epidemiology (ENGAGE) (HEALTH-F4-2007-201413 to L.P. and M.-R.J.), the MRC UK (G0500539, G0600705 and PrevMetSyn/Salve to M.-R.J.) and the Wellcome Trust (GR069224).

Finnish Twin Cohort Study Data collection for FinnTwin12 and FinnTwin16 were supported by the National Institute on Alcohol Abuse and Alcoholism (NIAAA) (AA-12502, AA-09203 and AA-08315 to R.J.Rose) and the Academy of Finland (100499, 205585, 118555, 265240, 263278 and 141054 (Skidi-Kids) to J. Ka). L.H.B was supported by 1.3 milj. klubi-klubben. Genotyping was performed at the Wellcome Trust Sanger Institute.

FINRISK The FINRISK/DILGOM study was supported by the Academy of Finland (118081) and by the European Union Seventh Framework Programme (FP7/2007-2013) under grant agreement No. HEALTH-F2-2011-278913 (BiomarCaRE). Abbott Diagnostics provided test reagents for Lp(a). Academy of Finland supported V.S. (grant #139635), M.P. (grant #269517) and J.K. (grant #283045) and V.S. was supported by Finnish Foundation for Cardiovascular Research. M.P. was supported also through EU FP7 under grant agreements nr. 313010 (BBMRI-LPC), nr. 305280

(MIMOmics), and nr. 261433 (BioSHaRE-EU), the Yrjö Jahnsson Foundation and Juho Vainio Foundation.

KORA The KORA study was initiated and financed by the Helmholtz Zentrum München – German Research Center for Environmental Health, which is funded by the German Federal Ministry of Education and Research (BMBF) and by the State of Bavaria. Furthermore, KORA research was supported within the Munich Center of Health Sciences (MC-Health), Ludwig-Maximilians-Universität, as part of LMUinnovativ. This work was financed by the European Community's Seventh Framework Programme (FP7/2007-2013), ENGAGE project (HEALTH-F4-2007-201413), and the German National Genome Research Network (NGFN; NGFNPlus, project number 01GS0834). We thank Julia Scarpa and Katharina Sckell for metabolomics measurements performed at the Helmholtz Centrum München, Genome Analysis Center, Metabolomics Core Facility. Karsten Suhre is supported by Biomedical Research Program funds at Weill Cornell Medical College in Qatar, a program funded by the Qatar Foundation. Christian Gieger is supported by the European Union's Seventh Framework projects MIMOmics (FP7-Health-F5-2012-305280) and PAIN-OMICS (project nr. 602736), by the BMBF project e:AtheroSysMed (project nr.: 01ZX1313G) and by the Helmholtz-Russia joint research group HRJRG-310.

The quantitative NMR metabolomics platform and its development has been supported by the Academy of Finland, TEKES - the Finnish Funding Agency for Technology and Innovation, the Sigrid Juselius Foundation, the Novo Nordisk Foundation, the Finnish Diabetes Research Foundation, the Paavo Nurmi Foundation, and the strategic and infrastructural research funding from the University of Oulu, Finland, as well as by the British Heart Foundation, the Welcome Trust, and the Medical Research Council, UK.

GTEx: The Genotype-Tissue Expression (GTEx) Project was supported by the Common Fund of the Office of the Director of the National Institutes of Health. Additional funds were provided by the NCI, NHGRI, NHLBI, NIDA, NIMH, and NINDS. Donors were enrolled at Biospecimen

Source Sites funded by NCI\SAIC-Frederick, Inc. (SAIC-F) subcontracts to the National Disease Research Interchange (10XS170), Roswell Park Cancer Institute (10XS171), and Science Care, Inc. (X10S172). The Laboratory, Data Analysis, and Coordinating Center (LDACC) was funded through a contract (HHSN268201000029C) to The Broad Institute, Inc. Biorepository operations were funded through an SAIC-F subcontract to Van Andel Institute (10ST1035). Additional data repository and project management were provided by SAIC-F (HHSN261200800001E). The Brain Bank was supported by a supplements to University of Miami grants DA006227 & DA033684 and to contract N01MH000028. Statistical Methods development grants were made to the University of Geneva (MH090941 & MH101814), the University of Chicago (MH090951, MH090937, MH101820, MH101825), the University of North Carolina - Chapel Hill (MH090936 & MH101819), Harvard University (MH090948), Stanford University (MH101782), Washington University St Louis (MH101810), and the University of Pennsylvania (MH101822). The data used for the analyses described in this manuscript were obtained from: the GTEx Portal on 12/01/2014 and/or dbGaP accession number phs000424.v3.p1 on 08/03/2013.

Supplementary References

1. Vartiainen, E. et al. Thirty-five-year trends in cardiovascular risk factors in Finland. *Int J Epidemiol* **39**, 504-18 (2010).
2. Yliharsila, H. et al. Body mass index during childhood and adult body composition in men and women aged 56-70 y. *Am J Clin Nutr* **87**, 1769-75 (2008).
3. Perttila, J. et al. OSBPL10, a novel candidate gene for high triglyceride trait in dyslipidemic Finnish subjects, regulates cellular lipid metabolism. *J Mol Med (Berl)* **87**, 825-35 (2009).
4. Rantakallio, P. Groups at risk in low birth weight infants and perinatal mortality. *Acta Paediatr Scand* **193**, Suppl 193:1+ (1969).
5. Raitakari, O.T. et al. Cohort profile: the cardiovascular risk in Young Finns Study. *Int J Epidemiol* **37**, 1220-6 (2008).
6. Kaprio, J., Pulkkinen, L. & Rose, R.J. Genetic and environmental factors in health-related behaviors: studies on Finnish twins and twin families. *Twin Res* **5**, 366-71 (2002).
7. Aulchenko, Y.S. et al. Linkage disequilibrium in young genetically isolated Dutch population. *Eur J Hum Genet* **12**, 527-34 (2004).
8. Leitsalu, L. et al. Cohort Profile: Estonian Biobank of the Estonian Genome Center, University of Tartu. *Int J Epidemiol* (2014).

9. Holle, R., Happich, M., Lowel, H., Wichmann, H.E. & Group, M.K.S. KORA--a research platform for population based health research. *Gesundheitswesen* **67 Suppl 1**, S19-25 (2005).
10. Schoenmaker, M. et al. Evidence of genetic enrichment for exceptional survival using a family approach: the Leiden Longevity Study. *Eur J Hum Genet* **14**, 79-84 (2006).
11. Willemsen, G. et al. The Netherlands Twin Register biobank: a resource for genetic epidemiological studies. *Twin Res Hum Genet* **13**, 231-45 (2010).
